# Targeted Editing and Phenotypic Profiling of *CmOFP13* Mutants Reveal Its Role in Melon Fruit Morphogenesis

**DOI:** 10.1111/ppl.70641

**Published:** 2025-11-29

**Authors:** Carlos Mayobre, María José Gonzalo, Montserrat Vergés, Guillem Guardia‐Bersabé, Dídac Jiménez‐Sánchez, Antonio José Monforte, Jordi Garcia‐Mas, Marta Pujol

**Affiliations:** ^1^ Centre for Research in Agricultural Genomics (CRAG) CSIC‐IRTA‐UAB‐UB, Edifici CRAG, Campus UAB Barcelona Spain; ^2^ Instituto de Biología Molecular y Celular de Plantas (IBMCP), Consejo Superior de Investigaciones Científicas (CSIC), Universitat Politècnica de València Valencia Spain; ^3^ Institut de Recerca i Tecnologia Agroalimentàries (IRTA), Edifici CRAG, Campus UAB Barcelona Spain

## Abstract

The melon fruit shape is a trait that influences storage as well as consumer preference. Fruit shape is known to be regulated by factors such as Ovate Family Proteins (OFPs), Tonneau Recruiting Motif proteins (TRMs), IQ67 domain proteins (IQDs) and plant hormones. *CmOFP13* (*MELO3C025206*) had been identified as a regulator of shape in melon using a map‐based cloning approach and validated by overexpression in *Arabidopsis*, generating plants with rounder leaves and shorter siliques. In this work, CRISPR‐*Cas9* was applied in melon for the functional validation of *CmOFP13*. ‘Védrantais’ (VED) seed cotyledons were transformed, obtaining diploid edited plants with a slightly elongated phenotype compared to the wild type in both ovaries and fruits. This effect is associated with the presence of larger cells in the distal area of E1 stage flowers. The analysis of the mutation indicated the loss of the OVATE domain in the edited *CmOFP13*, while maintaining the DNA‐binding domain. In addition, new candidate genes have been proposed based on phylogenetic analyses and expression data. The mutation of *OFPs* offers the possibility of new melon fruit shapes, adapting the fruit for safer storage or to consumer demands.

## 1 Introduction

Fruit shape is one of the traits that determines consumer preference, and there is considerable variability in shapes among major fruit crops such as apples, peaches, and tomatoes on the market. Melon is one of the fruits with the richest diversity in fruit shapes, ranging from spherical to oval and elongated fruits (Pitrat 2017). These differences are visible in the ovary or during early fruit development and are caused by different cell division and elongation patterns (Lazzaro et al. 2018; Martínez‐Martínez et al. 2022; Wang et al. 2025; Xiao et al. 2008).

The genetic basis of fruit shape is very complex. Several genes from different signaling pathways have been shown to contribute to the final shape (Li et al. 2023). For instance, Ovate Family Proteins (OFPs), Tonneau Recruiting Motif proteins (TRMs), and IQ67 domain (IQD) proteins (e.g., SUN), have been reported to be involved in determining fruit shape in different plant species by modifying microtubule architecture (Goldman et al. 2023; Lazzaro et al. 2018). These genes are members of large gene families with diverse functions. For example, 19 OFPs have been annotated in melon (Ruggieri et al. 2018), a similar number to those found in other cucurbits (Feng, Wu, et al. 2023; Han et al. 2022; He et al. 2024; Pan et al. 2020).

A few plant hormones have also been associated with fruit shape and length (Li et al. 2023; Wang et al. 2025). Brassinosteroids (BRs) have been described as regulators of *OFP* expression in rice (Y. Xiao et al. 2017). The gibberellin synthesis gene *AtGA20ox1* (*AT1G78440*) has been reported to be regulated by *AtOFP1* (*AT5G01840*) (Hackbusch et al. 2005; Wang et al. 2007). In cucumber, *FRUITFUL1* (*FUL1, CsaV3_1G006220*) and *HEC1* (*CsaV3_4G034440*) have been associated with auxin biosynthesis and transport in the control of fruit shape (Z. Wang et al. 2022; Zhao, Jiang, et al. 2019). Genes such as *FASCIATED/CLAVATA3* (*FAS/CLV3, Solyc11g071380*) and *LOCULE NUMBER/WUSCHEL* (*LC/WUS, Solyc02g083950*) have also been reported to influence fruit shape and size in tomato by controlling organ polarity and carpel number (Rodríguez et al. 2011).

In melon, even though many QTLs have been described playing a role in fruit shape (Gur et al. 2017; Monforte et al. 2014; Pereira et al. 2018), very little information is available on the underlying causal genes. Pan et al. (2020) proposed 32 consensus QTLs related to melon fruit shape. Within these QTLs, chromosome 2 contains *CmACS7* (*MELO3C015444*), an ethylene biosynthesis gene involved in sex determination with pleiotropic effects on ovary shape (Boualem et al. 2008, 2022; Monforte et al. 2005). On chromosome 12, *CLAVATA3* (*CmCLV3, MELO3C035640*) controls carpel number with pleiotropic effects on fruit shape as well (L. Wang et al. 2022). On chromosome 8, *fsqs8.1* has been mapped in an F2 population generated from the “Piel de Sapo” (PS) × PI 124112 cross (Díaz et al. 2014). The QTL was verified by introducing the PI 124112 *fsqs8.1* allele into the PS genetic background in the introgression line (IL) CALC8‐1 that produced round fruits. Fine mapping of the region led to the identification of *CmOFP13* (*MELO3C025206*) as a candidate gene, and heterologous overexpression in *Arabidopsis thaliana* generated plants with round leaves, flat ovaries, and shorter siliques (Martínez‐Martínez et al. 2022). Only silent mutations were found between the PI 124112 and PS *CmOFP13* alleles; however, differences in the pattern of gene expression in early ovary development correlated with variations in ovary shape: the PI 124112 allele was expressed in very early stages of ovary development, whereas in PS, it was expressed in later stages. They also found major structural variations between PI 124112 and PS at the *fsqs8.1* locus. Additionally, the effect of *fsqs8.1* in different melon genetic backgrounds has also been demonstrated (Martínez‐Martínez et al. 2022). The current hypothesis is that the variation is due to heterochronic expression of *CmOFP13*. *CmOFP13* is likely an ortholog of the tomato *SlOFP20* (*Solyc10g076180*), which regulates organ shape as part of the OFP‐TRM pathway in tomato, and is likely orthologous to *PpOFP1* (*Prupe.6G290900*), *StOFP20* (*PGSC0003DMG400030384*) and *CaOPF20* (*CA10g10680*) (Ai et al. 2023; Borovsky et al. 2022; Wu et al. 2018; Zhou et al. 2021). The OFP‐TRM pathway has been proposed as a common genetic mechanism regulating plant organ morphology in different species (Wu et al. 2018).

CRISPR/*Cas9* gene editing is widely used to validate candidate genes (Feng, Wang, et al. 2023; Gao et al. 2019). This technique has been successfully used in melon but only in a limited number of cultivars (Hooghvorst et al. 2019; Nonaka et al. 2023; Shirazi Parsa et al. 2023). Some challenges that remain to be solved are the low transformation efficiency, the existence of recalcitrant cultivars such as PS, and the high level of polyploidy obtained after in vitro culture (Nonaka et al. 2023; Shirazi Parsa et al. 2023). IL CALC8‐1 has a PS genetic background, making it difficult to edit using CRISPR/*Cas9*. Therefore, for the functional validation of *CmOFP13*, we decided to use ‘Védrantais’ for gene editing. Some studies have already achieved successful gene editing in melon ripening‐related genes such as *CmCTR1* (*MELO3C024518*), *CmROS1* (*MELO3C024516*), *CmNAC‐NOR* (*MELO3C016540*), *CmACO1* (*MELO3C014437*) and *CmERF024* (*MELO3C024520*) (Giordano et al. 2022; Liu et al. 2022; Nonaka et al. 2023; Santo Domingo et al. 2024), as well as the translation initiation factor *eIF4E* (*MELO3C002698*) (Pechar et al. 2022), the virus resistance gene *Prv* (*MELO3C022145*) (Nizan et al. 2023), and recently in a tendril‐formation gene (*CmTCP1*, *MELO3C022091*) (Li et al. 2025). The aim of this work was to conduct a functional validation of *CmOFP13* through gene editing using CRISPR/*Cas9* in melon. This will give further insight into the mechanism involved in melon organ morphology and facilitate breeding efforts to increase the catalogue of melon fruit shapes, helping to match consumers' demands and storage and transport requirements.

## 2 Material and Methods

### 2.1 Guide RNA Design and Cloning

Guide RNAs (gRNAs) were designed using the Breaking‐Cas tool (https://bioinfogp.cnb.csic.es/tools/) (Oliveros et al. 2016). *Streptococcus pyogenes* Cas9 was used, recognizing the PAM sequence 5′‐NGG‐3′. The *CmOFP13* gene sequence was extracted from the Melonomics website (https://www.melonomics.net/melonomics.html#/). The two best gRNAs were designed for cloning into the pEn‐Chimera vector with *Bsb*I (BioLabs) restriction sites, following a single‐guide strategy (Table 1) (File S1). Finally, LR clonase II (Thermo Fisher Scientific) was used to transfer the gRNAs to the pDeCAS9 vector, containing the *CAS9* gene and a Phosphinothricin (*PPT*) resistance gene (File S2). Vectors were kindly provided by Prof. Holger Puchta (Botanisches Institut, KIT, Germany). The final vector was transformed into the *Agrobacterium tumefaciens* strain Agl‐0, as described in Giordano et al. (2022).

**TABLE 1:** Primer sequences and guide RNAs used in this work.

Name	Sequence (5′‐3′)	Use and references
gRNA1	AGATAGATTCCGCCGTTGTG	Guide 1 (This work)
gRNA2	GTCAGTTCGGAAATCAGGGG	Guide 2 (This work)
RO94 (Cas9_Fw)	GGACACTTCCTCATCGAGGGT	Cas9 PCR (Liu et al. 2022)
RO95 (Cas9_Rv)	GTGGAGCCTTGGTGATCTCGG	Cas9 PCR (Liu et al. 2022)
OFP‐Pre‐Fw	GTACCCAATTTTCGGGGAGT	OFP13 sequencing (This work)
OFP‐Pre‐Rv	ATCCAACGATGCCTACTTCG	OFP13 sequencing (This work)

### 2.2 Melon Transformation and In Vitro Culture

Seeds from the elite cultivar “Védrantais” (VED) ( *Cucumis melo* spp. *melo*, group *cantalupensis*) were used as starting material for melon transformation. Fifty seeds per gRNA were used. Cotyledon transformation was according to Castelblanque et al. (2008), with some modifications, cutting the explants as described by García‐Almodóvar et al. (2017). Seed coats were removed, and the seeds were hydrated for 2 h and disinfected with 50% commercial bleach (Conejo, Henkel) for 20 min. Then the tegument was removed, and seeds were placed on MS medium agar plates for 1 day. Embryos were cut out, and the proximal part of the cotyledon was cut in half, generating four explants per seed. Explants were incubated for 20 min in the *Agrobacterium* solution with 200 μM acetosyringone (Sigma‐Aldrich), and vacuum was applied with a 50 mL syringe. The explants were dried on sterile filter paper and co‐cultured for 3 days in the dark at 28°C in agar MS regeneration media containing 0.5 mg L^−1^ 6‐bencylaminopurine (BA, Duchefa Biochemie), 0.1 mg L^−1^ indole‐3‐acetic acid (IAA, Duchefa Biochemie), and 200 μM acetosyringone (Liu et al. 2022). They were then transferred to regeneration media containing 0.5 BA, 0.1 IAA, 4 mg L^−1^ DL‐Phosphinothricin (PPT, Duchefa Biochemie) and 1 mL L^−1^ Plant Preservative Mixture (PPM, Plant Cell Technology), with a 12 h light photoperiod (17 Wm^−2^, 80 μmol m^−2^ s^−1^, PHILIPS Master TL5 HO 39 W/840). Calli were cleaned every 2–3 weeks until shoots were obtained. Individualized plants were transferred to tubes with rooting media that did not contain plant hormones. Once plants were well developed, they were acclimated for 1 week and transplanted to pots in the greenhouse.

### 2.3 PCR and Sequencing

DNA was extracted from individual growing plants according to Doyle (1991) with some modifications (Pereira et al. 2018). PCR was used to verify the presence of *Cas9* and to assess gene editing using the primers described in Table 1. After sequencing, editions were inferred by the ICE Synthego software (https://ice.editco.bio/#/) and by visual analysis of chromatograms. The ploidy level of the positive plants was determined by flow cytometry using leaf samples from Iribov (Heerhugowaard, The Netherlands).

### 2.4 Genetic and Protein Analysis

To check the putative effect of the editions, the mutated DNA sequence was submitted to the ORF finder tool (https://www.ncbi.nlm.nih.gov/orffinder/). Wild‐type and edited forms of the CmOFP13 protein were compared to *Arabidopsis*, cucumber and tomato OFPs using the MEME motif elicitation tool (https://meme‐suite.org/meme/tools/meme), adding well‐studied OFPs from potato, peach and pepper. The found motifs were searched with the NCBI Conserved Domain tool (Marchler‐Bauer et al. 2017). OFP sequences from other plant species were obtained from TAIR (https://www.arabidopsis.org/), Sol Genomics (https://solgenomics.net/), the Genome Database for Rosaceae (https://rosaceae.org) and Cucurbit Genomics Database (http://cucurbitgenomics.org/v2/). Protein sequences and gene IDs are listed in File S3. Phylogenetic analysis was performed using MEGA 11 (Tamura et al. 2021). Alignment was performed using the MUSCLE approach (Edgar 2004) and the Neighbor‐Joining (NJ) method (Saitou and Nei 1987). An N‐J phylogenetic tree was generated using a p‐distance substitution model and gamma distributed with invariant sites (*G* + *I*, *γ* = 1.0), with partial deletion as gap treatment (95% Site Coverage Cutoff), eight threads, and 1000 bootstrap iterations.

### 2.5 Plant Growth

Edited plants were first grown in pots in greenhouses at the Centre for Research in Agricultural Genomics (CRAG). Plants were pruned weekly and manually pollinated. Wild‐type and polyploid edited plants were crossed, and fruits were harvested at the fully ripe stage. To phenotype the diploid edited plants, wild‐type, edited lines and hybrids (wild‐type by mutant) were grown at three different locations: CRAG (Cerdanyola del Vallès), Torre Marimon (TM, Caldes de Montbui) and at the Instituto de Biología Molecular y Celular de Plantas (IBMCP, Valencia). At CRAG, eight biological replicates per genotype were planted in pots in autumn 2023, allowing one fruit per plant. In summer 2024, eight replicates per genotype were planted at TM in coconut fiber sacks, allowing a single fruit per plant. While at IBMCP, 10 replicates were planted in pots, allowing for one to three fruits per plant. Flower measurements were only taken at IBMCP.

### 2.6 Fruit Phenotyping

Melon flowers and fruits were harvested and cut in half along the longitudinal axis. The fruit morphological parameters fruit length (FL), fruit width (FWI), fruit shape index (FSI) and fruit area (FA) were measured using Tomato Analyzer 3.0 (Gonzalo et al. 2009).

### 2.7 Ovary Development Analysis

For the developmental study, ovaries were collected from both wild and edited plants at various stages. At least three biological replicates of each ovary stage for wild and edited plants were collected for analysis.

For the measurements at the microscopic level, flowers were collected at developmental stages E1 (carpel primordial initiated), E2–E3 (stigma and style defined and anthers with pollen) and E4 (ovule primordial initiated), corresponding to stages 6, 8–1 to 8–2 and 8–3, respectively, from Bai et al. (2004). The fresh ovaries were placed in FAE fixative solution (4% formaldehyde, 5% acetic acid, and 50% ethanol) and stored overnight at 4°C for tissue fixation. They were then dehydrated in a graded ethanol series as described in Gómez et al. (2004, 2011) prior to embedding in paraffin wax. Embedded ovaries were sectioned using a Microm HM330 microtome, and the sections were attached to polysine slides and stained with 0.02% toluidine blue. Ovary images were captured using a Leica DMS microscope equipped with the LAS X application at the microscopy services at IBMCP. Several longitudinal cuts were made for each flower, and the three cuts that corresponded to the central part of the ovary after visual inspection were selected for measurements. For the measurements at the macroscopic level, fresh flowers from stages 7 days prior to anthesis to full anthesis were collected and sectioned longitudinally. The ovarian hairs were removed to facilitate the definition of the ovary contour.

The morphological parameters ovary length (OL), ovary width (OWI), ovary constriction (OC), ovary shape (OS) and ovary distal end blockiness (OS/OWI, ODEB) were measured by ImageJ (Abràmoff et al. 2004) and Tomato Analyzer 3.0 (Gonzalo et al. 2009).

### 2.8 Ovary Cell Imaging and Analysis

For cellular analysis at the E1 developmental stage, the distal end area of the ovary, defined as the area between the end of the ovules and the constriction, was examined using images captured with a Leica DM5000 microscope. Distances along the Y‐axis (from ovules to constriction) and the X‐axis (ovary diameter) were measured by ImageJ. Cell counts were performed at the midpoint of this region along both axes, following a method similar to that described by Wu et al. (2018). Cell size was calculated by dividing the distance along each axis by the number of cells, while cell shape was calculated as the ratio of cell size in the Y‐axis to that in the X‐axis.

### 2.9 Statistical Analyses

The R language (version 4.0.3) and RStudio (v2023.03.1) were used, along with the “tidyverse,” “readxl,” “coin,” and “welchADF” packages, to generate graphs and calculate statistical values. ANOVA and *t*‐tests were used for normally distributed and homoscedastic variables, while the Kruskal–Wallis and Wilcoxon tests were used for non‐normally distributed variables.

## 3 Results

### 3.1 Generation of CRISPR/Cas9 CmOFP13 Mutants

**FIGURE 1:**
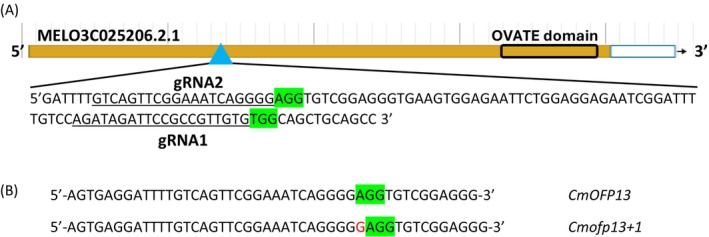
Visualization of *CmOFP13* and the gRNAs in the genome browser. (A) Schematic representation of *CmOFP13* (in 3′‐5′ orientation) and location of the gRNAs (in 5′‐3′ orientation). The OVATE domain is marked with a rectangle. The PAM sequence is highlighted in green in both gRNAs. (B) insertion (in red) found in plant 65 transformed with gRNA2.

*CmOFP13* has a 996 bp‐long CDS (331 amino acids), structured in a single exon, with the functional domain located in the C‐terminal region of the protein, between amino acids 263 and 322. Guide RNAs were designed to avoid translation of the functional domain (Figure 1A). Starting with 413 explants (203 for gRNA1 and 210 for gRNA2), 308 calli survived to selection after 2 months (137 for gRNA1 and 171 for gRNA2). Transformation efficiency was 67% for gRNA1 and 81% for gRNA2. We isolated and successfully obtained genotypic data from 71 T0 plants, identifying four false positives lacking the *Cas9* gene (File S4). The ploidy test revealed that all the individualized plants were tetraploid. Among the 67 *Cas9‐positive* plants, 16 plants (23.9%) exhibited editing in more than 50% of their sequences, and 38 plants (56.7%) presented edits in over 25% of their sequences. Despite polyploidy, we selected plant number 65 (79% of the cells carrying a +1 insertion and 19% a −6 deletion) and multiplied it in vitro (File S4). Four T0 clones from plant 65 were moved to the greenhouse and self‐pollinated to obtain the T1 generation.

### 3.2 From Tetraploid to Diploid Background

The chimeric T0 tetraploid plants were self‐pollinated. More than 600 seeds were obtained, with only two of them being T1 viable seeds. T1 plants inherited the +1 insertion in homozygosis and the *Cas9* gene (Figure 1B, Files S4 and S5). These plants were also tetraploid (*ofp13+1_*4n), with a slightly dwarf phenotype with flatter fruits (File S6A). When the tetraploid T1 plants were crossed with diploid VED wild‐type plants, only crosses where the diploid was the female were successful, resulting in four fruits. Fifty‐two seeds germinated, but only two seeds inherited the *CmOFP13* mutation from the tetraploid parent; the rest were undesired VED self‐pollinations. The two selected offspring were triploid (*ofp13+1_*3n), inheriting two copies of the mutated *CmOFP13*, and the *Cas9* gene. The phenotype of *ofp13+1_*3n plants was similar to the diploid wild type except for the fruit shape, with the triploids being significantly more flattened (File S6B). A final cross between *ofp13+1*_3n plants and diploid wild‐type plants gave fruits. A low percentage of seed viability was expected due to the aberrant chromosomal segregation from triploid cells. Most viable seeds were obtained when the diploid plant was used as the female. One hundred and ninety‐two offspring were genotyped. Only three diploid plants inherited the edition in heterozygosis, two of them being *Cas9‐free*. Finally, the diploid heterozygous edited *Cas9‐negative* plants (*ofp13+1*_het) were self‐pollinated, resulting in a diploid edited homozygous line (*ofp13+1*_homo).

### 3.3 Putative Effect of the Mutation in the CmOFP13 Protein and Phylogenetic Analysis

**FIGURE 2:**
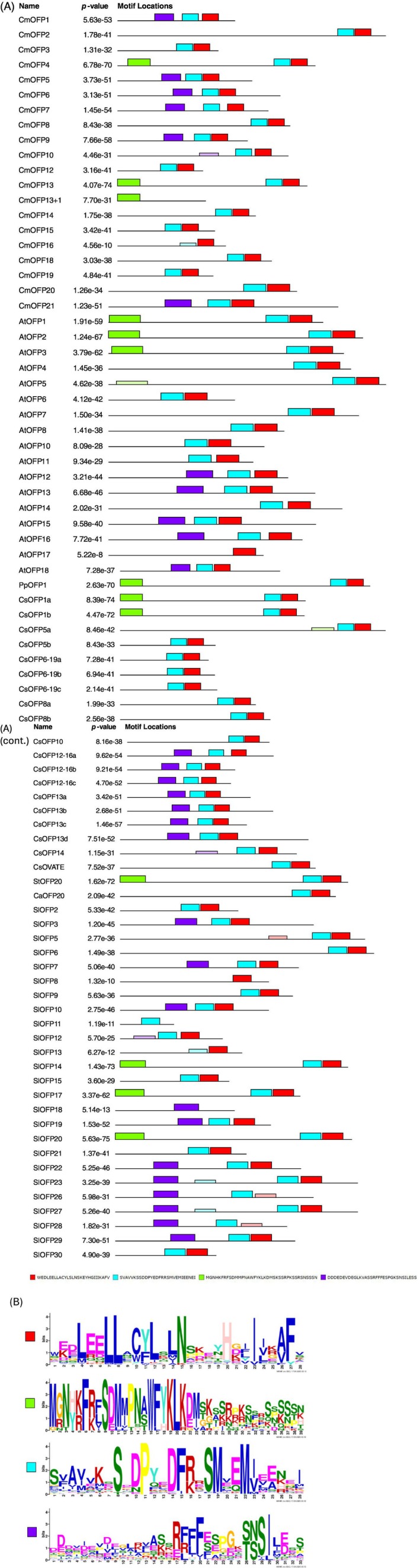
MEME analysis of OFP proteins. (A) Motif structure of OFP proteins from melon (Cm), cucumber (Cs), Arabidopsis (At), tomato (Sl), pepper (Ca), peach (Pp) and potato (St). (B) Logo of identified motifs. Colored squares correspond to the same colors in (A).

**FIGURE 3:**
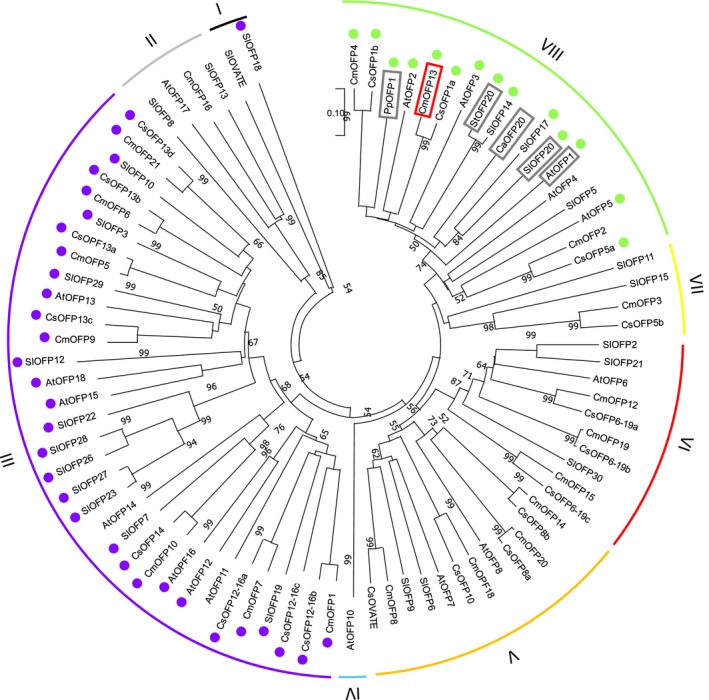
Neighbor‐Joining phylogenetic tree of melon (Cm), Arabidopsis (At), peach (Pp), pepper (Ca), potato (St), cucumber (Cs) and tomato (Sl) OFP proteins. CmOFP13 is highlighted within a red rectangle, whereas functionally validated orthologs are highlighted within grey rectangles. Proteins containing the predicted DNA‐binding motif are indicated with a green circle (in group VIII), whereas proteins containing the unidentified motif are indicated with a purple circle (in group III).

The insertion of one nucleotide introduced a frameshift mutation in the CmOFP13 protein (File S3). The resultant protein (CmOFP13+1) has a STOP codon after the mutation, preventing the formation of the OVATE domain. We analyzed the motif structure of CmOFP13 and the mutant by comparing melon OFPs with *Arabidopsis*, cucumber and tomato homologs, as well as those from potato, pepper and peach known to be related to fruit morphology (Figure 2). The MEME software identified four motifs: two belong to the OVATE domain (in blue and red), one is a DNA‐binding motif (PF13724, in green), and the last one is an unidentified motif (in purple). Most of the OFPs contain both OVATE motifs; a few proteins lack blue or red motifs. CmOFP13 also contains the N‐terminal DNA‐binding motif (in green), a structure shared with CmOFP4 (*MELO3C009113*), AtOFP1, AtOFP2 (*AT2G30400*), AtOFP3 (*AT5G58360*), AtOFP5 (*AT4G18830*), CsOFP1a (*CsaV3_4G027080*), CsOFP1b (*CsaV3_3G033200*), CsOFP5a (*CsaV3_6G051110*), SlOFP14 (*Solyc06g082460*), SlOFP17 (*Solyc09g018200*), SlOFP20, PpOFP1 and StOFP20 (Figures 2 and 3) (File S7). The mutant CmOFP13+1 lacks the OVATE domain motifs, while retaining the DNA‐binding motif (Figure 2).

MEGA 11 generated a tree with eight main clusters and several sub‐groups (Figure 3). CmOFP13 grouped together with CmOFP2 (*MELO3C007193*), CmOFP4, AtOFP1, AtOFP2, AtOFP3, AtOFP4 (*AT1G06920*), AtOFP5, CsOFP1a, CsOFP1b, CsOFP5a, SlOFP5 (*Solyc02g072030*), SlOFP14, SlOFP17, SlOFP20, PpOFP1, StOFP20, and CaOFP20 (CA10g10680). All of them shared the 3‐motif structure except CaOFP20, AtOFP4, SlOFP5 and CmOFP2, which did not contain the DNA‐binding domain. Interestingly, cluster VIII included three melon OFP proteins (CmOFP13, CmOFP4 and CmOFP2). On the other hand, cluster III included all proteins containing the unidentified purple motif, except for SlOFP18, which lacks the OVATE domain.

### 3.4 Phenotyping of the Gene‐Edited Plants

Since the studied gene had a putative effect on fruit shape, we phenotyped the fruits. Those from the diploid edited plants were harvested in three different locations and two seasons, and phenotyped for morphological traits (FL, FWI, FSI, and FA). Flowers were phenotyped at IBMCP in the summer of 2024. The effect on FSI of genotype (edited vs. wild type), location (TM vs. IBMCP), season (autumn 2023 vs. summer 2024), and the interactions between these factors were tested, finding significant differences only due to genotype and season, but no significant interactions (Table 2) (File S8A–E). The results were the same for FL, whereas differences in FWI and FA were only dependent on the season (Table 2). Differences observed between TM and CRAG fruits were attributed to both season and location, as the conditions did not permit any other comparison.

**TABLE 2:** Summary of ANOVA, Kruskal–Wallis and Welch‐ANOVA results for fruit and flower morphological traits.

Trait	Factor	*p*
FSI	Genotype	**4.28 × 10** ^**−12**^ *** and **5.06 × 10** ^**−15**^ ***
	Location (TM vs. IBMCP)	0.879
	Season (autumn 2023 vs. summer 2024)	**2.00 × 10** ^**−5**^ ***
	Genotype × Location	0.632
	Genotype × Season	0.307
FL	Genotype	0.005** and **3.13 × 10** ^**−4**^ ***
	Location (TM vs. IBMCP)	0.435
	Season (autumn 2023 vs. summer 2024)	**2.19 × 10** ^**−10**^ ***
	Genotype × Location	0.384
	Genotype × Season	0.852
FWI	Genotype	0.214 and 0.335
	Location (TM vs. IBMCP)	0.298
	Season (autumn 2023 vs. summer 2024)	**3.31 × 10** ^**−11**^ ***
	Genotype × Location	0.140
	Genotype × Season	0.607
FA	Genotype	0.665
	Season‐Location (CRAG 2023 vs. TM 2024)	**5.10 × 10** ^**−10**^ ***
	Genotype × Season‐Location	0.523

*Note:* Location refers to comparisons between TM and IBMCP, whereas season refers to comparisons between autumn 2023 and summer 2024. Parameters measured: Fruit length (FL), fruit width (FWI), fruit shape index (FSI) and fruit area (FA). Asterisks represent significant differences (*p < 0.05, **p < 0.01, ***p < 0.001). Numbers in bold represent statistically significant differences.

**FIGURE 4:**
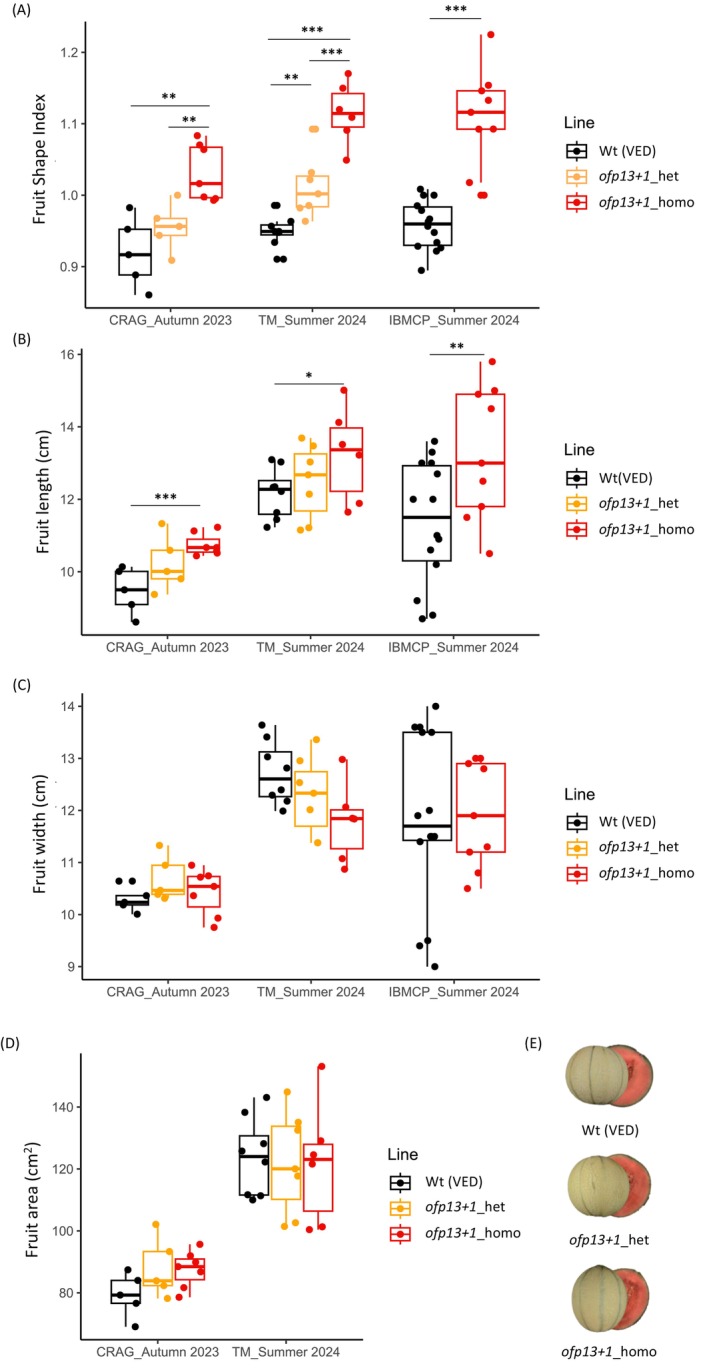
Fruit shape phenotype from wild‐type and *ofp13+1* diploid plants. (A) Boxplots of fruit shape index comparisons. (B) Boxplots of fruit length comparisons. (C) Boxplots of fruit height comparisons. (D) Boxplot of fruit area comparisons. (E) Fruit images of three genotypes. Asterisks represent significant differences (*p < 0.05, **p < 0.01, ***p < 0.001).

Considering seasons and locations, edited homozygous plants developed more elongated fruits compared to the wild type (Figure 4) (File S9A). FSI in knockout plants increased 12.1%–17.4% when compared to the wild type, whereas heterozygous lines had an intermediate phenotype, with a 3.8%–6.5% increase in shape, closer to wild type than to the mid‐parent mean, indicating partial dominance of the wild type allele. Regarding FL, knockout plants generated 8.8%–19.9% longer fruits, whereas heterozygous fruits were 2.6%–7.9% longer than the wild type. In contrast, although the interaction between season and genotype was not detected, the autumn 2023 fruits were flatter and smaller, with less prominent differences observed between genotypes.

**FIGURE 5:**
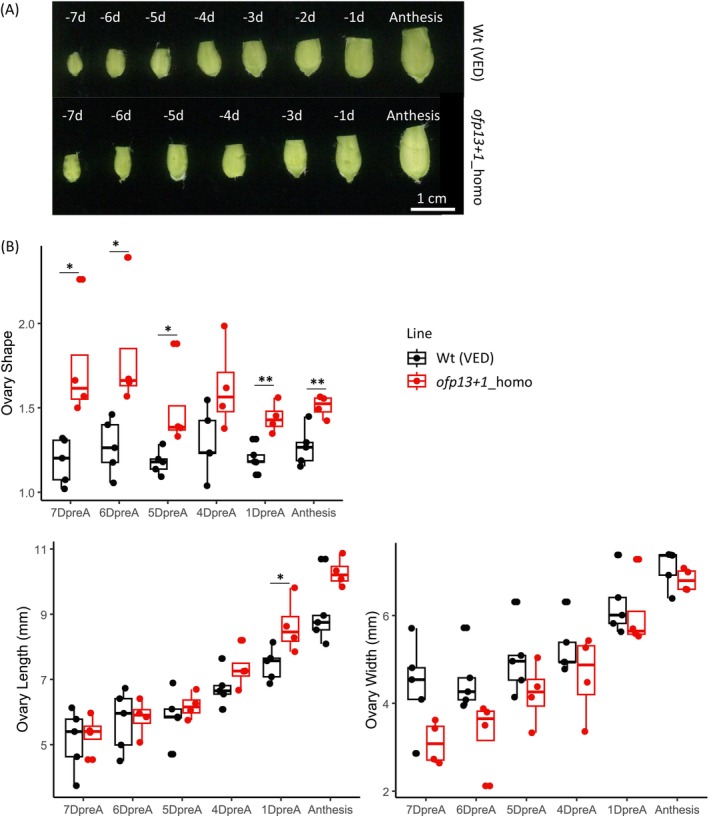
Ovary phenotype from wild‐type and *ofp13+1*_homo plants at the macroscopic level. (A) Ovary images. (B) Boxplots of ovary shape (OS), ovary length (OL) and ovary width (OWI). Asterisks represent significant differences between the wild type and the mutant (*p < 0.05, **p < 0.01, ***p < 0.001).

**FIGURE 6:**
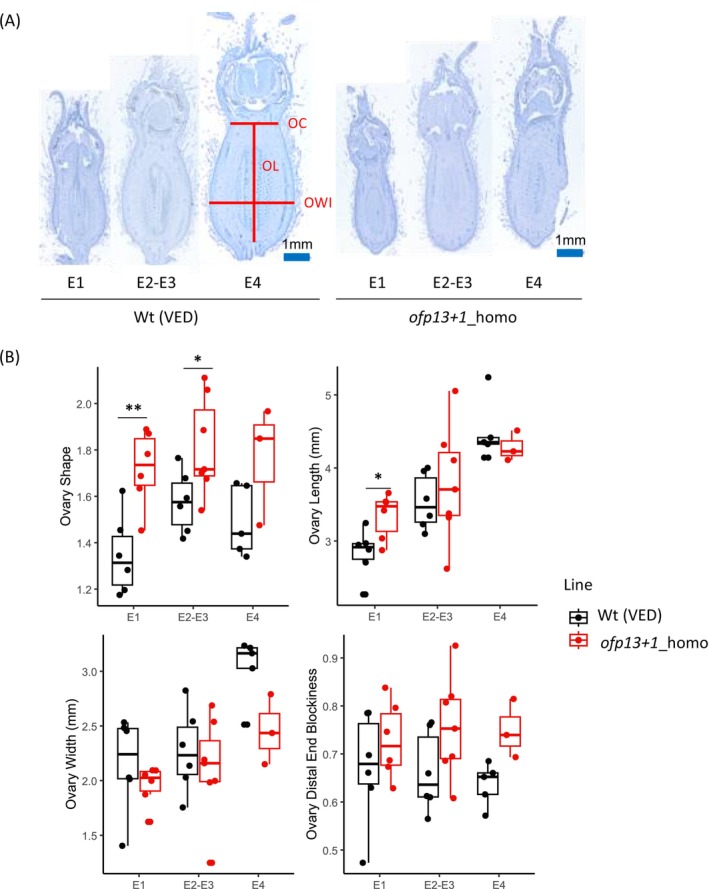
Ovary phenotype from wild‐type and *ofp13+1*_homo plants at the microscopic level. (A) Ovary images, red lines corresponding to ovary constriction (OC), ovary length (OL) and ovary width (OWI). (B) Boxplots of ovary shape, ovary length, ovary width and ovary distal end blockiness (ODEB). Asterisks represent significant differences between the wild type and the mutant (*p < 0.05, **p < 0.01, ***p < 0.001).

After analysing the fruit, we examined ovary development to determine the developmental stage at which shape differences became significant. Both macroscopic and microscopic data were used for flower morphology analyses. Macroscopic data compared ovaries from 7 days pre‐anthesis to anthesis (Figure 5A) (File S9B), whereas microscopic data compared developmental stages E1, E2–E3, and E4 (Figure 6A) (File S9C). ANOVA gave significant differences between groups in all ovary parameters measured (OS, OL, OWI, and OC), except for ODEB (File S8F–I). As with fruits, the edited line had a more elongated phenotype (Figures 5 and 6). Using pair comparisons by genotype, significant differences were observed in OS at the macroscopic level, from 7 days pre‐anthesis up to anthesis (Figure 5B). There was an increase in OL and OWI during ovary development (Figure 5B), with significant differences in OL only 1 day pre‐anthesis. At the microscopic level, significant differences were observed in OS from the very early stage E1, although they were less significant in later stages, probably due to the low number of replicates (Figure 6B). We also found differences in OL at this early stage, disappearing in the later stages. OWI differences were not detected, although slightly thinner ovaries were observed in edited lines at stage E4. In addition, no differences were detected at the ODEB (a constriction to width ratio), but slightly higher values were observed at stage E4 for edited lines.

**FIGURE 7:**
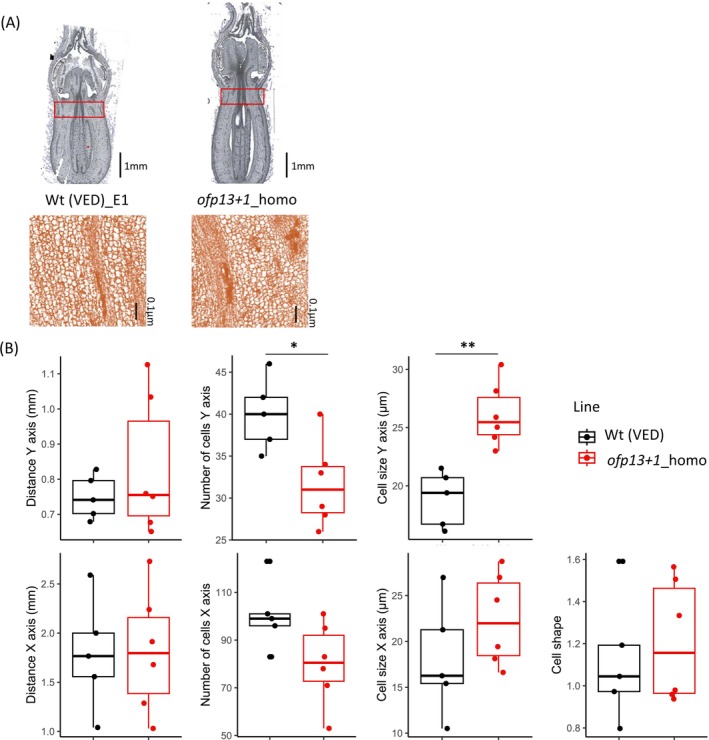
Ovary cell phenotypes in wild‐type and *CmOFP13* mutant (*ofp13+1*_homo) plants at the E1 developmental stage. (A) Ovary images, where the distal end area is delimited by a red rectangle. (B) Boxplots of distal end area and cell measurements. Asterisks represent significant differences between the wild type and the mutant (*p < 0.05, **p < 0.01).

Finally, in order to decipher the physiological origin of ovary shape differences, we looked at differences in cell size and shape in the distal end area at the E1 stage (Figure 7; Files S9D and S10). The distal end area of the ovary was comparable between wild‐type and mutant plants. However, edited plants exhibited a significantly lower number of cells along the Y‐axis compared to the wild type. Despite the increased cell size in the Y‐axis direction in edited ovaries, no significant differences in overall cell shape were observed between genotypes. These results suggest that CmOFP13 contributes to limiting cell expansion along the Y‐axis during ovary development.

## 4 Discussion

In this work, we generated melon edited plants for *CmOFP13* and compared them to the wild type (VED) to validate the function of this gene in melon. Transformation efficiency was similar to that previously found (Castelblanque et al. 2008; Giordano et al. 2022; Shirazi Parsa et al. 2023), despite the initial polyploidy of individual plants. Polyploidy is a common outcome of melon transformation and regeneration protocols. A variable percentage of tetraploids is usually obtained when regenerating plants from different starting tissues, such as leaves (Nizan et al. 2023), cotyledons (Guis et al. 2000; Shirazi Parsa et al. 2023), protoplasts (Debeaujon and Branchard 1992), adventitious shoots, shoot primordia and somatic embryos (Ezura et al. 1992). García‐Almodóvar et al. (2017) reported a positive correlation between tetraploidy and the germination time of the seeds used for explant preparation. Despite our methodology, aimed to maximize the percentage of diploids, only tetraploids were regenerated. This outcome could be due to the prolonged time the explants were cultivated in vitro (Ezura et al. 1992), as it took 6 months from callus to the first individual plants after transformation. Mutation efficiency in our tetraploid plants was approximately 56.7%, which is comparable to the rate reported by Giordano et al. (2022).

### 4.1 Polyploidy Affects Fruit Shape in Melon, Which Can Be Overcome by Crossing

The phenotype of tetraploid fruits was flat. In previous studies, tetraploid melons have also been described as small and flat (Ezura et al. 1993; Fassuliotis and Nelson 2019; Nonaka et al. 2023). However, the effect of ploidy on plant size and fruit shape depends on the cultivar, as some studies have generated tetraploid plants that were taller and had larger fruits (Zhang et al. 2010). In the triploid edited fruits, the flatter phenotype was observed again. *Cantalupensis* triploid fruits have been described as similar in size and shape to the diploid fruits in the ‘Harukei‐3’ cultivar, which belongs to the *cantalupensis* group as VED (Ezura et al. 1993). However, a cultivar‐biased effect could explain this difference, as observed in our results.

Polyploidy has been a challenge when applying CRISPR/*Cas9* in melon, necessitating the comparison of wild‐type and edited plants at the tetraploid stage (Nonaka et al. 2023). Polyploidy can mask the effect of a mutation, so these comparisons may not always be reliable. In this study, we successfully addressed the issue of polyploidy through two generations of crosses. Although the seed viability from the crossings was very low, due to the low probability of homogeneous chromosomic segregation during meiosis in polyploid plants (Chen and Palmer 1985; Yang et al. 2019), it could be a partial solution for CRISPR/*Cas9* experiments where no diploid plant can be obtained.

### 4.2 *CmOPF13* Is a Regulator of Ovary and Fruit Shape in Melon

Biotechnological approaches, such as gene overexpression or CRISPR‐based knockout of *CmOFP13*, had not been used in melon in previous studies. Heterologous expression of the PI 124112 allele of *CmOFP13* in *Arabidopsis* confirmed that the main effect of this melon gene is to shorten the organ length (Martínez‐Martínez et al. 2022), and these authors associated the rounder shape with an increase in the expression of *CmOFP13* during early ovary development, relating the low or lack of expression to the elongated phenotype. Therefore, we would expect that *CmOFP13* knockouts would induce an elongated shape. Our experimental approaches confirmed this hypothesis, with edited plants yielding more elongated ovaries and fruits. Differences in the magnitude of elongation were observed. For example, during the autumn of 2023 we observed flatter fruits than in the summer of 2024, suggesting an environmental effect on fruit shape. Temperature has been proven to affect fruit growth. For instance, cucumber plants grow bigger fruits when exposed to high diurnal temperature variation compared to constant temperatures (Kläring and Schmidt 2017). In our experiment, autumn melons were cultivated at CRAG under more controlled conditions than at TM or IBMCP, resulting in lower temperature variations between day and night, which could have slowed longitudinal growth and contributed to the flatter melons. Autumn melons also received a greater amount of artificial white light. The amount and type of light might also impact fruit growth, as artificial white light presents a different spectrum than natural sunlight (Hogewoning et al. 2010). These factors should be further studied to clarify the roles of temperature and light on melon fruit growth. Nevertheless, the effect of the mutation was observed to be significant in all cases. The lack of interaction observed between genotype, location, and season suggests a major genetic contribution of *CmOFP13* to fruit shape, in line with the findings of Martínez‐Martínez et al. (2022).

Our results also corroborate that melon fruit shape is determined at the flower stage in female and hermaphrodite flowers, since we detected significant differences at early flower developmental stages, as did Martínez‐Martínez et al. (2022), although the stage where differences are clear depends on the genotype. The same authors found that the more elongated shape of the SC8‐3 line was evident at the E1 stage, while the round shape of CALC8‐1 was clear at the E4 stage. In this *ofp13+1*, an elongated shape is evident at the E1 stage, as in SC8‐3. There was no expression of *CmOFP13* at the E2 stage in the SC8‐3 line, so it seems the knockout had the same effect as the lack of expression.

Shape differences were determined dependent on length and width in the ovary, although differences were only significant at some developmental stages. The small number of replicates might have masked the contribution of these parameters to the change in shape. Length seems to have a greater impact on the final shape. In fact, cell measurements at the E1 stage highlighted the predominant role of *CmOFP13* in restricting cell growth along the Y axis in the distal end area of the ovary, while also promoting cell division. In mature fruits, phenotypic differences were limited to fruit length. Overall, *Cmofp13+1* mutants exhibit a similar but more pronounced phenotype compared to the tomato mutant *Slofp20* (*sov1*), which likely results from its interaction with *Slovate*, as demonstrated in the *ovate/sov1* double mutant (Wu et al. 2018). The elongated phenotype observed in melon mutants was not as intense as in *flexuosus* groups (Pitrat 2017). This could be desired for breeding, as consumers are not accustomed to very elongated melons. The observed phenotype supports the idea that other *OFPs* or other gene families may play a role in melon fruit shape.

Regarding the effect of the mutation at the molecular level, CmOFP13+1 putatively lacks the OVATE domain. This domain is known to interact with TRMs in tomato through the M8 domain in vitro, favoring round phenotypes (Wu et al. 2018). An aspartic residue in SlOFP20 has been reported to be essential for the OFP‐TRM interaction, and the loss of this interaction by an OFP antisense was reported to produce elongated fruits. The predicted version of the edited melon protein could impair CmOFP13 normal activity, making it impossible to interact with TRMs (Wu et al. 2018). In the potato ortholog *StOFP20*, tuber elongation was associated with a whole‐gene deletion, whereas in other orthologous genes, the effect on organ morphology has been linked to differential expression (Borovsky et al. 2022; Martínez‐Martínez et al. 2022; Wu et al. 2018; Zhou et al. 2021). In melon, a time‐based differential expression pattern has been detected in *CmOFP13* when comparing SC8‐3, PS and CALC8‐1 lines (Martínez‐Martínez et al. 2022). Together with the fact that shape difference begins at the early development of the ovary, *CmOFP13* expression at the onset of flower differentiation might determine a rounder fruit shape. In contrast, the function of the DNA‐binding domain of this protein remains unclear and needs to be further investigated through protein–protein interaction assays, such as the yeast two‐hybrid system, in melon. In *Arabidopsis*, the AtOFP1 DNA‐binding domain is reportedly involved in non‐homologous end‐joining (NHEJ) during DNA repair. However, this function does not seem to be related to the control of fruit shape (Wang et al. 2010).

### 4.3 OVATE Domain of CmOFP13 Is Not Essential for Plant Development

Martínez‐Martínez et al. (2022) studied the genetic variation of *CmOFP13* using data from Zhao, Lian, et al. (2019), concluding that there was no genetic diversity in the coding sequence, nor were the structural variations surrounding the gene associated with fruit shape. The high conservation of the *CmOFP13* coding sequence suggested an essential role of the protein, the differences in fruit shape being putatively caused by changes in expression or interactions with other proteins. An essential role has been reported for *AtOFP1*, affecting organ development and pollen function by modifying the subcellular localization of TALE proteins and inhibiting *AtGA20ox1* expression (Hackbusch et al. 2005). Borovsky et al. (2022) and Wu et al. (2018) silenced the respective *CmOFP13* orthologous genes in potato and tomato with no evident effect on plant viability, although the effect of a complete knockout was not tested. In this report, the *CmOPF13* knockout plants exhibited no pleiotropic or deleterious phenotype, as they were similar to wild‐type plants and could produce viable seeds. This indicates that the OVATE domain of this gene is non‐essential in melon.

### 4.4 Phylogenetical Analyses Reveal New Candidate Genes to Study Melon Shape

Results from the MEME motif search and the phylogenetic tree were similar to a previous study by Zhang et al. (2020) using banana OFPs. These authors identified motifs apart from the OVATE, one of them coinciding with the DNA‐binding domain we observed. Our analysis confirmed that *CmOFP13* is orthologous to *SlOFP20*, *AtOFP1*, *PpOFP1* and *StOFP20*, validated genes modulating organ shape (Ai et al. 2023; S. Wang et al. 2007; Zhang et al. 2023; Zhou et al. 2021). Two other melon OFP proteins, CmOFP4 (*MELO3C009113*) and CmOFP2 (*MELO3C007193*), clustered with CmOFP13 making them interesting for further study, as they could contribute to shaping other organs. According to expression data, *CmOPF4* is mainly expressed in the fruit flesh and epicarp of round “Harukei‐3” melons, as well as in roots (Yano et al. 2020) (File S11A). In elongated PS and round VED melons, RNAseq data revealed relevant expression in fruit flesh. Significant differences were found between PS and VED correlating with shape, with higher levels of expression of *CmOPF4* in the round melon (Santo Domingo et al. 2024) (File S11B). In the Melonomics database (https://melonomics.net), no SNPs differentiate PS from VED in the coding sequence nor in the promoter region, suggesting that expression differences might be due to trans regulation. Although *CmOFP4* does not colocalize with any consensus QTL for fruit shape (Pan et al. 2020), homology and expression data make it a potential candidate to continue studying melon fruit shape regulation. On the other hand, *CmOFP2* has low expression levels in “Harukei‐3,” PS, and VED fruits, but exhibits some expression in seeds and ovaries (File S11) (Yano et al. 2020), suggesting that it may be involved in shaping other plant organs.

In addition, our phylogenetic analysis could be used to detect candidate genes in other species. For instance, a recent study revealed the negative role of *MELO3C009515* (*CmOFP19* in this paper) in fruit size (Chi et al. 2025). Genes in cluster VI, and more specifically cucumber ortholog *CsOFP6‐19b*, might also be involved in fruit size and would merit further study. Besides, *CmOFP8* (*MELO3C017554*), orthologous to *CsOVATE* (*CsaV3_4G005140*), a controller of fruit neck growth in cucumber, might also play a role in melon shape (Z. Wang et al. 2022).

## 5 Conclusions

The present study aimed to generate *CmOFP13* CRISPR/*Cas9* knockouts in melon to study its contribution to fruit shape. The induced edition demonstrated the effect of *CmOFP13* in governing melon fruit shape from the very early flower developmental stages. This work opens up the possibility of producing new melon fruit shapes for commercial purposes, for example by changing the gene expression or the activity of the protein. Our mutant line adds to the still short list of melon genes successfully edited by CRISPR/*Cas9* to date.

## Author Contributions

C.M., J.G.‐M. and M.P. conceived and designed the experiments. C.M. and D.J.‐S. obtained CRISPR/*Cas9* gene‐edited plants. C.M., M.‐J.G., M.V., and G.G.‐B. performed the phenotyping. C.M. wrote the original draft of the manuscript. A.J.M., J.G.‐M. and M.P. coordinated the project, revised and edited the manuscript. All authors read and approved the final manuscript.

## Funding

This work was supported by grants PID2021‐125998OB‐C21 to J.G.‐M and M.P. and grant PID2021‐125998OB‐C22 to A. J. M. both funded by MICIU/AEI/10.13039/501100011033 and by “ERDF A way of making Europe”; grant TED2021‐131955B‐I00 funded by MICIU/AEI/10.13039/501100011033 and by the “European Union NextGenerationEU/PRTR”; CEX2019‐000902‐S funded by MICIU/AEI/10.13039/501100011033; the CERCA Programme/Generalitat de Catalunya and the 2021 SGR 00756 grant from the Generalitat de Catalunya to J.G.‐M and M.P. C.M. was supported by grant 2019 FI_B 00124 from the Secretaria d'Universitats i Recerca del Departament d'Empresa i Coneixement de la Generalitat de Catalunya and by grant 2019FI_B00124 from the “ESF investing in your future”; M.V. was supported by grant PRE2022‐102735 funded by MICIU/AEI/10.13039/501100011033 and by ESF+.

## Disclosure

The authors declare that they have not used Generative AI tools to prepare this manuscript.

## Supporting information


**File S1:** ppl70641‐sup‐0001‐FileS1.pdf.

**File S2:** ppl70641‐sup‐0002‐FileS2.pdf.

**File S3:** ppl70641‐sup‐0003‐FileS3.docx.

**File S4:** ppl70641‐sup‐0004‐FileS4.xlsx.

**File S5:** ppl70641‐sup‐0005‐FileS5.pdf.

**File S6:** ppl70641‐sup‐0006‐FileS6.pdf.

**File S7:** ppl70641‐sup‐0007‐FileS7.docx.

**File S8:** ppl70641‐sup‐0008‐FileS8.docx.

**File S9:** ppl70641‐sup‐0009‐FileS9.xlsx.

**File S10:** ppl70641‐sup‐0011‐FileS10.pdf.

**File S11:** ppl70641‐sup‐0011‐FileS11.pdf.

## Data Availability

The data that supports the findings of this study is available in the Supporting Information of this article or is cited in the main body of the text.
