# Supplementary material for: Targeted Editing and Phenotypic Profiling of CmOFP13 Mutants Reveal Its Role in Melon Fruit Morphogenesis
Source: Physiol Plant. 2025 Nov 29;177(6):e70641. doi: 10.1111/ppl.70641 (PMC12664293; doi:10.1111/ppl.70641)

Supporting information

Targeted editing and phenotypic profiling of *CmOFP13* mutants reveal its role in melon fruit morphogenesis

Carlos MAYOBRE[1,†], María José GONZALO[2], Montserrat VERGÉS[1], Guillem GUARDIA-BERSABÉ[1], Dídac JIMÉNEZ-SÁNCHEZ[1], Antonio José MONFORTE[2], Jordi GARCIA-MAS[1,3], Marta PUJOL[1,3,*]

[1]Centre for Research in Agricultural Genomics (CRAG) CSIC-IRTA-UAB-UB, Edifici CRAG, Campus UAB, Bellaterra, 08193 Barcelona, Spain

[2]Instituto de Biología Molecular y Celular de Plantas (IBMCP), Consejo Superior de Investigaciones Científicas (CSIC), Universitat Politècnica de València, Ingeniero Fausto Elio s/n, 46022 Valencia, Spain

[3]Institut de Recerca i Tecnologia Agroalimentàries (IRTA), Edifici CRAG, Campus UAB, Bellaterra, 08193 Barcelona, Spain

[†] Current address: INRAE, UR1052, GAFL, 84143 Montfavet, France

[*]Correspondence

Marta Pujol

E-mail: marta.pujol@irta.cat

File S1. pEn-Chimera map.

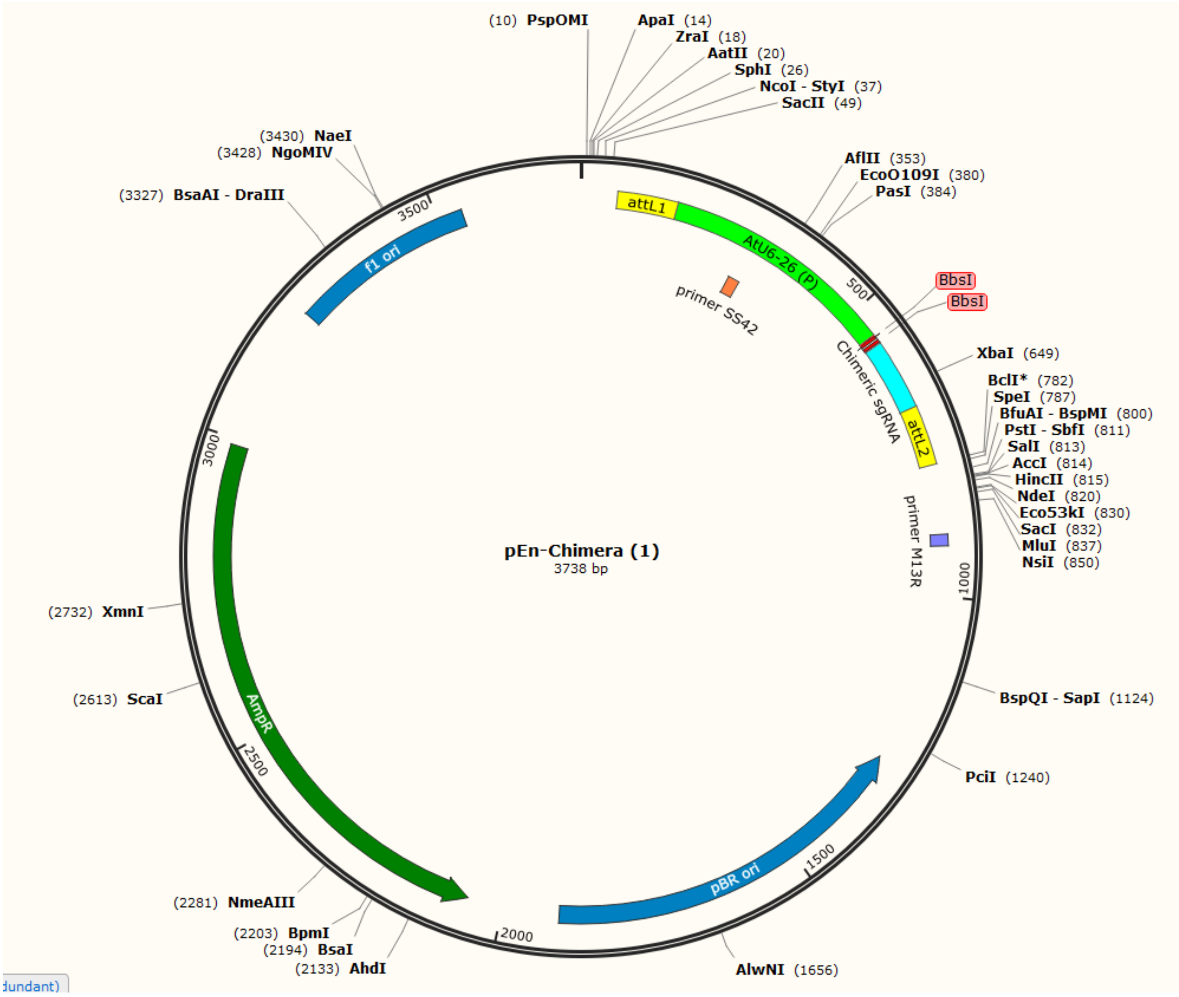

File S2. pDe-CAS9 map.

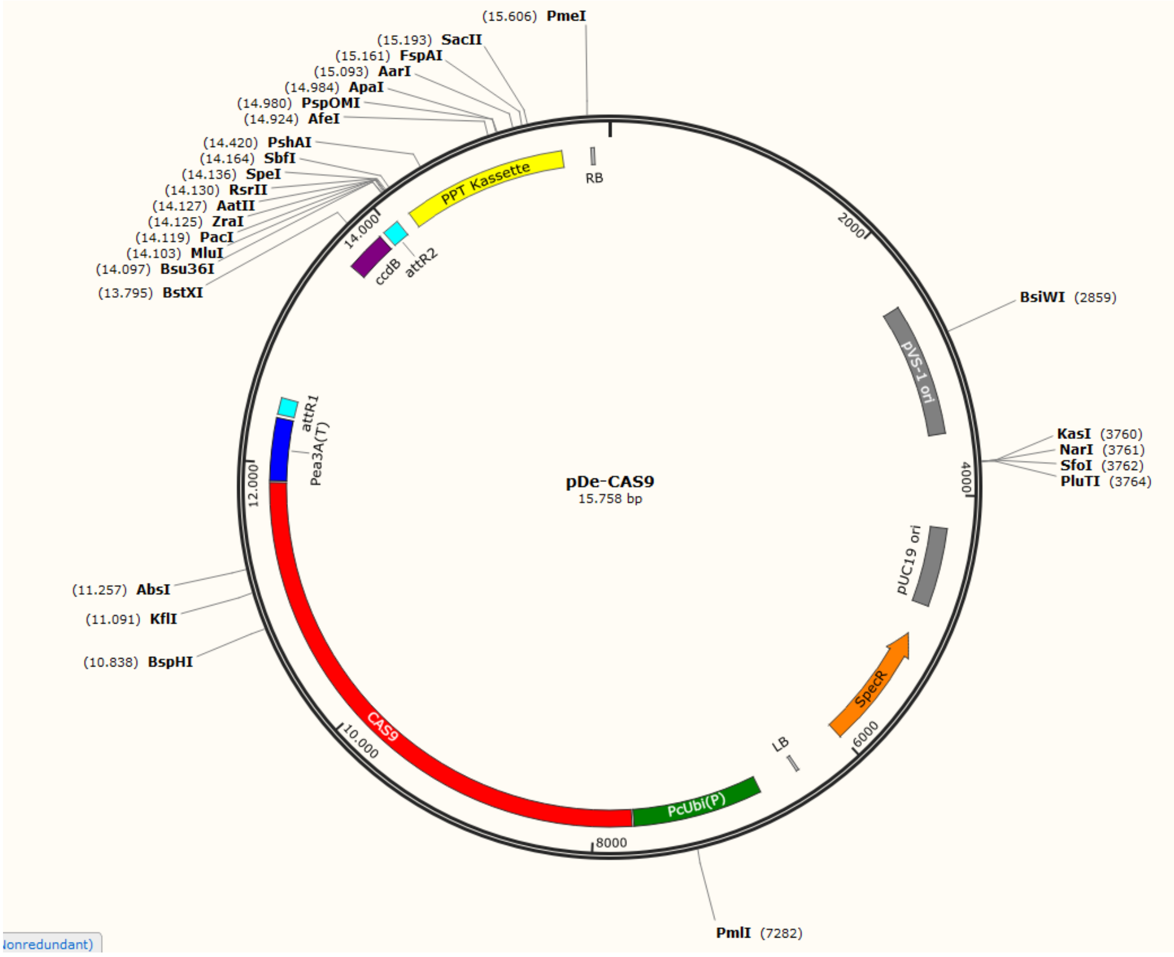

File S3. OFP DNA and protein sequences

>*CmOFP13* (ATG and STOP codon in red, Kozak sequence is highlighted in yellow)
TCCGATGAGAAATCACAAGTTCCGTTTCTCCGACATGATACCCAACGCCTGGTTTTACAAACTCAAAGAAA
TTGGCGGCGCCTCCAGACCAAAATCTTTCCGTTCCAACAAAAACCCTCACCACCCACCTCCACCTCCCCCG
CCCTCCAAACACAAACAACCACCCCCTCCTCCTCCCCACTCTCGTTCCAGAAAATCTTACTATTTCACTAGA
CAACTCGAATCCAACGATGCCTACTTCGTCAATTCCCCTCCACCGTCGCCTCCGCTTCTACCGGTACCAATC
CCCCCGAGAAAGTCAACAAAACAACTCAAACCAGGAAGAAAACAAACGAGTTCCCGGTCCTCCGCCAA
GCTCCTCAGCTCCTCCTCCGTCGGCTGCAGCTGCCACACAACGGCGGAATCTATCTGGACAAAATCCGATT
CTCCTCCAGAATTCTCCACCTCACCCTCCGACACCTCCCCTGATTTCCGAACTGACAAAATCCTCACTGCCG
AAGCATCCAAACACTTCGAGCACGACATCGTAATCGACGTATCGTCGAATTACTCCAACAATGCCGTCATC
GGCGCCTTTGACGAACTGGAACTCCCGCCGATCATCACGAAACAGAGGAAGAAAACAGAGACAAAACA
GAGAACGACGACGACAACGACGGCAGGAACAAAGAAAGTTGCGGGGAATTCCCCGGGCGTACGGCTG
CGGATTCACTCCCCGAAAATTGGGTACCGGAAAATGGGAGGGAGGAAAAGCGTTTCGTCACGGCGGAG
CTTGTCGGAGAGTTTAGCGATAATGAAATCATCGTACGATCCACAAAAGGACTTCAGAGAATCAATGGTG
GAGATGATTGTTGAGAATAACATTAGGGGTTCGAAAGAATTGGAAGATCTTCTTGCATGTTATCTGTGTTT
GAACGCCGATGAATATCATGATCTTATTATCAAAGTTTTTAAGCAGATCTGGTTTGATCTTACGCAACCTTCT
CCTCCACCTCTTTGATTTTCTTTCGTCTTTTCTCTTTCTTTCTCCCCCATTTTGTAATTCTCTTTTGTACAGTTC
TTCTTCTCTAATTTCTAAACATAATCTTAATAAAATTTTTACTTTCATTCACTTTTTAGTTT

>*OFP13+1v1* (ATG, mutation and STOP codon in red, Kozak sequence is highlighted in yellow)
TCCGATGAGAAATCACAAGTTCCGTTTCTCCGACATGATACCCAACGCCTGGTTTTACAAACTCAAAGAAA
TTGGCGGCGCCTCCAGACCAAAATCTTTCCGTTCCAACAAAAACCCTCACCACCCACCTCCACCTCCCCCG
CCCTCCAAACACAAACAACCACCCCCTCCTCCTCCCCACTCTCGTTCCAGAAAATCTTACTATTTCACTAGA
CAACTCGAATCCAACGATGCCTACTTCGTCAATTCCCCTCCACCGTCGCCTCCGCTTCTACCGGTACCAATC
CCCCCGAGAAAGTCAACAAAACAACTCAAACCAGGAAGAAAACAAACGAGTTCCCGGTCCTCCGCCAA
GCTCCTCAGCTCCTCCTCCGTCGGCTGCAGCTGCCACACAACGGCGGAATCTATCTGGACAAAATCCGATT
CTCCTCCAGAATTCTCCACCTCACCCTCCGACACCTCCCCCTGATTTCCGAACTGACAAAATCCTCACTGCC
GAAGCATCCAAACACTTCGAGCACGACATCGTAATCGACGTATCGTCGAATTACTCCAACAATGCCGTCAT
CGGCGCCTTTGACGAACTGGAACTCCCGCCGATCATCACGAAACAGAGGAAGAAAACAGAGACAAAAC
AGAGAACGACGACGACAACGACGGCAGGAACAAAGAAAGTTGCGGGGAATTCCCCGGGCGTACGGCT
GCGGATTCACTCCCCGAAAATTGGGTACCGGAAAATGGGAGGGAGGAAAAGCGTTTCGTCACGGCGGA
GCTTGTCGGAGAGTTTAGCGATAATGAAATCATCGTACGATCCACAAAAGGACTTCAGAGAATCAATGGT
GGAGATGATTGTTGAGAATAACATTAGGGGTTCGAAAGAATTGGAAGATCTTCTTGCATGTTATCTGTGTT
TGAACGCCGATGAATATCATGATCTTATTATCAAAGTTTTTAAGCAGATCTGGTTTGATCTTACGCAACCTTC
TCCTCCACCTCTTTGATTTTCTTTCGTCTTTTCTCTTTCTTTCTCCCCCATTTTGTAATTCTCTTTTGTACAGTT
CTTCTTCTCTAATTTCTAAACATAATCTTAATAAAATTTTTACTTTCATTCACTTTTTAGTTT

OFP protein sequences in melon, Arabidopsis, tomato and rice, plus validated OFP proteins from peach, cucumber, potato and pepper

>MELO3C006531_CmOFP1
MSSLKKSNFHLWFSKLRCFPATVKPSSPPKTPNKKPSPITHLENSDYSSTSTAADDGFFSDESTSDSDAIVPDFS
AAVASHRFFFSSPGCSNSIFDSSPDTHHSAAVSAAVHGGVEVRKVSMDPFVDFRASMQEMVEARDRPVDV
RRDWEYLQDLLLCYLRINPVDTHKFILRAFSDLVVYLLECSPESFSDRRLRPHNINSNSW

> MELO3C007193_CmOFP2

MKWGRRKPHNPSSSSSTSSSSSSRPSFMSNILPASWLSKLKQKKSNHEARPRKVKGTEKGNSPCIQSPDYAN
VTPSPGQVNGNRNRLFTGDNGEFWKLSFGGDDIDVKKSSGILRSVWYNSEDEHDLPRTSCRSCRTKCTESE
GKEEIQNLDDMVSRMTRRRRRRREAPTQVKLLRRESETESRTPRRKYRENGNFGYLGKKSMEKKGFKPERET
DKGKERARRLVGKKMLGVEEESGVRKNERDKTNLTINSRKHRYVPSTMSKSSNLGTIEENCVFSSMKAEESD
GHDTVGIEIDSDWERMKELKIEELKLRYEKQRQPLYIRKDSNEKNPKGRRKIRVYSPRTANKIEICKIKALEDMK
KAKLKMKKKVRESMVDDETDLESFAVVKSSFDPQQDFRDSMVEMIMERRISKAEELEELLACYLTLNSDQYH
DLIIKVFRQVWFDLNQAALESELHKQFPCNEQLV

> MELO3C007422_CmOFP3
MIIFGLQAPFKAVRMKAAGSTQHHHQAQNAYRALCCGCSCNCRLSFSSSEETESFNSDKFPSVSSIAHAMV
QERLDQMIREKREVRNGKERKKQRSEDTKFVVMVAMEKCSDDPKEDFRVSMTEMILANRIEEPKDLRNLLN
YYISMNSDECHGVIFEVFHEVCSNLFLACKRHYW

> MELO3C009113_CmOFP4
MVGDRVEWSRERNNHFWRMGNYRFRVSDMMPNSWFYKLKDMTTIIRRRNKNKDQPSKNTHTTTDLAY
SHPRKSIHFTASQLAANSPPEPPRRSSKGKKPRRRPTSSSAAAPTSTLLLTSSSGCSCGRTALQSVATTSTTPPPI
LTHHSYLHAEKEEEDANAVISGKAHKVSPKKINGSDEEYLKSLPQIIDQLPPIITRSSSSSSNAADASTCPSISIAK
NDKSEPIRSSPSRRFLLNSPGPKLRIVNSPRVSSSKRFGHVGRRKSGKRSLNDSLAIVKSTEDPQRDFRESMME
MIVENKISGSSELEDLLACYLSLNTDEYHDIIVKVFKQIWFDMTDIIGVHY

> MELO3C009514_CmOFP5
MLTKKKKMMMMMRLPSLFKILAIDDKSTFPWPSCRQPRTLSFRTTSAAVATATDSSGSFFTLSSESSGSLSTV
SESSGGDPIERMIRDLRSTKRLHFEPTGKSSSIVEDETVSHPLKEGTTVMSMDSNDPYSDFRKSMEEMVEAH
GMKDWESLEELLNWYLRVNGKKNHGFILGAFVDLLVSLAMASSSSSSSSSSSSLCCYSSSSSSSSSLPCVSSSME
IEEISSLDEHHHHVYS

> MELO3C012340_CmOFP6
MANTKKKHHLIKNFPSIFKSKSPTSSHPWEWDWPSCKHPKTLSFRSQNDLVFKTVNSIFFDQPFETITTTTTT
TPDYSSSLHTNSSDSVSATNSTPAMDSEESLETVVRGARSERLFFEPDDTSSILEKSKPIESVETDELPRSGFKES
LIVSIESENPYEDFRKSMGEMVESHGVKDWDGLEELLGWYLKANWKNNHRFIIGAFVDLLIHILLASSSSSSST
STSSSSSSSLCSNSDSNYYTCTESSSSRSCSSSLRSLNPSSIRKELDHEEEDDHDHITETL

> MELO3C015818_CmOFP7
MPTKLGRNYLNLCFTKIKNPLSATQSSPPITHTPDRRRQTTRSFSSTAAAFITNYNSLYEITTTTNSDSNSPLFGL
ANDIGVADPDAYVAVDFITAFTSHRFFFSSPGSSNSIIESTTPTTTESTTTMSLSSEYSARYEGNDDELMIFNNS
HVIPTYSPDPYMDFRRSMQEMVEAREKMTTAATTTTMKKSSWEFLHELLLCYLALNPKATHKHILKAFADVA
TVIKPPLAMKETEEEGNNVDRESESMVDDGGPGGGCEISG

> MELO3C017554_CmOFP8
MMTPKRFKLLRIPSFHCCRSNDISVVPTDPPSPSPPSKPQHSSLRRHVSSAFRTAACGCRSSSTKSDDDQIPKS
TPTLPTHVPPTPLLHSYDDGSTFPKRRRRRRNKNKSKSTTLTRLRTSTSSTESGLFSSESFDEIDELEETETLISSSK
TISTSDDDNDSSSEFNPQLETIREKPNKIKLRRKKEKEKRRRKQKRTTIIKTSPSPEIESPARLSVFQRLIPCTVEGK
IRESFAVVKKSADPFEDFKRSMVEMIMEKEMFEEKDLEQLLHCLLSLNDREHHGIIVEAFAEIWQSLFCNN

> MELO3C019910_CmOFP9
MGKKMKLLPSLFKNRQSPDRPWQWPLCGTSKTPSFRAPHHHQIFTTLNSTFFHNFLSDPIHTPDSWFANSS
LFESARVSLSTEFEDDLELVIQGAKSERLIFEPGETNSILDQSRGGKSEGGKCESILRFEGSVVVLMAMESEDPY
LDFRRSMEEMVECHGIRNWEWLEELLNWYLRMNGMKNHGYILGAFVDLLVDLGGADGSTDSTSIFSDDLII
QPHDRERCVV

> MELO3C024232_CmOFP10
MLLSFFCIPLLFLYKNQYYHITLSTQNQRKLNKMPKKLQKSLQDYLSKIKKPTPQLQFPNPQTFSSSKSWILAGC
KHPKTLSFAIDRKQVDAVGNKEDAAATLADIDRFLFENFRSLYLKEDGDCGDRKVVGGGGGGRDCKNHRGV
VSPESPVDSYGGSHRFFFSPDLSGSDLPDDSHTESSENAGSSSSSLIGEDRGKDLKLPSDCIAILRKSPNPSEEFR
RSMQEMMDGHLKHHEKVDWEFMEELLFCYLNLNDKKSYKYILNAFVDLIVILRQKAEEEPAKPRTVRSVRM
VRRMI

> MELO3C024574_CmOFP12
MAAPRRNLQPTSLSVDLNICRPKLLSHLFHHLKPKPSLKSPNHHHRFSSASSDSESESETRTSITFRGFGRSGG
ESVAVEKDSDDPYLDFRHSMVQMILENEIYSKEDLRGLLRCFLQLNSPSHHGIIVRAFSEIWDSVFSATSPILRF

> MELO3C025206_CmOFP13
MRNHKFRFSDMIPNAWFYKLKEIGGASRPKSFRSNKNPHHPPPPPPPPSKHKQPPPPPPHSRSRKSYYFTRQL
ESNDAYFVNSPPPSPPLLPVPIPPRKSTKQLKPGRKQTSSRSSAKLLSSSSVGCSCHTTAESIWTKSDSPPEFSTS
PSDTSPDFRTDKILTAEASKHFEHDIVIDVSSNYSNNAVIGAFDELELPPIITKQRKKTETKQRTTTTTTAGTKKV
AGNSPGVRLRIHSPKIGYRKMGGRKSVSSRRSLSESLAIMKSSYDPQKDFRESMVEMIVENNIRGSKELEDLL
ACYLCLNADEYHDLIIKVFKQIWFDLTQPSPPPL

> MELO3C025206_CmOFP13+1
MRNHKFRFSDMIPNAWFYKLKEIGGASRPKSFRSNKNPHHPPPPPPPPSKHKQPPPPPPHSRSRKSYYFTRQL
ESNDAYFVNSPPPSPPLLPVPIPPRKSTKQLKPGRKQTSSRSSAKLLSSSSVGCSCHTTAESIWTKSDSPPEFSTS
PSDTSP

> MELO3C025343_CmOFP14
MDTRFKLRLSRVFQSSFASCRSRNLSDILHKAVFIPSSSDDASFRKISPSETSSDFLLPRRKISRRFPLSPLPSAISG
GRTCPPTSPISPSKPISEKMTTSTKKKNKHKKQRKQRKQSKREIPFSLFSSSNFGGTWWYSSEDEDDDDETDT
LFSSKSRSSDSSKHLTDDRNSFAVVKKSSDPYNDFRMSMLEMIVEKQIFSAKDLEQLLQCFLSLNSHHHHNVIL
EVFTEIWEALFSDWGS

> MELO3C025581_CmOFP15
MSNIRKNKFFTTIFSSTAGCGRCQKPKLSDIVQPNKKPPTTIVRRSSSSSSTDHNGTFSLDEDYSSASKSTGSQS
PVAILICDSIAVEKDSDDPYEDFRRSMVQMIVEKRIYSPNGLQELLNCFLHLNSPYHHEIILKAFTQISNEFESSH
RLWNISNDNEWKPRSAGEG

> MELO3C026874_CmOFP16
MKVEVGLVSFKSKLSKPCSKLLHLFKFPMKKPFSIKSLWTRHPRRNSRAISKPRRRTWSWLRWLRRVGKMER
VRDHLRSSESVRSDNECREKLLFPSPMIRGRKVAAGTSWEEKEEVEDACKSFENYLVEMIIEEGKVRDLMDVE
ELLYCWRNLKCPVFVDLVSRFYGELCKDLFSSHIQAFTPNFQPK

> MELO3C010932_CmOPF18
MARSFKLRVRLFISSFRFCRPKHLRALTFPKTSSPENFHYKKQIPTNPKFCSCYSNPPSPPPSTPVDLFADFPKEK
STSICNSCKLKSFARKNGLLQGKENRAETEHGITNEENERSALFSWITRKGSKIKKKLKKTGLKSKPFKANGYRE
EESEGTEALVNSSMSFSDDVSPVKRSKRATCLRKLEGKMGKSFVQVKRSKEPQEDFKRSMVQMILEKEIFETK
GLEELLQCYLTLNSPEYHRIIVGAFSEVWEFLFCDSHSNKAVQCD

> MELO3C009515_CmOFP19
MAAVKKKLLINTISVDIGCGSCRKSKSIISQIFRPKPKSPTSYSDRRLFRSLSSSSEKKLSDSDMAYAPEVVGGGG
FWKIGGVSVAVEKDSNDPYVDFRQSMLQMILENEIYTQEGLRELLSCFLHLNSPCNHGIIIRAFAEIWDGVFC
ARSAAPAKQRRHVRSRAF

> MELO3C004557_CmOFP20
MEKRFKLRFSRLFQSSFFSCRSKKNSDILITQKPISKPLISPKPPQLSSNYTPKLPLNPSHFQFPPPPASSPIISPPL
SHLNDCLSRHKSKTKIKRRKNPPSRPAPPPPPRLPEEDFGGAWWYGGHDDTEDETETLFSSRSLTSDSSVSRR
RHRQRHGRRRPPERKMRDGFFAVVKNSSDPYKDFKASMAEMVVEKKIFGGKELEELLQCFISLNSRHYHKVI
FEVYSEIKEALFFL

> MELO3C024573_CmOFP21
MANKLKKLPNFLNKSPWLWRSCTQSRTLSFRHPNDIFRTINSAYDEEEEDFYDYDDEDDEKETTQDDMSND
EDQIEALVRGLRVRQGKRLFLELDETNSIMTATATTTVAVATVGAGNYHVPFKESVAMAMESKDPYLDFKKS
MEEMVEAHELKDWKGMERLLSWYLKANGNANHEFIIGAFVDLLVDLAFAASSNLSNNSSSSPSSSSSSTTTT
SSLLCSSSSSIFPNSSSCSSCSSFRAPNSIISSNSVETADELECSCSSSSIRVAPPCLSSLFEDDEEEDIEEGF

> AT5G01840_AtOFP1
MGNNYRFKLSELIPNAWFYKLRDMSKSKKKNLQSQPNSTTSKKKHHAVSTPTSTTPLSPRPPRRPSHSSK
APPSHPPRKSSGNRLRHRATVDSKSSTTSGDSTTTETGSFSPDFRSDQVLLPDESLTGSWHSPCSSKLSK
TATFTPPPELELRPIITKTAATARKTAVNSPAGVRLRMRSPRISVSSSARRSGSSARRSRAVVKASVDPK
RDFKESMEEMIAENKIRATKDLEELLACYLCLNSDEYHAIIINVFKQIWLDLNLPPPHSK

> AT2G30400_AtOFP2
MGNYKFRISEMLPNAWFHKLKDVTKHSKPKNKASSSSSNTCSKKKPSSDSLPQHSYFSNSLVANNPPHHN
SPRNSLHTKKMSKRKTLYKPSLKPLTPPPLLVSASFNKSKINDQDSSYSLFPAIETSPESFVYSFYEEDD
DDEFVEFSNFKINTKNKAFTKQKVKVIDSVEKACTASKPIKKPQKSHLSVKISRDEDDDEYKAEKKYQRQ
VSSGRKPSAGINLKRVNSPRIQLSGTRRSTSRRSESKQDVLESFAVMKRSVDPKKDFRESMIEMIEENNI
RASKDLEDLLACYLTLNPKEYHDLIIHVFEQIWLQLTKTK

> AT5G58360_AtOFP3
MKQKMGTHKFRFSDMMPHSWLYKLKGMSRSSRKHQLSSPKHLSSADASSSRKLRDPLRRLSSTAHHPQAS
NSPPKSSSFKRKIKRKTVYKPSSRLKLSTSSSLNHRSKSSSSANAISDSAVGSFLDRVSSPSDQNFVHDP
EPHSSIDIKDELSVRKLDDVPEDPSVSPNLSPETAKEPPFEMMTQQKLKKPKAHSSGIKIPTKIVRKKKK
ERTSQVSKKKGVVKSFAIVLSSVDPEKDFRESMVEMIMENKMREQKDLEDLLACYLSLNSSEYHDVIIKA
FENTWLHLTQGLSISL

> AT1G06920_AtOFP4
MSPSEWFHKLKNMTKPRKKHSLPLYSINTTKKRKPSSESKSLPYSSTSYFFNRSRSRTSFESRILQISPR
NSLHNIQSKRKTVYKPSPPSSSIVSAGFNKTFHQSHDSLSASSNLKVISSEDDIIIDMNNRDFKKKTFKE
ITKFDSTEKACRASNRTKETHIPHHLSVKVSKEKEDEEEDACRTKKKHQKTLVSSGRRSSAKSPRIKLRA
RSPRIQVSPRRSKSRSQNKQILDSFAVIKSSIDPSKDFRESMVEMIAENNIRTSNDMEDLLVCYLTLNPK
EYHDLIIKVFVQVWLEVINSTFASK

>AT4G18830_AtOFP5
MMRWGRKKPVSSSSSSGLSRALPVSWFSKLSGSSDLKPAKEKKQDEKASQNISVKTSLGSTTRRSDIHEN
SKRFQRVSVEKENSATRSADKESNEKFEEIMSSVRKKVRDFQKETCGFLEVEAMDRDNGTVILTPRIQVN
RDKQRCERRDQRLLEQKPKRSEQDAGVKVKKPARRTGTGGYSREDSVILGHTITKPAHQWEKLKEVKLRE
VKLKADQQRKSLYLKRELNRIGTKGNNKVRVFSPRASEKCRVKAIEDLKKAKQRAREHELLIETADGGME
NESFAVVKCSSDPQKDFRDSMIEMIMENGINHPEELKELLVCYLRLNTDEYHDMIISVFQQVHNDFNFH

> AT3G52525_AtOFP6
MATKSKKKILKTVSVVDISCGNCIKPTFASIFNFFSKKPKRPSSTYRHCHSSISSATPSSTPLATASVAV
EKDSDDPYLDFRQSMLQMILENQIYSKDELRELLQCFLSLNSHYHHGIIVRAFSEIWEDVSSAAASAVEA
SPLITRHVSRASRDYYNYY

> AT2G18500_AtOFP7
MTKRFKLKISRILSFKSCRLKDPSSLPFNPVSSSLRRTSPPVNSSADVTTVPQRRRSSFRLHVLTVFGCG
RSSTPLDVDLRNSPVLSPPQTPTFQWESEGKWHVIAQVTEEEYETPRRKIYNGGSEKDNRRRLKKKEKSN
SRRRGSISSAEEETDRESLLPSSTNLSPEYSSSELPRVTRRPRQLLKKAVIEEESESSSPPPSPARLSSF
VQRLMPCTMAAAVMVEGVAVVKRSEDPYEDFKGSMMEMIVEKKMFEVAELEQLLSCFLSLNAKRHHRAI
VRAFSEIWVALFSGGSGGGRRSSSFSSVRLSDYDEC

> AT5G19650_AtOFP8
MEKRMKLRVSRIVRSSLSSCRPRDLYDVVETCAVTSQATSSERFFVTKAKTKTPSRPKSHASSCPRASPI
FPPNPFYEESRSFRDLRKKVKTNRKQRSQFGSDPLFASRFKSTGSWYWSCSEEEDEGDKEESEDDDSDTL
FSSRSFSSDSSKAESFAVVKKSKDPYEDFRTSMVEMIVERQIFAPAELQQLLQCFLSLNSRQHHKVIVQV
FLEIYATLFSP

> AT5G22240_AtOFP10
MLNLQAKLNEKKVPLLTNFPTPTSSLNNLNLFSLPLVKKKMSFKKMMKYILKTIFKPIFMACGCGSTVPPSSH
SHYTPGPPVSPTVLRSPCPKIDESVAMAKESINPFEDYKKSMNQMIEERYIETESELKELLRCFLDINPSPQHN
LIVRAFVDVCSHLQPPHDRRGKSLGRLLRLYVNNPLDNNDDDSHQTSSK

> AT4G14860_AtOFP11
MSNFLRKKLHLCFSSSGGLSPSIPSSPIIVSNHNAQSHPHHTPSIFINNFNSLYDQLSVSSPLHRRHSSENPAGV
FSTNRREEEEEDETTTSVSKLLSGGTAIMKHIESPDPYRDFGRSMREMVEARDLTRDVVADREYLHELLFCYL
YLNPKHTHRFIVSAFADTLLWLLSPSPSPEHFLS

> AT1G05420_AtOFP12
MPRVMWKNFHLCFPSNLTKPSSSPSGATSDDPNRPSILLINNFNLLYDDSSAAHRRLSKPLIHDVEPSSTFTAS
TSTAANSSSSSASYDDSDNYGFAPDDDSPPPDLTAVLASRRFFFSSPGCSNSITDSPDLRCRDNYDTATRLLTGG
TAVKHYVQSPDPYNDFRRSMQEMIDAVTNAGDLRRYEFLHELLLSYLSLNAADTHKFIIRAFADILVSLLSDGH
RIS

> AT5G04820_AtOFP13
MGKKKMKLSSLFKGGAGGLLAVPLCYNAKTLSFRVGDDMIKTVNSVFFDHHHNNNNGGDLLEAETPESWF
TNSSETASHSTESDQDLDAESLEMVVRGVVRSERLFFDPGVTSSILEEIEEKSKSDLKSKETVAVGEDRSTPIEEI
SVAVAMESEDPYGDFRRSMEEMVTSHGELAKDWESLESMLAWYLRMNGRKSHGVIVSAFVDLLSGLSDSG
AGITSASVSDSARYSTAVSSLPSSPVYSLSQGQTEIQEEERRSC

> AT1G79960_AtOFP14
MPNPLQKSLHGYLSKIKKETGKLQLSSSHSFSSSKNWVLGKHPKKLSFSFKHRRRSSKTRFSKEEPVYHQDSAH
AATLSDIDRFLEENFKSLCIRDDQEDDQHQARVTKNKEKRESSSDDSDDDDDDDDYRHRFERTWGHAVYDS
PKQPPDLLRTERLSPPPGSSEGRPSMETTSTSSERQSRSTLVLPENCIAVLRYTDEPQEDFRQSMVEMMESKL
GMRESEVDWDLMEELLFCYLDLNDKKSHKFILSAFVDLIIALREKEKRITRKGHVRSLSTRAARDRLRKRMIM
SDN

> AT2G36050_AtOFP15
MKLPFLNKNHSTSSYSSNSSSSSWPWPSCNQNPKTLSFRATITFTNPIHDQDDDELDLLDPPEITDSVENVIK
GLRSSERLIFESKGETNSILEETATSKREEEDEEEGFMLFSLESDDPYSDFKRSMEEMVEAHALHHDWKSLEKLL
LQFLKVNAKTSHRYIFAAFVDLLMNLALDTKKAIINNDISKDDGVSASRAAAAGEASTSCCNSMTLGESPSSPL
SFYTSCSSSSSSDETSSMSVRFLPLSSLLEMDEKTKEILV

> AT2G32100_AtOPF16
MPKILWKSLHLCFPSNLTKCYSSPCIPPSSADPDGIIQPNRPSIVLLNNFNLLYHNDNHHHPHRVIDLPSSSTTT
TPAATSSSSTSSYESDISPDVSAAFASRRFFFSSPGRSNAITDSPEPRSREFSDNYDDATITSTKKKKKKVYDNSV

TTTTTRLISGGTAVTQHVDSPDPLTDFRRSMQEMIDAAIDAGELSRDPNDGYDFLDELLLTYLSLNPADTHKF
VIRAFSDILVSLLSEERRIC

> AT2G30395_AtOFP17
MRVKATLINFKSKLSKSCNRFVSLFRFRVKRPVFIRPLRARHGNVKPRHQHHHSKKPICSCLCFLNSSKNHKM
SNAKHRSSSFSVNDDDYSKFMQSPLTPATAKKLFTSPITTPYSSRTRKSLNARDTFEDNAVEDACRSFENYLIHL
IVEEGKIDDLMDIEELLFCWKNLKSPVFIELVSRFYGELCRDLFSGE

> AT3G52540_AtOFP18
MVRKMKLPFLNKNTSSSSFSSNSSSSSSSWPWPSSHQQNLKTISSKASFIVNKPKDVYEPEPPPRSFSSSPSSS
SYSSFSSTSHAIENPPEIESIENVIKGLKSSKRLIFERRGTSNSILEEATKRDDHEEEEDGLMLLSLESNDPYTDFK
NSMEKMVEVHVLHHDWISLEKLLFWFLKVNVKASHRYIFAAFVDLVLNLAVGPSKDVAGEPNSDVVVEDSL
SSSWPVSLYSSSDENSSTSVRFLPETSIGEKGRDVCCLSSLFELEEKIKDNIDPNDYVSS

>CsaV3_4G027080_CsOFP1a
MRNHKFRFSDMIPNAWFYKLKEIGGASRPKSFRSNKNPHAHPPPPPPPSKHKQQPPPPPPPPHSRSRKSYY
FTRQLQSNDAYFINSPPPSPPFYLYQSPRERRKQTSSRSSAKLLSSSSLACSCHTTAESIWTKSDSPPQFSTSPSD
TSPDFRTDKILTAEASEHFEHDIVIDVSSNYSNNAVIGAFDELELPPIITKQKKKTETKQRTTTTTTTGTKKVAGN
SPGVRLRIHSPKIGYRKMGGRKSVSSRRSLSESLAIMKSSYDPQKDFRESMVEMIVENNIRSSKELEDLLACYL
CLNADEYHDLIIKVFKQIWFDLTQPSPPPL

>CsaV3_3G033200_CsOFP1b
MGNYRFRVSDMMPNSWFYKLKDMTTIIRRRNSKKDQSSKNSHTTDLVYSHPRKSIHFTPSQLAANNSPLEP
PRRSSKGKKPRRRPTSAAAPTSTLLLTSSSGCSCGRTALESVTTTSTTTPPVLTHHSYLHAEKEEEDPNAVIFGKE
HKISPKKINGSDEEYLKSLPQIIDQLPPIITRSSSSSSNAADASTCPSLTITKNDKSEPIRSSPSRRFLLNSPGPKLRI
VNSPRVSSSKRFSHVSRRRSGKRSLNDSLAIVKSTKDPQRDFRESMVEMIVENKISGSNELEDLLACYLSLNTD
EYHDIIVKVFKQIWFDMTDIIGDHY

>CsaV3_6G051110_CsOFP5a
MKWGRRKPHNPSSSSSTSSSSSSRPSFMSNILPASWLSKLKQKKSNQEARPRKVKGTEKRSSPCIQSPDFANV
TPSPGQVNGNRNRLCTGDNGEFWKLPFGGEDIDVKKSSEILRSVWYNSENEHDLPRTSCRSCRTKYTEFEGN
EEIQNLDDMVSRMTRRRRRRREAPIQVKLLRRESETESTTPRSKYRENGNFGNFGKKGVEKKGFKPERETDK
GKEIRARRLVGKKMLGVEEESGVRKNERDKTKLTNSRKHRYVPSTMSKSSNLGTIEENCVFSSMKAEESDGH
DTLGIEIDSDWERMKELKIEELKLRYEKQRQPLYIRKDSNEKNPKGRRKIRVYSPRTANKIEICKIKALEDMKKAK
LKMKKKVKESTVEDDTDLESFAVVKSSFDPQQDFRDSMVEMIMERRISKAEELEELLACYLTLNSDQYHDLIIK
VFRQVWFDLNQAALESELHKQFPCNEQLV

>CsaV3_6G048660_CsOFP5b
MKAAGSTQHHHQEQSAYRALCCGCSCNCRLSFSSSEETESFNSDKFPSVSSIAHAMVQERLEQMIREKREVR
NGKERRKQRSEDTKFVVMVAMEKCSDDPKEDFRVSMTEMILANRIEEPKDLRNLLNYYISMNSDECHGVIF
EVFHEKEVSKNPRDDRILKSKLEFD

>CsaV3_6G040960_CsOFP6-19a
MAAPRRNLQLTSLSVDLNICRPKLLSHFFHHLKPKPSPKSPNHHHHRFSSASSDSESPPFSDSDSETRTSITFR
GFGRSGGESVAVEKDSDDPYLDFRHSMVQMILENEIYSKEDLRGLLRCFLQLNSPSHHGIIVRAFSEIWDSVF
SSTSPILRF

>CsaV3_3G037720_CsOFP6-19b
MAAVKKKLLINTISVDIGCGSCRNPKSIISQIFRPKPKSPSSYSDRRLFRSLSSSSEKKLSDSDMAYAPEVVGGG
GFWKIGGVSVAVEKDSNDPYVDFRQSMLQMILENEIYTQEGLRELLSCFLHLNSPCNHGIIIRAFAEIWDSVF
CARSAAPARQRRHVRSRAF

>CsaV3_1G015160_CsOFP6-19c
MSNIRKNKFFTTIFSSTAGCGGCQKPKLSDIVQPDKKPPTTIVRRSSSSSSTDQNGTFSLDEDYTSSASKSTGT
QSPVAILIGDSIAVEKDSDDPYEDFRGSMVEMIVEKRIYSPNGLQELLNCFLHLNSPYHHEIIVKAFTQISNEFE
SSHRLWNMSNNTDWKRRSGGEG

>CsaV3_2G002260_CsOFP8a
MEKRFKLRFSRLFQSSFFSCRSKKNSDILISHKPISKPLISPKPPQLSSNYTPKLPLNPSHFQFPPPPASSPIISPPL
SHLNDCLSRHRSKSKIKRRKNPHSRPAPPAPPLPRPHPEDFGSAWWYGGHDETEEDETETLFSSRSLTSDSS
VSRRRHRRRHGRRRPERKMRDGFFAVVKNSSNPYMDFKASMAEMVVEKKIFGGKELEELLQCFISLNSRHY
HKVIFEVYSEIKEALFFL

>CsaV3_1G024220_CsOFP8b
MDTRFKLRLSRVFQSSFASCRSRNLSDILHKAVFIPSSSDDASFRKISPSETSSDFLLPRRKISHRFPLSPFPSAIS
RPRTCPPASPISPSKPISQKTTTSTKKKKKQKKQRKQRKQSKKEIPFSPFRSSNFGGTWWYSSEDEDDDDETD
TLFSSKSRSSDSSASHRRHKSRRRRGCRSRGSEMGVLPLKGKVKDSFAVVKKSSDPYNDFRMSMLEMIVEK
QIFSAKDLEQLLQCFLSLNSHHHHNVILEVFTEIWEALFSDWGS

>CsaV3_2G028590_CsOFP10
MARSFKLKVRLFISFFRFCRPKYLNALTFPKTSSPENSPYRKQIPANPESCYSCYPNPPSPPPSTPVDFFADPSK
EKSTSICSSCKLKSYARKNGLLQGKESRAETEHEISSEENQRSTPFSWITRKASKIKKKLKKTGLRSKPLKANGY
GEKESEETDALVNSSISFSDDVSPVKRSKRALYLRKLEGKMGKSFVQVKRSKEPQEDFKRSMAQMILEKEIFEI
KGLEELLQCYLTLNSPEYHRIIVGAFSEVWEFLFYDSHLNKAVQRD

>CsaV3_7G027990_CsOFP12-16a
MPTKLGRNYLNLCFTKIKNPLSTAQSSPPITHTADRRRQTTRSFSSTAAAFITNYNSLYEITTTTTTNSDSNSPS
TPLFGLTNDIGVADPDAYVAVDFITAFTSHRFFFSSPGSSNSIIESTTTTTTESTTTMSLSSEYSARYEGNDDDL
MIFNNSHVIPTYSPDPYMDFRRSMQEMMEAREKMTTAVATTTTMKKSSWEFLHELLLCYLALNPKTTHKH
ILKAFADIATVIKPPLAMKETEEEENVDREKGESMVDDRGAGGGGCECEMSGQQNDRD

>CsaV3_3G013290_CsOFP12-16b
MSSLKKRNFHLWFSKLRCFPATVKPSSPPQTPNKKPFSITHLENSDYSSTSTAADDGFFSDDSSSDSDAIVPD
FSAAVASHRFFFSSPGCSNSIFDSSPDTHHSTAVSAAVHGGVEVRKVSMDPFVDFRASMQEMVEARDRPV
DVRRDWEYLQELLLCYLQINPVDTHKFIL RAFSDLVVYLLESSPESFSDRRIRPHNINSNSW

>CsaV3_7G022480_CsOFP12-16c
MSNLKFLNNLYSFFSKLKFSPPPPVIASHTPPSDCYFTSNPISSTTADDCDDFFSTSSDADDSISDDLAALLASR
RFFFSSPGRSNSIFEYSSCSRRQQPHDVLVSEGHRIRKYSMDPYADFRRSMQEMVEARELEDVRSDSEFLREL
LSCYLRLNPKNTHKFIVKAFSDLVLSLLAS SSPTPAPASIARRKVVTSR

>CsaV3_3G037710_CsOPF13a
MLTKKKKMMMMMRLPSLFKYLAIDDKSTFPWPSCRQPRTLSFRTTSAAVATATDSSDSFFTLSSESSGSLST
VSESSGGDPIERMIRDLRSTKRLHFEPTGKSSSIVEDDTVSHPLKEGTTVMSMDSDDPYSDFRKSMEEMVEA
HGMKDWESLEELLNWYLRVNGKKNHGFILGAFVDLLVSLAMASSSSSSSCSSSLCCYSSSSSSSSLPCVSSSM
EIEEISSLDEHHHHVYS

>CsaV3_5G035260_CsOFP13b
MANTKKKHHLIKNFPSIFKSKLPTSHPWEWPDWPSCKHPKTLSFRSQNDLVFKTVNSIFFDQPFETTTTTTTP
DYSVSATNSTPAVDSEESLETVVRGARSERLFFEPDDTSSILEKSKSIDSVETELLPKSGFKESLIVSIESENPYED
FRKSMGEMVESHGVKDWDGLEELLGWYLKANWKNNHRFIIGAFVDLLIHILLASSSSSSSSTSTSTSSSSSSSL
CSNSDSNYYTCTESSSSSCSCSSSLRSLNPSSIRKELDHDQVEDNHDHITETL

>CsaV3_6G004380_CsOFP13c
MGKKMKLLPSLFKNRASPDRPWQWPLCGPSKTPSFRAGPDDHQIFSTLNSIFFDNFFSDPIHTPDSWFATS
SLFESARVSLSTEFEDDLELVIRGAKSERLIFEPGETNSILEKSRGVEEGGKCEESIRFEGSVVVLMAMESEDPYL
DFRRSMEEMVECHGIRNWEWLEELLNWYLRMNGMKNHGYILGAFVDLLVDLGGGDGSTDSTSIFSDDLII
QRHDRERCDV

>CsaV3_6G040950_CsOFP13d
MANKLKKLPNFLNKSPWLWRSCTQSRTLSFRHPNDIFRTINSAYDDEEDQDYYDYDYDEDDEEETEGDDM
SNDEDQIEALVRGLRVRQGKRLFLELDETNSIMTTTVAVATVVGGNYQVPFKESVAMAMESKDPYLDFKKS
MEEMVEAHELKNWKGMERLLSWYLKANGKANHEFIIGAFVDLLVDLAFSASSNFSNNSSSSPSSSSSSSTTT
TSSLLCSSTSTFPNSSSCSSCSSFRAPNSIISSNSVETAEEIVELECSCSSSSIRVAPPCLSSLFEDDEEEDIEEGF

>CsaV3_7G033180_CsOFP14
MPKKLQKSLQDYLSKIKKPTPQLQFPNPQTFSSSKSWILHGCKHPKTLSFAIDRKQVDAVGNKEDAAATLADI
DRFLFENFRSLYLKEDGDFSDRKVVVGGGGGGGGGGGRDCKNHRGRVVSPESPVDSYGGSHRFFFSPDLS
GSDLPDDSHTESSENAGSSSSSLIGEDRGKDLKLPSDCIAILRKSPNPSEEFRRSMQEMMDAHLKQHEKVD
WEFMEELLFCYLNLNEKKSYKYILNAFVDLIVILRQKAEEAPAKPRTVRSVRMVRRMI

>CsaV3_4G005140_CsOVATE
MMMTPKRFKLLRIPSFHCCRSNDISVVPTDPPSPPPPPKPHHSSLRRHVSSAFRTAACGCRSSSTNSDDDQI
CKSSPTLPTHVPPTPLLHSFDDGSTFPKRQRRRKNKKKSKSKSTTLTRLRTSTSSTESGLFSSESFDEIDELEETET
LISSSKTISTSDDDNDSSSEFNPQLETIREKPNKINLRRKKEKEKRRRKQKRTTIISPSPEIESPARLSVFQRLIPCT
VEGKIRESFAVVKKSADPFEDFKRSMMEMIMEKEMFEEKDLEQLLHCLLSLNDREHHGIIVEAFSEIWQSLF
CN

>Solyc02g085500_SlOVATE
MGKSLKLRFSRVIASFNSCRSKNPSSLPQNPNFFPHKLTSTKHISPDFPLIDQNQNQNHRNYVPESTMISVGC
CRSEFKWEKEEKFHVVSSSFVSEEEECEEEINLALRPPLTPPRFSRIVVEKKKKKQQRVKKTKTKSRIIRMSTSSA
DEYSGILSGTNTDWDNNEEETESLVSSSRSCYDFSSDDSSTDFNPHLETICETTTMRRRHKRNANTKRRSIKQ
SRPSFSSSKGRRSSVSTSSDSELPARLSVFKKLIPCSVDGKVKESFAIVKKSQDPY

>Solyc01g007800_SlOFP2
MSTHRRRIILSNVTVKLGCSSSCIRPKLSSIFHPKPRKSPKSQTQNKNYSNYSSCSSWDTTTTTFSPNSDSTTNE
SSDFKTSKAVQGFGRIGGESVAVEKDSDDPYLDFRQSMLQMILEKEIYSKDDLKELLNCFLQLNSPYYHGIIVR
AFTEIWNGVFSLRPGVAGASSPFLHGGSHVTYR

>Solyc01g007810_SlOFP3
MKLSSLFKNSSQNSSSTTTTTPWPWSLPTCGKPKTLSFRLEKNQHNIYNSTFHLDDINDTTSCSFDDFFSEIDE
TSSSSTTTINGQDCIEKVIKGLRLEKERLFFEPEETSSILDFQENKNISITSSNININVVDEGNIISFVPMGLDSND
PFVDFRKSMEEMVEAYEIKDWENLEELLTCYLKVNCKSNHGYIVGAFVDLLVNLATFSDNNNNVGVDIGAGV
GAGVDESTIIMTTIDEEQQCLSSSTTTTTTTTNHSFTSPLSFCSSSCSTSSSITSTSACLSLLLEEDEVIQTKKH

>Solyc02g072030_SlOFP5
MKWGKKKPSSSLMTHVFPVSWLSKFKQKKVCRSEDQEGAKMRKVDLRTNVCLKQGRFYEDDPYWRISFSE
ENHPQNPLWCGECDQNSKSSLGEENHKFNDMVSRKISEKPKNEAEFSNRKRNSVKDEKLRKLSRKALEERIA
ENAREEVTEKDIFEIEPEDEKVMKRGKEKPTAYKSRKARSLSYNDSSPNSVEESCMMFTSLNLEEEADALSEEE
FESECLKIKEMSEKSGCQQRKSVYINQKRRRKHGIKVRAYSPRTAKMECRIKALEDMKKARMKTRHETKESFT
GDRTVFDSYAIMKSSFDPFSDFRDSMIEMITQRGIKSSEELEELLACYLTLNCDEYHDIIIKVFRQVWFELNQINI
GEELQKCCCSDE

>Solyc03g034100_SlOFP6
MAKLLKFRISKAISNSFHSCRSKDPCTLPQHPVPSFLQNTQFITDDHLLFEEMIQQNNESQLISTHHEHFPIITS
PSFKHHVSVTPITATGQCSSRNGEAFSTTSDDSHTRSPSHEFKWKKEEDKWQHFIKTNSDDDTKQQPRRKIS
YSFSSDNSDNDKILIEIKKKISTSKTNFFMMMSTTSSSSSMDENEINFTSKKTKWDYHEDIDITNEDEENETET
FISSSRKSHVEFPDDSSLNFSHEFDTIYKNTTRRCQKKIGYSKRRDHVKNTRSRSSRDMNNIGRRSSISTSTTSS
DGELPPRLSVFKKLIPCNVEGKVKESFAIVKKSEDPYEDFKSSMMEMILEKKIFEKNDLEQLLQCFLSLNAKNC
HGVIVEAFSEIWETLFSPNHN

>Solyc03g120190_SlOFP7
MPKQLQKSLSDYLTKKKKKATAQQTTNSANKTLSSSTSWLLRGCRHPKTPSFSAVDRKEKNVQGENEAATLA
DVDRFVFENFKSFYYKDDDNEAEIVENPNSLSESPRHIIPPLNHTGSRRFFIAPGSSSSLIEEARTSMTVSDDTG
STSAITITTVTNTNSNELSAISTEYSKETLNANDFITLVTYSPSPYDDFRQSMQEMMEARLKDQGKINWEFME
ELLFCYLDLNDKKSYKYILSAFVDQIVILRENSGRVPAISRNVRPLDGELNQRDT

>Solyc03g120790_SlOFP8
MEESYKKIEPQMLLKKTIQKTKNFLYRTPHNLKSFLFGGHHKLPKTACHFNPFLSVSKRFSSSKRIPKTNVKELD
DLYRDYYQQWNQPDHNEIQERKMTSKNARKFQGMAEGDYSESQRELAVRFGMEDIESERRKEDEKKGRE
VLTRSTSKGSLTLLKKMEELEMVEGEDMDHVLDIEEVLQCYTLLNSPVYVDIVDRFFMDMYTEFSIRKPSGSV
NSSMRRLGPLKL

>Solyc04g080210_SlOFP9
MTRRFKLKLSMPSFRFCRPKKASFLPKSPMPLSLYKFSPANILDNSPVPVPPSTPHHPYILRKAHNLASKTYNS
PSSEYSDPDNNNMRRGESRKSRLNMSFSSVDSGWFSFNSECCDEKPNDETESFMSSPSFESSFDVDHGIDP
LSGIRRKKNNNNTKVRRLRRYLSNSLKDSMMPCMADGKVNESFAIVKRSVDPYDDFKNSMKEMIMEKEMF
EAEDLEQLLLCFLSLNSRHHHAIIVEAFTEIWEELFGKSSKSMDLKLPRFQ

>Solyc05g055220_SlOFP10
MAKKLKISSIFKKKELGLATWQWPSCTHSKTLSFRGDDNIFKTINSVFFDPFDGIETPQSYSTNSSLDTNSISIES
HEEIIKGARSERLFFEQVATSSIFQEPQEENQENDLPFKESVILAMESKDPYLDFKKSMKEMVESQGIKDWDN
LQELLACYLKLNGEVNHGFVLGAFVDLLVELVIPTTPSTNSDNSITSYSSVASSSFSCPSSPLSSLGHKETEEQEN
AKVS

>Solyc06g073040_SlOFP11
MVNYKGGKLLKHQRNRVSFSAKLPEDVRGAFADSTCVVKYSMDPLTDIKESIKEMVKNVGIKDWKEMEELV
YCYIVLNSEETM

>Solyc06g074020_SlOFP12
MTRKYDQCLVDNMFGPFPESCCPDEALEMAKQALATRRLSFEENESCSVLSMVGFPFKDCLLLAVETENPK
MDFLHSMEQMTKVYGAQRGDMVDWEFMEELLTWFLKINNMKNQHFIVAAFIDLCLGGHVQDVEPVEN
VEPLTDDIVNVILEELWWGL

>Solyc06g082450_SlOFP13
MNTSCCLKFNNPCKKIVKLFKFKLRKPLFIRRLRIFRPSTRCESNTSTRRKQASQVLSVFRFIRRSKPREEDQVM
ALKSFSGHIKAPVPSPITPAYARLSGATKKEVVIFQDDVEDACRSFENYLAEMIVEEGKMRDIMDVEELLYCWK
NLKSPVFIDLVCRFYGELCKDLFSHTYKDDINSPQKIMQ

>Solyc06g082460_SlOFP14

MGNHKFKFSDMMPNTWFYKLKDMSKTKNHKSPFSSSSTNKSQYSQPRSSFSYTRRSIRVDKIYNSHSYNFL
DQPRRSSSSSSKKKSKRKTIYKPSPKHIPSSVTNYVSVSNKLNTSSSVYSTEEDKFPELDFLNSPSSEFDSVDSQT
FNELPSTWPNSCNCHFTSSATDIIIDVNDKALSNEFHNLTTEYAEFSDIDQLPPIFTKASNSIKNIKQDENVKAQ
REKEPKNRVGSPVSRKHYSSSSGVKLRTNSTKVANKRNSVSSSKRRSKTKKESCSASRGTSFAIVKASIDPEKDF
RESMVEMVVENNIRASKELENLLACYLSLNSNEYHDLIIKAFEQIWFDLSDLHL

>Solyc07g055240_SlOFP15
MQNSKAAVAAAAKKQKLKGCSALCCSCRLSVSSSSEEAESSSSSRYPTISSLTHAMVQERLDKMIREREEAKN
EEMRRRRRRAERDEKTKFIVMIAMEKSSYDPREDFRESIEQMIIANRICDPKDLRRLLNYYVSMNAEEYRGVI
LEVFHQVCTTFFLSCKQPSSQV

>Solyc09g018200_SlOFP17
MGNYRFKLSDMVTSSWFYKLKDMAKSRTQIKRKQTSSSTSSSSFSIFYSSSNVQQHHRKSYYFSRTLSPNPHQ
SNVTPMKSSKKRKNTRRNTPKFVNSPKSMILSPSHRRCNDHIFDSVSKIDLPPILTKPNKKEEKTELKFLTVKTE
QSTSPKRRISVSSSSTGVKLRTKSPRIISRRSVGEKSYAVVKSSKNPQKDFKESMVEMIVKNNIKTSKDLEELLAC
YLLLNSHHYHHLIITVFKQIWFDLQLK

>Solyc09g065350_SlOFP18
MPRTTLGTNFNLCFTKLKRSLPLRSIDDNDNDNDNERHQQQHHSMHFCNSVKNFNSLYDLSSSECNIPTSSS
TDESDYNYELENNTPDLATIYASQRFFFSSPGHSNSIIDSSSSISSSIASTSSSVGSDAPLEGASRFQRIHPTRIWIF
DDRCKKWWRHVD

>Solyc09g082080_SlOFP19
MPRILQKKFYHCLPSFKCLPTILSLPFEETEEEKTEQKKIKNFNSVFDIPSSDSATTSKSLTNSSTTTTEEEDNNTN
CTFTSFEDSDYTNIPDFSNIFASQRFFFSSPGNSNSIIDFPPENPKVVTGGVAVQTYSPDPYSDFRRSMQEMVE
AHELTNVKANWGFLHELLLCYLNLNPKHTHKYIIRAYSDLVVSLMSMDDSEKKTEGIARP

>Solyc10g076180_SlOFP20
MGNYRFRLSDMMPNAWFYKLKDMAKSSSRRHSHTTSSSNLQLDKKRQPHNNLGCQRKSYYISRNLTITSPI
SSNSPKLDHNVHITEPSRKSYKKRRSTNFRRRNSPKPVNSSASVESVWTKPDSTPEQYPNSSSSSSSSSPSSILP
HKSNPIASISPSCDCRTDYTNQNSANLDPGVHSVSKIDLPRIITKPEKFNEKIQEKQRIVKQEQRIVRRVSTNGV
KLRTNSPRITTTTTTNSRKSVSSKRTSVTTDSFAVVKSSRNPQKDFRESMVEMIIENNITTSKDLEELLACYLSLNS
DEYHDIIIKVFKQIWFEITEIRLK

>Solyc10g082050_SlOFP21
MSTTKKRVILRNVTVKLGCSSSCIRPKFSSIFHPKPRRHHSADSAAVFNHHKTPNNKYSFSNSTITTATTFSPSP
TPSPAHYSSDAERAVQGFGRIGGESVAVEKDSDDPYVDFRQSMLQMILEKEIYSKDELRELLNCFLQLNSPYY
HGIIVRAFTEIWHCVFSVNPGVTGAESPFL

>Solyc10g082060_SlOFP22
MNLSSLFKSKKKSSFSPFLCPLPHCGIPKTLSLRVENNDNIFNSQRLYNNVDDDMVDKMVEGLKIEKDRFFFE
AGEKTSSIMKVSSSILAKSNNELEILPIDESCVITPIDSMDIPCGEGAISIRDQVSSSTLSNNTNNSGKQVEYLPF
NDSCIIKLSSSMDPYGSFKKSMVKMVEANLGIKDWNEFLEEMLAWYLEVNEKNNHKYIIGAFCDLWISYSFT
SSTTNIPNSFLFSSSEPKSVISPTSTSFVIS

>Solyc10g083070_SlOFP23
MKFSSLFKSNKKPSFSPMLCRLPRCGDLRTLSIRDENNHNIFNSQRFYNNVDDEMVDEVIESLKLEKDRFFVE
AGQKTSLILDMSSSRLSKKRTISKRLEFLPFNNDSCVITSMDSIDAYGETSRSILEGSSSRLSKSTNNSTSSKRLGY

LPSNDSMDSYGDQETSSILDMSSSSSNDNISSKGLGYLPSNESMDATSILERSKSNSSHGFVYYVPCKKTYAIM
RLISRDPYEDIKYFLEKMVDENLEIEDWEESLEELCGWLLEINEKNIHKYIVGAFCDLWMSYSCTSTINTPFGFS
SSKPPSLYFMSLIENEADRMIAASTSSVTP

>Solyc10g083080_SlOFP26
MEFFSLFKSKKKPSFSPMLCRLPRCGNLRTLSIRDENNHNIFNSQRFCINVDDDIVDEVIEGLKFEKKRFFFESG
EKTSSILNVSSSKLSKSIGNKRLEFPPSDESCVITHIDSIDAYGETSTRSIFKGSSSRLTKNDYSTSNNKFESLPLNN
SCVISPSAMRVTSIDPYGYIKKYMEITVEENQGIKDWKESLKEICAWYLENNDNDKNIHKFIIGAFCDLWMSY
SGTSTTNTPFGFSTSEPPSPYFMSLIEAKADQIIATSTSSVIP

>Solyc10g083090_SlOFP27
MKFSSLFKSKKKPPFSPMLCRLSRCGNLRTLSIRDENNHNIFNSQRFYNNVDDEMVDEVIENLKLEKDRFFVE
SGQKTSSLLDMSSSRLSKRRTISKRLEFLPFNNDSYVITLMDSIDAYGETSRSILEGSSSRLSKSTNNSTSSKRLSY
RPSNDSMDSYGDQETSSILDMSSLSSNDSISSNGLGYLPSNESMDATSILERSKSNSSHGFVYYVPCKKTYVIM
RLISRDPYEDIKYFLERMVDENLEIEDWKESLEELCGWLLEINEKNIHKYIVGAFCDLWMSYSCTSTTNTPFEF
NSSKPPSLYFMSMIEDEADQMIAASTFSVIS

>Solyc10g083100_SlOFP28
MKFFSLFKSKKKPSFSPMLCRLPRCGNLRTLSIRDENNHNIFNSQRFCINVDDDIVDEVIEGLKFEKKRFYFEA
GEKTSSILDVSCAITHIDSIDAYGKTSTRSILKGTKSRLSKNDNSTSNDMVESLPLNDSCVITPSVMRVTSIDPYG
YIKKHMEMMVEENQGIKDWKESLKEICALYLEINYIDKNIHRFIIGAFCDLWMSYSGTSTTNTPFGFSTSEPPS
PYFMSLMEA

>Solyc11g006670_SlOFP29
MGKKMNLGSWQWPSCTHSKTQSFRANHIFKTINSIFLDPSNTDHHHHGVVEIETTPESWFTNSSESASFST
ESEETGEPLMELIIKGVRSERLFFEPNCTSSSILEHQDQSQDQNQNQNQSQSQSRDQDQSQSQSQEKLKEIE
EDVDEELPFKESVALALESEDPYLDFKKSMEEMVDTHEIKDWESLQELLQWYLKMNGKNNHGFIIGAFVDLL
IGFTPSNCDSITCYSSAASSFSSIEEKGE

>Solyc11g068780_SlOFP30
MSSKNKKIWNCITSNGTAGCGCSKPKLSEIIQPKPKPRPEPEPNAHSSSTSNSDSPSPTIMPAKIVGSVAVVKD
SDDPFGDFRRSMLQMIMEKEIYSYDDLNELLNCFLQLNSPSHHDIILQAFMEIWNNGKNYIAN

>Prupe.6G290900_PpOFP1
MGNHKFRLSDMMPNAWFHKLKDMSKPRKNPNSPHPSKKKKQQQKPTFASTAKFTEPSKPKQQLPHQCL
PRQSYYFTRELTSAAPGHRFCSSSPTNPKASDTNFPDPPPKKPSKQKPKKRITSLPSDPHLVTSSVSAGCGCRA
PIESVWTKSDSPPELWSSSTLDSSPEPESHDEDDGELELHEPEFRCDRVLATETFDGMVSMSSSCAAYLADSE
EKDVVIDVDKASLSMKLSDVKLSDMADNGLYSFSELELAPIITKPPKFSEMVRDVKKKKETKEPSRCRRSSAKF
QDRNAHGSLSVKVAKEESTSTKTIKEQRTASSVRRVSSNATSPGVRLRMNSPRIANRKINQANLSRRSVSSNS
SSKRRSLSESFAIVKSSFDPQRDFRESMVEMIMENNIKASKDLEDLLACYLSLNSDEYHELIIKVFKQIWFDLTD
LRSK

>PGSC0003DMG400030384_StOFP20
MGNHKFKFSDMMPNTWFYKLKDMSKTKNHKSPFSSSSTNKSQYSQPRSSFSYTRRSIRVDKIYNSHSYSFLD
QPRRSSSSSSKKKSKRKTIYKPSPKHIPSSVSNYVSVSNKLKSSSSVYSTEDDKFPELDFLNSPSSEFDSVESQSF
NELASTWPNSCSCHFTSSATDIIIDMNDKAHSNEFHNLNTEYAEISDIDQLPPIITKTSNSIKNINQDENVKAQ
REQEAKNRVSSPVSRKHYSSSSGVKLRTNSTKVASKRNSVSSSKRRSKAKKESCSTSTGTSFAIVKASIDPEKDF
RESMIEMVVENNIRASKDLENLLACYLSLNSNEYHDLIIKAFEQIWFNLSDLHL

>CA10g10680_CaOFP20
MMPNAWIYKLKDMSKVSSKSKGHSHTTSSSSTSSQHSSLNLQPDKKRQPPHKLCCQRKSYYISRNLNPSETF
PHNPVSSNSPKASDNFHFTEPPRRSSTKKRSVNRRRNSSSPKLVTSSVSASCSGRASIKSVWTKADSNTPEEY
PNSPHSSSSSSMSSSLSLSSDKIQAPKKLDPVTSISPSCIKDTDNLDQKFHSISKIDLPPIITKSEKFDQNQIIIKQE
QSIARRASSGVKLRTNSPRITNCRKIQASRKSVSSRRTSVTESFAVVKSSRNPQKDFRESMVEMIVENNIRAS
KDLEELLACYLSLNSDEYHDLIIKVFKQIWFDITKY

File S4. Individualized in vitro melon plants, Cas9-targeted PCR result, edition predicted by Synthego® v3 and ploidy tested by flow cytometry. Plants having more than 50% editions are marked in dark green, plants having more than 25% are marked in pale green, plant selected as T0 for greenhouse is markerd in bold.

										Ploidy				
Generation	Plant	CAS	Guide	Editions predicted by Synthego® v3 from sequencing data						2C	4C	8C	3C	6C
T0	1	+	g1	78% +0	5% +1	4% -1	2% -3				93%	7%		
T0	2	+	g2	55% +1	20% +2	22% -6					88%	12%		
T0	3	+	g1	95% +0	3% -3						86%	14%		
T0	4	+	g1	95% +0	3% -3						91%	9%		
T0	5	+	g2	54% +1	20% +2	23% -6					94%	6%		
T0	6	+	g1	91% +0	5% -3						89%	11%		
T0	7	+	g1	90% +0	4% -2	1% +1					92%	8%		
T0	8	+	g1	95% +0	3% -3						78%	22%		
T0	9	+	g1	88% +0	3% -3						89%	11%		
T0	10	+	g2	23% -4	20% +1	20% -3	24% -9	4% -3			100%			
T0	11	+	g1	71% +0	9% -10	2% -13	2% -4	1% -25	1% -1		88%	12%		
T0	12	+	g1	80% +0	7% -10	2% -13	2% -4				89%	11%		
T0	13	+	g1	96% +0	2% -3						79%	21%		
T0	15	+	g1	92% +0	4% -3						68%	32%		
T0	16	+	g1	77% +0	8% -10	2% -13	2% -4	1% -1			91%	9%		
T0	17	+	g1	95% +0	3% -3						89%	11%		
T0	18	+	g1	90% +0	5% -2						91%	9%		
T0	25	+	g2	57% +0	18% -23	22% -3					100%			
T0	26	+	g2	78% +0	21% -3						90%	10%		
T0	27	+	g1	78% +0	21% -3						100%			
T0	28	+	g1	45% -3	40% +0	13% -2					100%			
T0	29	+	g1	69% +0	30% -3						100%			
T0	30	+	g1	78% +0	21% -3						100%			
T0	31	+	g1	65% +0	17% -3	16% -5					100%			

T0	32	+	g1	59% +0	21% -3	18% +1					92%	8%		
T0	33	+	g1	60% +0	19% -3	18% -5					88%	12%		
T0	34	+	g1	65% +0	33% -3						-	-		
T0	35	+	g1	60% +0	19% -2	19% -3					88%	12%		
T0	36	+	g1	64% +0	19% -3	15% -1					100%			
T0	37	+	g1	64% +0	19% -3	15% -1					79%	21%		
T0	38	+	g1	63% +0	20% -3	15% -1					100%			
T0	39	+	g1	60% +0	19% -2	19% -3					100%			
T0	40	+	g1	63% +0	20% -3	15% -1					85%	15%		
T0	41	+	g1	67% +0	27% -3	4% +1					100%			
T0	42	+	g1	61% +0	21% -3	16% -1					88%	12%		
T0	43	+	g1	61% +0	21% -3	16% -1					100%			
T0	44	+	g1	60% +0	19% -3	18% -5					83%	17%		
T0	45	+	g1	73% +0	26% -3						100%			
T0	46	+	g1	61% -3	19% +0	16% -1					100%			
T0	47	+	g1	70% +0	29% -3						91%	9%		
T0	48	+	g1	69% +0	30% -3						100%			
T0	49	+	g1	72% +0	27% -3						91%	9%		
T0	50	+	g1	40% -4	20% +0	19% -3	18% -10				87%	13%		
T0	51	+	g1	38% +0	20% -3	18% -4	21% -10				100%			
T0	52	+	g1	50% -3	24% +0	23% +1					100%			
T0	53	+	g1	69% +0	28% -3	1% -2					88%	12%		
T0	54	+	g2	52% +1	24% -1	21% -2					100%			
T0	55	+	g1	76% +0	23% -3						100%			
T0	56	+	g1	56% -3	25% +0	16% +1					100%			
T0	57	+	g1	79% +0	20% -3						83%	17%		
T0	58	+	g1	78% +0	21% -3						74%	26%		
T0	59	+	g1	85% +0	14% -3						100%			
T0	60	+	g2	71% +1	27% +0						100%			
T0	61	+	g2	65% +1	23% -6	9% +2					100%			

T0	62	+	g2	47% +0	16% +1	13% -1	12% -3				100%			
T0	63	+	g1	100% +0							-	-		
T0	64	+	g2	76% +1	20% -6	2% +0					100%			
T0	**65**	+	g2	**79% +1**	**19%-6**						85%	15%		
T0	89	+	g1	87% +0	7% -3						100%			
T0	90	+	g1	93% +0	4% -3						100%			
T0	93	+	g2	75% +1	22% -2						92%	8%		
T0	94	-	g1	94% +0	3% -3									
T0	95	-	g1	96% +0	2% -3									
T0	96	+	g1	97% +0	1% -3									
T0	97	+	g1	94% +0	3% -3									
T0	98	+	g1	91% +0	3% +1	2% -3								
T0	99	+	g1	94% +0	3% -3									
T0	100	+	g1	92% +0	2% -1	1% -3								
T0	101	+	g1	92% +0	4% -3									
T0	102	-	g1	96% +0	2% -3									
T0	103	-	g1	92% +0	3% -1	1% -3								
T1	*ofp13+1*_4n_1	+	g2	100% +1							100%			
T1	*ofp13+1*_4n_2	+	g2	100% +1							100%			
4n x 2n	*ofp13+1*_3n_1	+	g2	66% +1									87%	13%
4n x 2n	*ofp13+1*_3n_2	+	g2	66% +1									89%	11%
3n x 2n	*ofp13+1*_het_1	+	g2	48% +0	48% +1					44%	56%			
3n x 2n	*ofp13+1*_het_2	-	g2	48% +0	48% +1					70%	30%			
3n x 2n	*ofp13+1*_het_3	-	g2	48% +0	48% +1					85%	15%			

File S5. Crossings performed from T0 plants to final edited homozygous plants. Self pollination is represented by a circle with a cross inside. Ploidy is marked in red.

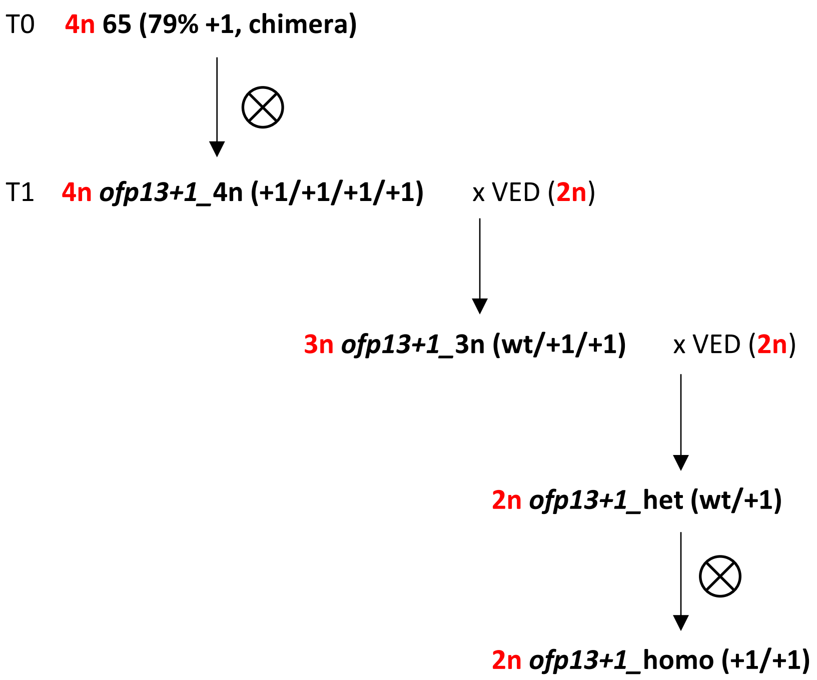

File S6. Fruit phenotype from the polyploid edited lines. (A) Fruit images of wild type VED (diploid), edited triploid (ofp13+1_3n) and edited tetraploid (ofp13+1_4n). (B) Boxplot comparing Fruit Shape Index from VED and edited triploid plants.

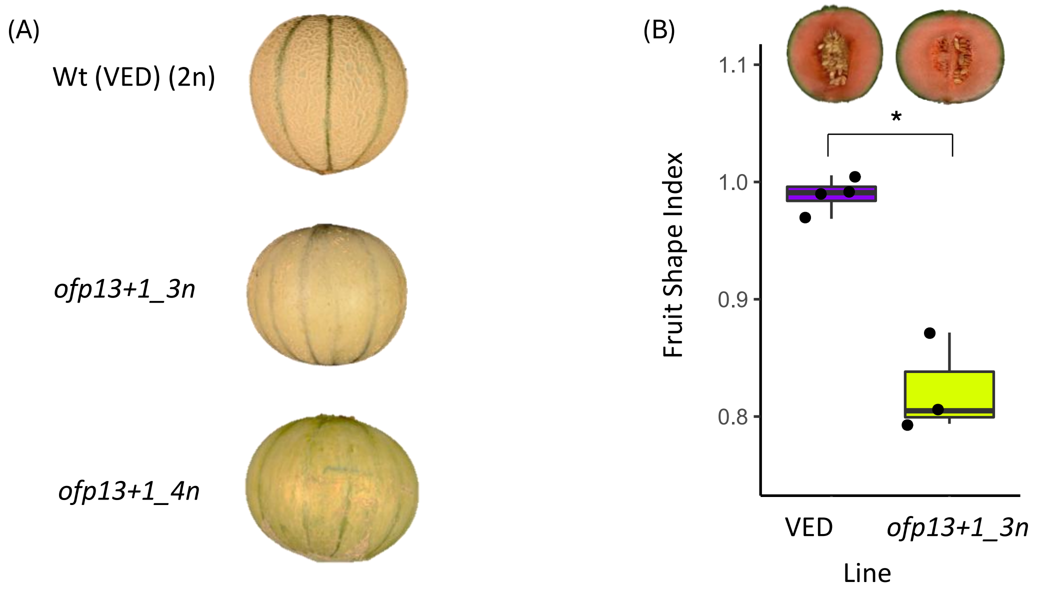

File S7. MEME motif analysis

****
MAST - Motif Alignment and Search Tool
****
MAST version 5.5.8 (Release date: Thu May 15 15:01:46 2025 -0700)

For further information on how to interpret these results please access https://meme-suite.org/meme.
To get a copy of the MAST software please access https://meme-suite.org.
****

****
REFERENCE
****
If you use this program in your research, please cite:

Timothy L. Bailey and Michael Gribskov,
"Combining evidence using p-values: application to sequence homology searches", Bioinformatics, 14(48-54), 1998.
****

****
DATABASE AND MOTIFS
****
DATABASE sequences.fa (peptide)
Last updated on Sat Aug 30 07:24:16 2025
Database contains 85 sequences, 21723 residues

MOTIFS meme.xml (peptide)
MOTIF ID ALT ID WIDTH BEST POSSIBLE MATCH
----- -- ------ ----- -------------------
1 YLSLNSKEYHGIIIKAFVDJW MEME-1 21 YLQLNGKEYHGYIVGAFVQIW
2 GESVAVVKSSDDPYEDFRRSMVEMIEENEIK MEME-2 31
GESVAVVKYSDDPYEDFRRSMVEMIVENGIY
3 MGNHKFRFSDMMPNAWFYKLKDMSKSSRPKS MEME-3 31
MGNYKFRFSDMMPNAWFYKLKDMGKAKRHKN
4 KPSFSPMLCRLPRCGNLRTLSIRDENNHNIFNSQRFYNNVDDDMVDEVIE MEME-4 50
KPSFSPMLCRLPRCGNLRTLSIRDENNHNIFNSQRFYNNVDDDMVDEVIE

PAIRWISE MOTIF CORRELATIONS:
MOTIF 1 2 3
----- ----- ----- -----
2 0.29
3 0.15 0.13
4 0.22 0.16 0.13
No overly similar pairs (correlation > 0.60) found.

Random model letter frequencies (from non-redundant database):
A 0.073 C 0.018 D 0.052 E 0.062 F 0.040 G 0.069 H 0.022 I 0.056 K 0.058
L 0.092 M 0.023 N 0.046 P 0.051 Q 0.041 R 0.052 S 0.074 T 0.059 V 0.064

W 0.013 Y 0.033
****

****
SECTION I: HIGH-SCORING SEQUENCES
****
- Each of the following 83 sequences has E-value less than 10.
- The E-value of a sequence is the expected number of sequences
  in a random database of the same size that would match the motifs as
  well as the sequence does and is equal to the combined p-value of the
  sequence times the number of sequences in the database.
- The combined p-value of a sequence measures the strength of the
  match of the sequence to all the motifs and is calculated by
    o finding the score of the single best match of each motif
      to the sequence (best matches may overlap),
    o calculating the sequence p-value of each score,
    o forming the product of the p-values,
    o taking the p-value of the product.
- The sequence p-value of a score is defined as the
  probability of a random sequence of the same length containing
  some match with as good or better a score.
- The score for the match of a position in a sequence to a motif
  is computed by by summing the appropriate entry from each column of
  the position-dependent scoring matrix that represents the motif.
- Sequences shorter than one or more of the motifs are skipped.
- The table is sorted by increasing E-value.
****

SEQUENCE NAME	DESCRIPTION	E-VALUE	LENGTH
SlOFP23		1.5e-75	329
SlOFP27		3e-75	329
SlOFP28		4.9e-72	233
SlOFP26		1.6e-71	269
SlOFP14		8e-65	351
SlOFP20		4.6e-64	321
StOFP20		7.2e-64	351
PpOFP1		5.6e-63	441
CsOFP1a		6.5e-63	327
CmOFP13		6.8e-63	331
CsOFP1b		1.5e-62	325
SlOFP22		2.8e-62	252
CmOFP4		4.9e-62	345
AtOFP2		3e-58	320
SlOFP17		1.1e-51	251
AtOFP3		1e-50	296
AtOFP1		8.9e-49	270
SlOFP2		4.4e-39	182
SlOFP21		1.3e-38	178
CmOFP19		4.4e-38	167
CsOFP6-19b		4.4e-38	167
CmOFP12		5e-38	149

CsOFP6-19a	5.7e-38	156
CaOFP20	2.3e-35	332
AtOFP6	4.4e-35	159
SlOFP30	2.8e-34	137
CsOFP6-19c	3.8e-34	171
CmOFP2	5.6e-33	468
CmOFP15	8.4e-33	170
CsOFP5a	1.5e-32	468
SlOFP29	4.3e-32	244
AtOFP4	5.8e-32	305
CsOFP13d	9.7e-32	290
CsOFP8b	1.1e-31	265
CmOFP8	1.6e-31	301
SlOFP6	1.8e-31	391
CsOFP13c	2.2e-31	227
CmOPF18	3.3e-31	269
CmOFP9	5.8e-31	227
CsOPF13a	8.8e-31	234
AtOFP8	1.2e-30	221
CsOVATE	1.5e-30	301
CmOFP5	1.5e-30	235
CmOFP21	1.9e-30	289
CmOFP14	3e-30	241
CsOFP10	3.1e-30	270
SlOFP9	4.6e-30	266
SlOFP3	4.7e-30	298
AtOFP7	7.1e-30	315
AtOFP5	1.2e-29	349
SlOFP19	4.6e-29	211
SlOFP5	1e-28	377
AtOFP13	1.3e-27	260
CmOFP3	1.4e-27	176
SlOFP10	2.4e-27	229
CmOFP6	3.2e-27	284
CsOFP12-16c	6e-27	197
CsOFP13b	1.2e-26	277
CsOFP8a	1.2e-26	239
CmOFP20	1.9e-26	237
CsOFP5b	3e-26	168
CmOFP7	3.1e-25	263
CsOFP12-16a	9.3e-25	278
CmOFP13+1	3e-24	154
CsOFP12-16b	1.8e-23	205
AtOFP10	3.2e-23	196
CmOFP1	3.7e-23	205
SlOFP15	5.3e-23	168
AtOFP12	6e-23	226
AtOFP14	8.3e-23	294
SlOFP7	1.9e-21	275
AtOFP15	2e-21	261
AtOPF16	2.5e-21	244
AtOFP11	4.1e-21	182
CsOFP14	7.2e-21	272

CmOFP10	3.9e-20	298
AtOFP18	1.5e-18	282
SlOFP12	1.9e-16	158
SlOFP11	1.5e-09	83
SlOFP13	2.5e-06	188
SlOFP8	0.00013	229
CmOFP16	0.00068	189
AtOFP17	0.023	195

****

****
SECTION II: MOTIF DIAGRAMS
****

- The ordering and spacing of all non-overlapping motif occurrences are shown for each high-scoring sequence listed in Section I.
- A motif occurrence is defined as a position in the sequence whose match to the motif has POSITION p-value less than 0.0001.
- The POSITION p-value of a match is the probability of a single random subsequence of the length of the motif scoring at least as well as the observed match.
- For each sequence, all motif occurrences are shown unless there are overlaps. In that case, a motif occurrence is shown only if its p-value is less than the product of the p-values of the other (lower-numbered) motif occurrences that it overlaps.
- The table also shows the E-value of each sequence.
- Spacers and motif occurences are indicated by
  - o -d- `d' residues separate the end of the preceding motif occurrence and the start of the following motif occurrence
  - o [n] occurrence of motif `n' with p-value less than 0.0001.

****

SEQUENCE NAME	E-VALUE	MOTIF DIAGRAM
-------------	--------	-------------
SlOFP23	1.5e-75	11-[4]-45-[2]-81-[2]-13-[1]-46
SlOFP27	3e-75	11-[4]-45-[2]-81-[2]-13-[1]-46
SlOFP28	4.9e-72	11-[4]-74-[2]-15-[1]-31
SlOFP26	1.6e-71	11-[4]-95-[2]-15-[1]-46
SlOFP14	8e-65	[3]-249-[2]-11-[1]-8
SlOFP20	4.6e-64	[3]-218-[2]-11-[1]-9
StOFP20	7.2e-64	[3]-249-[2]-11-[1]-8
PpOFP1	5.6e-63	[3]-338-[2]-11-[1]-9
CsOFP1a	6.5e-63	[3]-139-[1]-62-[2]-11-[1]-11
CmOFP13	6.8e-63	[3]-143-[1]-62-[2]-11-[1]-11
CsOFP1b	1.5e-62	[3]-220-[2]-11-[1]-11
SlOFP22	2.8e-62	11-[4]-89-[2]-13-[1]-37
CmOFP4	4.9e-62	18-[3]-222-[2]-11-[1]-11
AtOFP2	3e-58	[3]-219-[2]-11-[1]-7
SlOFP17	1.1e-51	[3]-151-[2]-11-[1]-6
AtOFP3	1e-50	4-[3]-187-[2]-11-[1]-11
AtOFP1	8.9e-49	1-[3]-164-[2]-11-[1]-11

SlOFP2	4.4e-39	92-[2]-11-[1]-27
SlOFP21	1.3e-38	97-[2]-11-[1]-18
CmOFP19	4.4e-38	81-[2]-11-[1]-23
CsOFP6-19b	4.4e-38	81-[2]-11-[1]-23
CmOFP12	5e-38	73-[2]-11-[1]-13
CsOFP6-19a	5.7e-38	80-[2]-11-[1]-13
CaOFP20	2.3e-35	263-[2]-11-[1]-6
AtOFP6	4.4e-35	64-[2]-11-[1]-32
SlOFP30	2.8e-34	65-[2]-11-[1]-9
CsOFP6-19c	3.8e-34	82-[2]-11-[1]-26
CmOFP2	5.6e-33	382-[2]-11-[1]-23
CmOFP15	8.4e-33	81-[2]-11-[1]-26
CsOFP5a	1.5e-32	382-[2]-11-[1]-23
SlOFP29	4.3e-32	1-[4]-101-[2]-11-[1]-29
AtOFP4	5.8e-32	231-[2]-11-[1]-11
CsOFP13d	9.7e-32	121-[2]-11-[1]-106
CsOFP8b	1.1e-31	193-[2]-11-[1]-9
CmOFP8	1.6e-31	231-[2]-11-[1]-7
SlOFP6	1.8e-31	319-[2]-11-[1]-9
CsOFP13c	2.2e-31	132-[2]-11-[1]-32
CmOPF18	3.3e-31	190-[2]-11-[1]-16
CmOFP9	5.8e-31	132-[2]-11-[1]-32
CsOPF13a	8.8e-31	25-[4]-42-[2]-11-[1]-54
AtOFP8	1.2e-30	152-[2]-11-[1]-6
CsOVATE	1.5e-30	232-[2]-11-[1]-6
CmOFP5	1.5e-30	25-[4]-42-[2]-11-[1]-55
CmOFP21	1.9e-30	123-[2]-11-[1]-103
CmOFP14	3e-30	169-[2]-11-[1]-9
CsOFP10	3.1e-30	191-[2]-11-[1]-16
SlOFP9	4.6e-30	184-[2]-11-[1]-19
SlOFP3	4.7e-30	16-[4]-73-[2]-11-[1]-96
AtOFP7	7.1e-30	224-[2]-11-[1]-28
AtOFP5	1.2e-29	280-[2]-11-[1]-6
SlOFP19	4.6e-29	125-[2]-15-[1]-19
SlOFP5	1e-28	90-[3]-173-[2]-11-[1]-20
AtOFP13	1.3e-27	11-[4]-83-[2]-13-[1]-51
CmOFP3	1.4e-27	101-[2]-11-[1]-12
SlOFP10	2.4e-27	114-[2]-11-[1]-52
CmOFP6	3.2e-27	146-[2]-11-[1]-75
CsOFP12-16c	6e-27	105-[2]-15-[1]-25
CsOFP13b	1.2e-26	134-[2]-11-[1]-80
CsOFP8a	1.2e-26	170-[2]-11-[1]-6
CmOFP20	1.9e-26	168-[2]-11-[1]-6
CsOFP5b	3e-26	87-[2]-11-[1]-18
CmOFP7	3.1e-25	148-[2]-23-[1]-40
CsOFP12-16a	9.3e-25	153-[2]-24-[1]-49
CmOFP13+1	3e-24	[3]-123
CsOFP12-16b	1.8e-23	111-[2]-16-[1]-26
AtOFP10	3.2e-23	94-[2]-11-[1]-39
CmOFP1	3.7e-23	111-[2]-16-[1]-26
SlOFP15	5.3e-23	91-[2]-11-[1]-14
AtOFP12	6e-23	148-[2]-15-[1]-11
AtOFP14	8.3e-23	189-[2]-16-[1]-37

SlOFP7 1.9e-21 48-[4]-81-[2]-15-[1]-29
AtOFP15 2e-21 106-[2]-12-[1]-91
AtOPF16 2.5e-21 161-[2]-20-[1]-11
AtOFP11 4.1e-21 99-[2]-16-[1]-15
CsOFP14 7.2e-21 178-[2]-15-[1]-27
CmOFP10 3.9e-20 204-[2]-15-[1]-27
AtOFP18 1.5e-18 133-[2]-12-[1]-85
SlOFP12 1.9e-16 57-[2]-16-[1]-33
SlOFP11 1.5e-09 31-[2]-21
SlOFP13 2.5e-06 104-[2]-12-[1]-20
SlOFP8 0.00013 184-[1]-24
CmOFP16 0.00068 107-[2]-12-[1]-18
AtOFP17 0.023 167-[1]-7

****

****
SECTION III: ANNOTATED SEQUENCES
****
- The positions and p-values of the non-overlapping motif occurrences are shown above the actual sequence for each of the high-scoring sequences from Section I.
- A motif occurrence is defined as a position in the sequence whose match to the motif has POSITION p-value less than 0.0001 as defined in Section II.
- For each sequence, the first line specifies the name of the sequence.
- The second (and possibly more) lines give a description of the sequence.
- Following the description line(s) is a line giving the length, combined p-value, and E-value of the sequence as defined in Section I.
- The next line reproduces the motif diagram from Section II.
- The entire sequence is printed on the following lines.
- Motif occurrences are indicated directly above their positions in the sequence on lines showing
  o the motif number of the occurrence,
  o the position p-value of the occurrence,
  o the best possible match to the motif, and
  o columns whose match to the motif has a positive score (indicated by a plus sign).
****

SlOFP23
LENGTH = 329 COMBINED P-VALUE = 1.76e-77 E-VALUE = 1.5e-75
DIAGRAM: 11-[4]-45-[2]-81-[2]-13-[1]-46

[4]
2.1e-62
KPSFSPMLCRLPRCGNLRTLSIRDENNHNIFNSQRFYNNVDDDMVDEVIE
++++++++++++++ ++++++++++++++++++++++++++++++++++

1
MKFSSLFKSNKKPSFSPMLCRLPRCGDLRTLSIRDENNHNIFNSQRFYNNVDDEMVDEVIESLKLEKDRF
FVEAG

[2]
4.1e-05
GESVAVVKYSDDPYEDFRRSMVEMIVENGIY
+ +++ +++++ ++ ++ ++ ++ +
76
QKTSLILDMSSSRLSKKRTISKRLEFLPFNNDSCVITSMDSIDAYGETSRSILEGSSSRLSKSTNNSTSSKRL
GY

[2]
6.3e-15
GESVAVV
+++ +++
151
LPSNDSMDSYGDQETSSILDMSSSSSNDNISSKGLGYLPSNESMDATSILERSKSNSSHGFVYYVPCKKT
YAIMR

[1]
4.6e-15
KYSDDPYEDFRRSMVEMIVENGIY YLQLNGKEYHGYIVGAFVQIW
+ + ++++++++ + ++++++ + + ++ ++ ++++++++++++
226
LISRDPYEDIKYFLEKMVDENLEIEDWEESLEELCGWLLEINEKNIHKYIVGAFCDLWMSYSCTSTINTPFG
FSS

SlOFP27
LENGTH = 329 COMBINED P-VALUE = 3.55e-77 E-VALUE = 3e-75
DIAGRAM: 11-[4]-45-[2]-81-[2]-13-[1]-46

[4]
1.8e-61
KPSFSPMLCRLPRCGNLRTLSIRDENNHNIFNSQRFYNNVDDDMVDEVIE
+++++++++++ +++++++++++++++++++++++++++++++++++++++
1
MKFSSLFKSKKKPPFSPMLCRLSRCGNLRTLSIRDENNHNIFNSQRFYNNVDDEMVDEVIENLKLEKDRF
FVESG

[2]
5.3e-05
GESVAVVKYSDDPYEDFRRSMVEMIVENGIY
+ +++ +++++ ++ ++ ++ ++ +
76
QKTSSLLDMSSSRLSKRRTISKRLEFLPFNNDSYVITLMDSIDAYGETSRSILEGSSSRLSKSTNNSTSSKRL
SY

[2]
1.4e-15
GESVAVV
+++++++

151
RPSNDSMDSYGDQETSSILDMSSLSSNDSISSNGLGYLPSNESMDATSILERSKSNSSHGFVYYVPCKKT
YVIMR

[1]
4.6e-15
KYSDDPYEDFRRSMVEMIVENGIY YLQLNGKEYHGYIVGAFVQIW
+ + ++++++++ + ++++++ + + ++ ++ ++++++++++++
226
LISRDPYEDIKYFLERMVDENLEIEDWKESLEELCGWLLEINEKNIHKYIVGAFCDLWMSYSCTSTTNTPFE
FNS

SlOFP28
LENGTH = 233 COMBINED P-VALUE = 5.72e-74 E-VALUE = 4.9e-72
DIAGRAM: 11-[4]-74-[2]-15-[1]-31

[4]
1.8e-63
KPSFSPMLCRLPRCGNLRTLSIRDENNHNIFNSQRFYNNVDDDMVDEVIE
++
1
MKFFSLFKSKKKPSFSPMLCRLPRCGNLRTLSIRDENNHNIFNSQRFCINVDDDIVDEVIEGLKFEKKRFY
FEAG

[2]
5.8e-16
GESVAVVKYSDDPYE
+ ++++ ++++++
76
EKTSSILDVSCAITHIDSIDAYGKTSTRSILKGTKSRLSKNDNSTSNDMVESLPLNDSCVITPSVMRVTSIDPY
G

[1]
8.7e-09
DFRRSMVEMIVENGIY YLQLNGKEYHGYIVGAFVQIW
+++ ++ ++++++++ + +++ ++++++++++++
151
YIKKHMEMMVEENQGIKDWKESLKEICALYLEINYIDKNIHRFIIGAFCDLWMSYSGTSTTNTPFGFSTSEP
PSP

SlOFP26
LENGTH = 269 COMBINED P-VALUE = 1.92e-73 E-VALUE = 1.6e-71
DIAGRAM: 11-[4]-95-[2]-15-[1]-46

[4]
1.8e-63
KPSFSPMLCRLPRCGNLRTLSIRDENNHNIFNSQRFYNNVDDDMVDEVIE
++
1
MEFFSLFKSKKKPSFSPMLCRLPRCGNLRTLSIRDENNHNIFNSQRFCINVDDDIVDEVIEGLKFEKKRFF
FESG

[2] [1]
2.3e-14 8.2e-10
GESVAVVKYSDDPYEDFRRSMVEMIVENGIY YLQLNGKEYHGYIVGAFVQIW
++++ ++++++ +++ ++ +++++++ + ++++ ++++++++++++
151
LNNSCVISPSAMRVTSIDPYGYIKKYMEITVEENQGIKDWKESLKEICAWYLENNDNDKNIHKFIIGAFCDL
WMS

SlOFP14
LENGTH = 351 COMBINED P-VALUE = 9.47e-67 E-VALUE = 8e-65
DIAGRAM: [3]-249-[2]-11-[1]-8

[3]
6.9e-35
MGNYKFRFSDMMPNAWFYKLKDMGKAKRHKN
+++++++++++++++++++++++++++++++
1
MGNHKFKFSDMMPNTWFYKLKDMSKTKNHKSPFSSSSTNKSQYSQPRSSFSYTRRSIRVDKIYNSHSYN
FLDQPR

[2]
7.5e-27
GESVAVVKYSDDPYEDFRRS
+++++++++++++ ++++++
226
EPKNRVGSPVSRKHYSSSSGVKLRTNSTKVANKRNSVSSSKRRSKTKKESCSASRGTSFAIVKASIDPEKD
FRES

[1]
8.2e-20
MVEMIVENGIY YLQLNGKEYHGYIVGAFVQIW
+++++++++++ ++++++ ++++++++++ +++
301 MVEMVVENNIRASKELENLLACYLSLNSNEYHDLIIKAFEQIWFDLSDLHL

SlOFP20
LENGTH = 321 COMBINED P-VALUE = 5.40e-66 E-VALUE = 4.6e-64
DIAGRAM: [3]-218-[2]-11-[1]-9

[3]
2.1e-34
MGNYKFRFSDMMPNAWFYKLKDMGKAKRHKN
++++++++++++++++++++++++++++ +++
1
MGNYRFRLSDMMPNAWFYKLKDMAKSSSRRHSHTTSSSNLQLDKKRQPHNNLGCQRKSYYISRNLTIT
SPISSNS

[2] [1]
4.9e-25 4.9e-21
GESVAVVKYSDDPYEDFRRSMVEMIVENGIY YLQLNGKEY
++++++++++ +++++++++++++ ++++ +++++++++

226
NSPRITTTTTNSRKSVSSKRTSVTTDSFAVVKSSRNPQKDFRESMVEMIIENNITTSKDLEELLACYLSLNSD
EY

HGYIVGAFVQIW
++++++++++++
301 HDIIIKVFKQIWFEITEIRLK

StOFP20
LENGTH = 351 COMBINED P-VALUE = 8.42e-66 E-VALUE = 7.2e-64
DIAGRAM: [3]-249-[2]-11-[1]-8

[3]
6.9e-35
MGNYKFRFSDMMPNAWFYKLKDMGKAKRHKN
+++++++++++++++++++++++++++++++
1
MGNHKFKFSDMMPNTWFYKLKDMSKTKNHKSPFSSSSTNKSQYSQPRSSFSYTRRSIRVDKIYNSHSYS
FLDQPR

[2]
8.3e-26
GESVAVVKYSDDPYEDFRRS
+++++++++++++ ++++++
226
EAKNRVSSPVSRKHYSSSSGVKLRTNSTKVASKRNSVSSSKRRSKAKKESCSTSTGTSFAIVKASIDPEKDF
RES

[1]
8.2e-20
MVEMIVENGIY YLQLNGKEYHGYIVGAFVQIW
+++++++++++ ++++++ ++++++++++ +++
301 MIEMVVENNIRASKDLENLLACYLSLNSNEYHDLIIKAFEQIWFNLSDLHL

PpOFP1
LENGTH = 441 COMBINED P-VALUE = 6.58e-65 E-VALUE = 5.6e-63
DIAGRAM: [3]-338-[2]-11-[1]-9

[3]
3.9e-33
MGNYKFRFSDMMPNAWFYKLKDMGKAKRHKN
+++++++++++++++++++++++++++++++
1
MGNHKFRLSDMMPNAWFHKLKDMSKPRKNPNSPHPSKKKKQQQKPTFASTAKFTEPSKPKQQLPHQC
LPRQSYYF

[2]
2.2e-2
GESVAV

+++++
301
KVAKEESTSTKTIKEQRTASSVRRVSSNATSPGVRLRMNSPRIANRKINQANLSRRSVSSNSSSKRRSLSES
FAI

[1]
7 1.6e-19
VKYSDDPYEDFRRSMVEMIVENGIY YLQLNGKEYHGYIVGAFVQIW
++++++++++++++++++++++++ ++++++++++++++++++++
376 VKSSFDPQRDFRESMVEMIMENNIKASKDLEDLLACYLSLNSDEYHELIIKVFKQIWFDLTDLRSK

CsOFP1a
LENGTH = 327 COMBINED P-VALUE = 7.70e-65 E-VALUE = 6.5e-63
DIAGRAM: [3]-139-[1]-62-[2]-11-[1]-11

[3]
4.9e-33
MGNYKFRFSDMMPNAWFYKLKDMGKAKRHKN
++++++++++++++++++++++++++++++
1
MRNHKFRFSDMIPNAWFYKLKEIGGASRPKSFRSNKNPHAHPPPPPPPSKHKQQPPPPPPPPHSRSRK
SYYFTRQ

[1]
6.7e-06
YLQLNGKEYHGYIVGAFVQIW
+ + + +++++ ++
151
DFRTDKILTAEASEHFEHDIVIDVSSNYSNNAVIGAFDELELPPIITKQKKKTETKQRTTTTTTTGTKKVAGNSP

[2] [1]
3.2e-26 2.6e-
GESVAVVKYSDDPYEDFRRSMVEMIVENGIY YLQLN
+++++++++++++++++++++++++++++ +++++
226
GVRLRIHSPKIGYRKMGGRKSVSSRRSLSESLAIMKSSYDPQKDFRESMVEMIVENNIRSSKELEDLLACY
LCLN

20
GKEYHGYIVGAFVQIW
++++++++++++++++
301 ADEYHDLIIKVFKQIWFDLTQPSPPPL

CmOFP13
LENGTH = 331 COMBINED P-VALUE = 7.95e-65 E-VALUE = 6.8e-63
DIAGRAM: [3]-143-[1]-62-[2]-11-[1]-11

[3]
4.9e-33
MGNYKFRFSDMMPNAWFYKLKDMGKAKRHKN

++++++++++++++++++++++++++++++
1
MRNHKFRFSDMIPNAWFYKLKEIGGASRPKSFRSNKNPHHPPPPPPPSKHKQPPPPPPHSRSRKSYYFT
RQLESN

[1]
6.7e-06
YLQLNGKEYHGYIVGAFVQIW
+ + + +++++ ++
151
DTSPDFRTDKILTAEASKHFEHDIVIDVSSNYSNNAVIGAFDELELPPIITKQRKKTETKQRTTTTTTAGTKKVA

[2] [
3.2e-26 2
GESVAVVKYSDDPYEDFRRSMVEMIVENGIY Y
+++++++++++++++++++++++++++++ +
226
GNSPGVRLRIHSPKIGYRKMGGRKSVSSRRSLSESLAIMKSSYDPQKDFRESMVEMIVENNIRGSKELED
LLACY

1]
.6e-20
LQLNGKEYHGYIVGAFVQIW
++++++++++++++++++++
301 LCLNADEYHDLIIKVFKQIWFDLTQPSPPPL

CsOFP1b
LENGTH = 325 COMBINED P-VALUE = 1.77e-64 E-VALUE = 1.5e-62
DIAGRAM: [3]-220-[2]-11-[1]-11

[3]
1.2e-34
MGNYKFRFSDMMPNAWFYKLKDMGKAKRHKN
+++++++++++++++++++++++++++++++
1
MGNYRFRVSDMMPNSWFYKLKDMTTIIRRRNSKKDQSSKNSHTTDLVYSHPRKSIHFTPSQLAANNSPL
EPPRRS

[2] [1]
2.0e-24 4.3e-20
GESVAVVKYSDDPYEDFRRSMVEMIVENGIY YLQLNGK
+++++++++ +++++++++++++++++++ +++++ +
226
IVNSPRVSSSKRFSHVSRRRSGKRSLNDSLAIVKSTKDPQRDFRESMVEMIVENKISGSNELEDLLACYLS
LNTD

EYHGYIVGAFVQIW
++++++++++++++
301 EYHDIIVKVFKQIWFDMTDIIGDHY

SlOFP22
LENGTH = 252 COMBINED P-VALUE = 3.30e-64 E-VALUE = 2.8e-62
DIAGRAM: 11-[4]-89-[2]-13-[1]-37

[4]
9.8e-45
KPSFSPMLCRLPRCGNLRTLSIRDENNHNIFNSQRFYNNVDDDMVDEVIE
+ ++++ ++ ++ ++ + +++++ +++++++++++ ++++++++++ +++
1
MNLSSLFKSKKKSSFSPFLCPLPHCGIPKTLSLRVENNDNIFNSQRLYNNVDDDMVDKMVEGLKIEKDRF
FFEAG

[2]   [1]
5.8e-16   1.0e-17
GESVAVVKYSDDPYEDFRRSMVEMIVENGIY   YLQLNGKEYHGYIVGAFVQIW
+ +++ +++++++ ++++++ ++++++++   ++ ++ +++++++++++++++
151
DSCIIKLSSSMDPYGSFKKSMVKMVEANLGIKDWNEFLEEMLAWYLEVNEKNNHKYIIGAFCDLWISYSF
TSSTT

CmOFP4
LENGTH = 345 COMBINED P-VALUE = 5.75e-64 E-VALUE = 4.9e-62
DIAGRAM: 18-[3]-222-[2]-11-[1]-11

[3]
1.2e-34
MGNYKFRFSDMMPNAWFYKLKDMGKAKRHKN
+++++++++++++++++++++++++++++++
1
MVGDRVEWSRERNNHFWRMGNYRFRVSDMMPNSWFYKLKDMTTIIRRRNKNKDQPSKNTHTTTDLA
YSHPRKSIH

[2]
5.2e-24
GESVAVVKYSDDPYEDFRRSMVEMIVENG
+++++++++ +++++++++++++++++++
226
PIRSSPSRRFLLNSPGPKLRIVNSPRVSSSKRFGHVGRRKSGKRSLNDSLAIVKSTEDPQRDFRESMMEM
IVENK

[1]
4.3e-20
IY   YLQLNGKEYHGYIVGAFVQIW
+   +++++ +++++++++++++
301 ISGSSELEDLLACYLSLNTDEYHDIIVKVFKQIWFDMTDIIGVHY

AtOFP2
LENGTH = 320 COMBINED P-VALUE = 3.48e-60 E-VALUE =   3e-58
DIAGRAM: [3]-219-[2]-11-[1]-7

[3]
9.6e-31
MGNYKFRFSDMMPNAWFYKLKDMGKAKRHKN
+++++++++ ++++++++++++++++++++
1
MGNYKFRISEMLPNAWFHKLKDVTKHSKPKNKASSSSSNTCSKKKPSSDSLPQHSYFSNSLVANNPPH
HNSPRNS

[2] [1]
4.9e-25 6.9e-19
GESVAVVKYSDDPYEDFRRSMVEMIVENGIY YLQLNGKE
++++++++++++ +++++++++++++++++ ++++++++
226
VNSPRIQLSGTRRSTSRRSESKQDVLESFAVMKRSVDPKKDFRESMIEMIEENNIRASKDLEDLLACYLTL
NPKE

YHGYIVGAFVQIW
++++++ ++ +++
301 YHDLIIHVFEQIWLQLTKTK

SlOFP17
LENGTH = 251 COMBINED P-VALUE = 1.29e-53 E-VALUE = 1.1e-51
DIAGRAM: [3]-151-[2]-11-[1]-6

[3]
1.0e-27
MGNYKFRFSDMMPNAWFYKLKDMGKAKRHKN
++++++++++++ +++++++++++++++++
1
MGNYRFKLSDMVTSSWFYKLKDMAKSRTQIKRKQTSSSTSSSSFSIFYSSSNVQQHHRKSYYFSRTLSPN
PHQSN

[2] [
5.2e-23 6
GESVAVVKYSDDPYEDFRRSMVEMIVENGIY Y
+ +++++++++++++++++++++++ ++++ +
151
TSPKRRISVSSSSTGVKLRTKSPRIISRRSVGEKSYAVVKSSKNPQKDFKESMVEMIVKNNIKTSKDLEELLA
CY

1]
.2e-17
LQLNGKEYHGYIVGAFVQIW
+ +++++++ +++ ++++++
226 LLLNSHHYHHLIITVFKQIWFDLQLK

AtOFP3
LENGTH = 296 COMBINED P-VALUE = 1.22e-52 E-VALUE = 1e-50
DIAGRAM: 4-[3]-187-[2]-11-[1]-11

[3]
1.5e-27
MGNYKFRFSDMMPNAWFYKLKDMGKAKRHKN
++ ++++++++++ ++ ++++ +++++++++
1
MKQKMGTHKFRFSDMMPHSWLYKLKGMSRSSRKHQLSSPKHLSSADASSSRKLRDPLRRLSSTAHHP
QASNSPPK

[2]
1.1
GES
+ +
151
ELSVRKLDDVPEDPSVSPNLSPETAKEPPFEMMTQQKLKKPKAHSSGIKIPTKIVRKKKKERTSQVSKKKGV
VKS

[1]
e-23 9.4e-16
VAVVKYSDDPYEDFRRSMVEMIVENGIY YLQLNGKEYHGYIVGAFVQIW
++++++++++ +++++++++++++++++ ++++++ ++++++++++ +
226
FAIVLSSVDPEKDFRESMVEMIMENKMREQKDLEDLLACYLSLNSSEYHDVIIKAFENTWLHLTQGLSISL

AtOFP1
LENGTH = 270 COMBINED P-VALUE = 1.04e-50 E-VALUE = 8.9e-49
DIAGRAM: 1-[3]-164-[2]-11-[1]-11

[3]
1.9e-24
MGNYKFRFSDMMPNAWFYKLKDMGKAKRHKN
+++++++ +++++++++ +++++++++
1
MGNNYRFKLSELIPNAWFYKLRDMSKSKKKNLQSQPNSTTSKKKHHAVSTPTSTTPLSPRPPRRPSHSSK
APPSH

[2]
1.6e-21
GESVAVVKYSDDPYEDFRRSMVEMIVENG
+ +++++++++ +++++++++++ +++
151
ELRPIITKTAATARKTAVNSPAGVRLRMRSPRISVSSSARRSGSSARRSRAVVKASVDPKRDFKESMEEMIA
ENK

[1]
6.9e-19
IY YLQLNGKEYHGYIVGAFVQIW
++ ++++++++++ +++ ++++++
226 IRATKDLEELLACYLCLNSDEYHAIIINVFKQIWLDLNLPPPHSK

SlOFP2

LENGTH = 182 COMBINED P-VALUE = 5.23e-41 E-VALUE = 4.4e-39
DIAGRAM: 92-[2]-11-[1]-27

```
            [2]                         [1]
            7.3e-30                         1.0e-20
            GESVAVVKYSDDPYEDFRRSMVEMIVENGIY       YLQLNGKEYHGYIVGA
            +++++++++++++++++++++++++++++++       ++++++++++++++++
76
SSDFKTSKAVQGFGRIGGESVAVEKDSDDPYLDFRQSMLQMILEKEIYSKDDLKELLNCFLQLNSPYYHGI
IVRA
```

```
   FVQIW
   +++++
151  FTEIWNGVFSLRPGVAGASSPFLHGGSHVTYR
```

SlOFP21
LENGTH = 178 COMBINED P-VALUE = 1.55e-40 E-VALUE = 1.3e-38
DIAGRAM: 97-[2]-11-[1]-18

```
               [2]                         [1]
               7.3e-30                         1.0e-20
               GESVAVVKYSDDPYEDFRRSMVEMIVENGIY       YLQLNGKEYHG
               +++++++++++++++++++++++++++++++       +++++++++++
76
TPSPAHYSSDAERAVQGFGRIGGESVAVEKDSDDPYVDFRQSMLQMILEKEIYSKDELRELLNCFLQLNS
PYYHG
```

```
   YIVGAFVQIW
   ++++++++++
151  IIVRAFTEIWHCVFSVNPGVTGAESPFL
```

CmOFP19
LENGTH = 167 COMBINED P-VALUE = 5.18e-40 E-VALUE = 4.4e-38
DIAGRAM: 81-[2]-11-[1]-23

```
      [2]                         [1]
      2.7e-29                         3.9e-19
      GESVAVVKYSDDPYEDFRRSMVEMIVENGIY       YLQLNGKEYHGYIVGAFVQIW
      +++++++++++++++++++++++++++++++       +++++++++++++++++++++
76
GFWKIGGVSVAVEKDSNDPYVDFRQSMLQMILENEIYTQEGLRELLSCFLHLNSPCNHGIIIRAFAEIWD
GVFCA
```

CsOFP6-19b
LENGTH = 167 COMBINED P-VALUE = 5.18e-40 E-VALUE = 4.4e-38
DIAGRAM: 81-[2]-11-[1]-23

[2] [1]
2.7e-29 3.9e-19
GESVAVVKYSDDPYEDFRRSMVEMIVENGIY YLQLNGKEYHGYIVGAFVQIW
+++++++++++++++++++++++++++++++ +++++++++++++++++++++
76
GFWKIGGVSVAVEKDSNDPYVDFRQSMLQMILENEIYTQEGLRELLSCFLHLNSPCNHGIIIRAFAEIWDS
VFCA

CmOFP12
LENGTH = 149 COMBINED P-VALUE = 5.89e-40 E-VALUE = 5e-38
DIAGRAM: 73-[2]-11-[1]-13

[2
2.
GE
++
1
MAAPRRNLQPTSLSVDLNICRPKLLSHLFHHLKPKPSLKSPNHHHRFSSASSDSESESETRTSITFRGFGR
SGGE

] [1]
5e-31 1.2e-18
SVAVVKYSDDPYEDFRRSMVEMIVENGIY YLQLNGKEYHGYIVGAFVQIW
+++++++++++++++++++++++++++++ +++++++ +++++++++++++
76
SVAVEKDSDDPYLDFRHSMVQMILENEIYSKEDLRGLLRCFLQLNSPSHHGIIVRAFSEIWDSVFSATSPIL
RF

CsOFP6-19a
LENGTH = 156 COMBINED P-VALUE = 6.76e-40 E-VALUE = 5.7e-38
DIAGRAM: 80-[2]-11-[1]-13

[2] [1]
2.5e-31 1.2e-18
GESVAVVKYSDDPYEDFRRSMVEMIVENGIY YLQLNGKEYHGYIVGAFVQIW
+++++++++++++++++++++++++++++++ +++++++ +++++++++++++
76
FGRSGGESVAVEKDSDDPYLDFRHSMVQMILENEIYSKEDLRGLLRCFLQLNSPSHHGIIVRAFSEIWDS
VFSST

CaOFP20
LENGTH = 332 COMBINED P-VALUE = 2.76e-37 E-VALUE = 2.3e-35
DIAGRAM: 263-[2]-11-[1]-6

[2]
3.8e-27
GESVAVVKYSDDPYEDFRRSMVEMIVENGIY
++++++++++ +++++++++++++++++++

226
IARRASSGVKLRTNSPRITNCRKIQASRKSVSSRRTSVTESFAVVKSSRNPQKDFRESMVEMIVENNIRASK
DLE

[1]
1.8e-20
YLQLNGKEYHGYIVGAFVQIW
+++++++++++++++++++++
301 ELLACYLSLNSDEYHDLIIKVFKQIWFDITKY

AtOFP6
LENGTH = 159 COMBINED P-VALUE = 5.14e-37 E-VALUE = 4.4e-35
DIAGRAM: 64-[2]-11-[1]-32

[2]
1.3e-27
GESVAVVKYSD
+ +++++++++
1
MATKSKKKILKTVSVVDISCGNCIKPTFASIFNFFSKKPKRPSSTYRHCHSSISSATPSSTPLATASVAVEKDS
D

[1]
2.9e-19
DPYEDFRRSMVEMIVENGIY YLQLNGKEYHGYIVGAFVQIW
++++++++++++++++++++ +++++++++++++++++++++
76
DPYLDFRQSMLQMILENQIYSKDELRELLQCFLSLNSHYHHGIIVRAFSEIWEDVSSAAASAVEASPLITRH
VSR

SlOFP30
LENGTH = 137 COMBINED P-VALUE = 3.30e-36 E-VALUE = 2.8e-34
DIAGRAM: 65-[2]-11-[1]-9

[2]
9.3e-29
GESVAVVKYS
++++++++++
1
MSSKNKKIWNCITSNGTAGCGCSKPKLSEIIQPKPKPRPEPEPNAHSSSTSNSDSPSPTIMPAKIVGSVAV
VKDS

[1]
4.4e-17
DDPYEDFRRSMVEMIVENGIY YLQLNGKEYHGYIVGAFVQIW
+++++++++++++++++++++ +++++++ +++++++++ +++
76 DDPFGDFRRSMLQMIMEKEIYSYDDLNELLNCFLQLNSPSHHDIILQAFMEIWNNGKNYIAN

CsOFP6-19c
LENGTH = 171 COMBINED P-VALUE = 4.47e-36 E-VALUE = 3.8e-34

DIAGRAM: 82-[2]-11-[1]-26

```
        [2]                            [1]
        2.2e-29                        5.0e-16
        GESVAVVKYSDDPYEDFRRSMVEMIVENGIY         YLQLNGKEYHGYIVGAFVQIW
        ++++++++++++++++++ ++++++++++++         ++++++++++++++++++++
76
SPVAILIGDSIAVEKDSDDPYEDFRGSMVEMIVEKRIYSPNGLQELLNCFLHLNSPYHHEIIVKAFTQISNEF
ES
```

CmOFP2
LENGTH = 468 COMBINED P-VALUE = 6.53e-35 E-VALUE = 5.6e-33
DIAGRAM: 382-[2]-11-[1]-23

```
        [2]                            [1]
        2.7e-25                        1.1e-18
        GESVAVVKYSDDPYEDFRRSMVEMIVENGIY         YLQLNGKEYHGYIVGAFVQIW
         +++++++++++++++++ +++++++++++          +++++++++++++++++ +++
376
MVDDETDLESFAVVKSSFDPQQDFRDSMVEMIMERRISKAEELEELLACYLTLNSDQYHDLIIKVFRQVW
FDLNQ
```

CmOFP15
LENGTH = 170 COMBINED P-VALUE = 9.94e-35 E-VALUE = 8.4e-33
DIAGRAM: 81-[2]-11-[1]-26

```
        [2]                            [1]
        1.4e-28                        1.2e-15
        GESVAVVKYSDDPYEDFRRSMVEMIVENGIY         YLQLNGKEYHGYIVGAFVQIW
         +++++++++++++++++++++++++++++          ++++++++++++++++++++
76
PVAILICDSIAVEKDSDDPYEDFRRSMVQMIVEKRIYSPNGLQELLNCFLHLNSPYHHEIILKAFTQISNEFE
SS
```

CsOFP5a
LENGTH = 468 COMBINED P-VALUE = 1.74e-34 E-VALUE = 1.5e-32
DIAGRAM: 382-[2]-11-[1]-23

```
        [2]                            [1]
        2.7e-25                        1.1e-18
        GESVAVVKYSDDPYEDFRRSMVEMIVENGIY         YLQLNGKEYHGYIVGAFVQIW
         +++++++++++++++++ +++++++++++          +++++++++++++++++ +++
376
TVEDDTDLESFAVVKSSFDPQQDFRDSMVEMIMERRISKAEELEELLACYLTLNSDQYHDLIIKVFRQVWF
DLNQ
```

SlOFP29
LENGTH = 244 COMBINED P-VALUE = 5.05e-34 E-VALUE = 4.3e-32
DIAGRAM: 1-[4]-101-[2]-11-[1]-29

[4]
8.0e-05
KPSFSPMLCRLPRCGNLRTLSIRDENNHNIFNSQRFYNNVDDDMVDEVIE
++ +++ ++ + +++
1
MGKKMNLGSWQWPSCTHSKTQSFRANHIFKTINSIFLDPSNTDHHHHGVVEIETTPESWFTNSSESASF
STESEE

[2] [1]
8.8e-24 9.2e-19
GESVAVVKYSDDPYEDFRRSMVEMIVENGIY YLQLNGKEYHGYIVGAFVQIW
+++++ +++++++++++++++++++ ++++ ++++++++++++++++++++
151
PFKESVALALESEDPYLDFKKSMEEMVDTHEIKDWESLQELLQWYLKMNGKNNHGFIIGAFVDLLIGFTP
SNCDS

AtOFP4
LENGTH = 305 COMBINED P-VALUE = 6.86e-34 E-VALUE = 5.8e-32
DIAGRAM: 231-[2]-11-[1]-11

[2] [1]
1.7e-23 1.3e-20
GESVAVVKYSDDPYEDFRRSMVEMIVENGIY YLQLNGKEYHGYIVGAFVQIW
+++++ ++++++ +++++++++++ +++++ ++++++++++++++++++++
226
SQNKQILDSFAVIKSSIDPSKDFRESMVEMIAENNIRTSNDMEDLLVCYLTLNPKEYHDLIIKVFVQVWLEVI
NS

CsOFP13d
LENGTH = 290 COMBINED P-VALUE = 1.14e-33 E-VALUE = 9.7e-32
DIAGRAM: 121-[2]-11-[1]-106

[2]
2.4e-26
GESVAVVKYSDDPYEDFRRSMVEMIVENG
+++++++++++++++++++++++++++++
76
QIEALVRGLRVRQGKRLFLELDETNSIMTTTVAVATVVGGNYQVPFKESVAMAMESKDPYLDFKKSMEEM
VEAHE

[1]
6.2e-17
IY YLQLNGKEYHGYIVGAFVQIW
++ ++++++++++++++++++++
151
LKNWKGMERLLSWYLKANGKANHEFIIGAFVDLLVDLAFSASSNFSNNSSSSPSSSSSSSTTTTSSLLCSS
TSTF

CsOFP8b

LENGTH = 265 COMBINED P-VALUE = 1.26e-33 E-VALUE = 1.1e-31
DIAGRAM: 193-[2]-11-[1]-9

```
                    [2]
                    1.7e-26
                    GESVAVVKYSDDPYEDFRRSMVEMIVENGIY
                    ++++++++++ +++ +++ ++++++++++++
151
TLFSSKSRSSDSSASHRRHKSRRRRGCRSRGSEMGVLPLKGKVKDSFAVVKKSSDPYNDFRMSMLEMIV
EKQIFS

      [1]
      8.7e-17
      YLQLNGKEYHGYIVGAFVQIW
      ++++++++++ ++++++++++
226  AKDLEQLLQCFLSLNSHHHHNVILEVFTEIWEALFSDWGS
```

CmOFP8
LENGTH = 301 COMBINED P-VALUE = 1.88e-33 E-VALUE = 1.6e-31
DIAGRAM: 231-[2]-11-[1]-7

```
     [2]                    [1]
     1.7e-26                   1.3e-17
     GESVAVVKYSDDPYEDFRRSMVEMIVENGIY     YLQLNGKEYHGYIVGAFVQIW
      +++++++++ +++++++++++++++++++      ++++++++++++++++++++
226
TVEGKIRESFAVVKKSADPFEDFKRSMVEMIMEKEMFEEKDLEQLLHCLLSLNDREHHGIIVEAFAEIWQS
LFCN
```

SlOFP6
LENGTH = 391 COMBINED P-VALUE = 2.17e-33 E-VALUE = 1.8e-31
DIAGRAM: 319-[2]-11-[1]-9

```
          [2]                    [1]
          2.0e-25                   4.7e-18
          GESVAVVKYSDDPYEDFRRSMVEMIVENGIY     YLQLNGKEYHGYIV
          ++++++++++++++++++ ++++++++++++     ++++++++++++++
301
PPRLSVFKKLIPCNVEGKVKESFAIVKKSEDPYEDFKSSMMEMILEKKIFEKNDLEQLLQCFLSLNAKNCH
GVIV

  GAFVQIW
  +++++++
376  EAFSEIWETLFSPNHN
```

CsOFP13c
LENGTH = 227 COMBINED P-VALUE = 2.59e-33 E-VALUE = 2.2e-31
DIAGRAM: 132-[2]-11-[1]-32

[2]
4.5e-24
GESVAVVKYSDDPYEDFR
+ + +++++++++++++

76
SARVSLSTEFEDDLELVIRGAKSERLIFEPGETNSILEKSRGVEEGGKCEESIRFEGSVVVLMAMESEDPYL
DFR

[1]
7.9e-18
RSMVEMIVENGIY YLQLNGKEYHGYIVGAFVQIW
+++++++++++++ +++++++++++++++++++++

151
RSMEEMVECHGIRNWEWLEELLNWYLRMNGMKNHGYILGAFVDLLVDLGGGDGSTDSTSIFSDDLIIQ
RHDRERC

CmOPF18
LENGTH = 269 COMBINED P-VALUE = 3.88e-33 E-VALUE = 3.3e-31
DIAGRAM: 190-[2]-11-[1]-16

[2]
4.6e-23
GESVAVVKYSDDPYEDFRRSMVEMIVENGIY
+ +++++++++ +++++++++++++++++++

151
EEESEGTEALVNSSMSFSDDVSPVKRSKRATCLRKLEGKMGKSFVQVKRSKEPQEDFKRSMVQMILEKEI
FETKG

[1]
5.9e-20
YLQLNGKEYHGYIVGAFVQIW
+++++++++++++++++++++

226 LEELLQCYLTLNSPEYHRIIVGAFSEVWEFLFCDSHSNKAVQCD

CmOFP9
LENGTH = 227 COMBINED P-VALUE = 6.82e-33 E-VALUE = 5.8e-31
DIAGRAM: 132-[2]-11-[1]-32

[2]
4.5e-24
GESVAVVKYSDDPYEDFR
+ + +++++++++++++

76
ARVSLSTEFEDDLELVIQGAKSERLIFEPGETNSILDQSRGGKSEGGKCESILRFEGSVVVLMAMESEDPYL
DFR

[1]
7.9e-18
RSMVEMIVENGIY YLQLNGKEYHGYIVGAFVQIW
+++++++++++++ +++++++++++++++++++++

151
RSMEEMVECHGIRNWEWLEELLNWYLRMNGMKNHGYILGAFVDLLVDLGGADGSTDSTSIFSDDLIIQ
PHDRERC

CsOPF13a
LENGTH = 234 COMBINED P-VALUE = 1.04e-32 E-VALUE = 8.8e-31
DIAGRAM: 25-[4]-42-[2]-11-[1]-54

[4]
6.0e-05
KPSFSPMLCRLPRCGNLRTLSIRDENNHNIFNSQRFYNNVDDDMVDEVIE
+ ++   ++ ++   ++ +   +   + +
1
MLTKKKKMMMMMRLPSLFKYLAIDDKSTFPWPSCRQPRTLSFRTTSAAVATATDSSDSFFTLSSESSGSLS
TVSE

[2]
1.1e-22
GESVAVVKYSDDPYEDFRRSMVEMIVENGIY
++ ++ +++++++ ++++++++++++++++
76
SSGGDPIERMIRDLRSTKRLHFEPTGKSSSIVEDDTVSHPLKEGTTVMSMDSDDPYSDFRKSMEEMVEAH
GMKDW

[1]
7.9e-18
YLQLNGKEYHGYIVGAFVQIW
+++++++++++++++++++++
151
ESLEELLNWYLRVNGKKNHGFILGAFVDLLVSLAMASSSSSSSCSSSLCCYSSSSSSSSLPCVSSSMEIEE
ISSL

AtOFP8
LENGTH = 221 COMBINED P-VALUE = 1.44e-32 E-VALUE = 1.2e-30
DIAGRAM: 152-[2]-11-[1]-6

[2]   [1]
3.6e-28   1.4e-14
GESVAVVKYSDDPYEDFRRSMVEMIVENGIY   YLQLNGKEYHGYIVGAFVQIW
++++++++++++++++ ++++++++++++   +++++++++++++++++ +++
151
SKAESFAVVKKSKDPYEDFRTSMVEMIVERQIFAPAELQQLLQCFLSLNSRQHHKVIVQVFLEIYATLFSP

CsOVATE
LENGTH = 301 COMBINED P-VALUE = 1.78e-32 E-VALUE = 1.5e-30
DIAGRAM: 232-[2]-11-[1]-6

[2]   [1]
8.3e-26   2.7e-17
GESVAVVKYSDDPYEDFRRSMVEMIVENGIY   YLQLNGKEYHGYIVGAFVQIW

+++++++++ +++++++++++++++++++ +++++++++++++++++++

226
CTVEGKIRESFAVVKKSADPFEDFKRSMMEMIMEKEMFEEKDLEQLLHCLLSLNDREHHGIIVEAFSEIW
QSLFC

CmOFP5
LENGTH = 235 COMBINED P-VALUE = 1.82e-32 E-VALUE = 1.5e-30
DIAGRAM: 25-[4]-42-[2]-11-[1]-55

[4]
6.2e-05
KPSFSPMLCRLPRCGNLRTLSIRDENNHNIFNSQRFYNNVDDDMVDEVIE
+ ++ ++ ++ ++ + + + +

1
MLTKKKKMMMMMRLPSLFKILAIDDKSTFPWPSCRQPRTLSFRTTSAAVATATDSSGSFFTLSSESSGSLS
TVSE

[2]
2.5e-22
GESVAVVKYSDDPYEDFRRSMVEMIVENGIY
++ ++ +++++++ ++++++++++++++++

76
SSGGDPIERMIRDLRSTKRLHFEPTGKSSSIVEDETVSHPLKEGTTVMSMDSNDPYSDFRKSMEEMVEAH
GMKDW

[1]
7.9e-18
YLQLNGKEYHGYIVGAFVQIW
+++++++++++++++++++++

151
ESLEELLNWYLRVNGKKNHGFILGAFVDLLVSLAMASSSSSSSSSSSLCCYSSSSSSSSLPCVSSSMEIE
EISS

CmOFP21
LENGTH = 289 COMBINED P-VALUE = 2.20e-32 E-VALUE = 1.9e-30
DIAGRAM: 123-[2]-11-[1]-103

[2]
2.4e-26
GESVAVVKYSDDPYEDFRRSMVEMIVE
+++++++++++++++++++++++++++

76
EALVRGLRVRQGKRLFLELDETNSIMTATATTTVAVATVGAGNYHVPFKESVAMAMESKDPYLDFKKSMEE
MVEA

[1]
3.0e-16
NGIY YLQLNGKEYHGYIVGAFVQIW
++++ ++++++ +++++++++++++

151
HELKDWKGMERLLSWYLKANGNANHEFIIGAFVDLLVDLAFAASSNLSNNSSSSPSSSSSSTTTTSSLLC
SSSSS

CmOFP14
LENGTH = 241 COMBINED P-VALUE = 3.48e-32 E-VALUE = 3e-30
DIAGRAM: 169-[2]-11-[1]-9

```
               [2]                        [1]
               2.0e-25                        8.7e-17
               GESVAVVKYSDDPYEDFRRSMVEMIVENGIY         YLQLNGKEYHGYIV
                +++++++++ +++ +++ ++++++++++++         ++++++++++ +++
151
TLFSSKSRSSDSSKHLTDDRNSFAVVKKSSDPYNDFRMSMLEMIVEKQIFSAKDLEQLLQCFLSLNSHHH
HNVIL

   GAFVQIW
   +++++++
226  EVFTEIWEALFSDWGS
```

CsOFP10
LENGTH = 270 COMBINED P-VALUE = 3.67e-32 E-VALUE = 3.1e-30
DIAGRAM: 191-[2]-11-[1]-16

```
                            [2]
                            2.8e-22
                            GESVAVVKYSDDPYEDFRRSMVEMIVENGIY
                            + +++++++++ ++++++++++++++++++
151
GEKESEETDALVNSSISFSDDVSPVKRSKRALYLRKLEGKMGKSFVQVKRSKEPQEDFKRSMAQMILEKEI
FEIK

        [1]
        5.9e-20
        YLQLNGKEYHGYIVGAFVQIW
        +++++++++++++++++++++
226  GLEELLQCYLTLNSPEYHRIIVGAFSEVWEFLFYDSHLNKAVQRD
```

SlOFP9
LENGTH = 266 COMBINED P-VALUE = 5.37e-32 E-VALUE = 4.6e-30
DIAGRAM: 184-[2]-11-[1]-19

```
                        [2]
                        2.3e-24
                        GESVAVVKYSDDPYEDFRRSMVEMIVENGIY
                        ++++++++++++++ +++ ++ +++++++++
```

151
RKKNNNNNTKVRRLRRYLSNSLKDSMMPCMADGKVNESFAIVKRSVDPYDDFKNSMKEMIMEKEMFEAE
DLEQLLL

[1]
1.1e-17
YLQLNGKEYHGYIVGAFVQIW
++++++++++ ++++++++++
226 CFLSLNSRHHHAIIVEAFTEIWEELFGKSSKSMDLKLPRFQ

SlOFP3
LENGTH = 298 COMBINED P-VALUE = 5.53e-32 E-VALUE = 4.7e-30
DIAGRAM: 16-[4]-73-[2]-11-[1]-96

[4]
3.7e-08
KPSFSPMLCRLPRCGNLRTLSIRDENNHNIFNSQRFYNNVDDDMVDEVIE
+ ++ ++ ++ + +++ + + ++ + +
1
MKLSSLFKNSSQNSSSTTTTTPWPWSLPTCGKPKTLSFRLEKNQHNIYNSTFHLDDINDTTSCSFDDFFS
EIDET

[2]
4.9e-20
GESVAVVKYSD
++ + ++++
76
SSSSTTTINGQDCIEKVIKGLRLEKERLFFEPEETSSILDFQENKNISITSSNININVVDEGNIISFVPMGLDS
N

[1]
1.9e-17
DPYEDFRRSMVEMIVENGIY YLQLNGKEYHGYIVGAFVQIW
++++++++++++++++++++ +++++++ +++++++++++++
151
DPFVDFRKSMEEMVEAYEIKDWENLEELLTCYLKVNCKSNHGYIVGAFVDLLVNLATFSDNNNNVGVDI
GAGVGA

AtOFP7
LENGTH = 315 COMBINED P-VALUE = 8.37e-32 E-VALUE = 7.1e-30
DIAGRAM: 224-[2]-11-[1]-28

[
3
G
+
151
EEETDRESLLPSSTNLSPEYSSSELPRVTRRPRQLLKKAVIEEESESSSPPPSPARLSSFVQRLMPCTMAAA
VMV

2] [1]

.2e-26 3.7e-16
ESVAVVKYSDDPYEDFRRSMVEMIVENGIY YLQLNGKEYHGYIVGAFVQIW
++++++++++++++++ ++++++++++++ +++++++ +++ +++++++++
226
EGVAVVKRSEDPYEDFKGSMMEMIVEKKMFEVAELEQLLSCFLSLNAKRHHRAIVRAFSEIWVALFSGGS
GGGRR

AtOFP5
LENGTH = 349 COMBINED P-VALUE = 1.41e-31 E-VALUE = 1.2e-29
DIAGRAM: 280-[2]-11-[1]-6

[2]
3.8e-26
GESVAVVKYSDDPYEDFRRS
++++++++++ +++++++ +
226
RELNRIGTKGNNKVRVFSPRASEKCRVKAIEDLKKAKQRAREHELLIETADGGMENESFAVVKCSSDPQK
DFRDS

[1]
8.9e-15
MVEMIVENGIY YLQLNGKEYHGYIVGAFVQIW
++++++++++ +++++ +++++ ++ ++ ++
301 MIEMIMENGINHPEELKELLVCYLRLNTDEYHDMIISVFQQVHNDFNFH

SlOFP19
LENGTH = 211 COMBINED P-VALUE = 5.44e-31 E-VALUE = 4.6e-29
DIAGRAM: 125-[2]-15-[1]-19

[2]
2.3e-24
GESVAVVKYSDDPYEDFRRSMVEMI
++++++ ++++++ ++++++++++
76
TNCTFTSFEDSDYTNIPDFSNIFASQRFFFSSPGNSNSIIDFPPENPKVVTGGVAVQTYSPDPYSDFRRSM
QEMV

[1]
1.2e-16
VENGIY YLQLNGKEYHGYIVGAFVQIW
+++++ +++++++++++++++++++++
151 EAHELTNVKANWGFLHELLLCYLNLNPKHTHKYIIRAYSDLVVSLMSMDDSEKKTEGIARP

SlOFP5
LENGTH = 377 COMBINED P-VALUE = 1.18e-30 E-VALUE = 1e-28
DIAGRAM: 90-[3]-173-[2]-11-[1]-20

[3]
5.6e-05
MGNYKFRFSDMMPNAWFYKLKDMGKAKRHKN

+ ++ +++ + + + ++++
76
NPLWCGECDQNSKSSLGEENHKFNDMVSRKISEKPKNEAEFSNRKRNSVKDEKLRKLSRKALEERIAEN
AREEVT

[2]
3.2e-2
GESVAV
+++++
226
EMSEKSGCQQRKSVYINQKRRRKHGIKVRAYSPRTAKMECRIKALEDMKKARMKTRHETKESFTGDRTVF
DSYAI

[1]
0 2.1e-19
VKYSDDPYEDFRRSMVEMIVENGIY YLQLNGKEYHGYIVGAFVQIW
++++++++ +++ ++++++ ++++ ++++++++++++++++ +++
301
MKSSFDPFSDFRDSMIEMITQRGIKSSEELEELLACYLTLNCDEYHDIIIKVFRQVWFELNQINIGEELQKC
CCS

AtOFP13
LENGTH = 260 COMBINED P-VALUE = 1.55e-29 E-VALUE = 1.3e-27
DIAGRAM: 11-[4]-83-[2]-13-[1]-51

[4]
7.4e-05
KPSFSPMLCRLPRCGNLRTLSIRDENNHNIFNSQRFYNNVDDDMVDEVIE
+ + + + +++ + ++
1
MGKKKMKLSSLFKGGAGGLLAVPLCYNAKTLSFRVGDDMIKTVNSVFFDHHHNNNNGGDLLEAETPES
WFTNSSE

[2]
7.2e-2
GESVAV
+ ++++
76
TASHSTESDQDLDAESLEMVVRGVVRSERLFFDPGVTSSILEEIEEKSKSDLKSKETVAVGEDRSTPIEEISV
AV

[1]
2 9.4e-16
VKYSDDPYEDFRRSMVEMIVENGIY YLQLNGKEYHGYIVGAFVQIW
+++++++++++++++++++ ++ ++++++++ +++++ ++++++
151
AMESEDPYGDFRRSMEEMVTSHGELAKDWESLESMLAWYLRMNGRKSHGVIVSAFVDLLSGLSDSGA
GITSASVS

CmOFP3
LENGTH = 176 COMBINED P-VALUE = 1.67e-29 E-VALUE = 1.4e-27

DIAGRAM: 101-[2]-11-[1]-12

```
                [2]                        [1]
                4.1e-23                       6.1e-15
                GESVAVVKYSDDPYEDFRRSMVEMIVENGIY        YLQLNGK
                +++++++++++++ ++++ ++ ++++++++         + +++++
76
DQMIREKREVRNGKERKKQRSEDTKFVVMVAMEKCSDDPKEDFRVSMTEMILANRIEEPKDLRNLLNYYI
SMNSD



   EYHGYIVGAFVQIW
   ++++++++++++++
151  ECHGVIFEVFHEVCSNLFLACKRHYW
```

SlOFP10
LENGTH = 229 COMBINED P-VALUE = 2.80e-29 E-VALUE = 2.4e-27
DIAGRAM: 114-[2]-11-[1]-52

```
                      [2]
                      1.1e-21
                      GESVAVVKYSDDPYEDFRRSMVEMIVENGIY
                      +++++ +++++++++++++++ ++++  +++
76
SHEEIIKGARSERLFFEQVATSSIFQEPQEENQENDLPFKESVILAMESKDPYLDFKKSMKEMVESQGIKD
WDNL

      [1]
      2.1e-16
      YLQLNGKEYHGYIVGAFVQIW
      ++++++ ++++++++++++++
151
QELLACYLKLNGEVNHGFVLGAFVDLLVELVIPTTPSTNSDNSITSYSSVASSSFSCPSSPLSSLGHKETEE
QEN
```

CmOFP6
LENGTH = 284 COMBINED P-VALUE = 3.76e-29 E-VALUE = 3.2e-27
DIAGRAM: 146-[2]-11-[1]-75

```
                                            [2]
                                            1.0e
                                            GESV
                                            ++++
76
DYSSSLHTNSSDSVSATNSTPAMDSEESLETVVRGARSERLFFEPDDTSSILEKSKPIESVETDELPRSGFK
ESL

                     [1]
   -20                   3.4e-17
   AVVKYSDDPYEDFRRSMVEMIVENGIY        YLQLNGKEYHGYIVGAFVQIW
```

++ +++++++++++++ ++++ ++ + ++++++++++++++++++++++

151 IVSIESENPYEDFRKSMGEMVESHGVKDWDGLEELLGWYLKANWKNNHRFIIGAFVDLLIHILLASSSSSSTST

CsOFP12-16c

LENGTH = 197 COMBINED P-VALUE = 7.09e-29 E-VALUE = 6e-27

DIAGRAM: 105-[2]-15-[1]-25

[2]

6.4e-22

GESVAVVKYSDDPYEDFRRSMVEMIVENGIY

+++ +++++++++ +++++++++++++++

76 RFFFSSPGRSNSIFEYSSCSRRQQPHDVLVSEGHRIRKYSMDPYADFRRSMQEMVEARELEDVRSDSEFLRELLS

[1]

7.8e-17

YLQLNGKEYHGYIVGAFVQIW

+++++++++++++++++++++

151 CYLRLNPKNTHKFIVKAFSDLVLSLLASSSPTPAPASIARRKVVTSR

CsOFP13b

LENGTH = 277 COMBINED P-VALUE = 1.37e-28 E-VALUE = 1.2e-26

DIAGRAM: 134-[2]-11-[1]-80

[2]

1.0e-20

GESVAVVKYSDDPYED

++++++ ++++++++

76 SVSATNSTPAVDSEESLETVVRGARSERLFFEPDDTSSILEKSKSIDSVETELLPKSGFKESLIVSIESENPYED

[1]

3.4e-17

FRRSMVEMIVENGIY YLQLNGKEYHGYIVGAFVQIW

+++++ ++++ ++ + +++++++++++++++++++++

151 FRKSMGEMVESHGVKDWDGLEELLGWYLKANWKNNHRFIIGAFVDLLIHILLASSSSSSSTSTSTSSSSSSSLC

CsOFP8a

LENGTH = 239 COMBINED P-VALUE = 1.42e-28 E-VALUE = 1.2e-26

DIAGRAM: 170-[2]-11-[1]-6

[2] [1]

1.9e-23 1.9e-13

GESVAVVKYSDDPYEDFRRSMVEMIVENGIY YLQLNGKEYHGYI

++++++++ + ++++++++++++++++++++ + +++++++++++
151
VSRRRHRRRHGRRRPERKMRDGFFAVVKNSSNPYMDFKASMAEMVVEKKIFGGKELEELLQCFISLNSR
HYHKVI

VGAFVQIW
+++++++
226 FEVYSEIKEALFFL

CmOFP20
LENGTH = 237 COMBINED P-VALUE = 2.28e-28 E-VALUE = 1.9e-26
DIAGRAM: 168-[2]-11-[1]-6

[2] [1]
1.5e-24 1.9e-13
GESVAVVKYSDDPYEDFRRSMVEMIVENGIY YLQLNGKEYHGYIVG
++++++++ + ++++++++++++++++++++ + +++++++++++++
151
RRHRQRHGRRRPPERKMRDGFFAVVKNSSDPYKDFKASMAEMVVEKKIFGGKELEELLQCFISLNSRHY
HKVIFE

AFVQIW
+++++
226 VYSEIKEALFFL

CsOFP5b
LENGTH = 168 COMBINED P-VALUE = 3.58e-28 E-VALUE = 3e-26
DIAGRAM: 87-[2]-11-[1]-18

[2] [1]
4.1e-23 1.0e-12
GESVAVVKYSDDPYEDFRRSMVEMIVENGIY YLQLNGKEYHGYIVGAFVQIW
+++++++++++++ ++++ ++ ++++++++ + +++++++++++++++++
76
ERRKQRSEDTKFVVMVAMEKCSDDPKEDFRVSMTEMILANRIEEPKDLRNLLNYYISMNSDECHGVIFEV
FHEKE

CmOFP7
LENGTH = 263 COMBINED P-VALUE = 3.62e-27 E-VALUE = 3.1e-25
DIAGRAM: 148-[2]-23-[1]-40

[2
5.
GE
++

76
LANDIGVADPDAYVAVDFITAFTSHRFFFSSPGSSNSIIESTTPTTTESTTTMSLSSEYSARYEGNDDELMIFN
N

]   [1]
1e-22   4.2e-15
SVAVVKYSDDPYEDFRRSMVEMIVENGIY   YLQLNGKEYHGYIVGAFVQIW
++++ ++++++++++++++++++++ +   ++ ++++++++ ++++++++
151
SHVIPTYSPDPYMDFRRSMQEMVEAREKMTTAATTTTMKKSSWEFLHELLLCYLALNPKATHKHILKAFAD
VATV

CsOFP12-16a
LENGTH = 278 COMBINED P-VALUE = 1.10e-26 E-VALUE = 9.3e-25
DIAGRAM: 153-[2]-24-[1]-49

[2]   [1]
2.0e-21   3.8e-15
GESVAVVKYSDDPYEDFRRSMVEMIVENGIY   YLQLNGKEYHGYIVGAF
++++++ ++++++++++++++++++++ +   ++ ++++ +++ +++++
151
MIFNNSHVIPTYSPDPYMDFRRSMQEMMEAREKMTTAVATTTTMKKSSWEFLHELLLCYLALNPKTTHKH
ILKAF

VQIW
+++
226 ADIATVIKPPLAMKETEEEENVDREKGESMVDDRGAGGGGCECEMSGQQNDRD

CmOFP13+1
LENGTH = 154 COMBINED P-VALUE = 3.58e-26 E-VALUE =   3e-24
DIAGRAM: [3]-123

[3]
4.9e-33
MGNYKFRFSDMMPNAWFYKLKDMGKAKRHKN
+++++++++++++++++++++++++++++++
1
MRNHKFRFSDMIPNAWFYKLKEIGGASRPKSFRSNKNPHHPPPPPPPSKHKQPPPPPPHSRSRKSYYFT
RQLESN

CsOFP12-16b
LENGTH = 205 COMBINED P-VALUE = 2.07e-25 E-VALUE = 1.8e-23
DIAGRAM: 111-[2]-16-[1]-26

[2]
2.9e-20
GESVAVVKYSDDPYEDFRRSMVEMIVENGIY
++++ +++ +++++++++++++++++++

76
AAVASHRFFFSSPGCSNSIFDSSPDTHHSTAVSAAVHGGVEVRKVSMDPFVDFRASMQEMVEARDRPV
DVRRDWE

[1]
7.4e-15
YLQLNGKEYHGYIVGAFVQIW
++++++ +++++++++++++
151 YLQELLLCYLQINPVDTHKFILRAFSDLVVYLLESSPESFSDRRIRPHNINSNSW

AtOFP10
LENGTH = 196 COMBINED P-VALUE = 3.73e-25 E-VALUE = 3.2e-23
DIAGRAM: 94-[2]-11-[1]-39

[2] [1]
3.1e-21 6.5e-13
GESVAVVKYSDDPYEDFRRSMVEMIVENGIY YLQLNGKEYHGYIV
+++++++++++++++ ++++ ++++++ + ++ +++ + +++
76
YTPGPPVSPTVLRSPCPKIDESVAMAKESINPFEDYKKSMNQMIEERYIETESELKELLRCFLDINPSPQHN
LIV

GAFVQIW
+++++++
151 RAFVDVCSHLQPPHDRRGKSLGRLLRLYVNNPLDNNDDDSHQTSSK

CmOFP1
LENGTH = 205 COMBINED P-VALUE = 4.30e-25 E-VALUE = 3.7e-23
DIAGRAM: 111-[2]-16-[1]-26

[2]
2.9e-20
GESVAVVKYSDDPYEDFRRSMVEMIVENGIY
++++ +++ +++++++++++++++++++
76
AAVASHRFFFSSPGCSNSIFDSSPDTHHSAAVSAAVHGGVEVRKVSMDPFVDFRASMQEMVEARDRPV
DVRRDWE

[1]
1.3e-14
YLQLNGKEYHGYIVGAFVQIW
++++++ +++++++++++++
151 YLQDLLLCYLRINPVDTHKFILRAFSDLVVYLLECSPESFSDRRLRPHNINSNSW

SlOFP15
LENGTH = 168 COMBINED P-VALUE = 6.26e-25 E-VALUE = 5.3e-23
DIAGRAM: 91-[2]-11-[1]-14

[2] [1]
4.4e-20 2.5e-13
GESVAVVKYSDDPYEDFRRSMVEMIVENGIY YLQLNGKEYHGYIVGAF
++++++++++++ ++++++ ++++ ++++ + ++++ ++ +++++++
76
MRRRRRRAERDEKTKFIVMIAMEKSSYDPREDFRESIEQMIIANRICDPKDLRRLLNYYVSMNAEEYRGVIL
EVF

VQIW
++++
151 HQVCTTFFLSCKQPSSQV

AtOFP12
LENGTH = 226 COMBINED P-VALUE = 7.06e-25 E-VALUE = 6e-23
DIAGRAM: 148-[2]-15-[1]-11

[2
7.
GE
++
76
STAANSSSSSASYDDSDNYGFAPDDDSPPPDLTAVLASRRFFFSSPGCSNSITDSPDLRCRDNYDTATRL
LTGGT

] [1]
2e-18 1.1e-16
SVAVVKYSDDPYEDFRRSMVEMIVENGIY YLQLNGKEYHGYIVGAFVQIW
++ + +++++ ++++++++++++ ++++++ +++++++++++++
151
AVKHYVQSPDPYNDFRRSMQEMIDAVTNAGDLRRYEFLHELLLSYLSLNAADTHKFIIRAFADILVSLLSD
GHRI

AtOFP14
LENGTH = 294 COMBINED P-VALUE = 9.76e-25 E-VALUE = 8.3e-23
DIAGRAM: 189-[2]-16-[1]-37

[2]
2.7e-19
GESVAVVKYSDDPYEDFRRSMVEMIVENGIY
++++++ + + ++++++++++++++ ++++
151
DLLRTERLSPPPGSSEGRPSMETTSTSSERQSRSTLVLPENCIAVLRYTDEPQEDFRQSMVEMMESKLGM
RESEV

[1]
9.8e-15
YLQLNGKEYHGYIVGAFVQIW
++ +++++ +++++ ++++++

226
DWDLMEELLFCYLDLNDKKSHKFILSAFVDLIIALREKEKRITRKGHVRSLSTRAARDRLRKRMIMSDN

SlOFP7
LENGTH = 275 COMBINED P-VALUE = 2.20e-23 E-VALUE = 1.9e-21
DIAGRAM: 48-[4]-81-[2]-15-[1]-29

```
                          [4]
                          1.8e-05
                          KPSFSPMLCRLPRCGNLRTLSIRDENN
                           ++++
1
MPKQLQKSLSDYLTKKKKKATAQQTTNSANKTLSSSTSWLLRGCRHPKTPSFSAVDRKEKNVQGENEAAT
LADVD

  HNIFNSQRFYNNVDDDMVDEVIE
   ++   +  +++  ++++
76
RFVFENFKSFYYKDDDNEAEIVENPNSLSESPRHIIPPLNHTGSRRFFIAPGSSSSLIEEARTSMTVSDDTG
STS

                [2]
                5.0e-18
                GESVAVVKYSDDPYEDFRRSMVEMIVENGIY
                ++++  + +++ ++ ++++++++++++++
151
AITITTVTNTNSNELSAISTEYSKETLNANDFITLVTYSPSPYDDFRQSMQEMMEARLKDQGKINWEFMEEL
LFC

  [1]
  5.1e-13
  YLQLNGKEYHGYIVGAFVQIW
  ++ +++++ +++++ ++++ +
226  YLDLNDKKSYKYILSAFVDQIVILRENSGRVPAISRNVRPLDGELNQRDT
```

AtOFP15
LENGTH = 261 COMBINED P-VALUE = 2.38e-23 E-VALUE = 2e-21
DIAGRAM: 106-[2]-12-[1]-91

```
                 [2]                      [
                 3.6e-19                     4
                 GESVAVVKYSDDPYEDFRRSMVEMIVENGIY        Y
                 ++++  +++++++ +++++++++++++ ++         +
76
RSSERLIFESKGETNSILEEATSKREEEDEEEGFMLFSLESDDPYSDFKRSMEEMVEAHALHHDWKSLEKL
LLQF

  1]
  .5e-14
```

LQLNGKEYHGYIVGAFVQIW
++++++ ++++++++++++
151
LKVNAKTSHRYIFAAFVDLLMNLALDTKKAIINNDISKDDGVSASRAAAAGEASTSCCNSMTLGESPSSPL
SFYT

AtOPF16
LENGTH = 244 COMBINED P-VALUE = 2.96e-23 E-VALUE = 2.5e-21
DIAGRAM: 161-[2]-20-[1]-11

[2] [1]
1.8e-17 2.1e-15
GESVAVVKYSDDPYEDFRRSMVEMIVENGIY YLQLNGKEYHGYI
++++ + ++++++ ++++++++++++ ++++++ +++++
151
VTTTTTRLISGGTAVTQHVDSPDPLTDFRRSMQEMIDAAIDAGELSRDPNDGYDFLDELLLTYLSLNPADT
HKFV

VGAFVQIW
++++++++
226 IRAFSDILVSLLSEERRIC

AtOFP11
LENGTH = 182 COMBINED P-VALUE = 4.77e-23 E-VALUE = 4.1e-21
DIAGRAM: 99-[2]-16-[1]-15

[2] [1]
1.6e-17 4.2e
GESVAVVKYSDDPYEDFRRSMVEMIVENGIY YLQL
++++ +++++++++ +++ ++++++ + ++ +
76
FSTNRREEEEEEDETTTSVSKLLSGGTAIMKHIESPDPYRDFGRSMREMVEARDLTRDVVADREYLHELLFC
YLYL

-15
NGKEYHGYIVGAFVQIW
++++++++++ ++++ +
151 NPKHTHRFIVSAFADTLLWLLSPSPSPEHFLS

CsOFP14
LENGTH = 272 COMBINED P-VALUE = 8.49e-23 E-VALUE = 7.2e-21
DIAGRAM: 178-[2]-15-[1]-27

[2] [
6.6e-18 3
GESVAVVKYSDDPYEDFRRSMVEMIVENGIY Y
+++++ +++++ + +++++++++++++ + +

151
SHTESSENAGSSSSSLIGEDRGKDLKLPSDCIAILRKSPNPSEEFRRSMQEMMDAHLKQHEKVDWEFME
ELLFCY

1]
.2e-14
LQLNGKEYHGYIVGAFVQIW
++++ ++ +++++ ++++++
226 LNLNEKKSYKYILNAFVDLIVILRQKAEEAPAKPRTVRSVRMVRRMI

CmOFP10
LENGTH = 298 COMBINED P-VALUE = 4.56e-22 E-VALUE = 3.9e-20
DIAGRAM: 204-[2]-15-[1]-27

[2]
4.6e-17
GESVAVVKYSDDPYEDFRRSM
+++++ +++++ + +++++
151
SPVDSYGGSHRFFFSPDLSGSDLPDDSHTESSENAGSSSSSLIGEDRGKDLKLPSDCIAILRKSPNPSEEF
RRSM

[1]
2.4e-14
VEMIVENGIY YLQLNGKEYHGYIVGAFVQIW
+++++ ++ + ++++++++ +++++ ++++++
226
QEMMDGHLKHHEKVDWEFMEELLFCYLNLNDKKSYKYILNAFVDLIVILRQKAEEEPAKPRTVRSVRMVR
RMI

AtOFP18
LENGTH = 282 COMBINED P-VALUE = 1.82e-20 E-VALUE = 1.5e-18
DIAGRAM: 133-[2]-12-[1]-85

[2]
2.5e-16
GESVAVVKYSDDPYEDF
++ + +++++++ ++
76
YSSFSSTSHAIENPPEIESIENVIKGLKSSKRLIFERRGTSNSILEEATKRDDHEEEEDGLMLLSLESNDPYTD
F

[1]
1.8e-13
RRSMVEMIVENGIY YLQLNGKEYHGYIVGAFVQIW
+ +++ +++ + ++ +++++ ++ ++++++++++++
151
KNSMEKMVEVHVLHHDWISLEKLLFWFLKVNVKASHRYIFAAFVDLVLNLAVGPSKDVAGEPNSDVVVE
DSLSSS

SlOFP12
LENGTH = 158 COMBINED P-VALUE = 2.22e-18 E-VALUE = 1.9e-16
DIAGRAM: 57-[2]-16-[1]-33

```
                              [2]
                              5.6e-14
                              GESVAVVKYSDDPYEDFR
                              ++++  +++ +++ +++
1
MTRKYDQCLVDNMFGPFPESCCPDEALEMAKQALATRRLSFEENESCSVLSMVGFPFKDCLLLAVETEN
PKMDFL

                [1]
                3.5e-12
  RSMVEMIVENGIY           YLQLNGKEYHGYIVGAFVQIW
  ++++++   ++ +           +++++ +++  ++++++ +++
76
HSMEQMTKVYGAQRGDMVDWEFMEELLTWFLKINNMKNQHFIVAAFIDLCLGGHVQDVEPVENVEPLT
DDIVNVI
```

SlOFP11
LENGTH = 83 COMBINED P-VALUE = 1.76e-11 E-VALUE = 1.5e-09
DIAGRAM: 31-[2]-21

```
                 [2]
                 2.3e-16
                 GESVAVVKYSDDPYEDFRRSMVEMIVENGIY
                  ++  ++++++++  +++++  +++   +++
1
MVNYKGGKLLKHQRNRVSFSAKLPEDVRGAFADSTCVVKYSMDPLTDIKESIKEMVKNVGIKDWKEMEE
LVYCYI
```

SlOFP13
LENGTH = 188 COMBINED P-VALUE = 2.89e-08 E-VALUE = 2.5e-06
DIAGRAM: 104-[2]-12-[1]-20

```
                [2]                      [1]
                6.1e-07                      4.5
                GESVAVVKYSDDPYEDFRRSMVEMIVENGIY        YLQ
                ++ ++  + ++  + +   ++++++ + +         + +
76
ALKSFSGHIKAPVPSPITPAYARLSGATKKEVVIFQDDVEDACRSFENYLAEMIVEEGKMRDIMDVEELLYC
WKN


  e-09
  LNGKEYHGYIVGAFVQIW
  + +++  +++ +++++++
151  LKSPVFIDLVCRFYGELCKDLFSHTYKDDINSPQKIMQ
```

SlOFP8
 LENGTH = 229 COMBINED P-VALUE = 1.59e-06 E-VALUE = 0.00013
 DIAGRAM: 184-[1]-24

[1]
2.2e-10
YLQLNGKEYHGYIVGAFVQIW
+ ++++++ +++ +++ + +

151
SKGSLTLLKKMEELEMVEGEDMDHVLDIEEVLQCYTLLNSPVYVDIVDRFFMDMYTEFSIRKPSGSVNSS
MRRLG

CmOFP16
 LENGTH = 189 COMBINED P-VALUE = 7.98e-06 E-VALUE = 0.00068
 DIAGRAM: 107-[2]-12-[1]-18

[2]
4.4e-05
GESVAVVKYSDDPYEDFRRSMVEMIVENGIY
+++   + ++ + +   ++++ + +

76
HLRSSESVRSDNECREKLLFPSPMIRGRKVAAGTSWEEKEEVEDACKSFENYLVEMIIEEGKVRDLMDVE
ELLYC

[1]
1.1e-08
YLQLNGKEYHGYIVGAFVQIW
+ ++ +++ +++ +++++++

151 WRNLKCPVFVDLVSRFYGELCKDLFSSHIQAFTPNFQPK

AtOFP17
 LENGTH = 195 COMBINED P-VALUE = 2.69e-04 E-VALUE =   0.023
 DIAGRAM: 167-[1]-7

[1]
1.9e-08
YLQLNGKEYHGYIVGAFVQIW
+ ++ +++ +++ +++++++

151 EEGKIDDLMDIEELLFCWKNLKSPVFIELVSRFYGELCRDLFSGE

****

CPU: noble-meme.grid.gs.washington.edu
Time 0.060 secs.

mast -oc . -nostatus meme.xml sequences.fa

File S8. ANOVA, Kruskal-Wallis and Welch-ANOVA results full tables.

File S8A. ANOVA results for fruit shape index comparing location effect (TM vs IBMCP).

	Df	Sum Sq	Mean Sq	F value	Pr(>F)
Genotype	2	0.21767	0.10884	55.148	**4.28e-12*** **
Location	1	0.00005	0.00005	0.024	0.879
Genotype*Location	1	0.00046	0.00046	0.232	0.632
Residuals	39	0.07697	0.00197		

File S8B. ANOVA results for fruit shape index comparing season effect (autumn 2023 vs summer 2024).

	Df	Sum Sq	Mean Sq	F value	Pr(>F)
Genotype	2	0.23301	0.11651	63.532	**5.06e-15*** **
Season	1	0.03994	0.03994	21.782	**2.00e-05*** **
Genotype*Season	2	0.00443	0.00222	1.208	0.307
Residuals	55	0.10086	0.00183		

File S8C. ANOVA results for fruit area comparing TM and CRAG melons.

	Df	Sum Sq	Mean Sq	F value	Pr(>F)
Genotype	2	140	70	0.414	0.665
Season-Location	1	13028	13028	76.879	**5.10e-10*** **
Genotype*Season-Location	2	224	112	0.662	0.523
Residuals	32	5423	169		

File S8D. Welch-ANOVA results for fruit morphology comparing location effect (TM vs IBMCP).

	Genotype	Location	Line*Location
Pr(>WJ) length	**0.004907566** **	0.4346025	0.3839009
Pr(>WJ) width	0.2138837	0.2975861	0.1397055

File S8E. Welch-ANOVA results for fruit morphology comparing season effect (autumn 2023 vs summer 2024.

	Genotype	Season	Line*Season
Pr(>WJ) length	**0.0003130685*** **	**2.192106e-10*** **	0.8519637
Pr(>WJ) width	0.3348592	**3.306155e-11*** **	0.6070456

File S8F. ANOVA results for macroscopic ovary morphology.

	**Df**	**Sum Sq**	**Mean Sq**	**F value**	**P value**
Ovary length	11	126.75	11.523	21.67	**$7.12*10^{-14}$***
Residuals	42	22.33	0.532		

File S8G. Kruskal-Wallis ANOVA results for macroscopic ovary morphology.

	**Df**	**K-W chi squared**	**P value**
Ovary width	11	41.291	**$2.148*10^{-5}$*****
Ovary shape	11	36.52	**0.0001386*** **

File S8H. ANOVA results for microscopic ovary morphology.

	**Df**	**Sum Sq**	**Mean Sq**	**F value**	**P value**
Ovary shape	5	0.9253	0.18506	5.815	**0.000907*** **
Residuals	27	0.8593	0.03183		
Ovary distal end blockiness	5	0.06729	0.013457	1.685	0.172
Residuals	27	0.21567	0.007988		

S8I. Kruskal-Wallis ANOVA results for microscopic ovary morphology.

	**Df**	**K-W chi squared**	**P value**
Ovary length	5	20.668	**0.0009358*** **
Ovary width	5	14.153	**0.01467***

File S9A. Fruit phenotype raw data.

Line	Replicate	Location	Season	Area (cm^2)	Length (cm)	Width (cm)	Fruit Shape Index	Weight (g)
Wt	1	TM	Summer 2024	122,25	12,2174	12,3952	0,9857	-
Wt	2	TM	Summer 2024	143,08	13,0937	13,6398	0,9566	-
Wt	3	TM	Summer 2024	138,28	13,0302	13,4112	0,9633	-
Wt	4	TM	Summer 2024	110,00	11,4427	11,9888	0,9493	-
Wt	5	TM	Summer 2024	111,34	11,2268	12,2936	0,9104	-
Wt	6	TM	Summer 2024	111,66	11,6332	12,1793	0,9479	-
Wt	7	TM	Summer 2024	128,17	12,3444	13,0302	0,9340	-
Wt	8	TM	Summer 2024	125,77	12,3317	12,8143	0,9491	-
*ofp13+1*_het	1	TM	Summer 2024	120,02	12,1412	12,5349	0,9635	-
*ofp13+1*_het	2	TM	Summer 2024	135,05	13,0302	12,954	1,0019	-
*ofp13+1*_het	3	TM	Summer 2024	117,71	12,6746	12,0142	1,0317	-
*ofp13+1*_het	4	TM	Summer 2024	144,86	13,6906	13,3604	1,0218	-
*ofp13+1*_het	6	TM	Summer 2024	102,63	11,1506	11,3792	0,9821	-
*ofp13+1*_het	7	TM	Summer 2024	101,43	11,2141	11,3792	0,9855	-
*ofp13+1*_het	8	TM	Summer 2024	132,51	13,4747	12,3317	1,0925	-
*ofp13+1*_homo	3	TM	Summer 2024	101,31	11,6459	11,0744	1,0491	-
*ofp13+1*_homo	4	TM	Summer 2024	100,40	11,8872	10,8712	1,0909	-
*ofp13+1*_homo	5	TM	Summer 2024	124,57	13,5128	12,065	1,1199	-
*ofp13+1*_homo	6	TM	Summer 2024	153,11	15,0114	12,9794	1,1498	-
*ofp13+1*_homo	7	TM	Summer 2024	129,08	14,1224	11,8364	1,1702	-
*ofp13+1*_homo	8	TM	Summer 2024	121,58	13,2207	11,8491	1,1090	-
Wt	1	IBMCP	Summer 2024	-	13,0	14,0	0,9286	1237
Wt	2	IBMCP	Summer 2024	-	8,8	9,5	0,9263	473
Wt	3	IBMCP	Summer 2024	-	10,6	11,5	0,9217	758
Wt	4	IBMCP	Summer 2024	-	11,0	11,5	0,9565	859
Wt	5	IBMCP	Summer 2024	-	13,3	13,5	0,9852	1303
Wt	6	IBMCP	Summer 2024	-	12,0	11,9	1,0084	901
Wt	7	IBMCP	Summer 2024	-	12,0	12,0	1,0000	923
Wt	8	IBMCP	Summer 2024	-	12,7	13,6	0,9338	1207
Wt	9	IBMCP	Summer 2024	-	13,6	13,6	1,0000	1264
Wt	10	IBMCP	Summer 2024	-	8,7	9,0	0,9667	419
Wt	11	IBMCP	Summer 2024	-	13,0	13,5	0,9630	1310
Wt	13	IBMCP	Summer 2024	-	10,9	11,5	0,9478	723
Wt	14	IBMCP	Summer 2024	-	9,2	9,4	0,9787	475
Wt	15	IBMCP	Summer 2024	-	10,2	11,4	0,8947	748
*ofp13+1*_homo	1	IBMCP	Summer 2024	-	15,0	13,0	1,1538	1279
*ofp13+1*_homo	2	IBMCP	Summer 2024	-	13,0	11,9	1,0924	1024
*ofp13+1*_homo	3	IBMCP	Summer 2024	-	12,5	11,2	1,1161	854
*ofp13+1*_homo	4	IBMCP	Summer 2024	-	15,8	12,9	1,2248	1326
*ofp13+1*_homo	5	IBMCP	Summer 2024	-	14,9	13,0	1,0177	1292
*ofp13+1*_homo	6	IBMCP	Summer 2024	-	11,5	11,3	1,1462	829
*ofp13+1*_homo	7	IBMCP	Summer 2024	-	10,5	10,5	1,0000	638
*ofp13+1*_homo	8	IBMCP	Summer 2024	-	11,8	10,8	1,0926	740
*ofp13+1*_homo	9	IBMCP	Summer 2024	-	14,5	12,8	1,1328	1220
Wt	3	CRAG	Autumn 2023	76,61	9,0932	10,2362	0,8883	-
Wt	4	CRAG	Autumn 2023	84,00	10,0076	10,1854	0,9825	-

Wt	5	CRAG	Autumn 2023	87,42	10,1346	10,6426	0,9523	-
Wt	6	CRAG	Autumn 2023	79,27	9,4996	10,3632	0,9167	-
Wt	7	CRAG	Autumn 2023	69,05	8,6106	10,0076	0,8604	-
*ofp13+1*_het	1	CRAG	Autumn 2023	83,87	10,0076	10,4648	0,9563	-
*ofp13+1*_het	4	CRAG	Autumn 2023	102,10	11,3284	11,3284	1,0000	-
*ofp13+1*_het	11	CRAG	Autumn 2023	93,32	10,5918	10,9474	0,9675	-
*ofp13+1*_het	17	CRAG	Autumn 2023	78,18	9,3726	10,3124	0,9089	-
*ofp13+1*_het	19	CRAG	Autumn 2023	82,33	9,8044	10,3886	0,9438	-
*ofp13+1*_homo	6	CRAG	Autumn 2023	81,67	10,5664	9,9314	1,063939	-
*ofp13+1*_homo	13	CRAG	Autumn 2023	88,45	10,6680	10,7188	0,995261	-
*ofp13+1*_homo	14	CRAG	Autumn 2023	86,78	10,5156	10,5410	0,99759	-
*ofp13+1*_homo	15	CRAG	Autumn 2023	89,89	10,6680	10,7442	0,992908	-
*ofp13+1*_homo	18	CRAG	Autumn 2023	95,66	11,1252	10,9474	1,016241	-
*ofp13+1*_homo	20	CRAG	Autumn 2023	78,57	10,4394	9,7536	1,070313	-
*ofp13+1*_homo	27	CRAG	Autumn 2023	91,95	11,2268	10,3632	1,083333	-

File S9B. Macroscopic ovary phenotype raw data.

Line	Flower	Stage	Length (mm)	Width (mm)	Shape
*ofp13+1*_homo	F1	7DpreA	5,970	2,640	2,261
*ofp13+1*_homo	F2	7DpreA	5,430	3,620	1,500
*ofp13+1*_homo	F3	7DpreA	4,540	2,730	1,663
*ofp13+1*_homo	F4	7DpreA	5,375	3,430	1,569
Wt	F1	7DpreA	6,130	5,710	1,074
Wt	F2	7DpreA	3,740	2,860	1,308
Wt	F3	7DpreA	5,780	4,810	1,202
Wt	F4	7DpreA	4,630	4,540	1,020
Wt	F5	7DpreA	5,400	4,090	1,320
*ofp13+1*_homo	F1	6DpreA	5,070	2,120	2,392
*ofp13+1*_homo	F2	6DpreA	5,960	3,800	1,568
*ofp13+1*_homo	F3	6DpreA	6,410	3,880	1,652
*ofp13+1*_homo	F4	6DpreA	5,850	3,500	1,671
Wt	F1	6DpreA	6,730	5,720	1,177
Wt	F2	6DpreA	4,500	4,265	1,055
Wt	F3	6DpreA	6,410	4,580	1,400
Wt	F4	6DpreA	4,990	3,950	1,263
Wt	F5	6DpreA	5,960	4,080	1,461
*ofp13+1*_homo	F1	5DpreA	6,260	3,330	1,880
*ofp13+1*_homo	F2	5DpreA	6,695	5,040	1,331
*ofp13+1*_homo	F3	5DpreA	5,750	4,140	1,389
*ofp13+1*_homo	F4	5DpreA	6,050	4,380	1,381
Wt	F1	5DpreA	6,890	6,310	1,092
Wt	F2	5DpreA	5,850	4,960	1,179
Wt	F3	5DpreA	4,707	4,141	1,137
Wt	F4	5DpreA	6,090	5,090	1,196
Wt	F5	5DpreA	5,824	4,529	1,286
*ofp13+1*_homo	F1	4DpreA	6,670	3,360	1,985
*ofp13+1*_homo	F2	4DpreA	7,260	5,270	1,378
*ofp13+1*_homo	F3	4DpreA	8,200	5,430	1,510

*ofp13+1*_homo	F4	4DpreA	7,250	4,480	1,618
Wt	F1	4DpreA	6,550	6,310	1,038
Wt	F2	4DpreA	6,080	4,940	1,231
Wt	F3	4DpreA	6,650	5,390	1,234
Wt	F4	4DpreA	6,810	4,780	1,425
Wt	F5	4DpreA	7,640	4,940	1,547
*ofp13+1*_homo	F1	1DpreA	9,810	7,280	1,348
*ofp13+1*_homo	F2	1DpreA	8,630	5,530	1,561
*ofp13+1*_homo	F3	1DpreA	8,280	5,700	1,453
*ofp13+1*_homo	F4	1DpreA	7,850	5,590	1,404
Wt	F1	1DpreA	8,140	7,380	1,103
Wt	F2	1DpreA	7,080	6,010	1,178
Wt	F3	1DpreA	6,871	5,630	1,220
Wt	F4	1DpreA	7,570	6,410	1,181
Wt	F5	1DpreA	7,650	5,820	1,314
*ofp13+1*_homo	F1	Anthesis	10,080	7,080	1,424
*ofp13+1*_homo	F2	Anthesis	10,870	6,990	1,555
*ofp13+1*_homo	F3	Anthesis	10,330	6,600	1,565
*ofp13+1*_homo	F4	Anthesis	9,840	6,590	1,493
Wt	F1	Anthesis	10,690	7,380	1,449
Wt	F2	Anthesis	8,960	6,920	1,295
Wt	F3	Anthesis	8,750	7,370	1,187
Wt	F4	Anthesis	8,520	7,390	1,153
Wt	F5	Anthesis	8,090	6,390	1,266

File S9C. Microscopic ovary phenotype raw data.

Line	Flower	Replicate	Stage	Length (mm)	Width (mm)	Constriction (mm)	Shape	Distal End Blockiness
*ofp13+1*_homo	F1	1	E1	3,467	2,105	1,593	1,647	0,757
*ofp13+1*_homo	F1	2	E1	3,565	2,089	1,609	1,707	0,770
*ofp13+1*_homo	F1	3	E1	3,562	2,084	1,483	1,709	0,712
*ofp13+1*_homo	F2	1	E1	2,944	1,851	1,576	1,590	0,851
*ofp13+1*_homo	F2	2	E1	3,205	2,444	1,945	1,311	0,796
*ofp13+1*_homo	F2	3	E1	2,475	1,699	1,472	1,457	0,866
*ofp13+1*_homo	F3	1	E1	2,787	1,575	1,011	1,770	0,642
*ofp13+1*_homo	F3	2	E1	3,098	1,671	1,099	1,854	0,658
*ofp13+1*_homo	F3	3	E1	3,224	1,622	1,169	1,988	0,721
*ofp13+1*_homo	F4	1	E1	3,603	2,037	1,374	1,769	0,675
*ofp13+1*_homo	F4	2	E1	3,720	2,021	1,452	1,841	0,718
*ofp13+1*_homo	F4	3	E1	3,650	2,100	1,401	1,738	0,667
*ofp13+1*_homo	F5	1	E1	3,505	1,903	1,466	1,842	0,770
*ofp13+1*_homo	F5	2	E1	3,396	1,802	1,501	1,885	0,833
*ofp13+1*_homo	F5	3	E1	3,715	1,915	1,503	1,940	0,785
*ofp13+1*_homo	F6	1	E1	3,281	2,142	1,281	1,532	0,598
*ofp13+1*_homo	F6	2	E1	3,448	2,029	1,281	1,699	0,631
*ofp13+1*_homo	F6	3	E1	3,528	2,111	1,385	1,671	0,656
Wt	F1	1	E1	3,032	2,516	1,923	1,205	0,764
Wt	F1	2	E1	2,897	2,410	1,917	1,202	0,795
Wt	F1	3	E1	2,978	2,522	2,011	1,181	0,797

Wt	F2	1	E1	2,113	1,232	0,988	1,715	0,802
Wt	F2	2	E1	2,479	1,474	1,210	1,682	0,821
Wt	F2	3	E1	2,216	1,504	1,101	1,473	0,732
Wt	F3	1	E1	2,826	2,408	0,990	1,174	0,411
Wt	F3	2	E1	3,010	2,538	1,500	1,186	0,591
Wt	F3	3	E1	2,814	2,416	1,010	1,165	0,418
Wt	F4	1	E1	2,660	1,971	1,262	1,350	0,640
Wt	F4	2	E1	2,718	2,050	1,293	1,326	0,631
Wt	F4	3	E1	2,742	2,020	1,249	1,357	0,618
Wt	F5	1	E1	3,047	2,031	1,350	1,500	0,665
Wt	F5	2	E1	2,856	1,962	1,443	1,456	0,735
Wt	F5	3	E1	2,940	2,089	1,446	1,407	0,692
Wt	F6	1	E1	3,202	2,558	1,489	1,252	0,582
Wt	F6	2	E1	3,291	2,534	1,767	1,299	0,697
Wt	F6	3	E1	3,247	2,505	1,762	1,296	0,703
*ofp13+1*_homo	F1	1	E2-E3	3,543	2,067	1,173	1,714	0,567
*ofp13+1*_homo	F1	2	E2-E3	3,926	2,252	1,428	1,743	0,634
*ofp13+1*_homo	F1	3	E2-E3	3,651	2,158	1,342	1,692	0,622
*ofp13+1*_homo	F2	1	E2-E3	2,586	1,348	1,081	1,918	0,802
*ofp13+1*_homo	F2	2	E2-E3	2,681	1,151	1,190	2,329	1,034
*ofp13+1*_homo	F2	3	E2-E3	2,596	1,245	1,171	2,085	0,941
*ofp13+1*_homo	F4	1	E2-E3	4,274	2,510	1,692	1,703	0,674
*ofp13+1*_homo	F4	2	E2-E3	4,268	2,527	1,802	1,689	0,713
*ofp13+1*_homo	F4	3	E2-E3	4,408	2,577	1,796	1,711	0,697
*ofp13+1*_homo	F6	1	E2-E3	3,663	2,303	1,894	1,591	0,822
*ofp13+1*_homo	F6	2	E2-E3	3,030	2,014	1,633	1,504	0,811
*ofp13+1*_homo	F6	3	E2-E3	3,438	2,255	1,863	1,525	0,826
*ofp13+1*_homo	F7	1	E2-E3	3,165	1,949	1,581	1,624	0,811
*ofp13+1*_homo	F7	2	E2-E3	3,527	2,097	1,688	1,682	0,805
*ofp13+1*_homo	F7	3	E2-E3	3,279	1,905	1,534	1,721	0,805
*ofp13+1*_homo	F8	1	E2-E3	4,326	2,169	1,438	1,994	0,663
*ofp13+1*_homo	F8	2	E2-E3	3,908	1,833	1,312	2,132	0,716
*ofp13+1*_homo	F8	3	E2-E3	4,082	1,990	1,351	2,051	0,679
*ofp13+1*_homo	F9	1	E2-E3	5,041	2,867	2,003	1,758	0,699
*ofp13+1*_homo	F9	2	E2-E3	4,962	2,514	1,934	1,974	0,769
*ofp13+1*_homo	F9	3	E2-E3	5,158	2,681	2,119	1,924	0,790
Wt	F1	1	E2-E3	2,914	1,699	1,291	1,715	0,760
Wt	F1	2	E2-E3	3,100	1,754	1,335	1,767	0,761
Wt	F1	3	E2-E3	3,281	1,810	1,404	1,813	0,776
Wt	F2	1	E2-E3	3,515	2,026	1,327	1,735	0,655
Wt	F2	2	E2-E3	3,688	2,263	1,566	1,630	0,692
Wt	F2	3	E2-E3	3,538	2,119	1,339	1,670	0,632
Wt	F3	1	E2-E3	3,612	2,758	1,569	1,310	0,569
Wt	F3	2	E2-E3	3,402	2,099	1,345	1,621	0,641
Wt	F3	3	E2-E3	3,024	2,126	1,318	1,422	0,620
Wt	F4	1	E2-E3	4,057	2,891	1,612	1,403	0,558
Wt	F4	2	E2-E3	3,935	2,830	1,555	1,390	0,549
Wt	F4	3	E2-E3	4,012	2,750	1,615	1,459	0,587
Wt	F5	1	E2-E3	4,123	2,577	1,561	1,600	0,606

Wt	F5	2	E2-E3	3,792	2,461	1,468	1,541	0,597
Wt	F5	3	E2-E3	3,960	2,587	1,642	1,531	0,635
Wt	F6	1	E2-E3	3,247	2,090	1,563	1,554	0,748
Wt	F6	2	E2-E3	3,206	1,958	1,559	1,637	0,796
Wt	F6	3	E2-E3	3,236	2,039	1,502	1,587	0,737
*ofp13+1*_homo	F1	1	E4	4,380	2,753	2,030	1,591	0,737
*ofp13+1*_homo	F1	2	E4	4,001	2,929	2,249	1,366	0,768
*ofp13+1*_homo	F1	3	E4	3,949	2,688	1,917	1,469	0,713
*ofp13+1*_homo	F2	1	E4	4,126	2,126	1,780	1,941	0,837
*ofp13+1*_homo	F2	2	E4	4,309	2,189	1,788	1,968	0,817
*ofp13+1*_homo	F2	3	E4	4,248	2,134	1,685	1,991	0,790
*ofp13+1*_homo	F3	1	E4	4,920	2,602	1,881	1,891	0,723
*ofp13+1*_homo	F3	2	E4	4,716	2,471	1,853	1,909	0,750
*ofp13+1*_homo	F3	3	E4	3,902	2,233	1,356	1,747	0,607
Wt	F1	1	E4	5,563	3,162	2,156	1,759	0,682
Wt	F1	2	E4	5,151	3,137	2,248	1,642	0,717
Wt	F1	3	E4	5,010	3,195	2,098	1,568	0,657
Wt	F2	1	E4	4,089	3,102	1,999	1,318	0,644
Wt	F2	2	E4	4,595	3,204	2,136	1,434	0,667
Wt	F2	3	E4	4,563	3,337	2,154	1,367	0,645
Wt	F3	1	E4	3,681	2,392	1,329	1,539	0,556
Wt	F3	2	E4	4,533	2,585	1,677	1,754	0,649
Wt	F3	3	E4	4,212	2,560	1,646	1,645	0,643
Wt	F4	1	E4	4,443	2,987	2,004	1,487	0,671
Wt	F4	2	E4	4,336	2,981	1,925	1,455	0,646
Wt	F4	3	E4	4,280	3,113	2,069	1,375	0,665
Wt	F5	1	E4	4,230	3,314	1,852	1,276	0,559
Wt	F5	2	E4	4,515	3,389	2,069	1,332	0,611
Wt	F5	3	E4	4,234	3,000	1,636	1,411	0,545

File S9D. Ovary cells phenotype raw data.

Line	Stage	Flower	Distance X axis (mm)	Distance Y axis (mm)	Nº cells X axis	Nº cells Y axis	Cell size X axis (distance/nº cells) (µm)	Cell size Y axis (distance/nº cells) (µm)	Cell shape (Y/X)
*ofp13+1*_homo	E1	F1	2,24	0,75	83	29	26,99	25,90	0,96
*ofp13+1*_homo	E1	F2	2,73	1,13	95	40	28,74	28,15	0,98
*ofp13+1*_homo	E1	F3	1,03	1,03	53	34	19,43	30,41	1,56
*ofp13+1*_homo	E1	F4	1,91	0,76	78	33	24,53	23,00	0,94
*ofp13+1*_homo	E1	F5	1,68	0,65	101	26	16,62	25,04	1,51
*ofp13+1*_homo	E1	F6	1,29	0,68	71	28	18,13	24,18	1,33
Wt	E1	F1	2,00	0,68	123	35	16,26	19,40	1,19
Wt	E1	F2	1,04	0,70	99	42	10,51	16,71	1,59
Wt	E1	F3	2,59	0,80	96	37	26,98	21,51	0,80
Wt	E1	F4	1,77	0,83	83	40	21,28	20,70	0,97
Wt	E1	F5	1,56	0,74	101	46	15,42	16,11	1,04

File S10. Microscopy images from the distal area of flowers from edited (A) and wild-type (B) plants. Red lines length equal 0.1 mm.

(A)

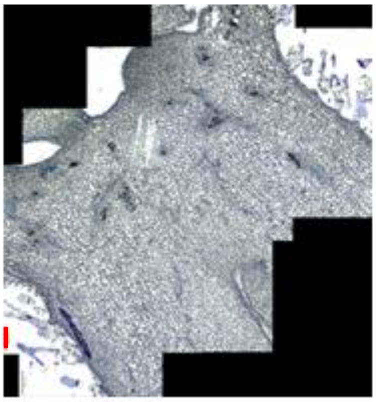

ofp13+1_homo F1

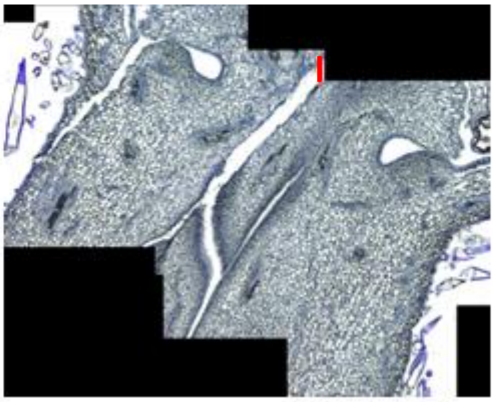

ofp13+1_homo F2

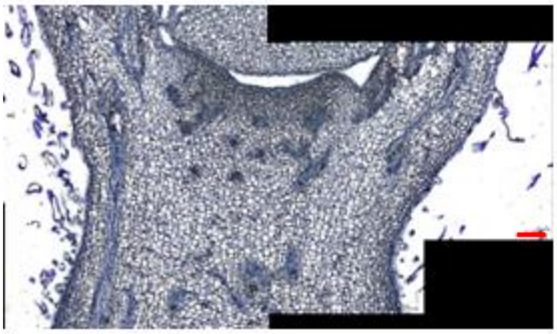

ofp13+1_homo F3

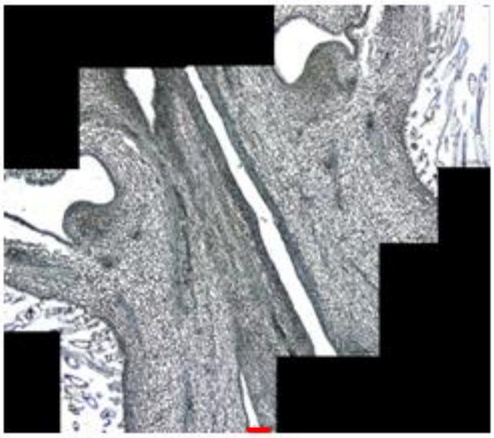

ofp13+1_homo F4

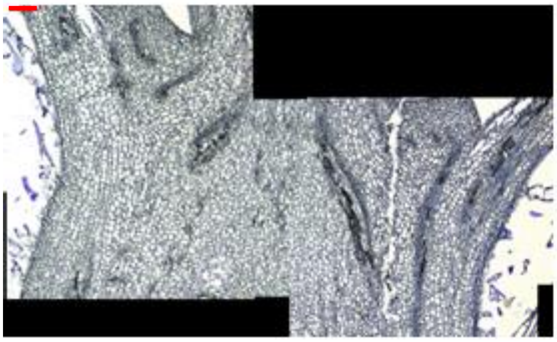

ofp13+1_homo F5

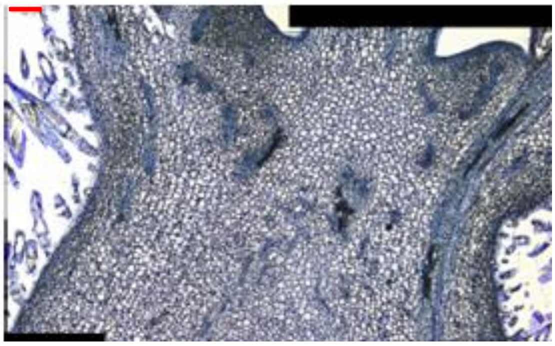

ofp13+1_homo F6

(B)

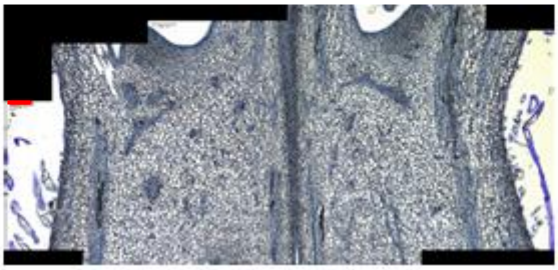

Wt (VED) F1

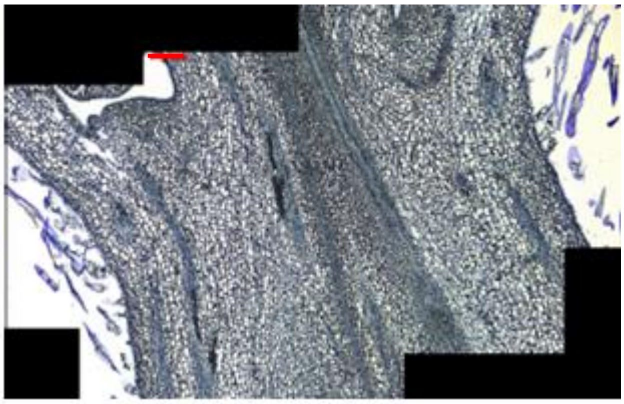

Wt (VED) F2

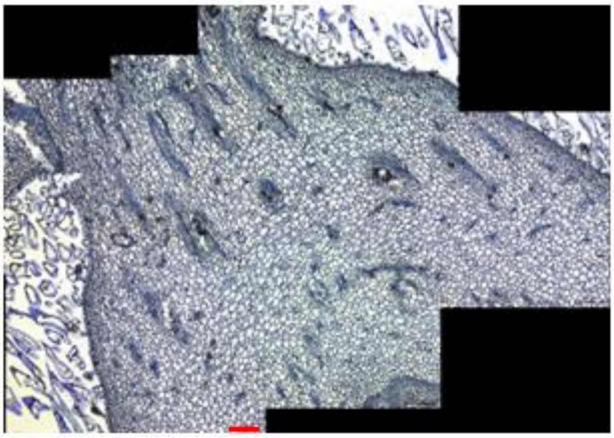

Wt (VED) F3

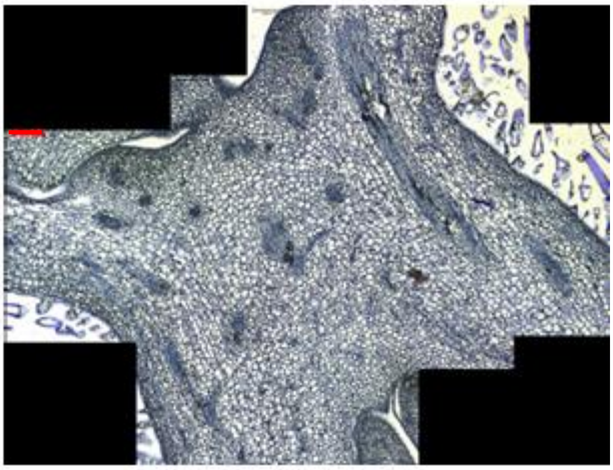

Wt (VED) F4

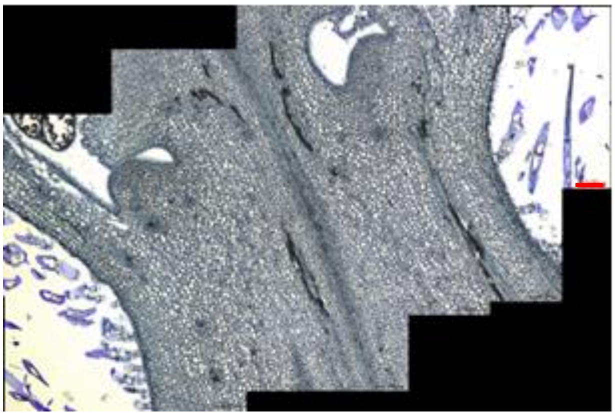

Wt (VED) F5

Supplementary File S11. Expression of CmOFP13, CmOFP4 and CmOFP2. (A) Expression in ‘Harukei-3’ from MelonetDB. (B) Expression from RNAseq in PS and VED fruits (Santo Domingo et al., 2024).

(A)

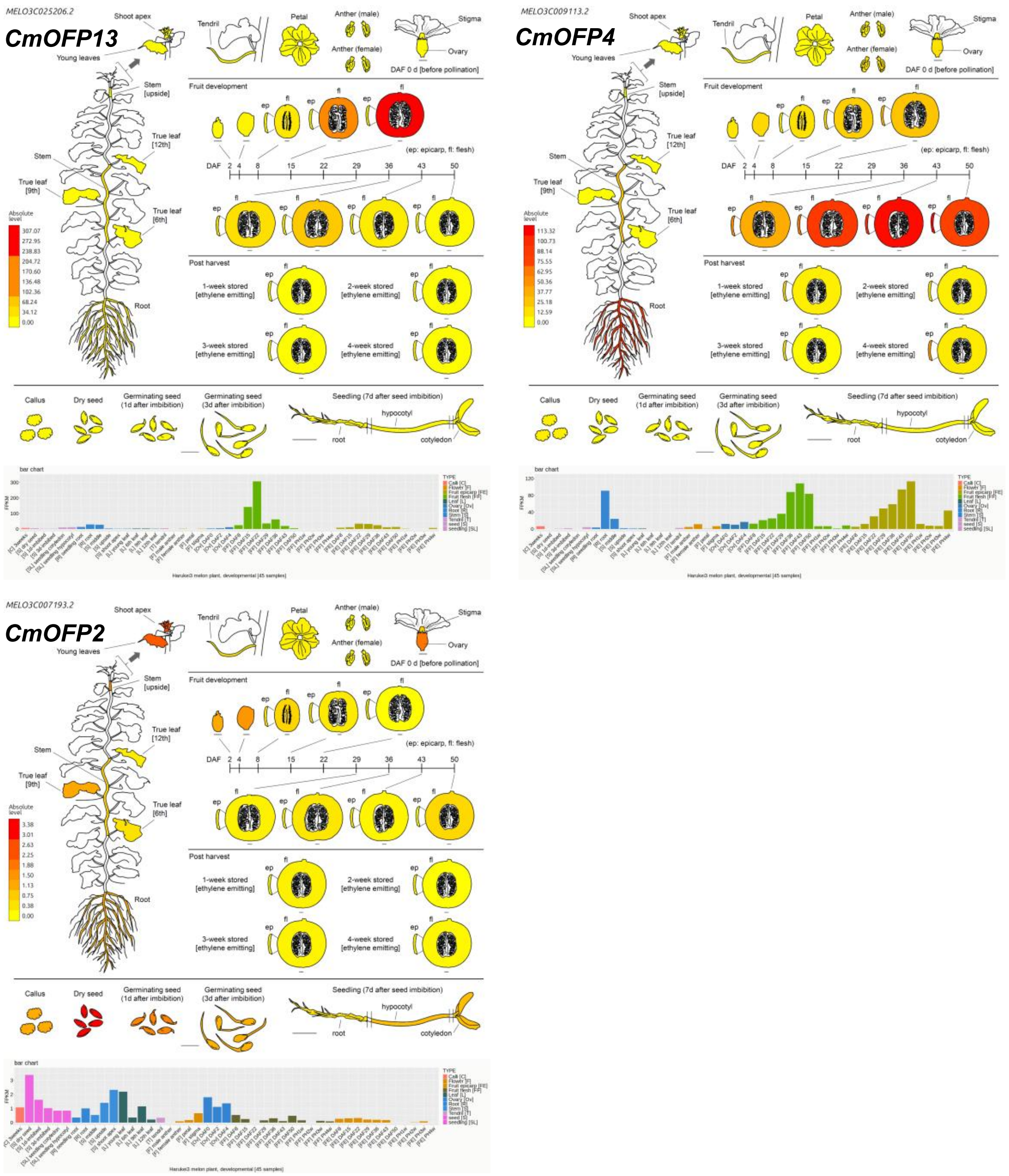

(B)

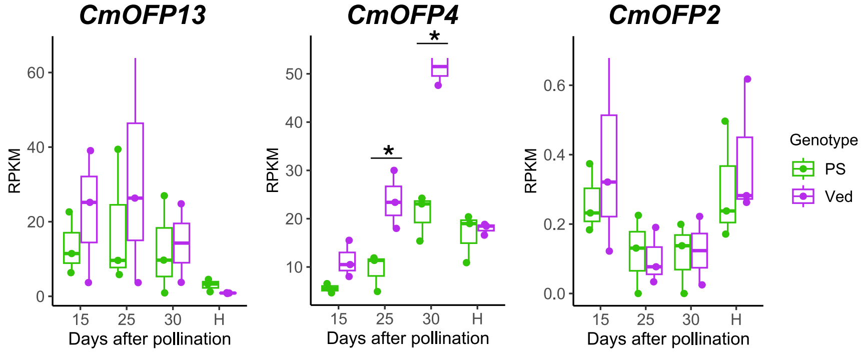

Supplement: Supplementary file 1 — File S1: ppl70641‐sup‐0001‐FileS1.pdf. [file PPL-177-e70641-s006.pdf]
