# Supplementary figures and images for: Targeted Editing and Phenotypic Profiling of CmOFP13 Mutants Reveal Its Role in Melon Fruit Morphogenesis
Source: Physiol Plant. 2025 Nov 29;177(6):e70641. doi: 10.1111/ppl.70641 (PMC12664293; doi:10.1111/ppl.70641)

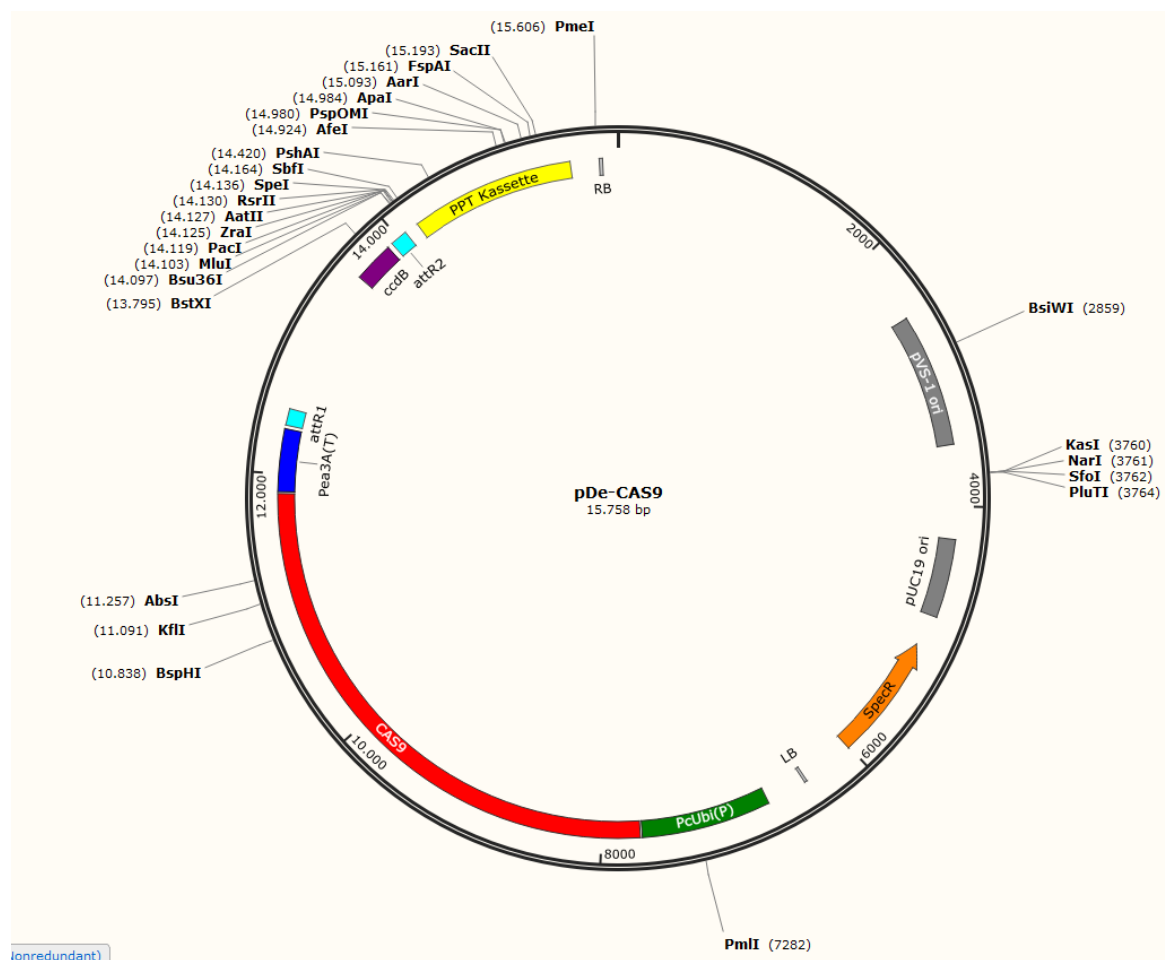

**Supplementary File S2. pDe-CAS9 map**

Supplement: Supplementary file 2 — File S2: ppl70641‐sup‐0002‐FileS2.pdf. [file PPL-177-e70641-s004.pdf]
