# Supplementary material for: Targeted Editing and Phenotypic Profiling of CmOFP13 Mutants Reveal Its Role in Melon Fruit Morphogenesis
Source: Physiol Plant. 2025 Nov 29;177(6):e70641. doi: 10.1111/ppl.70641 (PMC12664293; doi:10.1111/ppl.70641)
Supplement: Supplementary file 3 — File S3: ppl70641‐sup‐0003‐FileS3.docx. [file PPL-177-e70641-s007.docx]

**Targeted editing and phenotypic profiling of *CmOFP13* mutants reveal its role in melon fruit morphogenesis**

Carlos MAYOBRE, María José GONZALO, Montserrat VERGÉS, Guillem GUARDIA-BERSABÉ, Dídac JIMÉNEZ-SÁNCHEZ, Antonio Jose MONFORTE, Jordi GARCIA-MAS, Marta PUJOL

**Supplementary File S3.** OFP DNA and protein sequences

>*CmOFP13* (ATG and STOP codon in red, Kozak sequence is highlighted in yellow)

TCCG**ATG**AGAAATCACAAGTTCCGTTTCTCCGACATGATACCCAACGCCTGGTTTTACAAACTCAAAGAAATTGGCGGCGCCTCCAGACCAAAATCTTTCCGTTCCAACAAAAACCCTCACCACCCACCTCCACCTCCCCCGCCCTCCAAACACAAACAACCACCCCCTCCTCCTCCCCACTCTCGTTCCAGAAAATCTTACTATTTCACTAGACAACTCGAATCCAACGATGCCTACTTCGTCAATTCCCCTCCACCGTCGCCTCCGCTTCTACCGGTACCAATCCCCCCGAGAAAGTCAACAAAACAACTCAAACCAGGAAGAAAACAAACGAGTTCCCGGTCCTCCGCCAAGCTCCTCAGCTCCTCCTCCGTCGGCTGCAGCTGCCACACAACGGCGGAATCTATCTGGACAAAATCCGATTCTCCTCCAGAATTCTCCACCTCACCCTCCGACACCTCCCCTGATTTCCGAACTGACAAAATCCTCACTGCCGAAGCATCCAAACACTTCGAGCACGACATCGTAATCGACGTATCGTCGAATTACTCCAACAATGCCGTCATCGGCGCCTTTGACGAACTGGAACTCCCGCCGATCATCACGAAACAGAGGAAGAAAACAGAGACAAAACAGAGAACGACGACGACAACGACGGCAGGAACAAAGAAAGTTGCGGGGAATTCCCCGGGCGTACGGCTGCGGATTCACTCCCCGAAAATTGGGTACCGGAAAATGGGAGGGAGGAAAAGCGTTTCGTCACGGCGGAGCTTGTCGGAGAGTTTAGCGATAATGAAATCATCGTACGATCCACAAAAGGACTTCAGAGAATCAATGGTGGAGATGATTGTTGAGAATAACATTAGGGGTTCGAAAGAATTGGAAGATCTTCTTGCATGTTATCTGTGTTTGAACGCCGATGAATATCATGATCTTATTATCAAAGTTTTTAAGCAGATCTGGTTTGATCTTACGCAACCTTCTCCTCCACCTCTT**TGA**TTTTCTTTCGTCTTTTCTCTTTCTTTCTCCCCCATTTTGTAATTCTCTTTTGTACAGTTCTTCTTCTCTAATTTCTAAACATAATCTTAATAAAATTTTTACTTTCATTCACTTTTTAGTTT

>*OFP13+1v1* (ATG, mutation and STOP codon in red, Kozak sequence is highlighted in yellow)

TCCG**ATG**AGAAATCACAAGTTCCGTTTCTCCGACATGATACCCAACGCCTGGTTTTACAAACTCAAAGAAATTGGCGGCGCCTCCAGACCAAAATCTTTCCGTTCCAACAAAAACCCTCACCACCCACCTCCACCTCCCCCGCCCTCCAAACACAAACAACCACCCCCTCCTCCTCCCCACTCTCGTTCCAGAAAATCTTACTATTTCACTAGACAACTCGAATCCAACGATGCCTACTTCGTCAATTCCCCTCCACCGTCGCCTCCGCTTCTACCGGTACCAATCCCCCCGAGAAAGTCAACAAAACAACTCAAACCAGGAAGAAAACAAACGAGTTCCCGGTCCTCCGCCAAGCTCCTCAGCTCCTCCTCCGTCGGCTGCAGCTGCCACACAACGGCGGAATCTATCTGGACAAAATCCGATTCTCCTCCAGAATTCTCCACCTCACCCTCCGACACCT**C**CCCC**TGA**TTTCCGAACTGACAAAATCCTCACTGCCGAAGCATCCAAACACTTCGAGCACGACATCGTAATCGACGTATCGTCGAATTACTCCAACAATGCCGTCATCGGCGCCTTTGACGAACTGGAACTCCCGCCGATCATCACGAAACAGAGGAAGAAAACAGAGACAAAACAGAGAACGACGACGACAACGACGGCAGGAACAAAGAAAGTTGCGGGGAATTCCCCGGGCGTACGGCTGCGGATTCACTCCCCGAAAATTGGGTACCGGAAAATGGGAGGGAGGAAAAGCGTTTCGTCACGGCGGAGCTTGTCGGAGAGTTTAGCGATAATGAAATCATCGTACGATCCACAAAAGGACTTCAGAGAATCAATGGTGGAGATGATTGTTGAGAATAACATTAGGGGTTCGAAAGAATTGGAAGATCTTCTTGCATGTTATCTGTGTTTGAACGCCGATGAATATCATGATCTTATTATCAAAGTTTTTAAGCAGATCTGGTTTGATCTTACGCAACCTTCTCCTCCACCTCTTTGATTTTCTTTCGTCTTTTCTCTTTCTTTCTCCCCCATTTTGTAATTCTCTTTTGTACAGTTCTTCTTCTCTAATTTCTAAACATAATCTTAATAAAATTTTTACTTTCATTCACTTTTTAGTTT

**OFP protein sequences in melon, Arabidopsis, tomato and rice, plus validated OFP proteins from peach, cucumber, potato and pepper**

>MELO3C006531_CmOFP1

MSSLKKSNFHLWFSKLRCFPATVKPSSPPKTPNKKPSPITHLENSDYSSTSTAADDGFFSDESTSDSDAIVPDFSAAVASHRFFFSSPGCSNSIFDSSPDTHHSAAVSAAVHGGVEVRKVSMDPFVDFRASMQEMVEARDRPVDVRRDWEYLQDLLLCYLRINPVDTHKFILRAFSDLVVYLLECSPESFSDRRLRPHNINSNSW

> MELO3C007193_CmOFP2

MKWGRRKPHNPSSSSSTSSSSSSRPSFMSNILPASWLSKLKQKKSNHEARPRKVKGTEKGNSPCIQSPDYANVTPSPGQVNGNRNRLFTGDNGEFWKLSFGGDDIDVKKSSGILRSVWYNSEDEHDLPRTSCRSCRTKCTESEGKEEIQNLDDMVSRMTRRRRRRREAPTQVKLLRRESETESRTPRRKYRENGNFGYLGKKSMEKKGFKPERETDKGKERARRLVGKKMLGVEEESGVRKNERDKTNLTINSRKHRYVPSTMSKSSNLGTIEENCVFSSMKAEESDGHDTVGIEIDSDWERMKELKIEELKLRYEKQRQPLYIRKDSNEKNPKGRRKIRVYSPRTANKIEICKIKALEDMKKAKLKMKKKVRESMVDDETDLESFAVVKSSFDPQQDFRDSMVEMIMERRISKAEELEELLACYLTLNSDQYHDLIIKVFRQVWFDLNQAALESELHKQFPCNEQLV

> MELO3C007422_CmOFP3

MIIFGLQAPFKAVRMKAAGSTQHHHQAQNAYRALCCGCSCNCRLSFSSSEETESFNSDKFPSVSSIAHAMVQERLDQMIREKREVRNGKERKKQRSEDTKFVVMVAMEKCSDDPKEDFRVSMTEMILANRIEEPKDLRNLLNYYISMNSDECHGVIFEVFHEVCSNLFLACKRHYW

> MELO3C009113_CmOFP4

MVGDRVEWSRERNNHFWRMGNYRFRVSDMMPNSWFYKLKDMTTIIRRRNKNKDQPSKNTHTTTDLAYSHPRKSIHFTASQLAANSPPEPPRRSSKGKKPRRRPTSSSAAAPTSTLLLTSSSGCSCGRTALQSVATTSTTPPPILTHHSYLHAEKEEEDANAVISGKAHKVSPKKINGSDEEYLKSLPQIIDQLPPIITRSSSSSSNAADASTCPSISIAKNDKSEPIRSSPSRRFLLNSPGPKLRIVNSPRVSSSKRFGHVGRRKSGKRSLNDSLAIVKSTEDPQRDFRESMMEMIVENKISGSSELEDLLACYLSLNTDEYHDIIVKVFKQIWFDMTDIIGVHY

> MELO3C009514_CmOFP5

MLTKKKKMMMMMRLPSLFKILAIDDKSTFPWPSCRQPRTLSFRTTSAAVATATDSSGSFFTLSSESSGSLSTVSESSGGDPIERMIRDLRSTKRLHFEPTGKSSSIVEDETVSHPLKEGTTVMSMDSNDPYSDFRKSMEEMVEAHGMKDWESLEELLNWYLRVNGKKNHGFILGAFVDLLVSLAMASSSSSSSSSSSSLCCYSSSSSSSSLPCVSSSMEIEEISSLDEHHHHVYS

> MELO3C012340_CmOFP6

MANTKKKHHLIKNFPSIFKSKSPTSSHPWEWDWPSCKHPKTLSFRSQNDLVFKTVNSIFFDQPFETITTTTTTTPDYSSSLHTNSSDSVSATNSTPAMDSEESLETVVRGARSERLFFEPDDTSSILEKSKPIESVETDELPRSGFKESLIVSIESENPYEDFRKSMGEMVESHGVKDWDGLEELLGWYLKANWKNNHRFIIGAFVDLLIHILLASSSSSSSTSTSSSSSSSLCSNSDSNYYTCTESSSSRSCSSSLRSLNPSSIRKELDHEEEDDHDHITETL

> MELO3C015818_CmOFP7

MPTKLGRNYLNLCFTKIKNPLSATQSSPPITHTPDRRRQTTRSFSSTAAAFITNYNSLYEITTTTNSDSNSPLFGLANDIGVADPDAYVAVDFITAFTSHRFFFSSPGSSNSIIESTTPTTTESTTTMSLSSEYSARYEGNDDELMIFNNSHVIPTYSPDPYMDFRRSMQEMVEAREKMTTAATTTTMKKSSWEFLHELLLCYLALNPKATHKHILKAFADVATVIKPPLAMKETEEEGNNVDRESESMVDDGGPGGGCEISG

> MELO3C017554_CmOFP8

MMTPKRFKLLRIPSFHCCRSNDISVVPTDPPSPSPPSKPQHSSLRRHVSSAFRTAACGCRSSSTKSDDDQIPKSTPTLPTHVPPTPLLHSYDDGSTFPKRRRRRRNKNKSKSTTLTRLRTSTSSTESGLFSSESFDEIDELEETETLISSSKTISTSDDDNDSSSEFNPQLETIREKPNKIKLRRKKEKEKRRRKQKRTTIIKTSPSPEIESPARLSVFQRLIPCTVEGKIRESFAVVKKSADPFEDFKRSMVEMIMEKEMFEEKDLEQLLHCLLSLNDREHHGIIVEAFAEIWQSLFCNN

> MELO3C019910_CmOFP9

MGKKMKLLPSLFKNRQSPDRPWQWPLCGTSKTPSFRAPHHHQIFTTLNSTFFHNFLSDPIHTPDSWFANSSLFESARVSLSTEFEDDLELVIQGAKSERLIFEPGETNSILDQSRGGKSEGGKCESILRFEGSVVVLMAMESEDPYLDFRRSMEEMVECHGIRNWEWLEELLNWYLRMNGMKNHGYILGAFVDLLVDLGGADGSTDSTSIFSDDLIIQPHDRERCVV

> MELO3C024232_CmOFP10

MLLSFFCIPLLFLYKNQYYHITLSTQNQRKLNKMPKKLQKSLQDYLSKIKKPTPQLQFPNPQTFSSSKSWILAGCKHPKTLSFAIDRKQVDAVGNKEDAAATLADIDRFLFENFRSLYLKEDGDCGDRKVVGGGGGGRDCKNHRGVVSPESPVDSYGGSHRFFFSPDLSGSDLPDDSHTESSENAGSSSSSLIGEDRGKDLKLPSDCIAILRKSPNPSEEFRRSMQEMMDGHLKHHEKVDWEFMEELLFCYLNLNDKKSYKYILNAFVDLIVILRQKAEEEPAKPRTVRSVRMVRRMI

> MELO3C024574_CmOFP12

MAAPRRNLQPTSLSVDLNICRPKLLSHLFHHLKPKPSLKSPNHHHRFSSASSDSESESETRTSITFRGFGRSGGESVAVEKDSDDPYLDFRHSMVQMILENEIYSKEDLRGLLRCFLQLNSPSHHGIIVRAFSEIWDSVFSATSPILRF

> MELO3C025206_CmOFP13

MRNHKFRFSDMIPNAWFYKLKEIGGASRPKSFRSNKNPHHPPPPPPPSKHKQPPPPPPHSRSRKSYYFTRQLESNDAYFVNSPPPSPPLLPVPIPPRKSTKQLKPGRKQTSSRSSAKLLSSSSVGCSCHTTAESIWTKSDSPPEFSTSPSDTSPDFRTDKILTAEASKHFEHDIVIDVSSNYSNNAVIGAFDELELPPIITKQRKKTETKQRTTTTTTAGTKKVAGNSPGVRLRIHSPKIGYRKMGGRKSVSSRRSLSESLAIMKSSYDPQKDFRESMVEMIVENNIRGSKELEDLLACYLCLNADEYHDLIIKVFKQIWFDLTQPSPPPL

> MELO3C025206_CmOFP13+1

MRNHKFRFSDMIPNAWFYKLKEIGGASRPKSFRSNKNPHHPPPPPPPSKHKQPPPPPPHSRSRKSYYFTRQLESNDAYFVNSPPPSPPLLPVPIPPRKSTKQLKPGRKQTSSRSSAKLLSSSSVGCSCHTTAESIWTKSDSPPEFSTSPSDTSP

> MELO3C025343_CmOFP14

MDTRFKLRLSRVFQSSFASCRSRNLSDILHKAVFIPSSSDDASFRKISPSETSSDFLLPRRKISRRFPLSPLPSAISGGRTCPPTSPISPSKPISEKMTTSTKKKNKHKKQRKQRKQSKREIPFSLFSSSNFGGTWWYSSEDEDDDDETDTLFSSKSRSSDSSKHLTDDRNSFAVVKKSSDPYNDFRMSMLEMIVEKQIFSAKDLEQLLQCFLSLNSHHHHNVILEVFTEIWEALFSDWGS

> MELO3C025581_CmOFP15

MSNIRKNKFFTTIFSSTAGCGRCQKPKLSDIVQPNKKPPTTIVRRSSSSSSTDHNGTFSLDEDYSSASKSTGSQSPVAILICDSIAVEKDSDDPYEDFRRSMVQMIVEKRIYSPNGLQELLNCFLHLNSPYHHEIILKAFTQISNEFESSHRLWNISNDNEWKPRSAGEG

> MELO3C026874_CmOFP16

MKVEVGLVSFKSKLSKPCSKLLHLFKFPMKKPFSIKSLWTRHPRRNSRAISKPRRRTWSWLRWLRRVGKMERVRDHLRSSESVRSDNECREKLLFPSPMIRGRKVAAGTSWEEKEEVEDACKSFENYLVEMIIEEGKVRDLMDVEELLYCWRNLKCPVFVDLVSRFYGELCKDLFSSHIQAFTPNFQPK

> MELO3C010932_CmOPF18

MARSFKLRVRLFISSFRFCRPKHLRALTFPKTSSPENFHYKKQIPTNPKFCSCYSNPPSPPPSTPVDLFADFPKEKSTSICNSCKLKSFARKNGLLQGKENRAETEHGITNEENERSALFSWITRKGSKIKKKLKKTGLKSKPFKANGYREEESEGTEALVNSSMSFSDDVSPVKRSKRATCLRKLEGKMGKSFVQVKRSKEPQEDFKRSMVQMILEKEIFETKGLEELLQCYLTLNSPEYHRIIVGAFSEVWEFLFCDSHSNKAVQCD

> MELO3C009515_CmOFP19

MAAVKKKLLINTISVDIGCGSCRKSKSIISQIFRPKPKSPTSYSDRRLFRSLSSSSEKKLSDSDMAYAPEVVGGGGFWKIGGVSVAVEKDSNDPYVDFRQSMLQMILENEIYTQEGLRELLSCFLHLNSPCNHGIIIRAFAEIWDGVFCARSAAPAKQRRHVRSRAF

> MELO3C004557_CmOFP20

MEKRFKLRFSRLFQSSFFSCRSKKNSDILITQKPISKPLISPKPPQLSSNYTPKLPLNPSHFQFPPPPASSPIISPPLSHLNDCLSRHKSKTKIKRRKNPPSRPAPPPPPRLPEEDFGGAWWYGGHDDTEDETETLFSSRSLTSDSSVSRRRHRQRHGRRRPPERKMRDGFFAVVKNSSDPYKDFKASMAEMVVEKKIFGGKELEELLQCFISLNSRHYHKVIFEVYSEIKEALFFL

> MELO3C024573_CmOFP21

MANKLKKLPNFLNKSPWLWRSCTQSRTLSFRHPNDIFRTINSAYDEEEEDFYDYDDEDDEKETTQDDMSNDEDQIEALVRGLRVRQGKRLFLELDETNSIMTATATTTVAVATVGAGNYHVPFKESVAMAMESKDPYLDFKKSMEEMVEAHELKDWKGMERLLSWYLKANGNANHEFIIGAFVDLLVDLAFAASSNLSNNSSSSPSSSSSSTTTTSSLLCSSSSSIFPNSSSCSSCSSFRAPNSIISSNSVETADELECSCSSSSIRVAPPCLSSLFEDDEEEDIEEGF

> AT5G01840_AtOFP1

MGNNYRFKLSELIPNAWFYKLRDMSKSKKKNLQSQPNSTTSKKKHHAVSTPTSTTPLSPRPPRRPSHSSK

APPSHPPRKSSGNRLRHRATVDSKSSTTSGDSTTTETGSFSPDFRSDQVLLPDESLTGSWHSPCSSKLSK

TATFTPPPELELRPIITKTAATARKTAVNSPAGVRLRMRSPRISVSSSARRSGSSARRSRAVVKASVDPK

RDFKESMEEMIAENKIRATKDLEELLACYLCLNSDEYHAIIINVFKQIWLDLNLPPPHSK

> AT2G30400_AtOFP2

MGNYKFRISEMLPNAWFHKLKDVTKHSKPKNKASSSSSNTCSKKKPSSDSLPQHSYFSNSLVANNPPHHN

SPRNSLHTKKMSKRKTLYKPSLKPLTPPPLLVSASFNKSKINDQDSSYSLFPAIETSPESFVYSFYEEDD

DDEFVEFSNFKINTKNKAFTKQKVKVIDSVEKACTASKPIKKPQKSHLSVKISRDEDDDEYKAEKKYQRQ

VSSGRKPSAGINLKRVNSPRIQLSGTRRSTSRRSESKQDVLESFAVMKRSVDPKKDFRESMIEMIEENNI

RASKDLEDLLACYLTLNPKEYHDLIIHVFEQIWLQLTKTK

> AT5G58360_AtOFP3

MKQKMGTHKFRFSDMMPHSWLYKLKGMSRSSRKHQLSSPKHLSSADASSSRKLRDPLRRLSSTAHHPQAS

NSPPKSSSFKRKIKRKTVYKPSSRLKLSTSSSLNHRSKSSSSANAISDSAVGSFLDRVSSPSDQNFVHDP

EPHSSIDIKDELSVRKLDDVPEDPSVSPNLSPETAKEPPFEMMTQQKLKKPKAHSSGIKIPTKIVRKKKK

ERTSQVSKKKGVVKSFAIVLSSVDPEKDFRESMVEMIMENKMREQKDLEDLLACYLSLNSSEYHDVIIKA

FENTWLHLTQGLSISL

> AT1G06920_AtOFP4

MSPSEWFHKLKNMTKPRKKHSLPLYSINTTKKRKPSSESKSLPYSSTSYFFNRSRSRTSFESRILQISPR

NSLHNIQSKRKTVYKPSPPSSSIVSAGFNKTFHQSHDSLSASSNLKVISSEDDIIIDMNNRDFKKKTFKE

ITKFDSTEKACRASNRTKETHIPHHLSVKVSKEKEDEEEDACRTKKKHQKTLVSSGRRSSAKSPRIKLRA

RSPRIQVSPRRSKSRSQNKQILDSFAVIKSSIDPSKDFRESMVEMIAENNIRTSNDMEDLLVCYLTLNPK

EYHDLIIKVFVQVWLEVINSTFASK

>AT4G18830_AtOFP5

MMRWGRKKPVSSSSSSGLSRALPVSWFSKLSGSSDLKPAKEKKQDEKASQNISVKTSLGSTTRRSDIHEN

SKRFQRVSVEKENSATRSADKESNEKFEEIMSSVRKKVRDFQKETCGFLEVEAMDRDNGTVILTPRIQVN

RDKQRCERRDQRLLEQKPKRSEQDAGVKVKKPARRTGTGGYSREDSVILGHTITKPAHQWEKLKEVKLRE

VKLKADQQRKSLYLKRELNRIGTKGNNKVRVFSPRASEKCRVKAIEDLKKAKQRAREHELLIETADGGME

NESFAVVKCSSDPQKDFRDSMIEMIMENGINHPEELKELLVCYLRLNTDEYHDMIISVFQQVHNDFNFH

> AT3G52525_AtOFP6

MATKSKKKILKTVSVVDISCGNCIKPTFASIFNFFSKKPKRPSSTYRHCHSSISSATPSSTPLATASVAV

EKDSDDPYLDFRQSMLQMILENQIYSKDELRELLQCFLSLNSHYHHGIIVRAFSEIWEDVSSAAASAVEA

SPLITRHVSRASRDYYNYY

> AT2G18500_AtOFP7

MTKRFKLKISRILSFKSCRLKDPSSLPFNPVSSSLRRTSPPVNSSADVTTVPQRRRSSFRLHVLTVFGCG

RSSTPLDVDLRNSPVLSPPQTPTFQWESEGKWHVIAQVTEEEYETPRRKIYNGGSEKDNRRRLKKKEKSN

SRRRGSISSAEEETDRESLLPSSTNLSPEYSSSELPRVTRRPRQLLKKAVIEEESESSSPPPSPARLSSF

VQRLMPCTMAAAVMVEGVAVVKRSEDPYEDFKGSMMEMIVEKKMFEVAELEQLLSCFLSLNAKRHHRAIVRAFSEIWVALFSGGSGGGRRSSSFSSVRLSDYDEC

> AT5G19650_AtOFP8

MEKRMKLRVSRIVRSSLSSCRPRDLYDVVETCAVTSQATSSERFFVTKAKTKTPSRPKSHASSCPRASPI

FPPNPFYEESRSFRDLRKKVKTNRKQRSQFGSDPLFASRFKSTGSWYWSCSEEEDEGDKEESEDDDSDTL

FSSRSFSSDSSKAESFAVVKKSKDPYEDFRTSMVEMIVERQIFAPAELQQLLQCFLSLNSRQHHKVIVQV

FLEIYATLFSP

> AT5G22240_AtOFP10

MLNLQAKLNEKKVPLLTNFPTPTSSLNNLNLFSLPLVKKKMSFKKMMKYILKTIFKPIFMACGCGSTVPPSSHSHYTPGPPVSPTVLRSPCPKIDESVAMAKESINPFEDYKKSMNQMIEERYIETESELKELLRCFLDINPSPQHNLIVRAFVDVCSHLQPPHDRRGKSLGRLLRLYVNNPLDNNDDDSHQTSSK

> AT4G14860_AtOFP11

MSNFLRKKLHLCFSSSGGLSPSIPSSPIIVSNHNAQSHPHHTPSIFINNFNSLYDQLSVSSPLHRRHSSENPAGVFSTNRREEEEEDETTTSVSKLLSGGTAIMKHIESPDPYRDFGRSMREMVEARDLTRDVVADREYLHELLFCYLYLNPKHTHRFIVSAFADTLLWLLSPSPSPEHFLS

> AT1G05420_AtOFP12

MPRVMWKNFHLCFPSNLTKPSSSPSGATSDDPNRPSILLINNFNLLYDDSSAAHRRLSKPLIHDVEPSSTFTASTSTAANSSSSSASYDDSDNYGFAPDDDSPPPDLTAVLASRRFFFSSPGCSNSITDSPDLRCRDNYDTATRLLTGGTAVKHYVQSPDPYNDFRRSMQEMIDAVTNAGDLRRYEFLHELLLSYLSLNAADTHKFIIRAFADILVSLLSDGHRIS

> AT5G04820_AtOFP13

MGKKKMKLSSLFKGGAGGLLAVPLCYNAKTLSFRVGDDMIKTVNSVFFDHHHNNNNGGDLLEAETPESWFTNSSETASHSTESDQDLDAESLEMVVRGVVRSERLFFDPGVTSSILEEIEEKSKSDLKSKETVAVGEDRSTPIEEISVAVAMESEDPYGDFRRSMEEMVTSHGELAKDWESLESMLAWYLRMNGRKSHGVIVSAFVDLLSGLSDSGAGITSASVSDSARYSTAVSSLPSSPVYSLSQGQTEIQEEERRSC

> AT1G79960_AtOFP14

MPNPLQKSLHGYLSKIKKETGKLQLSSSHSFSSSKNWVLGKHPKKLSFSFKHRRRSSKTRFSKEEPVYHQDSAHAATLSDIDRFLEENFKSLCIRDDQEDDQHQARVTKNKEKRESSSDDSDDDDDDDDYRHRFERTWGHAVYDSPKQPPDLLRTERLSPPPGSSEGRPSMETTSTSSERQSRSTLVLPENCIAVLRYTDEPQEDFRQSMVEMMESKLGMRESEVDWDLMEELLFCYLDLNDKKSHKFILSAFVDLIIALREKEKRITRKGHVRSLSTRAARDRLRKRMIMSDN

> AT2G36050_AtOFP15

MKLPFLNKNHSTSSYSSNSSSSSWPWPSCNQNPKTLSFRATITFTNPIHDQDDDELDLLDPPEITDSVENVIKGLRSSERLIFESKGETNSILEEATSKREEEDEEEGFMLFSLESDDPYSDFKRSMEEMVEAHALHHDWKSLEKLLLQFLKVNAKTSHRYIFAAFVDLLMNLALDTKKAIINNDISKDDGVSASRAAAAGEASTSCCNSMTLGESPSSPLSFYTSCSSSSSSDETSSMSVRFLPLSSLLEMDEKTKEILV

> AT2G32100_AtOPF16

MPKILWKSLHLCFPSNLTKCYSSPCIPPSSADPDGIIQPNRPSIVLLNNFNLLYHNDNHHHPHRVIDLPSSSTTTTPAATSSSSTSSYESDISPDVSAAFASRRFFFSSPGRSNAITDSPEPRSREFSDNYDDATITSTKKKKKKVYDNSVTTTTTRLISGGTAVTQHVDSPDPLTDFRRSMQEMIDAAIDAGELSRDPNDGYDFLDELLLTYLSLNPADTHKFVIRAFSDILVSLLSEERRIC

> AT2G30395_AtOFP17

MRVKATLINFKSKLSKSCNRFVSLFRFRVKRPVFIRPLRARHGNVKPRHQHHHSKKPICSCLCFLNSSKNHKMSNAKHRSSSFSVNDDDYSKFMQSPLTPATAKKLFTSPITTPYSSRTRKSLNARDTFEDNAVEDACRSFENYLIHLIVEEGKIDDLMDIEELLFCWKNLKSPVFIELVSRFYGELCRDLFSGE

> AT3G52540_AtOFP18

MVRKMKLPFLNKNTSSSSFSSNSSSSSSSWPWPSSHQQNLKTISSKASFIVNKPKDVYEPEPPPRSFSSSPSSSSYSSFSSTSHAIENPPEIESIENVIKGLKSSKRLIFERRGTSNSILEEATKRDDHEEEEDGLMLLSLESNDPYTDFKNSMEKMVEVHVLHHDWISLEKLLFWFLKVNVKASHRYIFAAFVDLVLNLAVGPSKDVAGEPNSDVVVEDSLSSSWPVSLYSSSDENSSTSVRFLPETSIGEKGRDVCCLSSLFELEEKIKDNIDPNDYVSS

>CsaV3_4G027080_CsOFP1a

MRNHKFRFSDMIPNAWFYKLKEIGGASRPKSFRSNKNPHAHPPPPPPPSKHKQQPPPPPPPPHSRSRKSYYFTRQLQSNDAYFINSPPPSPPFYLYQSPRERRKQTSSRSSAKLLSSSSLACSCHTTAESIWTKSDSPPQFSTSPSDTSPDFRTDKILTAEASEHFEHDIVIDVSSNYSNNAVIGAFDELELPPIITKQKKKTETKQRTTTTTTTGTKKVAGNSPGVRLRIHSPKIGYRKMGGRKSVSSRRSLSESLAIMKSSYDPQKDFRESMVEMIVENNIRSSKELEDLLACYLCLNADEYHDLIIKVFKQIWFDLTQPSPPPL

>CsaV3_3G033200_CsOFP1b

MGNYRFRVSDMMPNSWFYKLKDMTTIIRRRNSKKDQSSKNSHTTDLVYSHPRKSIHFTPSQLAANNSPLEPPRRSSKGKKPRRRPTSAAAPTSTLLLTSSSGCSCGRTALESVTTTSTTTPPVLTHHSYLHAEKEEEDPNAVIFGKEHKISPKKINGSDEEYLKSLPQIIDQLPPIITRSSSSSSNAADASTCPSLTITKNDKSEPIRSSPSRRFLLNSPGPKLRIVNSPRVSSSKRFSHVSRRRSGKRSLNDSLAIVKSTKDPQRDFRESMVEMIVENKISGSNELEDLLACYLSLNTDEYHDIIVKVFKQIWFDMTDIIGDHY

>CsaV3_6G051110_CsOFP5a

MKWGRRKPHNPSSSSSTSSSSSSRPSFMSNILPASWLSKLKQKKSNQEARPRKVKGTEKRSSPCIQSPDFANVTPSPGQVNGNRNRLCTGDNGEFWKLPFGGEDIDVKKSSEILRSVWYNSENEHDLPRTSCRSCRTKYTEFEGNEEIQNLDDMVSRMTRRRRRRREAPIQVKLLRRESETESTTPRSKYRENGNFGNFGKKGVEKKGFKPERETDKGKEIRARRLVGKKMLGVEEESGVRKNERDKTKLTNSRKHRYVPSTMSKSSNLGTIEENCVFSSMKAEESDGHDTLGIEIDSDWERMKELKIEELKLRYEKQRQPLYIRKDSNEKNPKGRRKIRVYSPRTANKIEICKIKALEDMKKAKLKMKKKVKESTVEDDTDLESFAVVKSSFDPQQDFRDSMVEMIMERRISKAEELEELLACYLTLNSDQYHDLIIKVFRQVWFDLNQAALESELHKQFPCNEQLV

>CsaV3_6G048660_CsOFP5b

MKAAGSTQHHHQEQSAYRALCCGCSCNCRLSFSSSEETESFNSDKFPSVSSIAHAMVQERLEQMIREKREVRNGKERRKQRSEDTKFVVMVAMEKCSDDPKEDFRVSMTEMILANRIEEPKDLRNLLNYYISMNSDECHGVIFEVFHEKEVSKNPRDDRILKSKLEFD

>CsaV3_6G040960_CsOFP6-19a

MAAPRRNLQLTSLSVDLNICRPKLLSHFFHHLKPKPSPKSPNHHHHRFSSASSDSESPPFSDSDSETRTSITFRGFGRSGGESVAVEKDSDDPYLDFRHSMVQMILENEIYSKEDLRGLLRCFLQLNSPSHHGIIVRAFSEIWDSVFSSTSPILRF

>CsaV3_3G037720_CsOFP6-19b

MAAVKKKLLINTISVDIGCGSCRNPKSIISQIFRPKPKSPSSYSDRRLFRSLSSSSEKKLSDSDMAYAPEVVGGGGFWKIGGVSVAVEKDSNDPYVDFRQSMLQMILENEIYTQEGLRELLSCFLHLNSPCNHGIIIRAFAEIWDSVFCARSAAPARQRRHVRSRAF

>CsaV3_1G015160_CsOFP6-19c

MSNIRKNKFFTTIFSSTAGCGGCQKPKLSDIVQPDKKPPTTIVRRSSSSSSTDQNGTFSLDEDYTSSASKSTGTQSPVAILIGDSIAVEKDSDDPYEDFRGSMVEMIVEKRIYSPNGLQELLNCFLHLNSPYHHEIIVKAFTQISNEFESSHRLWNMSNNTDWKRRSGGEG

>CsaV3_2G002260_CsOFP8a

MEKRFKLRFSRLFQSSFFSCRSKKNSDILISHKPISKPLISPKPPQLSSNYTPKLPLNPSHFQFPPPPASSPIISPPLSHLNDCLSRHRSKSKIKRRKNPHSRPAPPAPPLPRPHPEDFGSAWWYGGHDETEEDETETLFSSRSLTSDSSVSRRRHRRRHGRRRPERKMRDGFFAVVKNSSNPYMDFKASMAEMVVEKKIFGGKELEELLQCFISLNSRHYHKVIFEVYSEIKEALFFL

>CsaV3_1G024220_CsOFP8b

MDTRFKLRLSRVFQSSFASCRSRNLSDILHKAVFIPSSSDDASFRKISPSETSSDFLLPRRKISHRFPLSPFPSAISRPRTCPPASPISPSKPISQKTTTSTKKKKKQKKQRKQRKQSKKEIPFSPFRSSNFGGTWWYSSEDEDDDDETDTLFSSKSRSSDSSASHRRHKSRRRRGCRSRGSEMGVLPLKGKVKDSFAVVKKSSDPYNDFRMSMLEMIVEKQIFSAKDLEQLLQCFLSLNSHHHHNVILEVFTEIWEALFSDWGS

>CsaV3_2G028590_CsOFP10

MARSFKLKVRLFISFFRFCRPKYLNALTFPKTSSPENSPYRKQIPANPESCYSCYPNPPSPPPSTPVDFFADPSKEKSTSICSSCKLKSYARKNGLLQGKESRAETEHEISSEENQRSTPFSWITRKASKIKKKLKKTGLRSKPLKANGYGEKESEETDALVNSSISFSDDVSPVKRSKRALYLRKLEGKMGKSFVQVKRSKEPQEDFKRSMAQMILEKEIFEIKGLEELLQCYLTLNSPEYHRIIVGAFSEVWEFLFYDSHLNKAVQRD

>CsaV3_7G027990_CsOFP12-16a

MPTKLGRNYLNLCFTKIKNPLSTAQSSPPITHTADRRRQTTRSFSSTAAAFITNYNSLYEITTTTTTNSDSNSPSTPLFGLTNDIGVADPDAYVAVDFITAFTSHRFFFSSPGSSNSIIESTTTTTTESTTTMSLSSEYSARYEGNDDDLMIFNNSHVIPTYSPDPYMDFRRSMQEMMEAREKMTTAVATTTTMKKSSWEFLHELLLCYLALNPKTTHKHILKAFADIATVIKPPLAMKETEEEENVDREKGESMVDDRGAGGGGCECEMSGQQNDRD

>CsaV3_3G013290_CsOFP12-16b

MSSLKKRNFHLWFSKLRCFPATVKPSSPPQTPNKKPFSITHLENSDYSSTSTAADDGFFSDDSSSDSDAIVPDFSAAVASHRFFFSSPGCSNSIFDSSPDTHHSTAVSAAVHGGVEVRKVSMDPFVDFRASMQEMVEARDRPVDVRRDWEYLQELLLCYLQINPVDTHKFIL RAFSDLVVYLLESSPESFSDRRIRPHNINSNSW

>CsaV3_7G022480_CsOFP12-16c

MSNLKFLNNLYSFFSKLKFSPPPPVIASHTPPSDCYFTSNPISSTTADDCDDFFSTSSDADDSISDDLAALLASRRFFFSSPGRSNSIFEYSSCSRRQQPHDVLVSEGHRIRKYSMDPYADFRRSMQEMVEARELEDVRSDSEFLRELLSCYLRLNPKNTHKFIVKAFSDLVLSLLAS SSPTPAPASIARRKVVTSR

>CsaV3_3G037710_CsOPF13a

MLTKKKKMMMMMRLPSLFKYLAIDDKSTFPWPSCRQPRTLSFRTTSAAVATATDSSDSFFTLSSESSGSLSTVSESSGGDPIERMIRDLRSTKRLHFEPTGKSSSIVEDDTVSHPLKEGTTVMSMDSDDPYSDFRKSMEEMVEAHGMKDWESLEELLNWYLRVNGKKNHGFILGAFVDLLVSLAMASSSSSSSCSSSLCCYSSSSSSSSLPCVSSSMEIEEISSLDEHHHHVYS

>CsaV3_5G035260_CsOFP13b

MANTKKKHHLIKNFPSIFKSKLPTSHPWEWPDWPSCKHPKTLSFRSQNDLVFKTVNSIFFDQPFETTTTTTTPDYSVSATNSTPAVDSEESLETVVRGARSERLFFEPDDTSSILEKSKSIDSVETELLPKSGFKESLIVSIESENPYEDFRKSMGEMVESHGVKDWDGLEELLGWYLKANWKNNHRFIIGAFVDLLIHILLASSSSSSSSTSTSTSSSSSSSLCSNSDSNYYTCTESSSSSCSCSSSLRSLNPSSIRKELDHDQVEDNHDHITETL

>CsaV3_6G004380_CsOFP13c

MGKKMKLLPSLFKNRASPDRPWQWPLCGPSKTPSFRAGPDDHQIFSTLNSIFFDNFFSDPIHTPDSWFATSSLFESARVSLSTEFEDDLELVIRGAKSERLIFEPGETNSILEKSRGVEEGGKCEESIRFEGSVVVLMAMESEDPYLDFRRSMEEMVECHGIRNWEWLEELLNWYLRMNGMKNHGYILGAFVDLLVDLGGGDGSTDSTSIFSDDLIIQRHDRERCDV

>CsaV3_6G040950_CsOFP13d

MANKLKKLPNFLNKSPWLWRSCTQSRTLSFRHPNDIFRTINSAYDDEEDQDYYDYDYDEDDEEETEGDDMSNDEDQIEALVRGLRVRQGKRLFLELDETNSIMTTTVAVATVVGGNYQVPFKESVAMAMESKDPYLDFKKSMEEMVEAHELKNWKGMERLLSWYLKANGKANHEFIIGAFVDLLVDLAFSASSNFSNNSSSSPSSSSSSSTTTTSSLLCSSTSTFPNSSSCSSCSSFRAPNSIISSNSVETAEEIVELECSCSSSSIRVAPPCLSSLFEDDEEEDIEEGF

>CsaV3_7G033180_CsOFP14

MPKKLQKSLQDYLSKIKKPTPQLQFPNPQTFSSSKSWILHGCKHPKTLSFAIDRKQVDAVGNKEDAAATLADIDRFLFENFRSLYLKEDGDFSDRKVVVGGGGGGGGGGGRDCKNHRGRVVSPESPVDSYGGSHRFFFSPDLSGSDLPDDSHTESSENAGSSSSSLIGEDRGKDLKLPSDCIAILRKSPNPSEEFRRSMQEMMDAHLKQHEKVDWEFMEELLFCYLNLNEKKSYKYILNAFVDLIVILRQKAEEAPAKPRTVRSVRMVRRMI

>CsaV3_4G005140_CsOVATE

MMMTPKRFKLLRIPSFHCCRSNDISVVPTDPPSPPPPPKPHHSSLRRHVSSAFRTAACGCRSSSTNSDDDQICKSSPTLPTHVPPTPLLHSFDDGSTFPKRQRRRKNKKKSKSKSTTLTRLRTSTSSTESGLFSSESFDEIDELEETETLISSSKTISTSDDDNDSSSEFNPQLETIREKPNKINLRRKKEKEKRRRKQKRTTIISPSPEIESPARLSVFQRLIPCTVEGKIRESFAVVKKSADPFEDFKRSMMEMIMEKEMFEEKDLEQLLHCLLSLNDREHHGIIVEAFSEIWQSLFCN

>Solyc02g085500_SlOVATE

MGKSLKLRFSRVIASFNSCRSKNPSSLPQNPNFFPHKLTSTKHISPDFPLIDQNQNQNHRNYVPESTMISVGCCRSEFKWEKEEKFHVVSSSFVSEEEECEEEINLALRPPLTPPRFSRIVVEKKKKKQQRVKKTKTKSRIIRMSTSSADEYSGILSGTNTDWDNNEEETESLVSSSRSCYDFSSDDSSTDFNPHLETICETTTMRRRHKRNANTKRRSIKQSRPSFSSSKGRRSSVSTSSDSELPARLSVFKKLIPCSVDGKVKESFAIVKKSQDPY

>Solyc01g007800_SlOFP2

MSTHRRRIILSNVTVKLGCSSSCIRPKLSSIFHPKPRKSPKSQTQNKNYSNYSSCSSWDTTTTTFSPNSDSTTNESSDFKTSKAVQGFGRIGGESVAVEKDSDDPYLDFRQSMLQMILEKEIYSKDDLKELLNCFLQLNSPYYHGIIVRAFTEIWNGVFSLRPGVAGASSPFLHGGSHVTYR

>Solyc01g007810_SlOFP3

MKLSSLFKNSSQNSSSTTTTTPWPWSLPTCGKPKTLSFRLEKNQHNIYNSTFHLDDINDTTSCSFDDFFSEIDETSSSSTTTINGQDCIEKVIKGLRLEKERLFFEPEETSSILDFQENKNISITSSNININVVDEGNIISFVPMGLDSNDPFVDFRKSMEEMVEAYEIKDWENLEELLTCYLKVNCKSNHGYIVGAFVDLLVNLATFSDNNNNVGVDIGAGVGAGVDESTIIMTTIDEEQQCLSSSTTTTTTTTNHSFTSPLSFCSSSCSTSSSITSTSACLSLLLEEDEVIQTKKH

>Solyc02g072030_SlOFP5

MKWGKKKPSSSLMTHVFPVSWLSKFKQKKVCRSEDQEGAKMRKVDLRTNVCLKQGRFYEDDPYWRISFSEENHPQNPLWCGECDQNSKSSLGEENHKFNDMVSRKISEKPKNEAEFSNRKRNSVKDEKLRKLSRKALEERIAENAREEVTEKDIFEIEPEDEKVMKRGKEKPTAYKSRKARSLSYNDSSPNSVEESCMMFTSLNLEEEADALSEEEFESECLKIKEMSEKSGCQQRKSVYINQKRRRKHGIKVRAYSPRTAKMECRIKALEDMKKARMKTRHETKESFTGDRTVFDSYAIMKSSFDPFSDFRDSMIEMITQRGIKSSEELEELLACYLTLNCDEYHDIIIKVFRQVWFELNQINIGEELQKCCCSDE

>Solyc03g034100_SlOFP6

MAKLLKFRISKAISNSFHSCRSKDPCTLPQHPVPSFLQNTQFITDDHLLFEEMIQQNNESQLISTHHEHFPIITSPSFKHHVSVTPITATGQCSSRNGEAFSTTSDDSHTRSPSHEFKWKKEEDKWQHFIKTNSDDDTKQQPRRKISYSFSSDNSDNDKILIEIKKKISTSKTNFFMMMSTTSSSSSMDENEINFTSKKTKWDYHEDIDITNEDEENETETFISSSRKSHVEFPDDSSLNFSHEFDTIYKNTTRRCQKKIGYSKRRDHVKNTRSRSSRDMNNIGRRSSISTSTTSSDGELPPRLSVFKKLIPCNVEGKVKESFAIVKKSEDPYEDFKSSMMEMILEKKIFEKNDLEQLLQCFLSLNAKNCHGVIVEAFSEIWETLFSPNHN

>Solyc03g120190_SlOFP7

MPKQLQKSLSDYLTKKKKKATAQQTTNSANKTLSSSTSWLLRGCRHPKTPSFSAVDRKEKNVQGENEAATLADVDRFVFENFKSFYYKDDDNEAEIVENPNSLSESPRHIIPPLNHTGSRRFFIAPGSSSSLIEEARTSMTVSDDTGSTSAITITTVTNTNSNELSAISTEYSKETLNANDFITLVTYSPSPYDDFRQSMQEMMEARLKDQGKINWEFMEELLFCYLDLNDKKSYKYILSAFVDQIVILRENSGRVPAISRNVRPLDGELNQRDT

>Solyc03g120790_SlOFP8

MEESYKKIEPQMLLKKTIQKTKNFLYRTPHNLKSFLFGGHHKLPKTACHFNPFLSVSKRFSSSKRIPKTNVKELDDLYRDYYQQWNQPDHNEIQERKMTSKNARKFQGMAEGDYSESQRELAVRFGMEDIESERRKEDEKKGREVLTRSTSKGSLTLLKKMEELEMVEGEDMDHVLDIEEVLQCYTLLNSPVYVDIVDRFFMDMYTEFSIRKPSGSVNSSMRRLGPLKL

>Solyc04g080210_SlOFP9

MTRRFKLKLSMPSFRFCRPKKASFLPKSPMPLSLYKFSPANILDNSPVPVPPSTPHHPYILRKAHNLASKTYNSPSSEYSDPDNNNMRRGESRKSRLNMSFSSVDSGWFSFNSECCDEKPNDETESFMSSPSFESSFDVDHGIDPLSGIRRKKNNNNTKVRRLRRYLSNSLKDSMMPCMADGKVNESFAIVKRSVDPYDDFKNSMKEMIMEKEMFEAEDLEQLLLCFLSLNSRHHHAIIVEAFTEIWEELFGKSSKSMDLKLPRFQ

>Solyc05g055220_SlOFP10

MAKKLKISSIFKKKELGLATWQWPSCTHSKTLSFRGDDNIFKTINSVFFDPFDGIETPQSYSTNSSLDTNSISIESHEEIIKGARSERLFFEQVATSSIFQEPQEENQENDLPFKESVILAMESKDPYLDFKKSMKEMVESQGIKDWDNLQELLACYLKLNGEVNHGFVLGAFVDLLVELVIPTTPSTNSDNSITSYSSVASSSFSCPSSPLSSLGHKETEEQENAKVS

>Solyc06g073040_SlOFP11

MVNYKGGKLLKHQRNRVSFSAKLPEDVRGAFADSTCVVKYSMDPLTDIKESIKEMVKNVGIKDWKEMEELVYCYIVLNSEETM

>Solyc06g074020_SlOFP12

MTRKYDQCLVDNMFGPFPESCCPDEALEMAKQALATRRLSFEENESCSVLSMVGFPFKDCLLLAVETENPKMDFLHSMEQMTKVYGAQRGDMVDWEFMEELLTWFLKINNMKNQHFIVAAFIDLCLGGHVQDVEPVENVEPLTDDIVNVILEELWWGL

>Solyc06g082450_SlOFP13

MNTSCCLKFNNPCKKIVKLFKFKLRKPLFIRRLRIFRPSTRCESNTSTRRKQASQVLSVFRFIRRSKPREEDQVMALKSFSGHIKAPVPSPITPAYARLSGATKKEVVIFQDDVEDACRSFENYLAEMIVEEGKMRDIMDVEELLYCWKNLKSPVFIDLVCRFYGELCKDLFSHTYKDDINSPQKIMQ

>Solyc06g082460_SlOFP14

MGNHKFKFSDMMPNTWFYKLKDMSKTKNHKSPFSSSSTNKSQYSQPRSSFSYTRRSIRVDKIYNSHSYNFLDQPRRSSSSSSKKKSKRKTIYKPSPKHIPSSVTNYVSVSNKLNTSSSVYSTEEDKFPELDFLNSPSSEFDSVDSQTFNELPSTWPNSCNCHFTSSATDIIIDVNDKALSNEFHNLTTEYAEFSDIDQLPPIFTKASNSIKNIKQDENVKAQREKEPKNRVGSPVSRKHYSSSSGVKLRTNSTKVANKRNSVSSSKRRSKTKKESCSASRGTSFAIVKASIDPEKDFRESMVEMVVENNIRASKELENLLACYLSLNSNEYHDLIIKAFEQIWFDLSDLHL

>Solyc07g055240_SlOFP15

MQNSKAAVAAAAKKQKLKGCSALCCSCRLSVSSSSEEAESSSSSRYPTISSLTHAMVQERLDKMIREREEAKNEEMRRRRRRAERDEKTKFIVMIAMEKSSYDPREDFRESIEQMIIANRICDPKDLRRLLNYYVSMNAEEYRGVILEVFHQVCTTFFLSCKQPSSQV

>Solyc09g018200_SlOFP17

MGNYRFKLSDMVTSSWFYKLKDMAKSRTQIKRKQTSSSTSSSSFSIFYSSSNVQQHHRKSYYFSRTLSPNPHQSNVTPMKSSKKRKNTRRNTPKFVNSPKSMILSPSHRRCNDHIFDSVSKIDLPPILTKPNKKEEKTELKFLTVKTEQSTSPKRRISVSSSSTGVKLRTKSPRIISRRSVGEKSYAVVKSSKNPQKDFKESMVEMIVKNNIKTSKDLEELLACYLLLNSHHYHHLIITVFKQIWFDLQLK

>Solyc09g065350_SlOFP18

MPRTTLGTNFNLCFTKLKRSLPLRSIDDNDNDNDNERHQQQHHSMHFCNSVKNFNSLYDLSSSECNIPTSSSTDESDYNYELENNTPDLATIYASQRFFFSSPGHSNSIIDSSSSISSSIASTSSSVGSDAPLEGASRFQRIHPTRIWIFDDRCKKWWRHVD

>Solyc09g082080_SlOFP19

MPRILQKKFYHCLPSFKCLPTILSLPFEETEEEKTEQKKIKNFNSVFDIPSSDSATTSKSLTNSSTTTTEEEDNNTNCTFTSFEDSDYTNIPDFSNIFASQRFFFSSPGNSNSIIDFPPENPKVVTGGVAVQTYSPDPYSDFRRSMQEMVEAHELTNVKANWGFLHELLLCYLNLNPKHTHKYIIRAYSDLVVSLMSMDDSEKKTEGIARP

>Solyc10g076180_SlOFP20

MGNYRFRLSDMMPNAWFYKLKDMAKSSSRRHSHTTSSSNLQLDKKRQPHNNLGCQRKSYYISRNLTITSPISSNSPKLDHNVHITEPSRKSYKKRRSTNFRRRNSPKPVNSSASVESVWTKPDSTPEQYPNSSSSSSSSSPSSILPHKSNPIASISPSCDCRTDYTNQNSANLDPGVHSVSKIDLPRIITKPEKFNEKIQEKQRIVKQEQRIVRRVSTNGVKLRTNSPRITTTTTNSRKSVSSKRTSVTTDSFAVVKSSRNPQKDFRESMVEMIIENNITTSKDLEELLACYLSLNSDEYHDIIIKVFKQIWFEITEIRLK

>Solyc10g082050_SlOFP21

MSTTKKRVILRNVTVKLGCSSSCIRPKFSSIFHPKPRRHHSADSAAVFNHHKTPNNKYSFSNSTITTATTFSPSPTPSPAHYSSDAERAVQGFGRIGGESVAVEKDSDDPYVDFRQSMLQMILEKEIYSKDELRELLNCFLQLNSPYYHGIIVRAFTEIWHCVFSVNPGVTGAESPFL

>Solyc10g082060_SlOFP22

MNLSSLFKSKKKSSFSPFLCPLPHCGIPKTLSLRVENNDNIFNSQRLYNNVDDDMVDKMVEGLKIEKDRFFFEAGEKTSSIMKVSSSILAKSNNELEILPIDESCVITPIDSMDIPCGEGAISIRDQVSSSTLSNNTNNSGKQVEYLPFNDSCIIKLSSSMDPYGSFKKSMVKMVEANLGIKDWNEFLEEMLAWYLEVNEKNNHKYIIGAFCDLWISYSFTSSTTNIPNSFLFSSSEPKSVISPTSTSFVIS

>Solyc10g083070_SlOFP23

MKFSSLFKSNKKPSFSPMLCRLPRCGDLRTLSIRDENNHNIFNSQRFYNNVDDEMVDEVIESLKLEKDRFFVEAGQKTSLILDMSSSRLSKKRTISKRLEFLPFNNDSCVITSMDSIDAYGETSRSILEGSSSRLSKSTNNSTSSKRLGYLPSNDSMDSYGDQETSSILDMSSSSSNDNISSKGLGYLPSNESMDATSILERSKSNSSHGFVYYVPCKKTYAIMRLISRDPYEDIKYFLEKMVDENLEIEDWEESLEELCGWLLEINEKNIHKYIVGAFCDLWMSYSCTSTINTPFGFSSSKPPSLYFMSLIENEADRMIAASTSSVTP

>Solyc10g083080_SlOFP26

MEFFSLFKSKKKPSFSPMLCRLPRCGNLRTLSIRDENNHNIFNSQRFCINVDDDIVDEVIEGLKFEKKRFFFESGEKTSSILNVSSSKLSKSIGNKRLEFPPSDESCVITHIDSIDAYGETSTRSIFKGSSSRLTKNDYSTSNNKFESLPLNNSCVISPSAMRVTSIDPYGYIKKYMEITVEENQGIKDWKESLKEICAWYLENNDNDKNIHKFIIGAFCDLWMSYSGTSTTNTPFGFSTSEPPSPYFMSLIEAKADQIIATSTSSVIP

>Solyc10g083090_SlOFP27

MKFSSLFKSKKKPPFSPMLCRLSRCGNLRTLSIRDENNHNIFNSQRFYNNVDDEMVDEVIENLKLEKDRFFVESGQKTSSLLDMSSSRLSKRRTISKRLEFLPFNNDSYVITLMDSIDAYGETSRSILEGSSSRLSKSTNNSTSSKRLSYRPSNDSMDSYGDQETSSILDMSSLSSNDSISSNGLGYLPSNESMDATSILERSKSNSSHGFVYYVPCKKTYVIMRLISRDPYEDIKYFLERMVDENLEIEDWKESLEELCGWLLEINEKNIHKYIVGAFCDLWMSYSCTSTTNTPFEFNSSKPPSLYFMSMIEDEADQMIAASTFSVIS

>Solyc10g083100_SlOFP28

MKFFSLFKSKKKPSFSPMLCRLPRCGNLRTLSIRDENNHNIFNSQRFCINVDDDIVDEVIEGLKFEKKRFYFEAGEKTSSILDVSCAITHIDSIDAYGKTSTRSILKGTKSRLSKNDNSTSNDMVESLPLNDSCVITPSVMRVTSIDPYGYIKKHMEMMVEENQGIKDWKESLKEICALYLEINYIDKNIHRFIIGAFCDLWMSYSGTSTTNTPFGFSTSEPPSPYFMSLMEA

>Solyc11g006670_SlOFP29

MGKKMNLGSWQWPSCTHSKTQSFRANHIFKTINSIFLDPSNTDHHHHGVVEIETTPESWFTNSSESASFSTESEETGEPLMELIIKGVRSERLFFEPNCTSSSILEHQDQSQDQNQNQNQSQSQSRDQDQSQSQSQEKLKEIEEDVDEELPFKESVALALESEDPYLDFKKSMEEMVDTHEIKDWESLQELLQWYLKMNGKNNHGFIIGAFVDLLIGFTPSNCDSITCYSSAASSFSSIEEKGE

>Solyc11g068780_SlOFP30

MSSKNKKIWNCITSNGTAGCGCSKPKLSEIIQPKPKPRPEPEPNAHSSSTSNSDSPSPTIMPAKIVGSVAVVKDSDDPFGDFRRSMLQMIMEKEIYSYDDLNELLNCFLQLNSPSHHDIILQAFMEIWNNGKNYIAN

>Prupe.6G290900_PpOFP1

MGNHKFRLSDMMPNAWFHKLKDMSKPRKNPNSPHPSKKKKQQQKPTFASTAKFTEPSKPKQQLPHQCLPRQSYYFTRELTSAAPGHRFCSSSPTNPKASDTNFPDPPPKKPSKQKPKKRITSLPSDPHLVTSSVSAGCGCRAPIESVWTKSDSPPELWSSSTLDSSPEPESHDEDDGELELHEPEFRCDRVLATETFDGMVSMSSSCAAYLADSEEKDVVIDVDKASLSMKLSDVKLSDMADNGLYSFSELELAPIITKPPKFSEMVRDVKKKKETKEPSRCRRSSAKFQDRNAHGSLSVKVAKEESTSTKTIKEQRTASSVRRVSSNATSPGVRLRMNSPRIANRKINQANLSRRSVSSNSSSKRRSLSESFAIVKSSFDPQRDFRESMVEMIMENNIKASKDLEDLLACYLSLNSDEYHELIIKVFKQIWFDLTDLRSK

>[PGSC0003DMG400030384](https://solgenomics.net/phenome/locus_display.pl?locus_id=100841)_StOFP20

MGNHKFKFSDMMPNTWFYKLKDMSKTKNHKSPFSSSSTNKSQYSQPRSSFSYTRRSIRVDKIYNSHSYSFLDQPRRSSSSSSKKKSKRKTIYKPSPKHIPSSVSNYVSVSNKLKSSSSVYSTEDDKFPELDFLNSPSSEFDSVESQSFNELASTWPNSCSCHFTSSATDIIIDMNDKAHSNEFHNLNTEYAEISDIDQLPPIITKTSNSIKNINQDENVKAQREQEAKNRVSSPVSRKHYSSSSGVKLRTNSTKVASKRNSVSSSKRRSKAKKESCSTSTGTSFAIVKASIDPEKDFRESMIEMVVENNIRASKDLENLLACYLSLNSNEYHDLIIKAFEQIWFNLSDLHL

>CA10g10680_CaOFP20

MMPNAWIYKLKDMSKVSSKSKGHSHTTSSSSTSSQHSSLNLQPDKKRQPPHKLCCQRKSYYISRNLNPSETFPHNPVSSNSPKASDNFHFTEPPRRSSTKKRSVNRRRNSSSPKLVTSSVSASCSGRASIKSVWTKADSNTPEEYPNSPHSSSSSSMSSSLSLSSDKIQAPKKLDPVTSISPSCIKDTDNLDQKFHSISKIDLPPIITKSEKFDQNQIIIKQEQSIARRASSGVKLRTNSPRITNCRKIQASRKSVSSRRTSVTESFAVVKSSRNPQKDFRESMVEMIVENNIRASKDLEELLACYLSLNSDEYHDLIIKVFKQIWFDITKY
