# Supplementary material for: Targeted Editing and Phenotypic Profiling of CmOFP13 Mutants Reveal Its Role in Melon Fruit Morphogenesis
Source: Physiol Plant. 2025 Nov 29;177(6):e70641. doi: 10.1111/ppl.70641 (PMC12664293; doi:10.1111/ppl.70641)
Supplement: Supplementary file 5 — File S5: ppl70641‐sup‐0005‐FileS5.pdf. [file PPL-177-e70641-s002.pdf]

T0 **4n 65 (79% +1, chimera)**

↓ ⊗

T1 **4n *ofp13+1*_4n (+1/+1/+1/+1)** x VED (**2n**)

↓

3n *ofp13+1*_3n (wt/+1/+1) x VED (**2n**)

↓

2n *ofp13+1*_het (wt/+1)

↓ ⊗

2n *ofp13+1*_homo (+1/+1)

Supplementary File S5. Crossings performed from T0 plants to final edited homozygous plants. Self pollination is represented by a circle with a cross inside. Ploidy is marked in red.
