# Supplementary material for: Targeted Editing and Phenotypic Profiling of CmOFP13 Mutants Reveal Its Role in Melon Fruit Morphogenesis
Source: Physiol Plant. 2025 Nov 29;177(6):e70641. doi: 10.1111/ppl.70641 (PMC12664293; doi:10.1111/ppl.70641)
Supplement: Supplementary file 6 — File S6: ppl70641‐sup‐0006‐FileS6.pdf. [file PPL-177-e70641-s009.pdf]

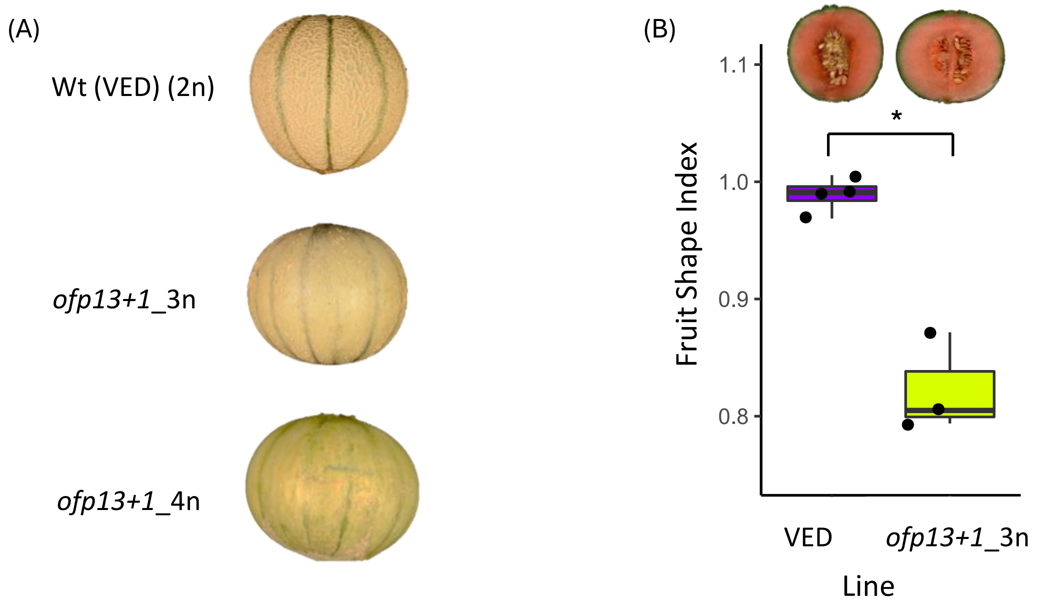

Supplementary File S6. Fruit phenotype from the polyploid edited lines. (A) Fruit images of wild type VED (diploid), edited triploid (*ofp13+1*_3n) and edited tetraploid (*ofp13+1*_4n). (B) Boxplot comparing Fruit Shape Index from VED and edited triploid plants.
