# Supplementary material for: Targeted Editing and Phenotypic Profiling of CmOFP13 Mutants Reveal Its Role in Melon Fruit Morphogenesis
Source: Physiol Plant. 2025 Nov 29;177(6):e70641. doi: 10.1111/ppl.70641 (PMC12664293; doi:10.1111/ppl.70641)
Supplement: Supplementary file 7 — File S7: ppl70641‐sup‐0007‐FileS7.docx. [file PPL-177-e70641-s005.docx]

**Targeted editing and phenotypic profiling of *CmOFP13* mutants reveal its role in melon fruit morphogenesis**

Carlos MAYOBRE, María José GONZALO, Montserrat VERGÉS, Guillem GUARDIA-BERSABÉ, Dídac JIMÉNEZ-SÁNCHEZ, Antonio Jose MONFORTE, Jordi GARCIA-MAS, Marta PUJOL

**Supplementary File S7.** MEME motif analysis

********************************************************************************

MAST - Motif Alignment and Search Tool

********************************************************************************

MAST version 5.5.8 (Release date: Thu May 15 15:01:46 2025 -0700)

For further information on how to interpret these results please access https://meme-suite.org/meme.

To get a copy of the MAST software please access https://meme-suite.org.

********************************************************************************

********************************************************************************

REFERENCE

********************************************************************************

If you use this program in your research, please cite:

Timothy L. Bailey and Michael Gribskov,

"Combining evidence using p-values: application to sequence homology

searches", Bioinformatics, 14(48-54), 1998.

********************************************************************************

********************************************************************************

DATABASE AND MOTIFS

********************************************************************************

DATABASE sequences.fa (peptide)

Last updated on Sat Aug 30 07:24:16 2025

Database contains 85 sequences, 21723 residues

MOTIFS meme.xml (peptide)

MOTIF ID ALT ID WIDTH BEST POSSIBLE MATCH

----- -------------------------------------------------- ------ ----- -------------------

1 YLSLNSKEYHGIIIKAFVDJW MEME-1 21 YLQLNGKEYHGYIVGAFVQIW

2 GESVAVVKSSDDPYEDFRRSMVEMIEENEIK MEME-2 31 GESVAVVKYSDDPYEDFRRSMVEMIVENGIY

3 MGNHKFRFSDMMPNAWFYKLKDMSKSSRPKS MEME-3 31 MGNYKFRFSDMMPNAWFYKLKDMGKAKRHKN

4 KPSFSPMLCRLPRCGNLRTLSIRDENNHNIFNSQRFYNNVDDDMVDEVIE MEME-4 50 KPSFSPMLCRLPRCGNLRTLSIRDENNHNIFNSQRFYNNVDDDMVDEVIE

PAIRWISE MOTIF CORRELATIONS:

MOTIF 1 2 3

----- ----- ----- -----

2 0.29

3 0.15 0.13

4 0.22 0.16 0.13

No overly similar pairs (correlation > 0.60) found.

Random model letter frequencies (from non-redundant database):

A 0.073 C 0.018 D 0.052 E 0.062 F 0.040 G 0.069 H 0.022 I 0.056 K 0.058

L 0.092 M 0.023 N 0.046 P 0.051 Q 0.041 R 0.052 S 0.074 T 0.059 V 0.064

W 0.013 Y 0.033

********************************************************************************

********************************************************************************

SECTION I: HIGH-SCORING SEQUENCES

********************************************************************************

- Each of the following 83 sequences has E-value less than 10.

- The E-value of a sequence is the expected number of sequences

in a random database of the same size that would match the motifs as

well as the sequence does and is equal to the combined p-value of the

sequence times the number of sequences in the database.

- The combined p-value of a sequence measures the strength of the

match of the sequence to all the motifs and is calculated by

o finding the score of the single best match of each motif

to the sequence (best matches may overlap),

o calculating the sequence p-value of each score,

o forming the product of the p-values,

o taking the p-value of the product.

- The sequence p-value of a score is defined as the

probability of a random sequence of the same length containing

some match with as good or better a score.

- The score for the match of a position in a sequence to a motif

is computed by by summing the appropriate entry from each column of

the position-dependent scoring matrix that represents the motif.

- Sequences shorter than one or more of the motifs are skipped.

- The table is sorted by increasing E-value.

********************************************************************************

SEQUENCE NAME DESCRIPTION E-VALUE LENGTH

------------- ----------- -------- ------

SlOFP23 1.5e-75 329

SlOFP27 3e-75 329

SlOFP28 4.9e-72 233

SlOFP26 1.6e-71 269

SlOFP14 8e-65 351

SlOFP20 4.6e-64 321

StOFP20 7.2e-64 351

PpOFP1 5.6e-63 441

CsOFP1a 6.5e-63 327

CmOFP13 6.8e-63 331

CsOFP1b 1.5e-62 325

SlOFP22 2.8e-62 252

CmOFP4 4.9e-62 345

AtOFP2 3e-58 320

SlOFP17 1.1e-51 251

AtOFP3 1e-50 296

AtOFP1 8.9e-49 270

SlOFP2 4.4e-39 182

SlOFP21 1.3e-38 178

CmOFP19 4.4e-38 167

CsOFP6-19b 4.4e-38 167

CmOFP12 5e-38 149

CsOFP6-19a 5.7e-38 156

CaOFP20 2.3e-35 332

AtOFP6 4.4e-35 159

SlOFP30 2.8e-34 137

CsOFP6-19c 3.8e-34 171

CmOFP2 5.6e-33 468

CmOFP15 8.4e-33 170

CsOFP5a 1.5e-32 468

SlOFP29 4.3e-32 244

AtOFP4 5.8e-32 305

CsOFP13d 9.7e-32 290

CsOFP8b 1.1e-31 265

CmOFP8 1.6e-31 301

SlOFP6 1.8e-31 391

CsOFP13c 2.2e-31 227

CmOPF18 3.3e-31 269

CmOFP9 5.8e-31 227

CsOPF13a 8.8e-31 234

AtOFP8 1.2e-30 221

CsOVATE 1.5e-30 301

CmOFP5 1.5e-30 235

CmOFP21 1.9e-30 289

CmOFP14 3e-30 241

CsOFP10 3.1e-30 270

SlOFP9 4.6e-30 266

SlOFP3 4.7e-30 298

AtOFP7 7.1e-30 315

AtOFP5 1.2e-29 349

SlOFP19 4.6e-29 211

SlOFP5 1e-28 377

AtOFP13 1.3e-27 260

CmOFP3 1.4e-27 176

SlOFP10 2.4e-27 229

CmOFP6 3.2e-27 284

CsOFP12-16c 6e-27 197

CsOFP13b 1.2e-26 277

CsOFP8a 1.2e-26 239

CmOFP20 1.9e-26 237

CsOFP5b 3e-26 168

CmOFP7 3.1e-25 263

CsOFP12-16a 9.3e-25 278

CmOFP13+1 3e-24 154

CsOFP12-16b 1.8e-23 205

AtOFP10 3.2e-23 196

CmOFP1 3.7e-23 205

SlOFP15 5.3e-23 168

AtOFP12 6e-23 226

AtOFP14 8.3e-23 294

SlOFP7 1.9e-21 275

AtOFP15 2e-21 261

AtOPF16 2.5e-21 244

AtOFP11 4.1e-21 182

CsOFP14 7.2e-21 272

CmOFP10 3.9e-20 298

AtOFP18 1.5e-18 282

SlOFP12 1.9e-16 158

SlOFP11 1.5e-09 83

SlOFP13 2.5e-06 188

SlOFP8 0.00013 229

CmOFP16 0.00068 189

AtOFP17 0.023 195

********************************************************************************

********************************************************************************

SECTION II: MOTIF DIAGRAMS

********************************************************************************

- The ordering and spacing of all non-overlapping motif occurrences

are shown for each high-scoring sequence listed in Section I.

- A motif occurrence is defined as a position in the sequence whose

match to the motif has POSITION p-value less than 0.0001.

- The POSITION p-value of a match is the probability of

a single random subsequence of the length of the motif

scoring at least as well as the observed match.

- For each sequence, all motif occurrences are shown unless there

are overlaps. In that case, a motif occurrence is shown only if its

p-value is less than the product of the p-values of the other

(lower-numbered) motif occurrences that it overlaps.

- The table also shows the E-value of each sequence.

- Spacers and motif occurences are indicated by

o -d- `d' residues separate the end of the preceding motif

occurrence and the start of the following motif occurrence

o [n] occurrence of motif `n' with p-value less than 0.0001.

********************************************************************************

SEQUENCE NAME E-VALUE MOTIF DIAGRAM

------------- -------- -------------

SlOFP23 1.5e-75 11-[4]-45-[2]-81-[2]-13-[1]-46

SlOFP27 3e-75 11-[4]-45-[2]-81-[2]-13-[1]-46

SlOFP28 4.9e-72 11-[4]-74-[2]-15-[1]-31

SlOFP26 1.6e-71 11-[4]-95-[2]-15-[1]-46

SlOFP14 8e-65 [3]-249-[2]-11-[1]-8

SlOFP20 4.6e-64 [3]-218-[2]-11-[1]-9

StOFP20 7.2e-64 [3]-249-[2]-11-[1]-8

PpOFP1 5.6e-63 [3]-338-[2]-11-[1]-9

CsOFP1a 6.5e-63 [3]-139-[1]-62-[2]-11-[1]-11

CmOFP13 6.8e-63 [3]-143-[1]-62-[2]-11-[1]-11

CsOFP1b 1.5e-62 [3]-220-[2]-11-[1]-11

SlOFP22 2.8e-62 11-[4]-89-[2]-13-[1]-37

CmOFP4 4.9e-62 18-[3]-222-[2]-11-[1]-11

AtOFP2 3e-58 [3]-219-[2]-11-[1]-7

SlOFP17 1.1e-51 [3]-151-[2]-11-[1]-6

AtOFP3 1e-50 4-[3]-187-[2]-11-[1]-11

AtOFP1 8.9e-49 1-[3]-164-[2]-11-[1]-11

SlOFP2 4.4e-39 92-[2]-11-[1]-27

SlOFP21 1.3e-38 97-[2]-11-[1]-18

CmOFP19 4.4e-38 81-[2]-11-[1]-23

CsOFP6-19b 4.4e-38 81-[2]-11-[1]-23

CmOFP12 5e-38 73-[2]-11-[1]-13

CsOFP6-19a 5.7e-38 80-[2]-11-[1]-13

CaOFP20 2.3e-35 263-[2]-11-[1]-6

AtOFP6 4.4e-35 64-[2]-11-[1]-32

SlOFP30 2.8e-34 65-[2]-11-[1]-9

CsOFP6-19c 3.8e-34 82-[2]-11-[1]-26

CmOFP2 5.6e-33 382-[2]-11-[1]-23

CmOFP15 8.4e-33 81-[2]-11-[1]-26

CsOFP5a 1.5e-32 382-[2]-11-[1]-23

SlOFP29 4.3e-32 1-[4]-101-[2]-11-[1]-29

AtOFP4 5.8e-32 231-[2]-11-[1]-11

CsOFP13d 9.7e-32 121-[2]-11-[1]-106

CsOFP8b 1.1e-31 193-[2]-11-[1]-9

CmOFP8 1.6e-31 231-[2]-11-[1]-7

SlOFP6 1.8e-31 319-[2]-11-[1]-9

CsOFP13c 2.2e-31 132-[2]-11-[1]-32

CmOPF18 3.3e-31 190-[2]-11-[1]-16

CmOFP9 5.8e-31 132-[2]-11-[1]-32

CsOPF13a 8.8e-31 25-[4]-42-[2]-11-[1]-54

AtOFP8 1.2e-30 152-[2]-11-[1]-6

CsOVATE 1.5e-30 232-[2]-11-[1]-6

CmOFP5 1.5e-30 25-[4]-42-[2]-11-[1]-55

CmOFP21 1.9e-30 123-[2]-11-[1]-103

CmOFP14 3e-30 169-[2]-11-[1]-9

CsOFP10 3.1e-30 191-[2]-11-[1]-16

SlOFP9 4.6e-30 184-[2]-11-[1]-19

SlOFP3 4.7e-30 16-[4]-73-[2]-11-[1]-96

AtOFP7 7.1e-30 224-[2]-11-[1]-28

AtOFP5 1.2e-29 280-[2]-11-[1]-6

SlOFP19 4.6e-29 125-[2]-15-[1]-19

SlOFP5 1e-28 90-[3]-173-[2]-11-[1]-20

AtOFP13 1.3e-27 11-[4]-83-[2]-13-[1]-51

CmOFP3 1.4e-27 101-[2]-11-[1]-12

SlOFP10 2.4e-27 114-[2]-11-[1]-52

CmOFP6 3.2e-27 146-[2]-11-[1]-75

CsOFP12-16c 6e-27 105-[2]-15-[1]-25

CsOFP13b 1.2e-26 134-[2]-11-[1]-80

CsOFP8a 1.2e-26 170-[2]-11-[1]-6

CmOFP20 1.9e-26 168-[2]-11-[1]-6

CsOFP5b 3e-26 87-[2]-11-[1]-18

CmOFP7 3.1e-25 148-[2]-23-[1]-40

CsOFP12-16a 9.3e-25 153-[2]-24-[1]-49

CmOFP13+1 3e-24 [3]-123

CsOFP12-16b 1.8e-23 111-[2]-16-[1]-26

AtOFP10 3.2e-23 94-[2]-11-[1]-39

CmOFP1 3.7e-23 111-[2]-16-[1]-26

SlOFP15 5.3e-23 91-[2]-11-[1]-14

AtOFP12 6e-23 148-[2]-15-[1]-11

AtOFP14 8.3e-23 189-[2]-16-[1]-37

SlOFP7 1.9e-21 48-[4]-81-[2]-15-[1]-29

AtOFP15 2e-21 106-[2]-12-[1]-91

AtOPF16 2.5e-21 161-[2]-20-[1]-11

AtOFP11 4.1e-21 99-[2]-16-[1]-15

CsOFP14 7.2e-21 178-[2]-15-[1]-27

CmOFP10 3.9e-20 204-[2]-15-[1]-27

AtOFP18 1.5e-18 133-[2]-12-[1]-85

SlOFP12 1.9e-16 57-[2]-16-[1]-33

SlOFP11 1.5e-09 31-[2]-21

SlOFP13 2.5e-06 104-[2]-12-[1]-20

SlOFP8 0.00013 184-[1]-24

CmOFP16 0.00068 107-[2]-12-[1]-18

AtOFP17 0.023 167-[1]-7

********************************************************************************

********************************************************************************

SECTION III: ANNOTATED SEQUENCES

********************************************************************************

- The positions and p-values of the non-overlapping motif occurrences

are shown above the actual sequence for each of the high-scoring

sequences from Section I.

- A motif occurrence is defined as a position in the sequence whose

match to the motif has POSITION p-value less than 0.0001 as

defined in Section II.

- For each sequence, the first line specifies the name of the sequence.

- The second (and possibly more) lines give a description of the

sequence.

- Following the description line(s) is a line giving the length,

combined p-value, and E-value of the sequence as defined in Section I.

- The next line reproduces the motif diagram from Section II.

- The entire sequence is printed on the following lines.

- Motif occurrences are indicated directly above their positions in the

sequence on lines showing

o the motif number of the occurrence,

o the position p-value of the occurrence,

o the best possible match to the motif, and

o columns whose match to the motif has a positive score (indicated

by a plus sign).

********************************************************************************

SlOFP23

LENGTH = 329 COMBINED P-VALUE = 1.76e-77 E-VALUE = 1.5e-75

DIAGRAM: 11-[4]-45-[2]-81-[2]-13-[1]-46

[4]

2.1e-62

KPSFSPMLCRLPRCGNLRTLSIRDENNHNIFNSQRFYNNVDDDMVDEVIE

+++++++++++++++ ++++++++++++++++++++++++++++++++++

1 MKFSSLFKSNKKPSFSPMLCRLPRCGDLRTLSIRDENNHNIFNSQRFYNNVDDEMVDEVIESLKLEKDRFFVEAG

[2]

4.1e-05

GESVAVVKYSDDPYEDFRRSMVEMIVENGIY

+ +++ +++++ ++ ++ ++ ++ +

76 QKTSLILDMSSSRLSKKRTISKRLEFLPFNNDSCVITSMDSIDAYGETSRSILEGSSSRLSKSTNNSTSSKRLGY

[2]

6.3e-15

GESVAVV

+++ +++

151 LPSNDSMDSYGDQETSSILDMSSSSSNDNISSKGLGYLPSNESMDATSILERSKSNSSHGFVYYVPCKKTYAIMR

[1]

4.6e-15

KYSDDPYEDFRRSMVEMIVENGIY YLQLNGKEYHGYIVGAFVQIW

+ + ++++++++ + ++++++ + + ++ ++ ++++++++++++

226 LISRDPYEDIKYFLEKMVDENLEIEDWEESLEELCGWLLEINEKNIHKYIVGAFCDLWMSYSCTSTINTPFGFSS

SlOFP27

LENGTH = 329 COMBINED P-VALUE = 3.55e-77 E-VALUE = 3e-75

DIAGRAM: 11-[4]-45-[2]-81-[2]-13-[1]-46

[4]

1.8e-61

KPSFSPMLCRLPRCGNLRTLSIRDENNHNIFNSQRFYNNVDDDMVDEVIE

+++++++++++ ++++++++++++++++++++++++++++++++++++++

1 MKFSSLFKSKKKPPFSPMLCRLSRCGNLRTLSIRDENNHNIFNSQRFYNNVDDEMVDEVIENLKLEKDRFFVESG

[2]

5.3e-05

GESVAVVKYSDDPYEDFRRSMVEMIVENGIY

+ +++ +++++ ++ ++ ++ ++ +

76 QKTSSLLDMSSSRLSKRRTISKRLEFLPFNNDSYVITLMDSIDAYGETSRSILEGSSSRLSKSTNNSTSSKRLSY

[2]

1.4e-15

GESVAVV

+++++++

151 RPSNDSMDSYGDQETSSILDMSSLSSNDSISSNGLGYLPSNESMDATSILERSKSNSSHGFVYYVPCKKTYVIMR

[1]

4.6e-15

KYSDDPYEDFRRSMVEMIVENGIY YLQLNGKEYHGYIVGAFVQIW

+ + ++++++++ + ++++++ + + ++ ++ ++++++++++++

226 LISRDPYEDIKYFLERMVDENLEIEDWKESLEELCGWLLEINEKNIHKYIVGAFCDLWMSYSCTSTTNTPFEFNS

SlOFP28

LENGTH = 233 COMBINED P-VALUE = 5.72e-74 E-VALUE = 4.9e-72

DIAGRAM: 11-[4]-74-[2]-15-[1]-31

[4]

1.8e-63

KPSFSPMLCRLPRCGNLRTLSIRDENNHNIFNSQRFYNNVDDDMVDEVIE

++++++++++++++++++++++++++++++++++++++++++++++++++

1 MKFFSLFKSKKKPSFSPMLCRLPRCGNLRTLSIRDENNHNIFNSQRFCINVDDDIVDEVIEGLKFEKKRFYFEAG

[2]

5.8e-16

GESVAVVKYSDDPYE

+ ++++ ++++++

76 EKTSSILDVSCAITHIDSIDAYGKTSTRSILKGTKSRLSKNDNSTSNDMVESLPLNDSCVITPSVMRVTSIDPYG

[1]

8.7e-09

DFRRSMVEMIVENGIY YLQLNGKEYHGYIVGAFVQIW

+++ ++ ++++++++ + +++ ++++++++++++

151 YIKKHMEMMVEENQGIKDWKESLKEICALYLEINYIDKNIHRFIIGAFCDLWMSYSGTSTTNTPFGFSTSEPPSP

SlOFP26

LENGTH = 269 COMBINED P-VALUE = 1.92e-73 E-VALUE = 1.6e-71

DIAGRAM: 11-[4]-95-[2]-15-[1]-46

[4]

1.8e-63

KPSFSPMLCRLPRCGNLRTLSIRDENNHNIFNSQRFYNNVDDDMVDEVIE

++++++++++++++++++++++++++++++++++++++++++++++++++

1 MEFFSLFKSKKKPSFSPMLCRLPRCGNLRTLSIRDENNHNIFNSQRFCINVDDDIVDEVIEGLKFEKKRFFFESG

[2] [1]

2.3e-14 8.2e-10

GESVAVVKYSDDPYEDFRRSMVEMIVENGIY YLQLNGKEYHGYIVGAFVQIW

++++ ++++++ +++ ++ +++++++ + ++++ ++++++++++++

151 LNNSCVISPSAMRVTSIDPYGYIKKYMEITVEENQGIKDWKESLKEICAWYLENNDNDKNIHKFIIGAFCDLWMS

SlOFP14

LENGTH = 351 COMBINED P-VALUE = 9.47e-67 E-VALUE = 8e-65

DIAGRAM: [3]-249-[2]-11-[1]-8

[3]

6.9e-35

MGNYKFRFSDMMPNAWFYKLKDMGKAKRHKN

+++++++++++++++++++++++++++++++

1 MGNHKFKFSDMMPNTWFYKLKDMSKTKNHKSPFSSSSTNKSQYSQPRSSFSYTRRSIRVDKIYNSHSYNFLDQPR

[2]

7.5e-27

GESVAVVKYSDDPYEDFRRS

+++++++++++++ ++++++

226 EPKNRVGSPVSRKHYSSSSGVKLRTNSTKVANKRNSVSSSKRRSKTKKESCSASRGTSFAIVKASIDPEKDFRES

[1]

8.2e-20

MVEMIVENGIY YLQLNGKEYHGYIVGAFVQIW

+++++++++++ ++++++ ++++++++++ +++

301 MVEMVVENNIRASKELENLLACYLSLNSNEYHDLIIKAFEQIWFDLSDLHL

SlOFP20

LENGTH = 321 COMBINED P-VALUE = 5.40e-66 E-VALUE = 4.6e-64

DIAGRAM: [3]-218-[2]-11-[1]-9

[3]

2.1e-34

MGNYKFRFSDMMPNAWFYKLKDMGKAKRHKN

+++++++++++++++++++++++++++ +++

1 MGNYRFRLSDMMPNAWFYKLKDMAKSSSRRHSHTTSSSNLQLDKKRQPHNNLGCQRKSYYISRNLTITSPISSNS

[2] [1]

4.9e-25 4.9e-21

GESVAVVKYSDDPYEDFRRSMVEMIVENGIY YLQLNGKEY

++++++++++ ++++++++++++++ ++++ +++++++++

226 NSPRITTTTTNSRKSVSSKRTSVTTDSFAVVKSSRNPQKDFRESMVEMIIENNITTSKDLEELLACYLSLNSDEY

HGYIVGAFVQIW

++++++++++++

301 HDIIIKVFKQIWFEITEIRLK

StOFP20

LENGTH = 351 COMBINED P-VALUE = 8.42e-66 E-VALUE = 7.2e-64

DIAGRAM: [3]-249-[2]-11-[1]-8

[3]

6.9e-35

MGNYKFRFSDMMPNAWFYKLKDMGKAKRHKN

+++++++++++++++++++++++++++++++

1 MGNHKFKFSDMMPNTWFYKLKDMSKTKNHKSPFSSSSTNKSQYSQPRSSFSYTRRSIRVDKIYNSHSYSFLDQPR

[2]

8.3e-26

GESVAVVKYSDDPYEDFRRS

+++++++++++++ ++++++

226 EAKNRVSSPVSRKHYSSSSGVKLRTNSTKVASKRNSVSSSKRRSKAKKESCSTSTGTSFAIVKASIDPEKDFRES

[1]

8.2e-20

MVEMIVENGIY YLQLNGKEYHGYIVGAFVQIW

+++++++++++ ++++++ ++++++++++ +++

301 MIEMVVENNIRASKDLENLLACYLSLNSNEYHDLIIKAFEQIWFNLSDLHL

PpOFP1

LENGTH = 441 COMBINED P-VALUE = 6.58e-65 E-VALUE = 5.6e-63

DIAGRAM: [3]-338-[2]-11-[1]-9

[3]

3.9e-33

MGNYKFRFSDMMPNAWFYKLKDMGKAKRHKN

+++++++++++++++++++++++++++++++

1 MGNHKFRLSDMMPNAWFHKLKDMSKPRKNPNSPHPSKKKKQQQKPTFASTAKFTEPSKPKQQLPHQCLPRQSYYF

[2]

2.2e-2

GESVAV

+++++

301 KVAKEESTSTKTIKEQRTASSVRRVSSNATSPGVRLRMNSPRIANRKINQANLSRRSVSSNSSSKRRSLSESFAI

[1]

7 1.6e-19

VKYSDDPYEDFRRSMVEMIVENGIY YLQLNGKEYHGYIVGAFVQIW

+++++++++++++++++++++++++ +++++++++++++++++++++

376 VKSSFDPQRDFRESMVEMIMENNIKASKDLEDLLACYLSLNSDEYHELIIKVFKQIWFDLTDLRSK

CsOFP1a

LENGTH = 327 COMBINED P-VALUE = 7.70e-65 E-VALUE = 6.5e-63

DIAGRAM: [3]-139-[1]-62-[2]-11-[1]-11

[3]

4.9e-33

MGNYKFRFSDMMPNAWFYKLKDMGKAKRHKN

+++++++++++++++++++++++++++++++

1 MRNHKFRFSDMIPNAWFYKLKEIGGASRPKSFRSNKNPHAHPPPPPPPSKHKQQPPPPPPPPHSRSRKSYYFTRQ

[1]

6.7e-06

YLQLNGKEYHGYIVGAFVQIW

+ + + +++++ ++

151 DFRTDKILTAEASEHFEHDIVIDVSSNYSNNAVIGAFDELELPPIITKQKKKTETKQRTTTTTTTGTKKVAGNSP

[2] [1]

3.2e-26 2.6e-

GESVAVVKYSDDPYEDFRRSMVEMIVENGIY YLQLN

++++++++++++++++++++++++++++++ +++++

226 GVRLRIHSPKIGYRKMGGRKSVSSRRSLSESLAIMKSSYDPQKDFRESMVEMIVENNIRSSKELEDLLACYLCLN

20

GKEYHGYIVGAFVQIW

++++++++++++++++

301 ADEYHDLIIKVFKQIWFDLTQPSPPPL

CmOFP13

LENGTH = 331 COMBINED P-VALUE = 7.95e-65 E-VALUE = 6.8e-63

DIAGRAM: [3]-143-[1]-62-[2]-11-[1]-11

[3]

4.9e-33

MGNYKFRFSDMMPNAWFYKLKDMGKAKRHKN

+++++++++++++++++++++++++++++++

1 MRNHKFRFSDMIPNAWFYKLKEIGGASRPKSFRSNKNPHHPPPPPPPSKHKQPPPPPPHSRSRKSYYFTRQLESN

[1]

6.7e-06

YLQLNGKEYHGYIVGAFVQIW

+ + + +++++ ++

151 DTSPDFRTDKILTAEASKHFEHDIVIDVSSNYSNNAVIGAFDELELPPIITKQRKKTETKQRTTTTTTAGTKKVA

[2] [

3.2e-26 2

GESVAVVKYSDDPYEDFRRSMVEMIVENGIY Y

++++++++++++++++++++++++++++++ +

226 GNSPGVRLRIHSPKIGYRKMGGRKSVSSRRSLSESLAIMKSSYDPQKDFRESMVEMIVENNIRGSKELEDLLACY

1]

.6e-20

LQLNGKEYHGYIVGAFVQIW

++++++++++++++++++++

301 LCLNADEYHDLIIKVFKQIWFDLTQPSPPPL

CsOFP1b

LENGTH = 325 COMBINED P-VALUE = 1.77e-64 E-VALUE = 1.5e-62

DIAGRAM: [3]-220-[2]-11-[1]-11

[3]

1.2e-34

MGNYKFRFSDMMPNAWFYKLKDMGKAKRHKN

+++++++++++++++++++++++++++++++

1 MGNYRFRVSDMMPNSWFYKLKDMTTIIRRRNSKKDQSSKNSHTTDLVYSHPRKSIHFTPSQLAANNSPLEPPRRS

[2] [1]

2.0e-24 4.3e-20

GESVAVVKYSDDPYEDFRRSMVEMIVENGIY YLQLNGK

+++++++++ ++++++++++++++++++++ +++++ +

226 IVNSPRVSSSKRFSHVSRRRSGKRSLNDSLAIVKSTKDPQRDFRESMVEMIVENKISGSNELEDLLACYLSLNTD

EYHGYIVGAFVQIW

++++++++++++++

301 EYHDIIVKVFKQIWFDMTDIIGDHY

SlOFP22

LENGTH = 252 COMBINED P-VALUE = 3.30e-64 E-VALUE = 2.8e-62

DIAGRAM: 11-[4]-89-[2]-13-[1]-37

[4]

9.8e-45

KPSFSPMLCRLPRCGNLRTLSIRDENNHNIFNSQRFYNNVDDDMVDEVIE

+ ++++ ++ ++ ++ + +++++ +++++++++++ ++++++++++ +++

1 MNLSSLFKSKKKSSFSPFLCPLPHCGIPKTLSLRVENNDNIFNSQRLYNNVDDDMVDKMVEGLKIEKDRFFFEAG

[2] [1]

5.8e-16 1.0e-17

GESVAVVKYSDDPYEDFRRSMVEMIVENGIY YLQLNGKEYHGYIVGAFVQIW

+ +++ +++++++ ++++++ ++++++++ ++ ++ +++++++++++++++

151 DSCIIKLSSSMDPYGSFKKSMVKMVEANLGIKDWNEFLEEMLAWYLEVNEKNNHKYIIGAFCDLWISYSFTSSTT

CmOFP4

LENGTH = 345 COMBINED P-VALUE = 5.75e-64 E-VALUE = 4.9e-62

DIAGRAM: 18-[3]-222-[2]-11-[1]-11

[3]

1.2e-34

MGNYKFRFSDMMPNAWFYKLKDMGKAKRHKN

+++++++++++++++++++++++++++++++

1 MVGDRVEWSRERNNHFWRMGNYRFRVSDMMPNSWFYKLKDMTTIIRRRNKNKDQPSKNTHTTTDLAYSHPRKSIH

[2]

5.2e-24

GESVAVVKYSDDPYEDFRRSMVEMIVENG

+++++++++ +++++++++++++++++++

226 PIRSSPSRRFLLNSPGPKLRIVNSPRVSSSKRFGHVGRRKSGKRSLNDSLAIVKSTEDPQRDFRESMMEMIVENK

[1]

4.3e-20

IY YLQLNGKEYHGYIVGAFVQIW

+ +++++ +++++++++++++++

301 ISGSSELEDLLACYLSLNTDEYHDIIVKVFKQIWFDMTDIIGVHY

AtOFP2

LENGTH = 320 COMBINED P-VALUE = 3.48e-60 E-VALUE = 3e-58

DIAGRAM: [3]-219-[2]-11-[1]-7

[3]

9.6e-31

MGNYKFRFSDMMPNAWFYKLKDMGKAKRHKN

+++++++++ +++++++++++++++++++++

1 MGNYKFRISEMLPNAWFHKLKDVTKHSKPKNKASSSSSNTCSKKKPSSDSLPQHSYFSNSLVANNPPHHNSPRNS

[2] [1]

4.9e-25 6.9e-19

GESVAVVKYSDDPYEDFRRSMVEMIVENGIY YLQLNGKE

++++++++++++ +++++++++++++++++ ++++++++

226 VNSPRIQLSGTRRSTSRRSESKQDVLESFAVMKRSVDPKKDFRESMIEMIEENNIRASKDLEDLLACYLTLNPKE

YHGYIVGAFVQIW

++++++ ++ +++

301 YHDLIIHVFEQIWLQLTKTK

SlOFP17

LENGTH = 251 COMBINED P-VALUE = 1.29e-53 E-VALUE = 1.1e-51

DIAGRAM: [3]-151-[2]-11-[1]-6

[3]

1.0e-27

MGNYKFRFSDMMPNAWFYKLKDMGKAKRHKN

++++++++++++ +++++++++++++++++

1 MGNYRFKLSDMVTSSWFYKLKDMAKSRTQIKRKQTSSSTSSSSFSIFYSSSNVQQHHRKSYYFSRTLSPNPHQSN

[2] [

5.2e-23 6

GESVAVVKYSDDPYEDFRRSMVEMIVENGIY Y

+ ++++++++++++++++++++++++ ++++ +

151 TSPKRRISVSSSSTGVKLRTKSPRIISRRSVGEKSYAVVKSSKNPQKDFKESMVEMIVKNNIKTSKDLEELLACY

1]

.2e-17

LQLNGKEYHGYIVGAFVQIW

+ +++++++ +++ ++++++

226 LLLNSHHYHHLIITVFKQIWFDLQLK

AtOFP3

LENGTH = 296 COMBINED P-VALUE = 1.22e-52 E-VALUE = 1e-50

DIAGRAM: 4-[3]-187-[2]-11-[1]-11

[3]

1.5e-27

MGNYKFRFSDMMPNAWFYKLKDMGKAKRHKN

++ ++++++++++ ++ ++++ +++++++++

1 MKQKMGTHKFRFSDMMPHSWLYKLKGMSRSSRKHQLSSPKHLSSADASSSRKLRDPLRRLSSTAHHPQASNSPPK

[2]

1.1

GES

+ +

151 ELSVRKLDDVPEDPSVSPNLSPETAKEPPFEMMTQQKLKKPKAHSSGIKIPTKIVRKKKKERTSQVSKKKGVVKS

[1]

e-23 9.4e-16

VAVVKYSDDPYEDFRRSMVEMIVENGIY YLQLNGKEYHGYIVGAFVQIW

++++++++++ +++++++++++++++++ ++++++ ++++++++++ +

226 FAIVLSSVDPEKDFRESMVEMIMENKMREQKDLEDLLACYLSLNSSEYHDVIIKAFENTWLHLTQGLSISL

AtOFP1

LENGTH = 270 COMBINED P-VALUE = 1.04e-50 E-VALUE = 8.9e-49

DIAGRAM: 1-[3]-164-[2]-11-[1]-11

[3]

1.9e-24

MGNYKFRFSDMMPNAWFYKLKDMGKAKRHKN

+++++++ +++++++++ +++++++++

1 MGNNYRFKLSELIPNAWFYKLRDMSKSKKKNLQSQPNSTTSKKKHHAVSTPTSTTPLSPRPPRRPSHSSKAPPSH

[2]

1.6e-21

GESVAVVKYSDDPYEDFRRSMVEMIVENG

+ +++++++++ +++++++++++ +++

151 ELRPIITKTAATARKTAVNSPAGVRLRMRSPRISVSSSARRSGSSARRSRAVVKASVDPKRDFKESMEEMIAENK

[1]

6.9e-19

IY YLQLNGKEYHGYIVGAFVQIW

++ ++++++++++ +++ ++++++

226 IRATKDLEELLACYLCLNSDEYHAIIINVFKQIWLDLNLPPPHSK

SlOFP2

LENGTH = 182 COMBINED P-VALUE = 5.23e-41 E-VALUE = 4.4e-39

DIAGRAM: 92-[2]-11-[1]-27

[2] [1]

7.3e-30 1.0e-20

GESVAVVKYSDDPYEDFRRSMVEMIVENGIY YLQLNGKEYHGYIVGA

+++++++++++++++++++++++++++++++ ++++++++++++++++

76 SSDFKTSKAVQGFGRIGGESVAVEKDSDDPYLDFRQSMLQMILEKEIYSKDDLKELLNCFLQLNSPYYHGIIVRA

FVQIW

+++++

151 FTEIWNGVFSLRPGVAGASSPFLHGGSHVTYR

SlOFP21

LENGTH = 178 COMBINED P-VALUE = 1.55e-40 E-VALUE = 1.3e-38

DIAGRAM: 97-[2]-11-[1]-18

[2] [1]

7.3e-30 1.0e-20

GESVAVVKYSDDPYEDFRRSMVEMIVENGIY YLQLNGKEYHG

+++++++++++++++++++++++++++++++ +++++++++++

76 TPSPAHYSSDAERAVQGFGRIGGESVAVEKDSDDPYVDFRQSMLQMILEKEIYSKDELRELLNCFLQLNSPYYHG

YIVGAFVQIW

++++++++++

151 IIVRAFTEIWHCVFSVNPGVTGAESPFL

CmOFP19

LENGTH = 167 COMBINED P-VALUE = 5.18e-40 E-VALUE = 4.4e-38

DIAGRAM: 81-[2]-11-[1]-23

[2] [1]

2.7e-29 3.9e-19

GESVAVVKYSDDPYEDFRRSMVEMIVENGIY YLQLNGKEYHGYIVGAFVQIW

+++++++++++++++++++++++++++++++ +++++++++++++++++++++

76 GFWKIGGVSVAVEKDSNDPYVDFRQSMLQMILENEIYTQEGLRELLSCFLHLNSPCNHGIIIRAFAEIWDGVFCA

CsOFP6-19b

LENGTH = 167 COMBINED P-VALUE = 5.18e-40 E-VALUE = 4.4e-38

DIAGRAM: 81-[2]-11-[1]-23

[2] [1]

2.7e-29 3.9e-19

GESVAVVKYSDDPYEDFRRSMVEMIVENGIY YLQLNGKEYHGYIVGAFVQIW

+++++++++++++++++++++++++++++++ +++++++++++++++++++++

76 GFWKIGGVSVAVEKDSNDPYVDFRQSMLQMILENEIYTQEGLRELLSCFLHLNSPCNHGIIIRAFAEIWDSVFCA

CmOFP12

LENGTH = 149 COMBINED P-VALUE = 5.89e-40 E-VALUE = 5e-38

DIAGRAM: 73-[2]-11-[1]-13

[2

2.

GE

++

1 MAAPRRNLQPTSLSVDLNICRPKLLSHLFHHLKPKPSLKSPNHHHRFSSASSDSESESETRTSITFRGFGRSGGE

] [1]

5e-31 1.2e-18

SVAVVKYSDDPYEDFRRSMVEMIVENGIY YLQLNGKEYHGYIVGAFVQIW

+++++++++++++++++++++++++++++ +++++++ +++++++++++++

76 SVAVEKDSDDPYLDFRHSMVQMILENEIYSKEDLRGLLRCFLQLNSPSHHGIIVRAFSEIWDSVFSATSPILRF

CsOFP6-19a

LENGTH = 156 COMBINED P-VALUE = 6.76e-40 E-VALUE = 5.7e-38

DIAGRAM: 80-[2]-11-[1]-13

[2] [1]

2.5e-31 1.2e-18

GESVAVVKYSDDPYEDFRRSMVEMIVENGIY YLQLNGKEYHGYIVGAFVQIW

+++++++++++++++++++++++++++++++ +++++++ +++++++++++++

76 FGRSGGESVAVEKDSDDPYLDFRHSMVQMILENEIYSKEDLRGLLRCFLQLNSPSHHGIIVRAFSEIWDSVFSST

CaOFP20

LENGTH = 332 COMBINED P-VALUE = 2.76e-37 E-VALUE = 2.3e-35

DIAGRAM: 263-[2]-11-[1]-6

[2]

3.8e-27

GESVAVVKYSDDPYEDFRRSMVEMIVENGIY

++++++++++ ++++++++++++++++++++

226 IARRASSGVKLRTNSPRITNCRKIQASRKSVSSRRTSVTESFAVVKSSRNPQKDFRESMVEMIVENNIRASKDLE

[1]

1.8e-20

YLQLNGKEYHGYIVGAFVQIW

+++++++++++++++++++++

301 ELLACYLSLNSDEYHDLIIKVFKQIWFDITKY

AtOFP6

LENGTH = 159 COMBINED P-VALUE = 5.14e-37 E-VALUE = 4.4e-35

DIAGRAM: 64-[2]-11-[1]-32

[2]

1.3e-27

GESVAVVKYSD

+ +++++++++

1 MATKSKKKILKTVSVVDISCGNCIKPTFASIFNFFSKKPKRPSSTYRHCHSSISSATPSSTPLATASVAVEKDSD

[1]

2.9e-19

DPYEDFRRSMVEMIVENGIY YLQLNGKEYHGYIVGAFVQIW

++++++++++++++++++++ +++++++++++++++++++++

76 DPYLDFRQSMLQMILENQIYSKDELRELLQCFLSLNSHYHHGIIVRAFSEIWEDVSSAAASAVEASPLITRHVSR

SlOFP30

LENGTH = 137 COMBINED P-VALUE = 3.30e-36 E-VALUE = 2.8e-34

DIAGRAM: 65-[2]-11-[1]-9

[2]

9.3e-29

GESVAVVKYS

++++++++++

1 MSSKNKKIWNCITSNGTAGCGCSKPKLSEIIQPKPKPRPEPEPNAHSSSTSNSDSPSPTIMPAKIVGSVAVVKDS

[1]

4.4e-17

DDPYEDFRRSMVEMIVENGIY YLQLNGKEYHGYIVGAFVQIW

+++++++++++++++++++++ +++++++ +++++++++ +++

76 DDPFGDFRRSMLQMIMEKEIYSYDDLNELLNCFLQLNSPSHHDIILQAFMEIWNNGKNYIAN

CsOFP6-19c

LENGTH = 171 COMBINED P-VALUE = 4.47e-36 E-VALUE = 3.8e-34

DIAGRAM: 82-[2]-11-[1]-26

[2] [1]

2.2e-29 5.0e-16

GESVAVVKYSDDPYEDFRRSMVEMIVENGIY YLQLNGKEYHGYIVGAFVQIW

++++++++++++++++++ ++++++++++++ ++++++++++++++++++++

76 SPVAILIGDSIAVEKDSDDPYEDFRGSMVEMIVEKRIYSPNGLQELLNCFLHLNSPYHHEIIVKAFTQISNEFES

CmOFP2

LENGTH = 468 COMBINED P-VALUE = 6.53e-35 E-VALUE = 5.6e-33

DIAGRAM: 382-[2]-11-[1]-23

[2] [1]

2.7e-25 1.1e-18

GESVAVVKYSDDPYEDFRRSMVEMIVENGIY YLQLNGKEYHGYIVGAFVQIW

+++++++++++++++++ +++++++++++ +++++++++++++++++ +++

376 MVDDETDLESFAVVKSSFDPQQDFRDSMVEMIMERRISKAEELEELLACYLTLNSDQYHDLIIKVFRQVWFDLNQ

CmOFP15

LENGTH = 170 COMBINED P-VALUE = 9.94e-35 E-VALUE = 8.4e-33

DIAGRAM: 81-[2]-11-[1]-26

[2] [1]

1.4e-28 1.2e-15

GESVAVVKYSDDPYEDFRRSMVEMIVENGIY YLQLNGKEYHGYIVGAFVQIW

++++++++++++++++++++++++++++++ ++++++++++++++++++++

76 PVAILICDSIAVEKDSDDPYEDFRRSMVQMIVEKRIYSPNGLQELLNCFLHLNSPYHHEIILKAFTQISNEFESS

CsOFP5a

LENGTH = 468 COMBINED P-VALUE = 1.74e-34 E-VALUE = 1.5e-32

DIAGRAM: 382-[2]-11-[1]-23

[2] [1]

2.7e-25 1.1e-18

GESVAVVKYSDDPYEDFRRSMVEMIVENGIY YLQLNGKEYHGYIVGAFVQIW

+++++++++++++++++ +++++++++++ +++++++++++++++++ +++

376 TVEDDTDLESFAVVKSSFDPQQDFRDSMVEMIMERRISKAEELEELLACYLTLNSDQYHDLIIKVFRQVWFDLNQ

SlOFP29

LENGTH = 244 COMBINED P-VALUE = 5.05e-34 E-VALUE = 4.3e-32

DIAGRAM: 1-[4]-101-[2]-11-[1]-29

[4]

8.0e-05

KPSFSPMLCRLPRCGNLRTLSIRDENNHNIFNSQRFYNNVDDDMVDEVIE

+ + + + + ++ + +++

1 MGKKMNLGSWQWPSCTHSKTQSFRANHIFKTINSIFLDPSNTDHHHHGVVEIETTPESWFTNSSESASFSTESEE

[2] [1]

8.8e-24 9.2e-19

GESVAVVKYSDDPYEDFRRSMVEMIVENGIY YLQLNGKEYHGYIVGAFVQIW

+++++ ++++++++++++++++++++ ++++ +++++++++++++++++++++

151 PFKESVALALESEDPYLDFKKSMEEMVDTHEIKDWESLQELLQWYLKMNGKNNHGFIIGAFVDLLIGFTPSNCDS

AtOFP4

LENGTH = 305 COMBINED P-VALUE = 6.86e-34 E-VALUE = 5.8e-32

DIAGRAM: 231-[2]-11-[1]-11

[2] [1]

1.7e-23 1.3e-20

GESVAVVKYSDDPYEDFRRSMVEMIVENGIY YLQLNGKEYHGYIVGAFVQIW

+++++ ++++++ +++++++++++ +++++ +++++++++++++++++++++

226 SQNKQILDSFAVIKSSIDPSKDFRESMVEMIAENNIRTSNDMEDLLVCYLTLNPKEYHDLIIKVFVQVWLEVINS

CsOFP13d

LENGTH = 290 COMBINED P-VALUE = 1.14e-33 E-VALUE = 9.7e-32

DIAGRAM: 121-[2]-11-[1]-106

[2]

2.4e-26

GESVAVVKYSDDPYEDFRRSMVEMIVENG

+++++++++++++++++++++++++++++

76 QIEALVRGLRVRQGKRLFLELDETNSIMTTTVAVATVVGGNYQVPFKESVAMAMESKDPYLDFKKSMEEMVEAHE

[1]

6.2e-17

IY YLQLNGKEYHGYIVGAFVQIW

++ +++++++++++++++++++++

151 LKNWKGMERLLSWYLKANGKANHEFIIGAFVDLLVDLAFSASSNFSNNSSSSPSSSSSSSTTTTSSLLCSSTSTF

CsOFP8b

LENGTH = 265 COMBINED P-VALUE = 1.26e-33 E-VALUE = 1.1e-31

DIAGRAM: 193-[2]-11-[1]-9

[2]

1.7e-26

GESVAVVKYSDDPYEDFRRSMVEMIVENGIY

++++++++++ +++ +++ ++++++++++++

151 TLFSSKSRSSDSSASHRRHKSRRRRGCRSRGSEMGVLPLKGKVKDSFAVVKKSSDPYNDFRMSMLEMIVEKQIFS

[1]

8.7e-17

YLQLNGKEYHGYIVGAFVQIW

++++++++++ ++++++++++

226 AKDLEQLLQCFLSLNSHHHHNVILEVFTEIWEALFSDWGS

CmOFP8

LENGTH = 301 COMBINED P-VALUE = 1.88e-33 E-VALUE = 1.6e-31

DIAGRAM: 231-[2]-11-[1]-7

[2] [1]

1.7e-26 1.3e-17

GESVAVVKYSDDPYEDFRRSMVEMIVENGIY YLQLNGKEYHGYIVGAFVQIW

+++++++++ ++++++++++++++++++++ ++++++++++++++++++++

226 TVEGKIRESFAVVKKSADPFEDFKRSMVEMIMEKEMFEEKDLEQLLHCLLSLNDREHHGIIVEAFAEIWQSLFCN

SlOFP6

LENGTH = 391 COMBINED P-VALUE = 2.17e-33 E-VALUE = 1.8e-31

DIAGRAM: 319-[2]-11-[1]-9

[2] [1]

2.0e-25 4.7e-18

GESVAVVKYSDDPYEDFRRSMVEMIVENGIY YLQLNGKEYHGYIV

++++++++++++++++++ ++++++++++++ ++++++++++++++

301 PPRLSVFKKLIPCNVEGKVKESFAIVKKSEDPYEDFKSSMMEMILEKKIFEKNDLEQLLQCFLSLNAKNCHGVIV

GAFVQIW

+++++++

376 EAFSEIWETLFSPNHN

CsOFP13c

LENGTH = 227 COMBINED P-VALUE = 2.59e-33 E-VALUE = 2.2e-31

DIAGRAM: 132-[2]-11-[1]-32

[2]

4.5e-24

GESVAVVKYSDDPYEDFR

+ + +++++++++++++

76 SARVSLSTEFEDDLELVIRGAKSERLIFEPGETNSILEKSRGVEEGGKCEESIRFEGSVVVLMAMESEDPYLDFR

[1]

7.9e-18

RSMVEMIVENGIY YLQLNGKEYHGYIVGAFVQIW

+++++++++++++ +++++++++++++++++++++

151 RSMEEMVECHGIRNWEWLEELLNWYLRMNGMKNHGYILGAFVDLLVDLGGGDGSTDSTSIFSDDLIIQRHDRERC

CmOPF18

LENGTH = 269 COMBINED P-VALUE = 3.88e-33 E-VALUE = 3.3e-31

DIAGRAM: 190-[2]-11-[1]-16

[2]

4.6e-23

GESVAVVKYSDDPYEDFRRSMVEMIVENGIY

+ +++++++++ +++++++++++++++++++

151 EEESEGTEALVNSSMSFSDDVSPVKRSKRATCLRKLEGKMGKSFVQVKRSKEPQEDFKRSMVQMILEKEIFETKG

[1]

5.9e-20

YLQLNGKEYHGYIVGAFVQIW

+++++++++++++++++++++

226 LEELLQCYLTLNSPEYHRIIVGAFSEVWEFLFCDSHSNKAVQCD

CmOFP9

LENGTH = 227 COMBINED P-VALUE = 6.82e-33 E-VALUE = 5.8e-31

DIAGRAM: 132-[2]-11-[1]-32

[2]

4.5e-24

GESVAVVKYSDDPYEDFR

+ + +++++++++++++

76 ARVSLSTEFEDDLELVIQGAKSERLIFEPGETNSILDQSRGGKSEGGKCESILRFEGSVVVLMAMESEDPYLDFR

[1]

7.9e-18

RSMVEMIVENGIY YLQLNGKEYHGYIVGAFVQIW

+++++++++++++ +++++++++++++++++++++

151 RSMEEMVECHGIRNWEWLEELLNWYLRMNGMKNHGYILGAFVDLLVDLGGADGSTDSTSIFSDDLIIQPHDRERC

CsOPF13a

LENGTH = 234 COMBINED P-VALUE = 1.04e-32 E-VALUE = 8.8e-31

DIAGRAM: 25-[4]-42-[2]-11-[1]-54

[4]

6.0e-05

KPSFSPMLCRLPRCGNLRTLSIRDENNHNIFNSQRFYNNVDDDMVDEVIE

+ ++ ++ ++ ++ + + + +

1 MLTKKKKMMMMMRLPSLFKYLAIDDKSTFPWPSCRQPRTLSFRTTSAAVATATDSSDSFFTLSSESSGSLSTVSE

[2]

1.1e-22

GESVAVVKYSDDPYEDFRRSMVEMIVENGIY

++ ++ +++++++ ++++++++++++++++

76 SSGGDPIERMIRDLRSTKRLHFEPTGKSSSIVEDDTVSHPLKEGTTVMSMDSDDPYSDFRKSMEEMVEAHGMKDW

[1]

7.9e-18

YLQLNGKEYHGYIVGAFVQIW

+++++++++++++++++++++

151 ESLEELLNWYLRVNGKKNHGFILGAFVDLLVSLAMASSSSSSSCSSSLCCYSSSSSSSSLPCVSSSMEIEEISSL

AtOFP8

LENGTH = 221 COMBINED P-VALUE = 1.44e-32 E-VALUE = 1.2e-30

DIAGRAM: 152-[2]-11-[1]-6

[2] [1]

3.6e-28 1.4e-14

GESVAVVKYSDDPYEDFRRSMVEMIVENGIY YLQLNGKEYHGYIVGAFVQIW

+++++++++++++++++ ++++++++++++ +++++++++++++++++ +++

151 SKAESFAVVKKSKDPYEDFRTSMVEMIVERQIFAPAELQQLLQCFLSLNSRQHHKVIVQVFLEIYATLFSP

CsOVATE

LENGTH = 301 COMBINED P-VALUE = 1.78e-32 E-VALUE = 1.5e-30

DIAGRAM: 232-[2]-11-[1]-6

[2] [1]

8.3e-26 2.7e-17

GESVAVVKYSDDPYEDFRRSMVEMIVENGIY YLQLNGKEYHGYIVGAFVQIW

+++++++++ ++++++++++++++++++++ ++++++++++++++++++++

226 CTVEGKIRESFAVVKKSADPFEDFKRSMMEMIMEKEMFEEKDLEQLLHCLLSLNDREHHGIIVEAFSEIWQSLFC

CmOFP5

LENGTH = 235 COMBINED P-VALUE = 1.82e-32 E-VALUE = 1.5e-30

DIAGRAM: 25-[4]-42-[2]-11-[1]-55

[4]

6.2e-05

KPSFSPMLCRLPRCGNLRTLSIRDENNHNIFNSQRFYNNVDDDMVDEVIE

+ ++ ++ ++ ++ + + + +

1 MLTKKKKMMMMMRLPSLFKILAIDDKSTFPWPSCRQPRTLSFRTTSAAVATATDSSGSFFTLSSESSGSLSTVSE

[2]

2.5e-22

GESVAVVKYSDDPYEDFRRSMVEMIVENGIY

++ ++ +++++++ ++++++++++++++++

76 SSGGDPIERMIRDLRSTKRLHFEPTGKSSSIVEDETVSHPLKEGTTVMSMDSNDPYSDFRKSMEEMVEAHGMKDW

[1]

7.9e-18

YLQLNGKEYHGYIVGAFVQIW

+++++++++++++++++++++

151 ESLEELLNWYLRVNGKKNHGFILGAFVDLLVSLAMASSSSSSSSSSSSLCCYSSSSSSSSLPCVSSSMEIEEISS

CmOFP21

LENGTH = 289 COMBINED P-VALUE = 2.20e-32 E-VALUE = 1.9e-30

DIAGRAM: 123-[2]-11-[1]-103

[2]

2.4e-26

GESVAVVKYSDDPYEDFRRSMVEMIVE

+++++++++++++++++++++++++++

76 EALVRGLRVRQGKRLFLELDETNSIMTATATTTVAVATVGAGNYHVPFKESVAMAMESKDPYLDFKKSMEEMVEA

[1]

3.0e-16

NGIY YLQLNGKEYHGYIVGAFVQIW

++++ ++++++ ++++++++++++++

151 HELKDWKGMERLLSWYLKANGNANHEFIIGAFVDLLVDLAFAASSNLSNNSSSSPSSSSSSTTTTSSLLCSSSSS

CmOFP14

LENGTH = 241 COMBINED P-VALUE = 3.48e-32 E-VALUE = 3e-30

DIAGRAM: 169-[2]-11-[1]-9

[2] [1]

2.0e-25 8.7e-17

GESVAVVKYSDDPYEDFRRSMVEMIVENGIY YLQLNGKEYHGYIV

+++++++++ +++ +++ ++++++++++++ ++++++++++ +++

151 TLFSSKSRSSDSSKHLTDDRNSFAVVKKSSDPYNDFRMSMLEMIVEKQIFSAKDLEQLLQCFLSLNSHHHHNVIL

GAFVQIW

+++++++

226 EVFTEIWEALFSDWGS

CsOFP10

LENGTH = 270 COMBINED P-VALUE = 3.67e-32 E-VALUE = 3.1e-30

DIAGRAM: 191-[2]-11-[1]-16

[2]

2.8e-22

GESVAVVKYSDDPYEDFRRSMVEMIVENGIY

+ +++++++++ +++++++++++++++++++

151 GEKESEETDALVNSSISFSDDVSPVKRSKRALYLRKLEGKMGKSFVQVKRSKEPQEDFKRSMAQMILEKEIFEIK

[1]

5.9e-20

YLQLNGKEYHGYIVGAFVQIW

+++++++++++++++++++++

226 GLEELLQCYLTLNSPEYHRIIVGAFSEVWEFLFYDSHLNKAVQRD

SlOFP9

LENGTH = 266 COMBINED P-VALUE = 5.37e-32 E-VALUE = 4.6e-30

DIAGRAM: 184-[2]-11-[1]-19

[2]

2.3e-24

GESVAVVKYSDDPYEDFRRSMVEMIVENGIY

++++++++++++++ +++ ++ +++++++++

151 RKKNNNNTKVRRLRRYLSNSLKDSMMPCMADGKVNESFAIVKRSVDPYDDFKNSMKEMIMEKEMFEAEDLEQLLL

[1]

1.1e-17

YLQLNGKEYHGYIVGAFVQIW

++++++++++ ++++++++++

226 CFLSLNSRHHHAIIVEAFTEIWEELFGKSSKSMDLKLPRFQ

SlOFP3

LENGTH = 298 COMBINED P-VALUE = 5.53e-32 E-VALUE = 4.7e-30

DIAGRAM: 16-[4]-73-[2]-11-[1]-96

[4]

3.7e-08

KPSFSPMLCRLPRCGNLRTLSIRDENNHNIFNSQRFYNNVDDDMVDEVIE

+ ++ ++ ++ + +++ + + ++ + +

1 MKLSSLFKNSSQNSSSTTTTTPWPWSLPTCGKPKTLSFRLEKNQHNIYNSTFHLDDINDTTSCSFDDFFSEIDET

[2]

4.9e-20

GESVAVVKYSD

++ + ++++

76 SSSSTTTINGQDCIEKVIKGLRLEKERLFFEPEETSSILDFQENKNISITSSNININVVDEGNIISFVPMGLDSN

[1]

1.9e-17

DPYEDFRRSMVEMIVENGIY YLQLNGKEYHGYIVGAFVQIW

++++++++++++++++++++ +++++++ +++++++++++++

151 DPFVDFRKSMEEMVEAYEIKDWENLEELLTCYLKVNCKSNHGYIVGAFVDLLVNLATFSDNNNNVGVDIGAGVGA

AtOFP7

LENGTH = 315 COMBINED P-VALUE = 8.37e-32 E-VALUE = 7.1e-30

DIAGRAM: 224-[2]-11-[1]-28

[

3

G

+

151 EEETDRESLLPSSTNLSPEYSSSELPRVTRRPRQLLKKAVIEEESESSSPPPSPARLSSFVQRLMPCTMAAAVMV

2] [1]

.2e-26 3.7e-16

ESVAVVKYSDDPYEDFRRSMVEMIVENGIY YLQLNGKEYHGYIVGAFVQIW

+++++++++++++++++ ++++++++++++ +++++++ +++ +++++++++

226 EGVAVVKRSEDPYEDFKGSMMEMIVEKKMFEVAELEQLLSCFLSLNAKRHHRAIVRAFSEIWVALFSGGSGGGRR

AtOFP5

LENGTH = 349 COMBINED P-VALUE = 1.41e-31 E-VALUE = 1.2e-29

DIAGRAM: 280-[2]-11-[1]-6

[2]

3.8e-26

GESVAVVKYSDDPYEDFRRS

++++++++++ +++++++ +

226 RELNRIGTKGNNKVRVFSPRASEKCRVKAIEDLKKAKQRAREHELLIETADGGMENESFAVVKCSSDPQKDFRDS

[1]

8.9e-15

MVEMIVENGIY YLQLNGKEYHGYIVGAFVQIW

++++++++++ +++++ +++++ ++ ++ ++

301 MIEMIMENGINHPEELKELLVCYLRLNTDEYHDMIISVFQQVHNDFNFH

SlOFP19

LENGTH = 211 COMBINED P-VALUE = 5.44e-31 E-VALUE = 4.6e-29

DIAGRAM: 125-[2]-15-[1]-19

[2]

2.3e-24

GESVAVVKYSDDPYEDFRRSMVEMI

++++++ ++++++ ++++++++++

76 TNCTFTSFEDSDYTNIPDFSNIFASQRFFFSSPGNSNSIIDFPPENPKVVTGGVAVQTYSPDPYSDFRRSMQEMV

[1]

1.2e-16

VENGIY YLQLNGKEYHGYIVGAFVQIW

+++++ +++++++++++++++++++++

151 EAHELTNVKANWGFLHELLLCYLNLNPKHTHKYIIRAYSDLVVSLMSMDDSEKKTEGIARP

SlOFP5

LENGTH = 377 COMBINED P-VALUE = 1.18e-30 E-VALUE = 1e-28

DIAGRAM: 90-[3]-173-[2]-11-[1]-20

[3]

5.6e-05

MGNYKFRFSDMMPNAWFYKLKDMGKAKRHKN

+ ++ +++ + + + ++++

76 NPLWCGECDQNSKSSLGEENHKFNDMVSRKISEKPKNEAEFSNRKRNSVKDEKLRKLSRKALEERIAENAREEVT

[2]

3.2e-2

GESVAV

+++++

226 EMSEKSGCQQRKSVYINQKRRRKHGIKVRAYSPRTAKMECRIKALEDMKKARMKTRHETKESFTGDRTVFDSYAI

[1]

0 2.1e-19

VKYSDDPYEDFRRSMVEMIVENGIY YLQLNGKEYHGYIVGAFVQIW

++++++++ +++ ++++++ ++++ +++++++++++++++++ +++

301 MKSSFDPFSDFRDSMIEMITQRGIKSSEELEELLACYLTLNCDEYHDIIIKVFRQVWFELNQINIGEELQKCCCS

AtOFP13

LENGTH = 260 COMBINED P-VALUE = 1.55e-29 E-VALUE = 1.3e-27

DIAGRAM: 11-[4]-83-[2]-13-[1]-51

[4]

7.4e-05

KPSFSPMLCRLPRCGNLRTLSIRDENNHNIFNSQRFYNNVDDDMVDEVIE

+ + + + +++ + ++

1 MGKKKMKLSSLFKGGAGGLLAVPLCYNAKTLSFRVGDDMIKTVNSVFFDHHHNNNNGGDLLEAETPESWFTNSSE

[2]

7.2e-2

GESVAV

+ ++++

76 TASHSTESDQDLDAESLEMVVRGVVRSERLFFDPGVTSSILEEIEEKSKSDLKSKETVAVGEDRSTPIEEISVAV

[1]

2 9.4e-16

VKYSDDPYEDFRRSMVEMIVENGIY YLQLNGKEYHGYIVGAFVQIW

+++++++++++++++++++ ++ ++++++++ +++++ ++++++

151 AMESEDPYGDFRRSMEEMVTSHGELAKDWESLESMLAWYLRMNGRKSHGVIVSAFVDLLSGLSDSGAGITSASVS

CmOFP3

LENGTH = 176 COMBINED P-VALUE = 1.67e-29 E-VALUE = 1.4e-27

DIAGRAM: 101-[2]-11-[1]-12

[2] [1]

4.1e-23 6.1e-15

GESVAVVKYSDDPYEDFRRSMVEMIVENGIY YLQLNGK

+++++++++++++ ++++ ++ ++++++++ + +++++

76 DQMIREKREVRNGKERKKQRSEDTKFVVMVAMEKCSDDPKEDFRVSMTEMILANRIEEPKDLRNLLNYYISMNSD

EYHGYIVGAFVQIW

++++++++++++++

151 ECHGVIFEVFHEVCSNLFLACKRHYW

SlOFP10

LENGTH = 229 COMBINED P-VALUE = 2.80e-29 E-VALUE = 2.4e-27

DIAGRAM: 114-[2]-11-[1]-52

[2]

1.1e-21

GESVAVVKYSDDPYEDFRRSMVEMIVENGIY

+++++ +++++++++++++++ ++++ +++

76 SHEEIIKGARSERLFFEQVATSSIFQEPQEENQENDLPFKESVILAMESKDPYLDFKKSMKEMVESQGIKDWDNL

[1]

2.1e-16

YLQLNGKEYHGYIVGAFVQIW

++++++ ++++++++++++++

151 QELLACYLKLNGEVNHGFVLGAFVDLLVELVIPTTPSTNSDNSITSYSSVASSSFSCPSSPLSSLGHKETEEQEN

CmOFP6

LENGTH = 284 COMBINED P-VALUE = 3.76e-29 E-VALUE = 3.2e-27

DIAGRAM: 146-[2]-11-[1]-75

[2]

1.0e

GESV

++++

76 DYSSSLHTNSSDSVSATNSTPAMDSEESLETVVRGARSERLFFEPDDTSSILEKSKPIESVETDELPRSGFKESL

[1]

-20 3.4e-17

AVVKYSDDPYEDFRRSMVEMIVENGIY YLQLNGKEYHGYIVGAFVQIW

++ +++++++++++++ ++++ ++ + +++++++++++++++++++++

151 IVSIESENPYEDFRKSMGEMVESHGVKDWDGLEELLGWYLKANWKNNHRFIIGAFVDLLIHILLASSSSSSSTST

CsOFP12-16c

LENGTH = 197 COMBINED P-VALUE = 7.09e-29 E-VALUE = 6e-27

DIAGRAM: 105-[2]-15-[1]-25

[2]

6.4e-22

GESVAVVKYSDDPYEDFRRSMVEMIVENGIY

+++ +++++++++ +++++++++++++++

76 RFFFSSPGRSNSIFEYSSCSRRQQPHDVLVSEGHRIRKYSMDPYADFRRSMQEMVEARELEDVRSDSEFLRELLS

[1]

7.8e-17

YLQLNGKEYHGYIVGAFVQIW

+++++++++++++++++++++

151 CYLRLNPKNTHKFIVKAFSDLVLSLLASSSPTPAPASIARRKVVTSR

CsOFP13b

LENGTH = 277 COMBINED P-VALUE = 1.37e-28 E-VALUE = 1.2e-26

DIAGRAM: 134-[2]-11-[1]-80

[2]

1.0e-20

GESVAVVKYSDDPYED

++++++ ++++++++

76 SVSATNSTPAVDSEESLETVVRGARSERLFFEPDDTSSILEKSKSIDSVETELLPKSGFKESLIVSIESENPYED

[1]

3.4e-17

FRRSMVEMIVENGIY YLQLNGKEYHGYIVGAFVQIW

+++++ ++++ ++ + +++++++++++++++++++++

151 FRKSMGEMVESHGVKDWDGLEELLGWYLKANWKNNHRFIIGAFVDLLIHILLASSSSSSSSTSTSTSSSSSSSLC

CsOFP8a

LENGTH = 239 COMBINED P-VALUE = 1.42e-28 E-VALUE = 1.2e-26

DIAGRAM: 170-[2]-11-[1]-6

[2] [1]

1.9e-23 1.9e-13

GESVAVVKYSDDPYEDFRRSMVEMIVENGIY YLQLNGKEYHGYI

++++++++ + ++++++++++++++++++++ + +++++++++++

151 VSRRRHRRRHGRRRPERKMRDGFFAVVKNSSNPYMDFKASMAEMVVEKKIFGGKELEELLQCFISLNSRHYHKVI

VGAFVQIW

+++++++

226 FEVYSEIKEALFFL

CmOFP20

LENGTH = 237 COMBINED P-VALUE = 2.28e-28 E-VALUE = 1.9e-26

DIAGRAM: 168-[2]-11-[1]-6

[2] [1]

1.5e-24 1.9e-13

GESVAVVKYSDDPYEDFRRSMVEMIVENGIY YLQLNGKEYHGYIVG

++++++++ + ++++++++++++++++++++ + +++++++++++++

151 RRHRQRHGRRRPPERKMRDGFFAVVKNSSDPYKDFKASMAEMVVEKKIFGGKELEELLQCFISLNSRHYHKVIFE

AFVQIW

+++++

226 VYSEIKEALFFL

CsOFP5b

LENGTH = 168 COMBINED P-VALUE = 3.58e-28 E-VALUE = 3e-26

DIAGRAM: 87-[2]-11-[1]-18

[2] [1]

4.1e-23 1.0e-12

GESVAVVKYSDDPYEDFRRSMVEMIVENGIY YLQLNGKEYHGYIVGAFVQIW

+++++++++++++ ++++ ++ ++++++++ + +++++++++++++++++

76 ERRKQRSEDTKFVVMVAMEKCSDDPKEDFRVSMTEMILANRIEEPKDLRNLLNYYISMNSDECHGVIFEVFHEKE

CmOFP7

LENGTH = 263 COMBINED P-VALUE = 3.62e-27 E-VALUE = 3.1e-25

DIAGRAM: 148-[2]-23-[1]-40

[2

5.

GE

++

76 LANDIGVADPDAYVAVDFITAFTSHRFFFSSPGSSNSIIESTTPTTTESTTTMSLSSEYSARYEGNDDELMIFNN

] [1]

1e-22 4.2e-15

SVAVVKYSDDPYEDFRRSMVEMIVENGIY YLQLNGKEYHGYIVGAFVQIW

++++ +++++++++++++++++++++ + ++ ++++++++ ++++++++

151 SHVIPTYSPDPYMDFRRSMQEMVEAREKMTTAATTTTMKKSSWEFLHELLLCYLALNPKATHKHILKAFADVATV

CsOFP12-16a

LENGTH = 278 COMBINED P-VALUE = 1.10e-26 E-VALUE = 9.3e-25

DIAGRAM: 153-[2]-24-[1]-49

[2] [1]

2.0e-21 3.8e-15

GESVAVVKYSDDPYEDFRRSMVEMIVENGIY YLQLNGKEYHGYIVGAF

++++++ +++++++++++++++++++++ + ++ ++++ +++ +++++

151 MIFNNSHVIPTYSPDPYMDFRRSMQEMMEAREKMTTAVATTTTMKKSSWEFLHELLLCYLALNPKTTHKHILKAF

VQIW

+++

226 ADIATVIKPPLAMKETEEEENVDREKGESMVDDRGAGGGGCECEMSGQQNDRD

CmOFP13+1

LENGTH = 154 COMBINED P-VALUE = 3.58e-26 E-VALUE = 3e-24

DIAGRAM: [3]-123

[3]

4.9e-33

MGNYKFRFSDMMPNAWFYKLKDMGKAKRHKN

+++++++++++++++++++++++++++++++

1 MRNHKFRFSDMIPNAWFYKLKEIGGASRPKSFRSNKNPHHPPPPPPPSKHKQPPPPPPHSRSRKSYYFTRQLESN

CsOFP12-16b

LENGTH = 205 COMBINED P-VALUE = 2.07e-25 E-VALUE = 1.8e-23

DIAGRAM: 111-[2]-16-[1]-26

[2]

2.9e-20

GESVAVVKYSDDPYEDFRRSMVEMIVENGIY

++++ +++ +++++++++++++++++++

76 AAVASHRFFFSSPGCSNSIFDSSPDTHHSTAVSAAVHGGVEVRKVSMDPFVDFRASMQEMVEARDRPVDVRRDWE

[1]

7.4e-15

YLQLNGKEYHGYIVGAFVQIW

++++++ +++++++++++++

151 YLQELLLCYLQINPVDTHKFILRAFSDLVVYLLESSPESFSDRRIRPHNINSNSW

AtOFP10

LENGTH = 196 COMBINED P-VALUE = 3.73e-25 E-VALUE = 3.2e-23

DIAGRAM: 94-[2]-11-[1]-39

[2] [1]

3.1e-21 6.5e-13

GESVAVVKYSDDPYEDFRRSMVEMIVENGIY YLQLNGKEYHGYIV

++++++++++++++++ ++++ ++++++ + ++ +++ + +++

76 YTPGPPVSPTVLRSPCPKIDESVAMAKESINPFEDYKKSMNQMIEERYIETESELKELLRCFLDINPSPQHNLIV

GAFVQIW

+++++++

151 RAFVDVCSHLQPPHDRRGKSLGRLLRLYVNNPLDNNDDDSHQTSSK

CmOFP1

LENGTH = 205 COMBINED P-VALUE = 4.30e-25 E-VALUE = 3.7e-23

DIAGRAM: 111-[2]-16-[1]-26

[2]

2.9e-20

GESVAVVKYSDDPYEDFRRSMVEMIVENGIY

++++ +++ +++++++++++++++++++

76 AAVASHRFFFSSPGCSNSIFDSSPDTHHSAAVSAAVHGGVEVRKVSMDPFVDFRASMQEMVEARDRPVDVRRDWE

[1]

1.3e-14

YLQLNGKEYHGYIVGAFVQIW

++++++ +++++++++++++

151 YLQDLLLCYLRINPVDTHKFILRAFSDLVVYLLECSPESFSDRRLRPHNINSNSW

SlOFP15

LENGTH = 168 COMBINED P-VALUE = 6.26e-25 E-VALUE = 5.3e-23

DIAGRAM: 91-[2]-11-[1]-14

[2] [1]

4.4e-20 2.5e-13

GESVAVVKYSDDPYEDFRRSMVEMIVENGIY YLQLNGKEYHGYIVGAF

++++++++++++ ++++++ ++++ ++++ + ++++ ++ +++++++

76 MRRRRRRAERDEKTKFIVMIAMEKSSYDPREDFRESIEQMIIANRICDPKDLRRLLNYYVSMNAEEYRGVILEVF

VQIW

++++

151 HQVCTTFFLSCKQPSSQV

AtOFP12

LENGTH = 226 COMBINED P-VALUE = 7.06e-25 E-VALUE = 6e-23

DIAGRAM: 148-[2]-15-[1]-11

[2

7.

GE

++

76 STAANSSSSSASYDDSDNYGFAPDDDSPPPDLTAVLASRRFFFSSPGCSNSITDSPDLRCRDNYDTATRLLTGGT

] [1]

2e-18 1.1e-16

SVAVVKYSDDPYEDFRRSMVEMIVENGIY YLQLNGKEYHGYIVGAFVQIW

++ + +++++ ++++++++++++ ++++++ +++++++++++++

151 AVKHYVQSPDPYNDFRRSMQEMIDAVTNAGDLRRYEFLHELLLSYLSLNAADTHKFIIRAFADILVSLLSDGHRI

AtOFP14

LENGTH = 294 COMBINED P-VALUE = 9.76e-25 E-VALUE = 8.3e-23

DIAGRAM: 189-[2]-16-[1]-37

[2]

2.7e-19

GESVAVVKYSDDPYEDFRRSMVEMIVENGIY

++++++ + + ++++++++++++++ ++++

151 DLLRTERLSPPPGSSEGRPSMETTSTSSERQSRSTLVLPENCIAVLRYTDEPQEDFRQSMVEMMESKLGMRESEV

[1]

9.8e-15

YLQLNGKEYHGYIVGAFVQIW

++ +++++ +++++ ++++++

226 DWDLMEELLFCYLDLNDKKSHKFILSAFVDLIIALREKEKRITRKGHVRSLSTRAARDRLRKRMIMSDN

SlOFP7

LENGTH = 275 COMBINED P-VALUE = 2.20e-23 E-VALUE = 1.9e-21

DIAGRAM: 48-[4]-81-[2]-15-[1]-29

[4]

1.8e-05

KPSFSPMLCRLPRCGNLRTLSIRDENN

++++

1 MPKQLQKSLSDYLTKKKKKATAQQTTNSANKTLSSSTSWLLRGCRHPKTPSFSAVDRKEKNVQGENEAATLADVD

HNIFNSQRFYNNVDDDMVDEVIE

++ + +++ ++++

76 RFVFENFKSFYYKDDDNEAEIVENPNSLSESPRHIIPPLNHTGSRRFFIAPGSSSSLIEEARTSMTVSDDTGSTS

[2]

5.0e-18

GESVAVVKYSDDPYEDFRRSMVEMIVENGIY

++++ + +++ ++ ++++++++++++++

151 AITITTVTNTNSNELSAISTEYSKETLNANDFITLVTYSPSPYDDFRQSMQEMMEARLKDQGKINWEFMEELLFC

[1]

5.1e-13

YLQLNGKEYHGYIVGAFVQIW

++ +++++ +++++ ++++ +

226 YLDLNDKKSYKYILSAFVDQIVILRENSGRVPAISRNVRPLDGELNQRDT

AtOFP15

LENGTH = 261 COMBINED P-VALUE = 2.38e-23 E-VALUE = 2e-21

DIAGRAM: 106-[2]-12-[1]-91

[2] [

3.6e-19 4

GESVAVVKYSDDPYEDFRRSMVEMIVENGIY Y

++++ +++++++ +++++++++++++ ++ +

76 RSSERLIFESKGETNSILEEATSKREEEDEEEGFMLFSLESDDPYSDFKRSMEEMVEAHALHHDWKSLEKLLLQF

1]

.5e-14

LQLNGKEYHGYIVGAFVQIW

++++++ ++++++++++++

151 LKVNAKTSHRYIFAAFVDLLMNLALDTKKAIINNDISKDDGVSASRAAAAGEASTSCCNSMTLGESPSSPLSFYT

AtOPF16

LENGTH = 244 COMBINED P-VALUE = 2.96e-23 E-VALUE = 2.5e-21

DIAGRAM: 161-[2]-20-[1]-11

[2] [1]

1.8e-17 2.1e-15

GESVAVVKYSDDPYEDFRRSMVEMIVENGIY YLQLNGKEYHGYI

++++ + ++++++ ++++++++++++ ++++++ +++++

151 VTTTTTRLISGGTAVTQHVDSPDPLTDFRRSMQEMIDAAIDAGELSRDPNDGYDFLDELLLTYLSLNPADTHKFV

VGAFVQIW

++++++++

226 IRAFSDILVSLLSEERRIC

AtOFP11

LENGTH = 182 COMBINED P-VALUE = 4.77e-23 E-VALUE = 4.1e-21

DIAGRAM: 99-[2]-16-[1]-15

[2] [1]

1.6e-17 4.2e

GESVAVVKYSDDPYEDFRRSMVEMIVENGIY YLQL

++++ +++++++++ +++ ++++++ + ++ +

76 FSTNRREEEEEDETTTSVSKLLSGGTAIMKHIESPDPYRDFGRSMREMVEARDLTRDVVADREYLHELLFCYLYL

-15

NGKEYHGYIVGAFVQIW

++++++++++ ++++ +

151 NPKHTHRFIVSAFADTLLWLLSPSPSPEHFLS

CsOFP14

LENGTH = 272 COMBINED P-VALUE = 8.49e-23 E-VALUE = 7.2e-21

DIAGRAM: 178-[2]-15-[1]-27

[2] [

6.6e-18 3

GESVAVVKYSDDPYEDFRRSMVEMIVENGIY Y

+++++ +++++ + +++++++++++++ + +

151 SHTESSENAGSSSSSLIGEDRGKDLKLPSDCIAILRKSPNPSEEFRRSMQEMMDAHLKQHEKVDWEFMEELLFCY

1]

.2e-14

LQLNGKEYHGYIVGAFVQIW

++++ ++ +++++ ++++++

226 LNLNEKKSYKYILNAFVDLIVILRQKAEEAPAKPRTVRSVRMVRRMI

CmOFP10

LENGTH = 298 COMBINED P-VALUE = 4.56e-22 E-VALUE = 3.9e-20

DIAGRAM: 204-[2]-15-[1]-27

[2]

4.6e-17

GESVAVVKYSDDPYEDFRRSM

+++++ +++++ + +++++

151 SPVDSYGGSHRFFFSPDLSGSDLPDDSHTESSENAGSSSSSLIGEDRGKDLKLPSDCIAILRKSPNPSEEFRRSM

[1]

2.4e-14

VEMIVENGIY YLQLNGKEYHGYIVGAFVQIW

+++++ ++ + ++++++++ +++++ ++++++

226 QEMMDGHLKHHEKVDWEFMEELLFCYLNLNDKKSYKYILNAFVDLIVILRQKAEEEPAKPRTVRSVRMVRRMI

AtOFP18

LENGTH = 282 COMBINED P-VALUE = 1.82e-20 E-VALUE = 1.5e-18

DIAGRAM: 133-[2]-12-[1]-85

[2]

2.5e-16

GESVAVVKYSDDPYEDF

++ + +++++++ ++

76 YSSFSSTSHAIENPPEIESIENVIKGLKSSKRLIFERRGTSNSILEEATKRDDHEEEEDGLMLLSLESNDPYTDF

[1]

1.8e-13

RRSMVEMIVENGIY YLQLNGKEYHGYIVGAFVQIW

+ +++ +++ + ++ +++++ ++ ++++++++++++

151 KNSMEKMVEVHVLHHDWISLEKLLFWFLKVNVKASHRYIFAAFVDLVLNLAVGPSKDVAGEPNSDVVVEDSLSSS

SlOFP12

LENGTH = 158 COMBINED P-VALUE = 2.22e-18 E-VALUE = 1.9e-16

DIAGRAM: 57-[2]-16-[1]-33

[2]

5.6e-14

GESVAVVKYSDDPYEDFR

++++ +++ +++ +++

1 MTRKYDQCLVDNMFGPFPESCCPDEALEMAKQALATRRLSFEENESCSVLSMVGFPFKDCLLLAVETENPKMDFL

[1]

3.5e-12

RSMVEMIVENGIY YLQLNGKEYHGYIVGAFVQIW

++++++ ++ + +++++ +++ ++++++ +++

76 HSMEQMTKVYGAQRGDMVDWEFMEELLTWFLKINNMKNQHFIVAAFIDLCLGGHVQDVEPVENVEPLTDDIVNVI

SlOFP11

LENGTH = 83 COMBINED P-VALUE = 1.76e-11 E-VALUE = 1.5e-09

DIAGRAM: 31-[2]-21

[2]

2.3e-16

GESVAVVKYSDDPYEDFRRSMVEMIVENGIY

++ ++++++++ +++++ +++ +++

1 MVNYKGGKLLKHQRNRVSFSAKLPEDVRGAFADSTCVVKYSMDPLTDIKESIKEMVKNVGIKDWKEMEELVYCYI

SlOFP13

LENGTH = 188 COMBINED P-VALUE = 2.89e-08 E-VALUE = 2.5e-06

DIAGRAM: 104-[2]-12-[1]-20

[2] [1]

6.1e-07 4.5

GESVAVVKYSDDPYEDFRRSMVEMIVENGIY YLQ

++ ++ + ++ + + ++++++ + + + +

76 ALKSFSGHIKAPVPSPITPAYARLSGATKKEVVIFQDDVEDACRSFENYLAEMIVEEGKMRDIMDVEELLYCWKN

e-09

LNGKEYHGYIVGAFVQIW

+ +++ +++ +++++++

151 LKSPVFIDLVCRFYGELCKDLFSHTYKDDINSPQKIMQ

SlOFP8

LENGTH = 229 COMBINED P-VALUE = 1.59e-06 E-VALUE = 0.00013

DIAGRAM: 184-[1]-24

[1]

2.2e-10

YLQLNGKEYHGYIVGAFVQIW

+ ++++++ +++ +++ + +

151 SKGSLTLLKKMEELEMVEGEDMDHVLDIEEVLQCYTLLNSPVYVDIVDRFFMDMYTEFSIRKPSGSVNSSMRRLG

CmOFP16

LENGTH = 189 COMBINED P-VALUE = 7.98e-06 E-VALUE = 0.00068

DIAGRAM: 107-[2]-12-[1]-18

[2]

4.4e-05

GESVAVVKYSDDPYEDFRRSMVEMIVENGIY

+++ + ++ + + ++++ + +

76 HLRSSESVRSDNECREKLLFPSPMIRGRKVAAGTSWEEKEEVEDACKSFENYLVEMIIEEGKVRDLMDVEELLYC

[1]

1.1e-08

YLQLNGKEYHGYIVGAFVQIW

+ ++ +++ +++ +++++++

151 WRNLKCPVFVDLVSRFYGELCKDLFSSHIQAFTPNFQPK

AtOFP17

LENGTH = 195 COMBINED P-VALUE = 2.69e-04 E-VALUE = 0.023

DIAGRAM: 167-[1]-7

[1]

1.9e-08

YLQLNGKEYHGYIVGAFVQIW

+ ++ +++ +++ +++++++

151 EEGKIDDLMDIEELLFCWKNLKSPVFIELVSRFYGELCRDLFSGE

********************************************************************************

CPU: noble-meme.grid.gs.washington.edu

Time 0.060 secs.

mast -oc . -nostatus meme.xml sequences.fa
