# Supplementary material for: Targeted Editing and Phenotypic Profiling of CmOFP13 Mutants Reveal Its Role in Melon Fruit Morphogenesis
Source: Physiol Plant. 2025 Nov 29;177(6):e70641. doi: 10.1111/ppl.70641 (PMC12664293; doi:10.1111/ppl.70641)
Supplement: Supplementary file 8 — File S8: ppl70641‐sup‐0008‐FileS8.docx. [file PPL-177-e70641-s001.docx]

**Targeted editing and phenotypic profiling of *CmOFP13* mutants reveal its role in melon fruit morphogenesis**

Carlos MAYOBRE, María José GONZALO, Montserrat VERGÉS, Guillem GUARDIA-BERSABÉ, Dídac JIMÉNEZ-SÁNCHEZ, Antonio Jose MONFORTE, Jordi GARCIA-MAS, Marta PUJOL

**Supplementary File S8.** ANOVA, Kruskal-Wallis and Welch-ANOVA results full tables

**Supplementary File S8A.** ANOVA results for fruit shape index comparing location effect (TM vs IBMCP).

|  | Df | Sum Sq | Mean Sq | F value | Pr(>F) |
| --- | --- | --- | --- | --- | --- |
| Genotype | 2 | 0.21767 | 0.10884 | 55.148 | **4.28e-12***** |
| Location | 1 | 0.00005 | 0.00005 | 0.024 | 0.879 |
| Genotype*Location | 1 | 0.00046 | 0.00046 | 0.232 | 0.632 |
| Residuals | 39 | 0.07697 | 0.00197 |  |  |

**Supplementary File S8B.** ANOVA results for fruit shape index comparing season effect (autumn 2023 vs summer 2024).

|  | Df | Sum Sq | Mean Sq | F value | Pr(>F) |
| --- | --- | --- | --- | --- | --- |
| Genotype | 2 | 0.23301 | 0.11651 | 63.532 | **5.06e-15***** |
| Season | 1 | 0.03994 | 0.03994 | 21.782 | **2.00e-05***** |
| Genotype*Season | 2 | 0.00443 | 0.00222 | 1.208 | 0.307 |
| Residuals | 55 | 0.10086 | 0.00183 |  |  |

**Supplementary File S8C.** ANOVA results for fruit area comparing TM and CRAG melons.

|  | Df | Sum Sq | Mean Sq | F value | Pr(>F) |
| --- | --- | --- | --- | --- | --- |
| Genotype | 2 | 140 | 70 | 0.414 | 0.665 |
| Season-Location | 1 | 13028 | 13028 | 76.879 | **5.10e-10***** |
| Genotype*Season-Location | 2 | 224 | 112 | 0.662 | 0.523 |
| Residuals | 32 | 5423 | 169 |  |  |

**Supplementary File S8D.** Welch-ANOVA results for fruit morphology comparing location effect (TM vs IBMCP).

|  | Genotype | Location | Line*Location |
| --- | --- | --- | --- |
| Pr(>WJ) length | **0.004907566**** | 0.4346025 | 0.3839009 |
| Pr(>WJ) width | 0.2138837 | 0.2975861 | 0.1397055 |

**Supplementary File S8E.** Welch-ANOVA results for fruit morphology comparing season effect (autumn 2023 vs summer 2024.

|  | Genotype | Season | Line*Season |
| --- | --- | --- | --- |
| Pr(>WJ) length | **0.0003130685***** | **2.192106e-10***** | 0.8519637 |
| Pr(>WJ) width | 0.3348592 | **3.306155e-11***** | 0.6070456 |

**Supplementary File S8F.** ANOVA results for macroscopic ovary morphology.

|  | **Df** | **Sum Sq** | **Mean Sq** | **F value** | **P value** |
| --- | --- | --- | --- | --- | --- |
| Ovary length | 11 | 126.75 | 11.523 | 21.67 | **7.12*10^-14^*** |
| Residuals | 42 | 22.33 | 0.532 |  |  |

**Supplementary File S8G.** Kruskal-Wallis ANOVA results for macroscopic ovary morphology.

|  | **Df** |  |  | **K-W chi squared** | **P value** |
| --- | --- | --- | --- | --- | --- |
| Ovary width | 11 |  |  | 41.291 | **2.148*10^-5^***** |
| Ovary shape | 11 |  |  | 36.52 | **0.0001386***** |

**Supplementary File S8H.** ANOVA results for microscopic ovary morphology.

|  | **Df** | **Sum Sq** | **Mean Sq** | **F value** | **P value** |
| --- | --- | --- | --- | --- | --- |
| Ovary shape | 5 | 0.9253 | 0.18506 | 5.815 | **0.000907***** |
| Residuals | 27 | 0.8593 | 0.03183 |  |  |
| Ovary distal end blockiness | 5 | 0.06729 | 0.013457 | 1.685 | 0.172 |
| Residuals | 27 | 0.21567 | 0.007988 |  |  |

**Supplementary File S8I.** Kruskal-Wallis ANOVA results for microscopic ovary morphology.

|  | **Df** |  |  | **K-W chi squared** | **P value** |
| --- | --- | --- | --- | --- | --- |
| Ovary length | 5 |  |  | 20.668 | **0.0009358***** |
| Ovary width | 5 |  |  | 14.153 | **0.01467*** |
