# Supplementary material for: Targeted Editing and Phenotypic Profiling of CmOFP13 Mutants Reveal Its Role in Melon Fruit Morphogenesis
Source: Physiol Plant. 2025 Nov 29;177(6):e70641. doi: 10.1111/ppl.70641 (PMC12664293; doi:10.1111/ppl.70641)
Supplement: Supplementary file 10 — File S10: ppl70641‐sup‐0011‐FileS10.pdf. [file PPL-177-e70641-s010.pdf]

(A)

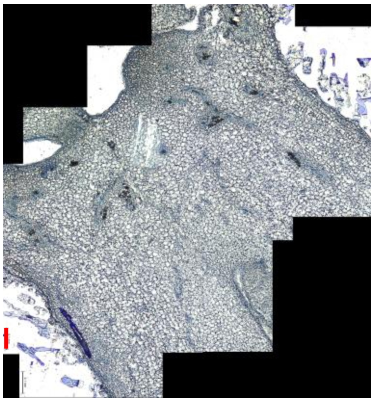

*ofp13+1*_homo F1

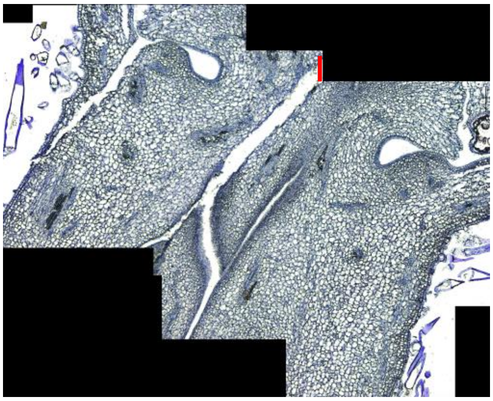

*ofp13+1*_homo F2

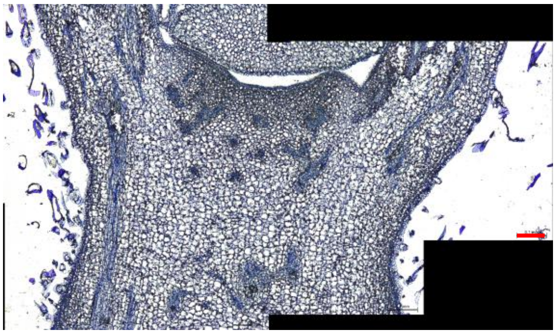

*ofp13+1*_homo F3

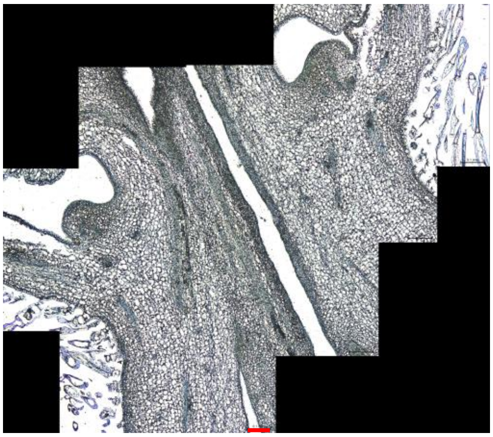

*ofp13+1*_homo F4

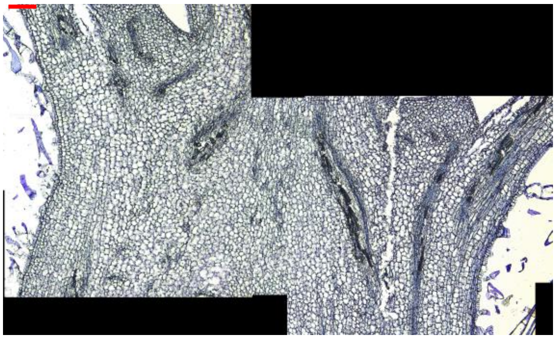

*ofp13+1*_homo F5

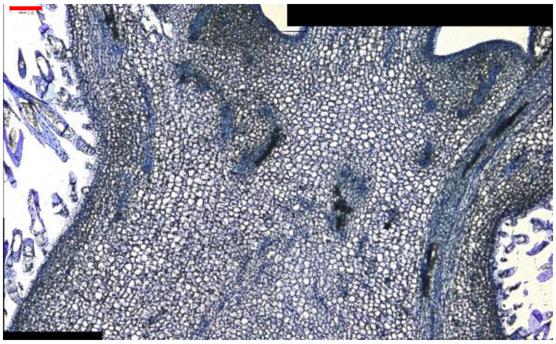

*ofp13+1*_homo F6

(B)

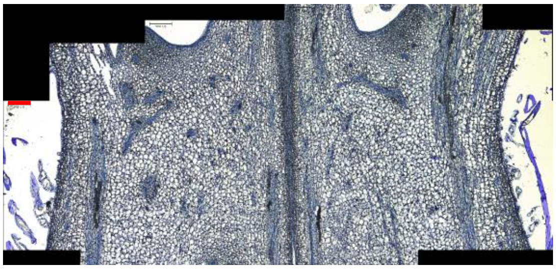

Wt (VED) F1

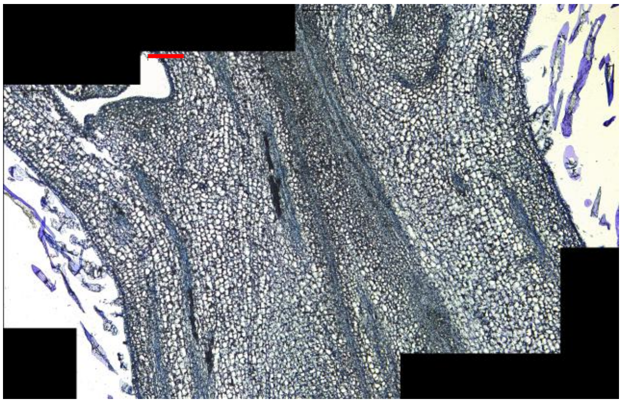

Wt (VED) F2

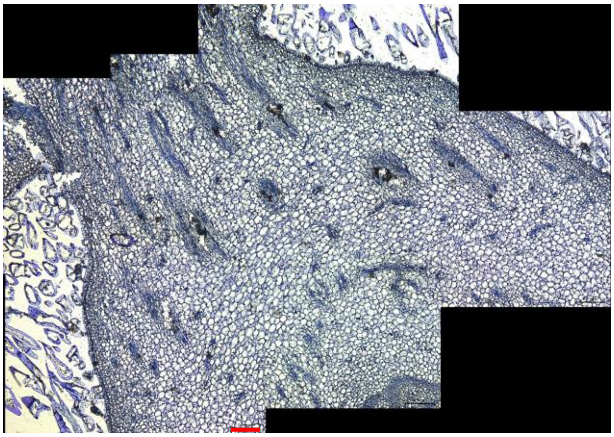

Wt (VED) F3

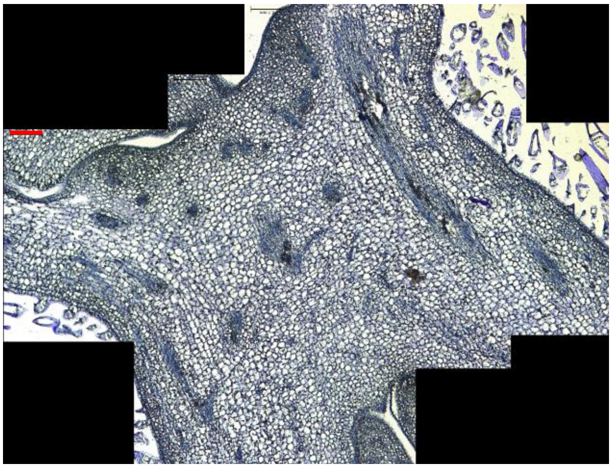

Wt (VED) F4

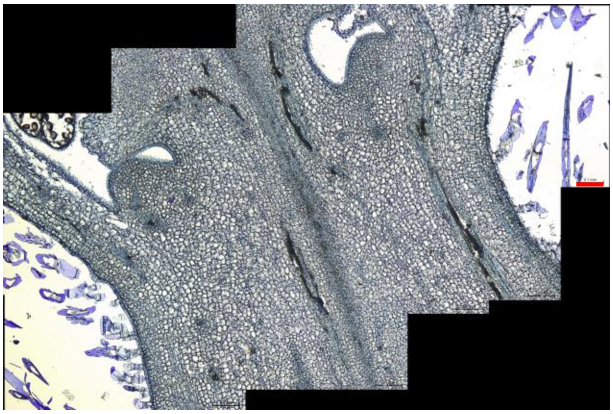

Wt (VED) F5

Supplementary File S10. Microscopy images from the distal area of flowers from edited (A) and wild-type (B) plants. Red lines length equal 0.1mm.
