# Supplementary material for: Targeted Editing and Phenotypic Profiling of CmOFP13 Mutants Reveal Its Role in Melon Fruit Morphogenesis
Source: Physiol Plant. 2025 Nov 29;177(6):e70641. doi: 10.1111/ppl.70641 (PMC12664293; doi:10.1111/ppl.70641)
Supplement: Supplementary file 11 — File S11: ppl70641‐sup‐0011‐FileS11.pdf. [file PPL-177-e70641-s008.pdf]

(A)

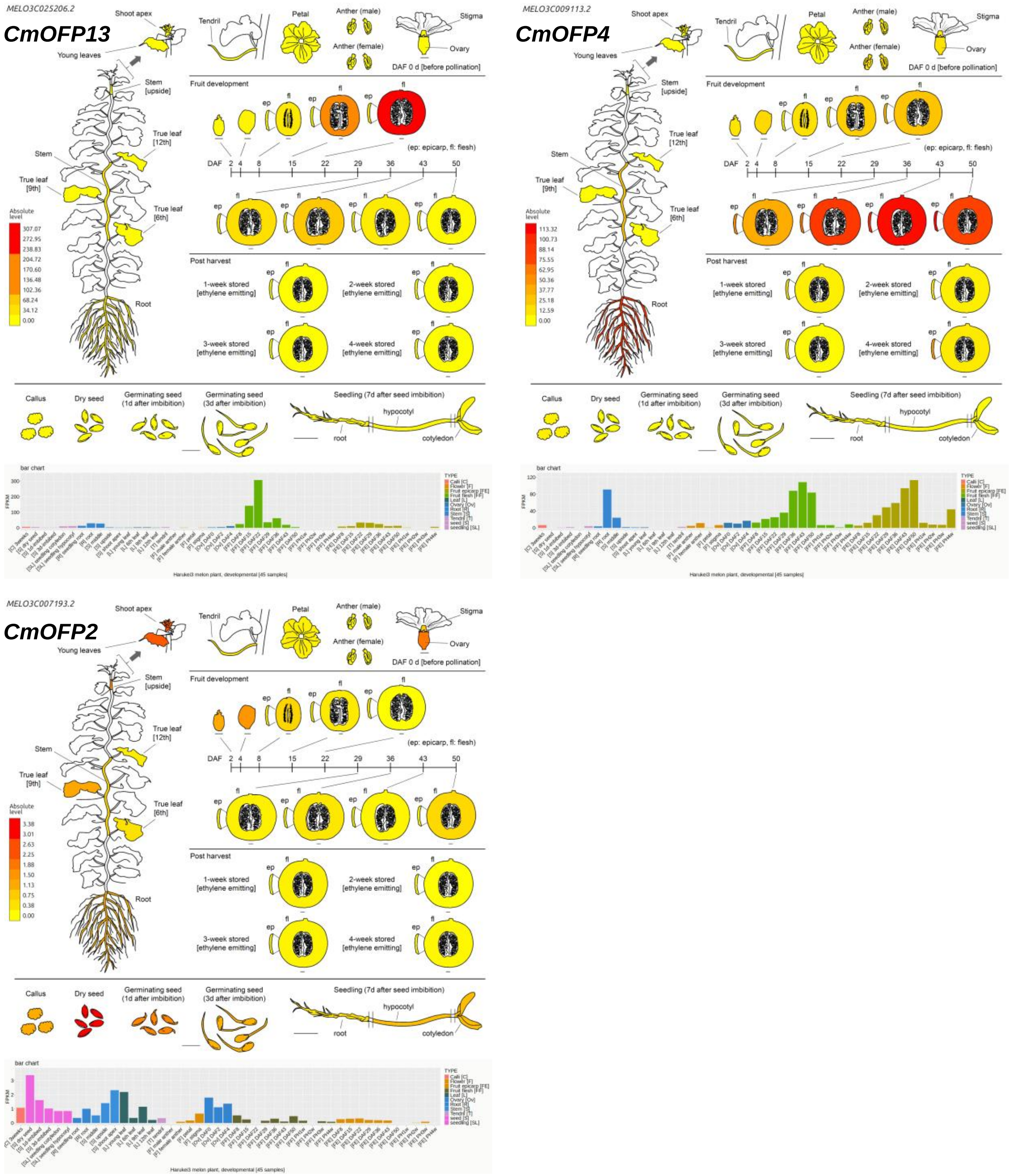

(B)

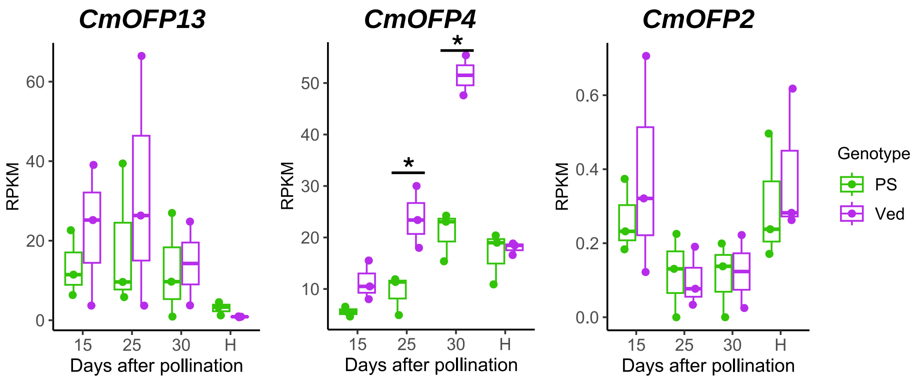

Supplementary File S11. Expression of CmOFP13, CmOFP4 and CmOFP2. (A) Expression in 'Harukei-3' from MelonetDB. (B) Expression from RNAseq in PS and VED fruits (Santo Domingo et al., 2024).
